# Development of PROTACs Targeting the Moonlighting Enzyme Nicotinamide Phosphoribosyltransferase (NAMPT) for Breast Cancer Therapy

**DOI:** 10.1021/acs.jmedchem.5c01827

**Published:** 2026-02-19

**Authors:** Ubaldina Galli, Marianna Moro, Federica Carolina Balestrero, Giorgia Colombo, Marco Koten, Benedetta Roncaglio, Armando A. Genazzani, Silvio Aprile, Alberto Massarotti, Giuseppe Orsomando, Tracey Pirali, Ambra A. Grolla

**Affiliations:** † Department of Pharmaceutical Sciences, 19050 Università degli Studi del Piemonte Orientale, Largo Donegani 2, 28100 Novara, Italy; ‡ Division of Haematology/Oncology Department of Medicine, Weill Cornell Medicine, Cornell University, 413 E 69th St, New York, New York 10021, United States; § Department of Drug Science and Technology, Università degli Studi di Torino, Via Pietro Giuria 9, 10125 Torino, Italy; ∥ Novanalitica Srls., Corso Trieste 15/a, 28100 Novara, Italy; ⊥ Department of Clinical Sciences (DISCO), Section of Biochemistry, 9294 Polytechnic University of Marche, Via Ranieri 67, 60131 Ancona, Italy

## Abstract

PROTACs (proteolysis-targeting chimeras) enable selective protein degradation through the ubiquitin–proteasome system and offer opportunities to target moonlighting proteins with nonenzymatic functions. We report the design, synthesis, and biological evaluation of NAMPT-directed PROTACs derived from our previously described inhibitor MV78 (**7**). A modular click chemistry strategy facilitated rapid assembly of a focused library by varying linker architectures and E3 ligase recruiters, with emphasis on the impact of a triazole unit. Structure–activity relationship studies revealed that eliminating the triazole from the linker and introducing an (*S*)-methyl group on the VHL ligand markedly enhanced degradation. The optimized degrader, **U42**, exhibited low nanomolar antiproliferative activity, robust intracellular and extracellular NAMPT degradation, excellent metabolic stability, favorable pharmacokinetics, and sustained efficacy in mammosphere models, three-dimensional breast cancer cultures not previously explored with NAMPT degraders. These findings highlight **U42** as a lead compound and provide strong rationale for advancing NAMPT-directed PROTACs as a therapeutic strategy in breast cancer.

## Graphical abstract

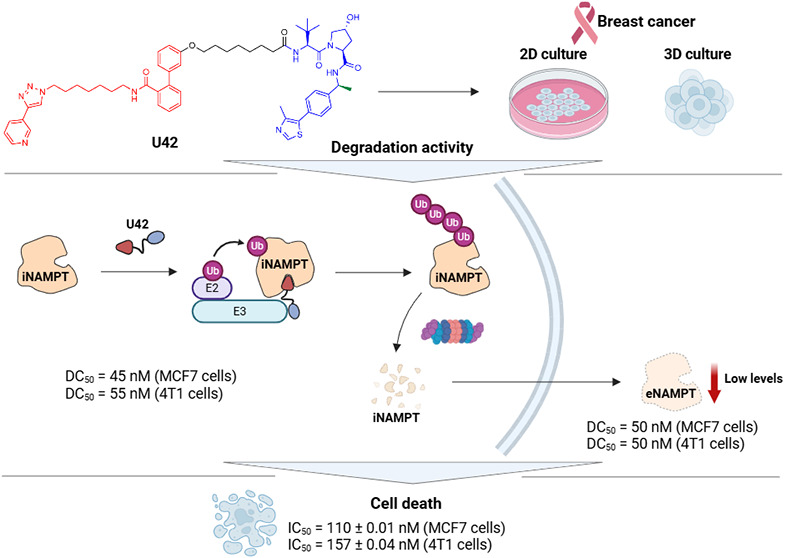

## Introduction

Moonlighting proteins exhibit multiple functions beyond their canonical enzymatic activity, often switching roles depending on cellular conditions or localizing to different subcellular compartments to perform different tasks. Extensive research has highlighted their critical involvement in cancer development and progression, making them attractive targets for drug discovery.^1^ Among these multifunctional enzymes, nicotinamide phosphoribosyltransferase (NAMPT) stands out as a “double-faced” protein existing in two forms: intracellular NAMPT (iNAMPT) which serves as the rate-limiting enzyme in the NAD^+^ salvage pathway, and the extracellular one (eNAMPT), which acts as a pro-inflammatory and tumorigenic cytokine.^2^

Tumor cells exploit iNAMPT overexpression to sustain their elevated demand for NAD^+^, essential for rapid proliferation, while eNAMPT promotes cancer progression by stimulating proliferation, angiogenesis, and other oncogenic processes.^3,4^ Additionally, iNAMPT can localize to the nucleus, particularly in tumor cells, through interaction with another moonlighting protein, GAPDH, which provides NAD^+^ at the nuclear level to support survival under conditions of oxidative stress and DNA damage.^5^

Given its multifaceted role in cancer, NAMPT has long been pursued as a therapeutic target. Several small molecule inhibitors (SMIs) have been developed to block the main salvage pathway, thereby depleting NAD^+^ and inducing apoptosis.^6,7^ To date, five NAMPT inhibitors have advanced into clinical trials: FK866 (phase II), CHS-828 (phase I), GMX-1777, a water-soluble prodrug of CHS-828 (phase I), OT-82 (phase I) and KPT-9274 (phase I), a dual PAK4/NAMPT inhibitor. However, three of these studies were terminated or withdrawn due to dose-limiting adverse effects, primarily thrombocytopenia and cardiotoxicity^8^ or poor efficacy. The limited success may stem from the inability of SMIs to interfere with the nonenzymatic functions of eNAMPT (Figure 1a), as well as their failure to modulate NAD^+^ homeostasis via feedback mechanisms.^9^

**Figure 1:**
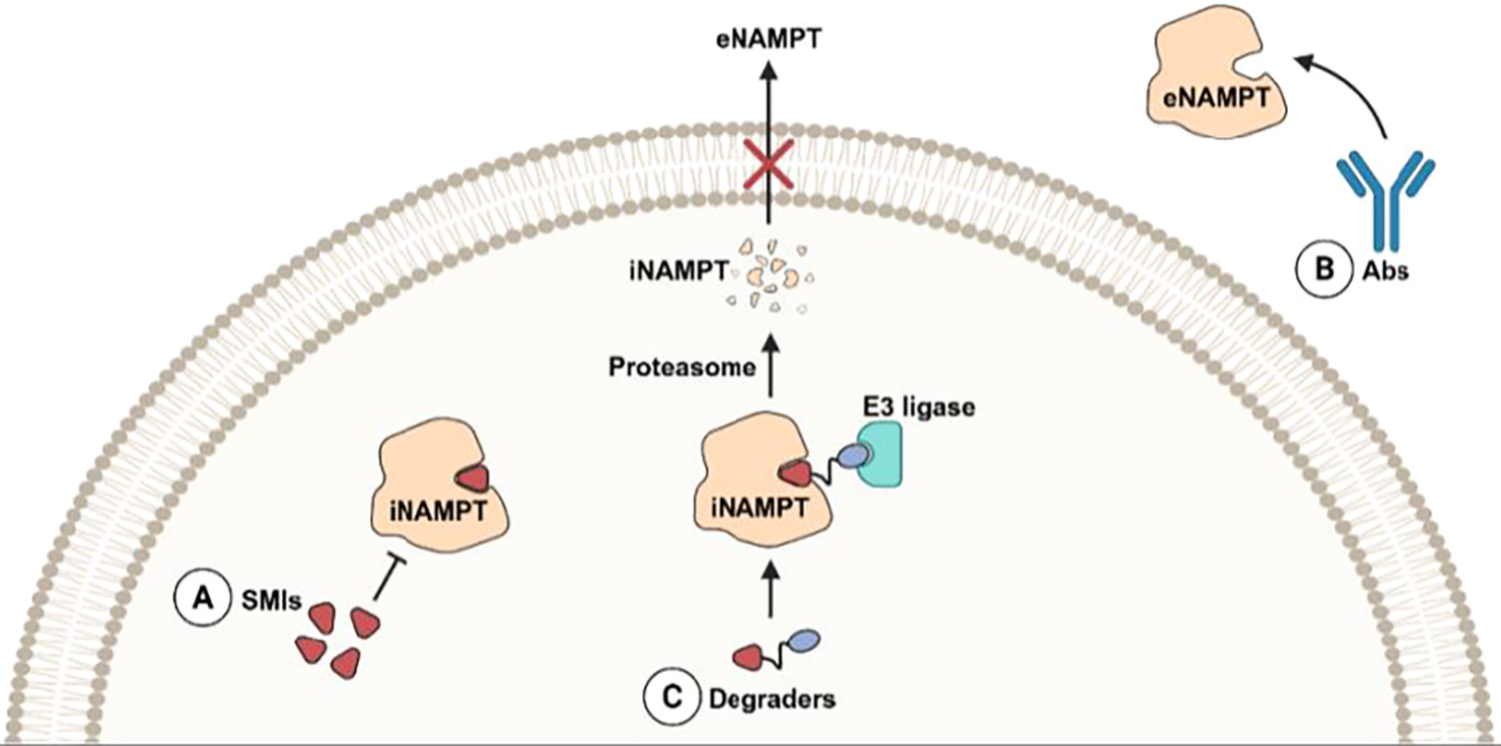
NAMPT and the main strategies to target NAMPT explored so far. NAMPT = nicotinamide phosphoribosyltransferase; iNAMPT = intracellular NAMPT; eNAMPT = extracellular NAMPT; SMIs = small molecule inhibitors; Abs = monoclonal antibodies.

Due to the limitations of conventional enzymatic inhibitors, alternative strategies have been explored in recent years,^7^ including combination therapy with chemotherapeutic agents (GMX1777 and temozolomide, phase III), dual inhibition, antibody-drug conjugates (ADCs) and monoclonal antibodies (Abs) (Figure 1b). In contrast to SMIs, Abs effectively neutralize eNAMPT,^3,10^ but lack activity against iNAMPT due to their macromolecular nature and limited cellular permeability. Therefore, there is a growing need for novel approaches able to simultaneously target both the enzymatic and nonenzymatic functions of NAMPT.

Protein degraders have recently emerged as a promising technology for the chemical knockdown of multifunctional proteins^11–13^ (Figure 1c). Among these, proteolysis targeting chimeras (PROTACs) induce the formation of a ternary complex between a protein of interest (POI) and an E3 ligase, promoting proteasomal degradation via the ubiquitin-proteasome system (UPS).^14^ On the other hand, autophagosome-tethering compounds (ATTECs) induce POI degradation through the autophagy-lysosomal pathway (ALP).^15^ Preclinical data suggest that protein degraders may outperform SMIs, primarily because the depletion of iNAMPT levels also leads to a reduction in eNAMPT secretion, thereby disrupting both enzymatic and nonenzymatic functions.^16^ Additional advantages of PROTACs over SMIs include their catalytic mode of action, which enables lower dosing and potentially reduces off-target toxicity and side effects, as well as improved target selectivity, reduced risk of mutation-driven resistance and prolonged pharmacodynamic effects.

To date, only a few PROTACs targeting NAMPT have been described (Figure 2). Sheng et al. reported von Hippel-Lindau (VHL)-recruiting PROTACs bearing the thiourea-^17^ and urea-derived^18^ inhibitors as POI ligands [PROTAC A7 (**1**) and B3 (**2**) Figure 2]. These degraders showed efficacy in murine models of colorectal and ovarian cancer, respectively. Building on the most promising degraders of the series, the same authors reported a drugtamer-PROTAC conjugation strategy for targeted delivery.^19^ In the same year, a photoswitchable PROTAC was developed, enabling light-dependent regulation of NAMPT and NAD^+^ levels and allowing optical control of antitumor activity in biological systems.^20^ Given that NAMPT degradation under physiological conditions is mainly mediated via the lysosomal pathway, the same team reported the first NAMPT-targeting ATTEC^21^ [A3 (**3**) Figure 2] by retaining the thiourea warhead and by replacing the E3 ligase recruiter with ispinesib, a ligand for LC3. Using FK866 inhibitor as the POI ligand, both cereblon (CRBN)-^22^ and VHL-^23^ recruiting PROTACs have been developed [PROTAC SIAIS630120 (**4**) and C5 (**5**) Figure 2]. Among these, the degrader LYP-8 (**6**) (Figure 2) discovered by Liu et al. demonstrated efficacy in a colon cancer model.^16^ Finally, by incorporating the fluorescent compound M049-0244 as the NAMPT-binding moiety, a theranostic PROTAC was described, enabling real-time detection and tracking of NAMPT degradation in living cells.^24^

**Figure 2:**
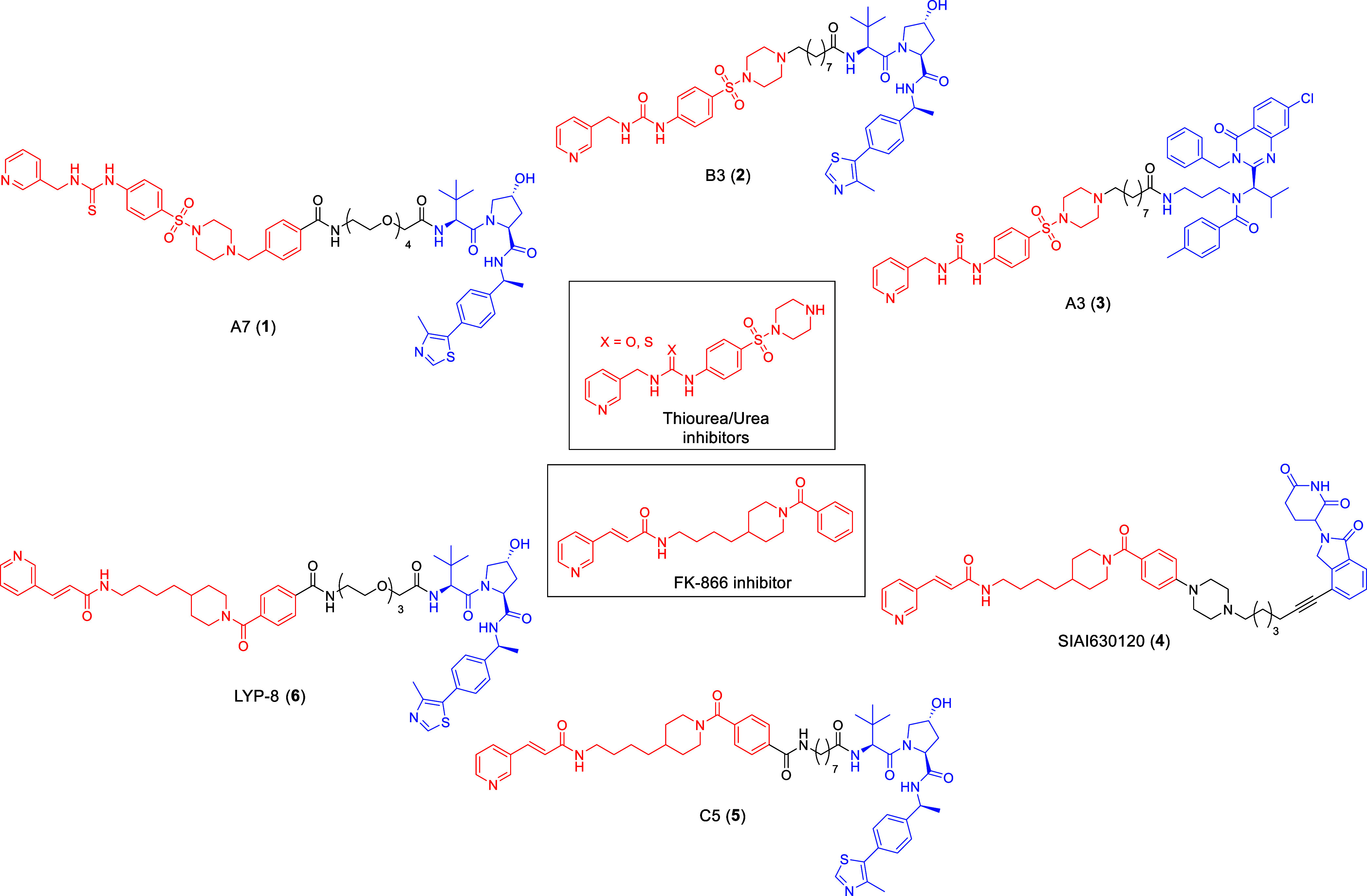
Structure of representative inhibitors and corresponding degraders (PROTACs and ATTEC).

In this study, we report the rational design, synthesis, and screening of a new series of PROTACs based on MV78 (**7**), a NAMPT inhibitor previously discovered by our group.^25^ The design strategy relied on click chemistry for modular synthesis^26,27^ (Figure 3). A systematic SAR study was performed to explore the impact of the E3 ligase selection (CRBN vs VHL), as well as the linker length and composition, with particular emphasis on the role of the embedded triazole ring in modulating degradation efficiency. We have also studied the pharmacokinetic properties of our lead compound as well as of its analogue featuring an additional (*S*)-methyl group at the benzylic position of the VHL ligand. Finally, we have investigated the efficacy of our most promising PROTAC in a breast cancer mammosphere model, a physiologically relevant system in which NAMPT-targeting PROTACs have not been studied to date.

**Figure 3:**
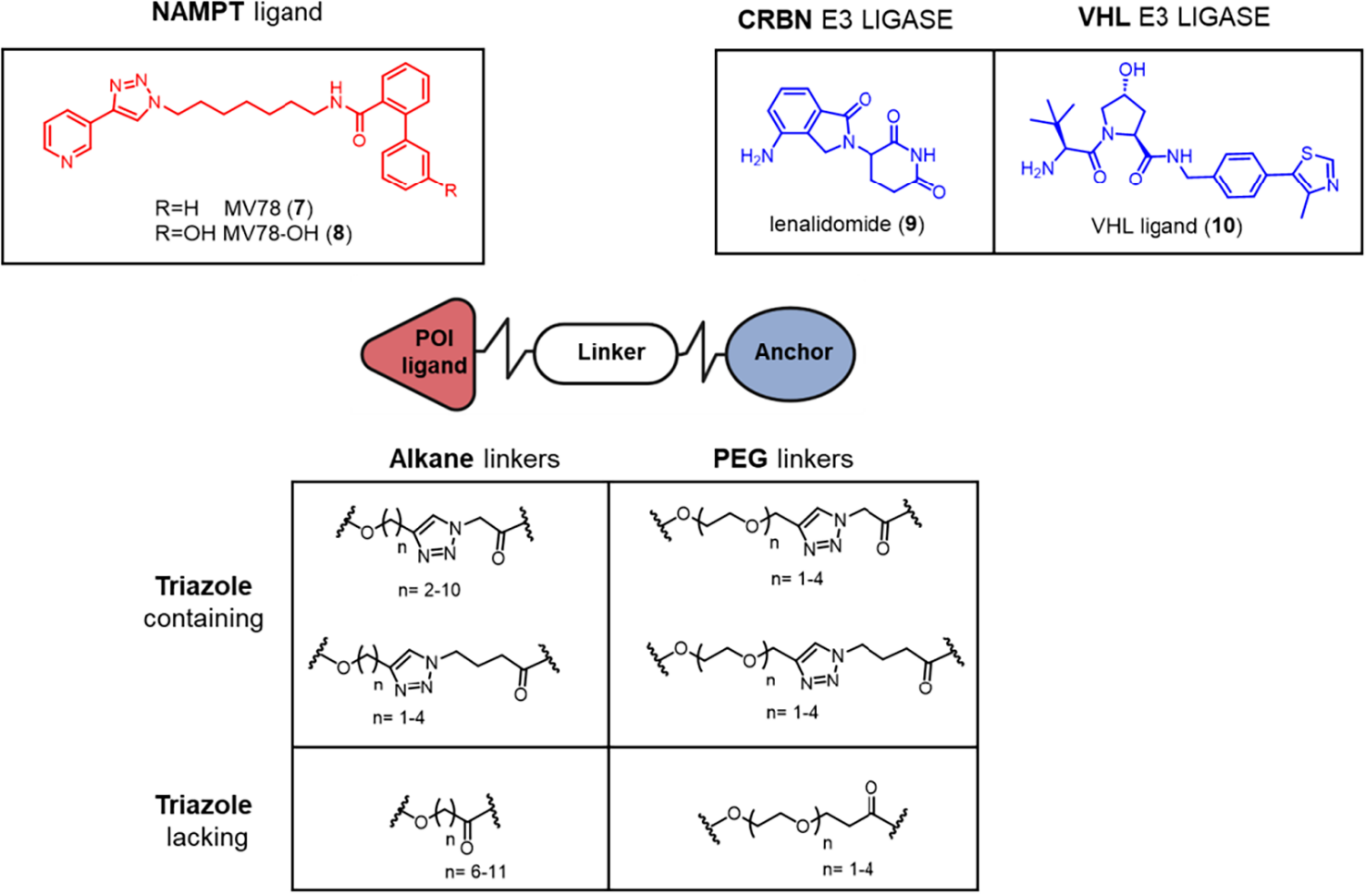
Design strategy for MV78-based NAMPT-targeting PROTACs.

## Results and Discussion

### Rational Design of NAMPT-Targeting PROTACs

For the design of PROTAC series, MV78 (**7**) (Figure 3) was selected as NAMPT-binding ligand. This inhibitor displays potent NAMPT inhibitory activity (IC_50_ = 18.2 nM), reducing cell proliferation at low nanomolar concentrations (IC_50_ in SH-SY5Y = 5.8 nM) and shows good *in vitro* metabolic stability (88% residual substrate in human liver microsomes after 60 min).

Based on the docking pose previously described,^25^ the terminal phenyl ring of the tail group, and particularly the *meta*-position, was identified as the optimal site for linker attachment, being oriented toward a solvent-exposed cavity. Accordingly, MV78-OH (**8**) (Figure 3), bearing a hydroxyl group at this position, was employed as the POI ligand.

To recruit E3 ligases, we used lenalidomide (**9**) for CRBN and an hydroxyproline-containing ligand (**10**) for VHL (Figure 3). These ligands were chosen as they are the best-characterized E3 ligases in targeted protein degradation, broadly expressed, supported by well-established tool ligands, and with the strongest clinical and preclinical track records.

A range of alkane and poly­(ethylene glycol) (PEG)-based linkers of varying lengths were evaluated to balance physicochemical and pharmacokinetic properties (Figure 3). Alkane linkers reduce polarity and the number of hydrogen-bond acceptors, favoring permeability and metabolic stability, whereas PEG linkers can improve aqueous solubility and, in certain cases, enhance effective permeability despite higher polarity. To account for this context-dependency, we explored both families systematically, generating matched alkane/PEG series in line with best practices for PROTAC optimization.

In our design strategy, we initially incorporated a 1,2,3-triazole moiety within the linker to exploit the efficiency of click chemistry and the well-known robustness of the triazole ring, which is known to resist hydrolysis, oxidation, and metabolic cleavage. Triazole-based linkers are widely used in degrader libraries and have yielded active PROTACs, which supported our choice at the outset. In particular, clickable linkers bearing a triazole ring in different positions relative to the E3 ligase ligand were synthesized, as well as linkers lacking the heterocycle, to assess the influence of the triazole and its positioning on degradation efficiency (Figure 3). This approach complements previous studies that highlighted the role of the triazole moiety in modulating both *in vitro* activity^28^ and physicochemical properties.^29^

Two points of attachment were investigated: an ether linkage connecting the POI ligand to the linker and a secondary amide group bridging the linker to the E3 ligase binder.

### Chemistry of NAMPT-Targeting PROTACs and Biological Identification of the Lead Compounds

For the synthesis of the PROTACs, the NAMPT inhibitor MV78 (**7**) was functionalized with a phenol group at the *meta*-position of the terminal phenyl ring. The synthesis of MV78-OH (**8**) is outlined in Scheme 1. 7-Azidoheptanoic acid (**11**), obtained from azidation of 7-bromoheptanoic acid, was subjected to the copper­(I)-catalyzed azide–alkyne cycloaddition (CuAAC) with 3-ethynylpyridine in the presence of CuSO_4_·5H_2_O and sodium ascorbate, affording the triazole intermediate **12**. The carboxylic acid was then esterified under Fisher conditions and the resulting ester **13** was reduced to alcohol **14** using lithium aluminum hydride. The hydroxyl group of **14** was converted into azide **15** via *one-pot* method with DPPA, DBU and NaN_3_. The azide was subsequently reduced to the corresponding amine **16** via a Staudinger reaction.^25^ Amine **16** was then coupled with 2-iodobenzoic acid using EDCI as a coupling agent to afford amide **17**. Finally, a Suzuki coupling with 3-hydroxyphenylboronic acid afforded NAMPT ligand MV78-OH (**8**).

**Scheme 1:**
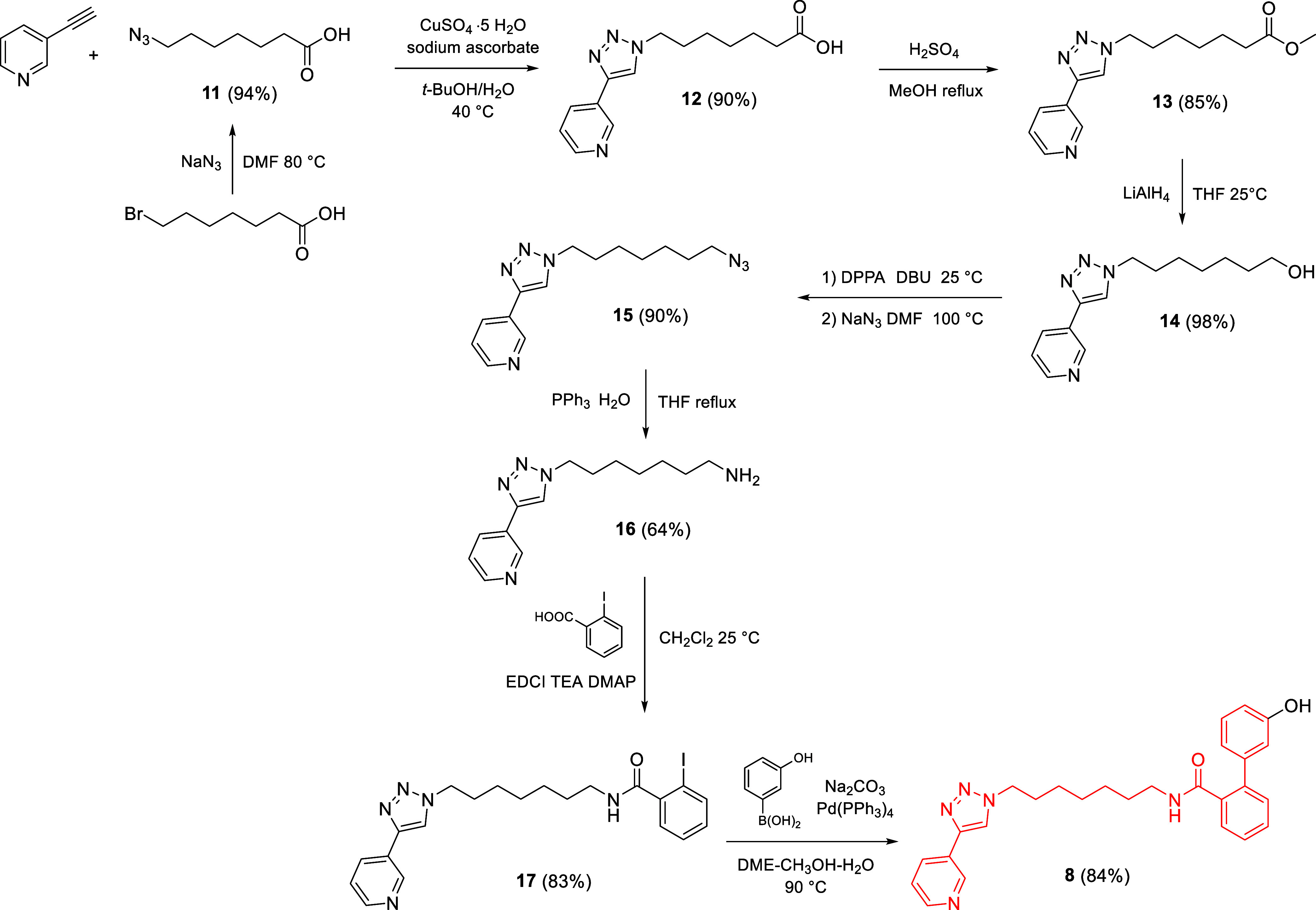
Synthesis of MV78-OH (**8**) NAMPT Ligand

The synthetic route for CRBN-recruiting PROTACs incorporating the triazole ring within alkane and PEG linkers is depicted in Scheme 2. Terminal alkynes of varying length **18–26** and **27–30** were tosylated at the hydroxyl group to afford intermediates **31–39** and **40–43**. These were then reacted with MV78-OH (**8**) via Williamson reaction under basic conditions to yield ethers **44–52** and **53–56**, respectively. Lenalidomide (**9**) was coupled with 2-bromoacetyl chloride to generate the bromo derivative **57**, which was subsequently converted to azide **58** via nucleophilic substitution with sodium azide. The final PROTACs were obtained through CuAAC reaction between the POI ligand-bearing alkynes **44–52** and **53–56** and the CRBN ligand-bearing azide (**58**) furnishing the PROTACs **U1–9** and **U10–13**, respectively.

**Scheme 2:**
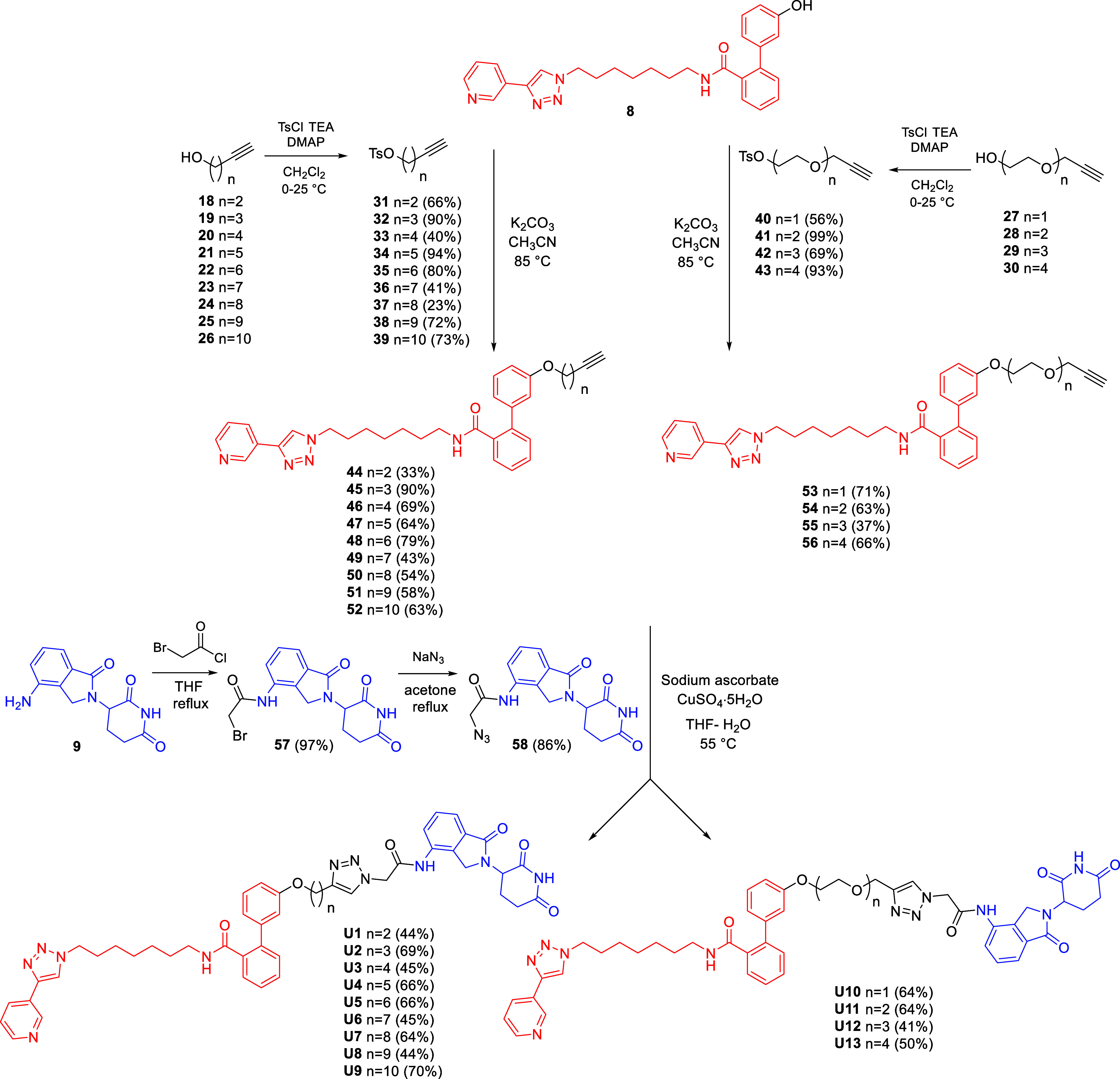
Synthesis of CRBN-Based NAMPT Targeting PROTACs with Triazole-Containing Alkane and PEG Linkers **U1–13**

VHL-recruiting PROTACs incorporating the triazole within alkane and PEG linkers were prepared as outlined in Scheme 3. Ethyl 4-bromobutanoate underwent a nucleophilic substitution with sodium azide to afford azide **59**, which was hydrolyzed under basic conditions to yield carboxylic acid **60**. This intermediate was coupled with VHL ligand (**10**) using HATU as the coupling agent, providing the azido-functionalized anchor **61**. The target PROTACs **U14–17** and **U18–21** were prepared via CuAAC by reacting azide **61** with alkyne **62**, obtained through the alkylation of MV78-OH (**8**) with propargyl bromide, and the previously described alkynes **44–46** and **53–56**, respectively.

**Scheme 3:**
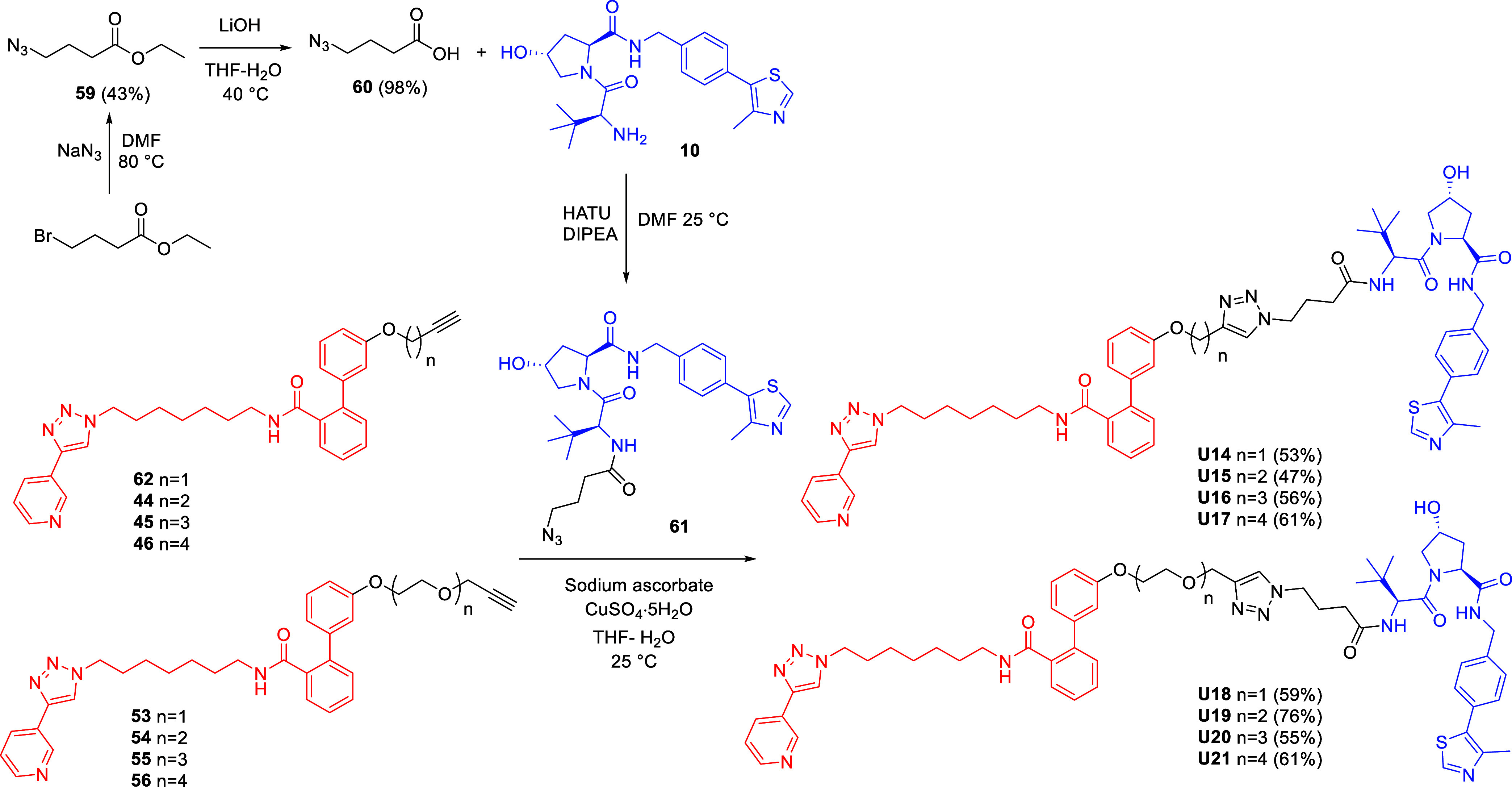
Synthesis of VHL-Based NAMPT Targeting PROTACs with Triazole-Containing Alkane and PEG Linkers **U14–21**

Compounds **U1–21** were biologically evaluated in cell-based assays to assess their ability to cross the plasma membrane as well as to target NAMPT. Given the essential role of NAMPT in NAD^+^ biosynthesis and cell viability, we investigated the cytotoxic effects of the synthesized compounds. MCF7 human breast cancer cells were treated with 1 or 10 μM of **U1–21** for 72 h, and cell viability was assessed using the MTT assay. NAMPT protein levels were also examined by Western blot analysis after 18 h of treatment at 1 μM.

As shown in Table 1, most compounds reduced cell viability by more than 50% at 10 μM concentration. However, none of them significantly decreased NAMPT protein levels, indicating that their activity is likely due to enzymatic inhibition rather than targeted protein degradation (Figure S1A).

**Table 1:**
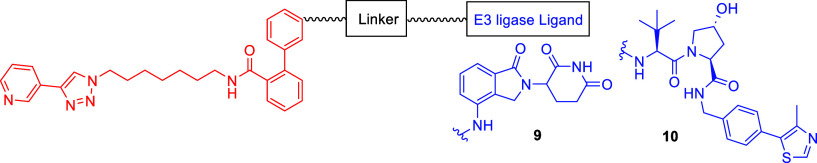

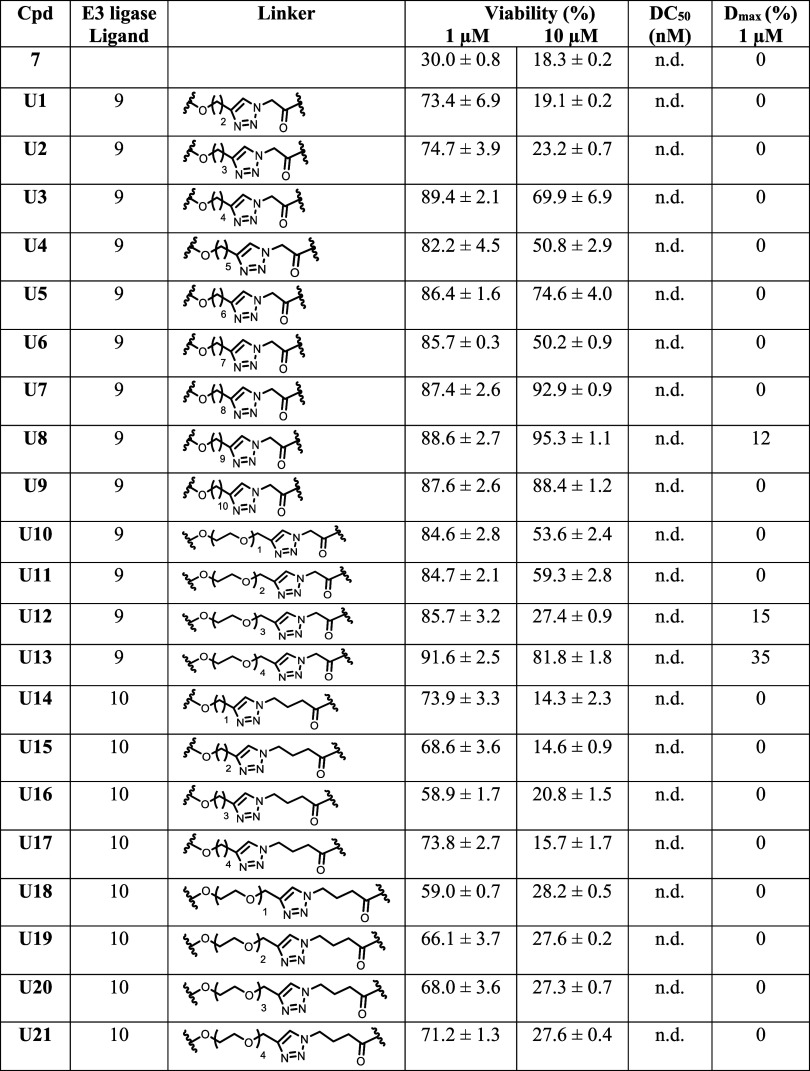
Drug Screening Based on: (1) Cell Viability Assessed by MTT Assay after 72 h on MCF7 Cells Treated with Compound **7** and **U1–21** at the Dose of 1 and 10 μM; (2) Western Blot Analysis Performed after 18 h on MCF7 Cells Treated with Compound **7** and **U1**–**21** at the Dose of 1 μM^a^

a n.d. = not determined.

Based on these findings, we proceeded to synthesize VHL- and CRBN-based PROTACs featuring PEG linkers lacking the triazole ring. These compounds were prepared as outlined in Scheme 4. MV78-OH (**8**) was reacted via a Williamson reaction with tosylated intermediates **67–70**, obtained from the corresponding alcohols **63–66**, to yield ethers **71–74**. The *tert*-butyl ester moieties were then hydrolyzed under acid conditions to afford the corresponding carboxylic acids **75–78**. Final coupling with the VHL ligand (**10**) and lenalidomide (**9**) provided the target PROTACs **U22–25** and **U26–29**, respectively.

**Scheme 4:**
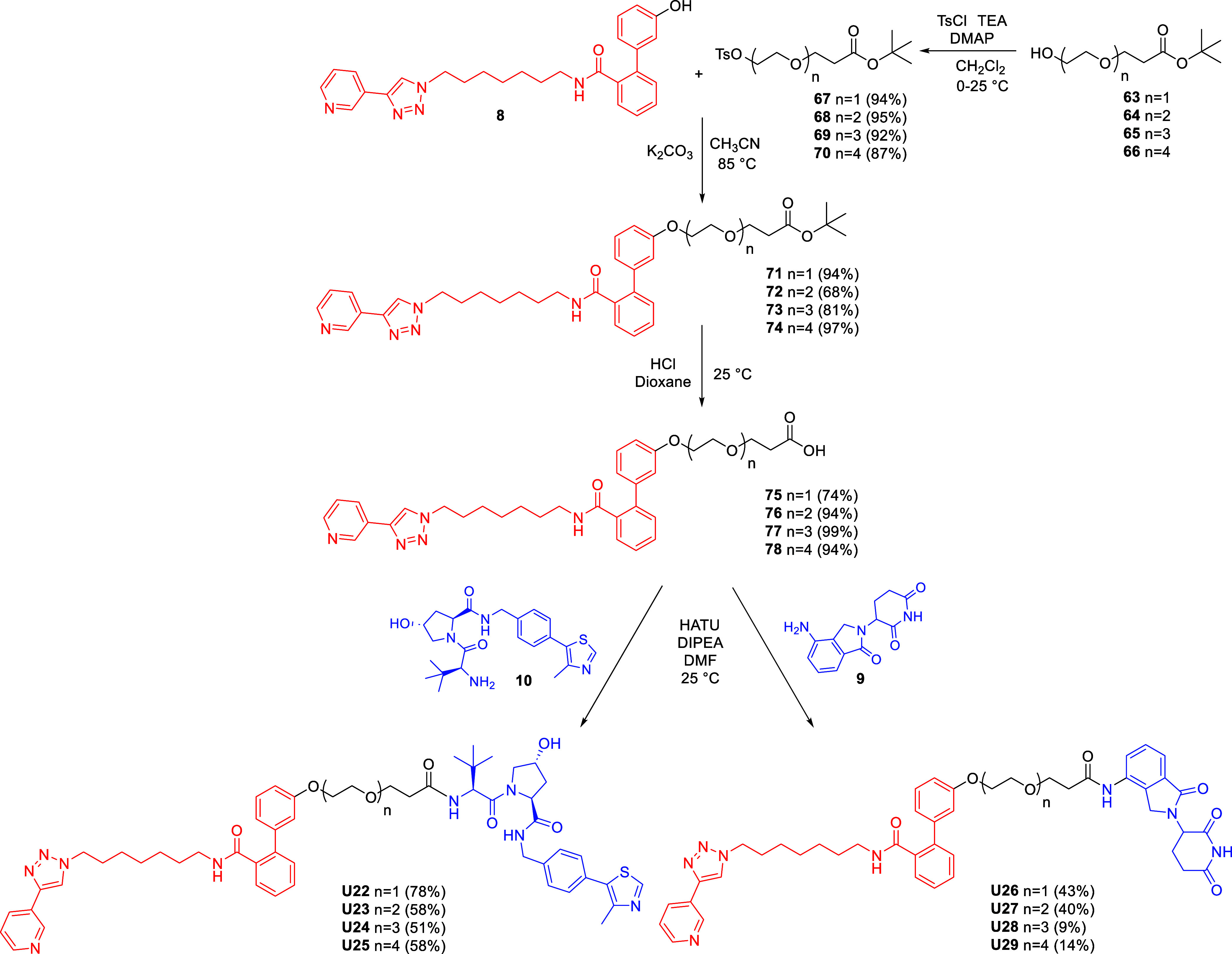
Synthesis of VHL and CRBN-Based NAMPT Targeting PROTACs with Triazole-Lacking PEG Linkers **U22–29**

The final series of PROTACs recruiting VHL and CRBN and bearing alkane linkers was prepared as depicted in Scheme 5. MV78-OH (**8**) was reacted via Williamson etherification with bromo methyl esters **85–90**, which were prepared from the corresponding acids **79–84** under Fisher esterification conditions. The resulting esters **91–96** were then hydrolyzed under basic conditions and coupled with the VHL ligand (**10**) and lenalidomide (**9**) to afford the target degraders **U30–35** and **U36–41**, respectively.

**Scheme 5:**
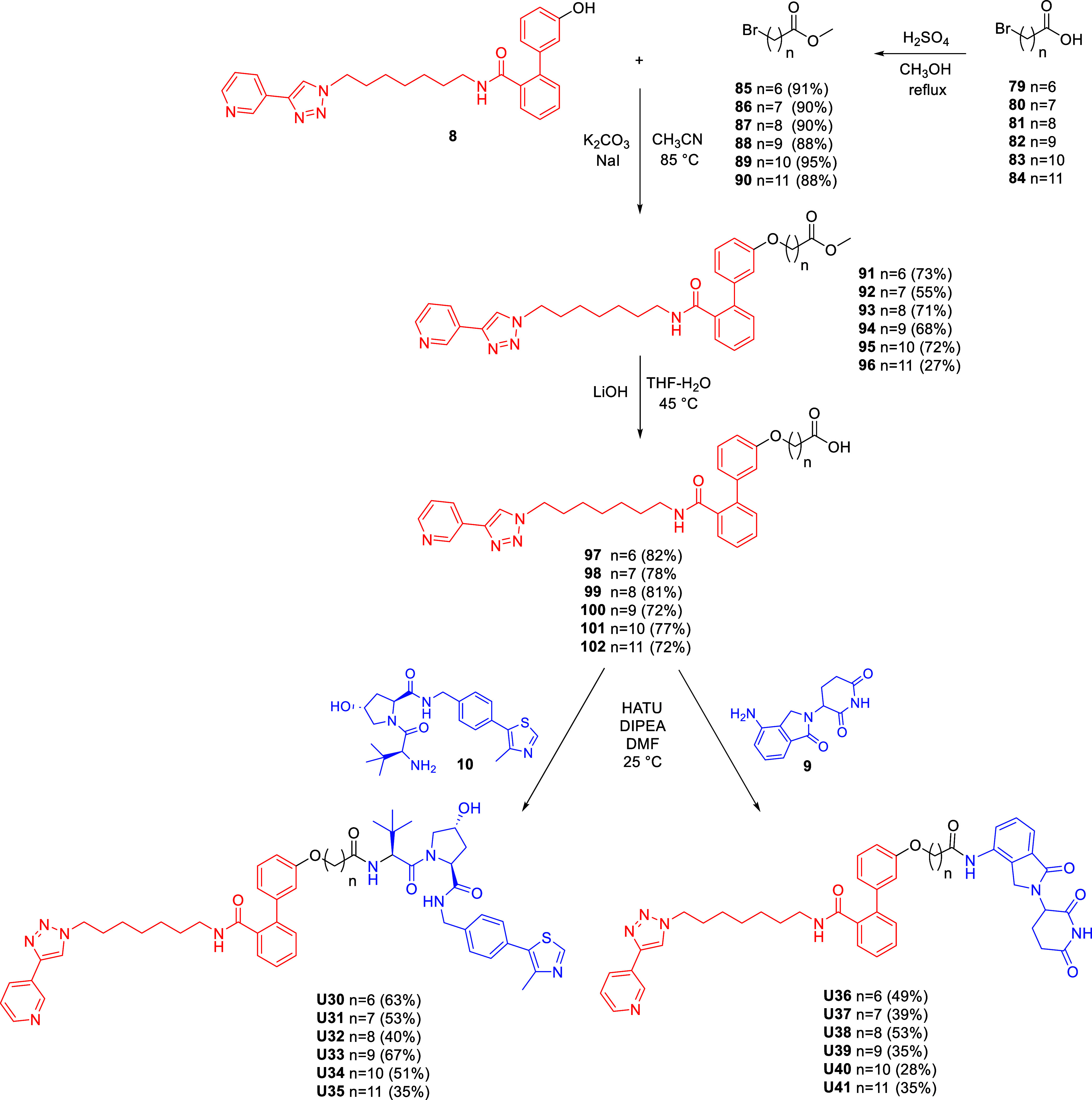
Synthesis of VHL and CRBN-Based NAMPT Targeting PROTACs with Triazole-Lacking Alkane Linkers **U30–41**

Next, we evaluated compounds **U22–41** for their biological activity using the same experimental setup described above. As shown in Table 2 (and Figure S1A), among the compounds lacking a triazole group in the linker, two (**U23** and **U31**) emerged as both potent cytotoxic agents and effective NAMPT degraders. To further optimize the degradation assay conditions, we performed a time-course study using **U23**, testing exposure times from 2 to 24 h. This experiment confirmed that 18 h is the optimal time point to assess the degradation activity of our PROTACs (Figure S1B).

**Table 2:**
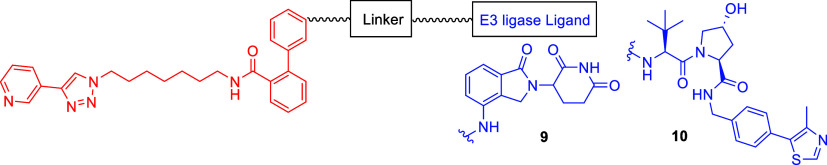

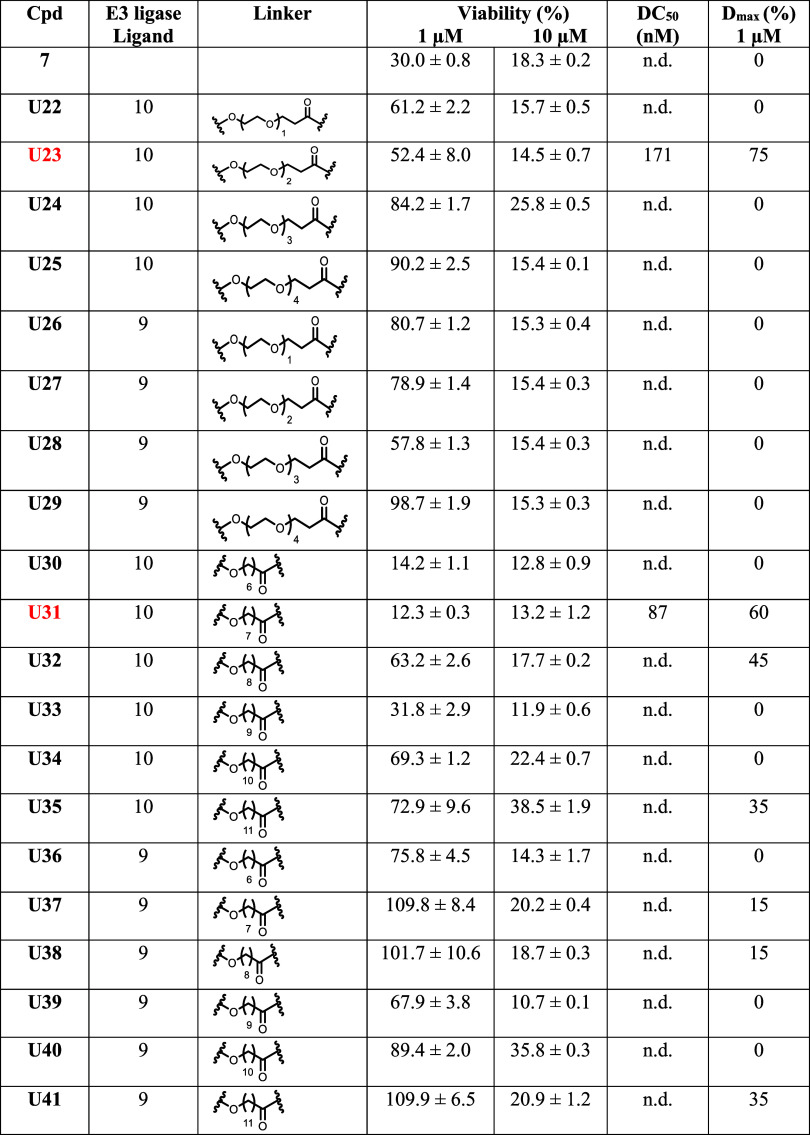
Drug Screening Based on: (1) Cell Viability Assessed by MTT Assay after 72 h on MCF7 Cells Treated with Compound **7** and **U22–41** at the Dose of 1 and 10 μM; (2) Western Blot Analysis Performed after 18 h on MCF7 Cells Treated with Compound **7** and **U22–41** at the Dose of 1 μM^a^

a n.d. = not determined.

Furthermore, we performed dose–response experiments for **U23** and **U31**, treating cells with concentrations ranging from 30 nM to 3 μM for 18 h (Figure 4) and measuring NAMPT degradation. These assays were conducted on MCF7 cells and on a more invasive and aggressive clone of 4T1 triple-negative breast cancer cells (4T1 clone 5).^30^ Of note, both compounds exhibited a Hook effect at concentrations above 1 μM. DC_50_ values, calculated by excluding the concentrations affected by the Hook effect, were 171 and 107 nM for **U23** and **U31**, respectively, in MCF7 cells (Figure 4A,B), and 500 and 350 nM for **U23** and **U31**, respectively, in 4T1 cells (Figure 4C,D). These results highlight **U31** as the most potent NAMPT degrader among the VHL-based NAMPT targeting PROTACs bearing triazole-free alkane linkers.

**Figure 4:**
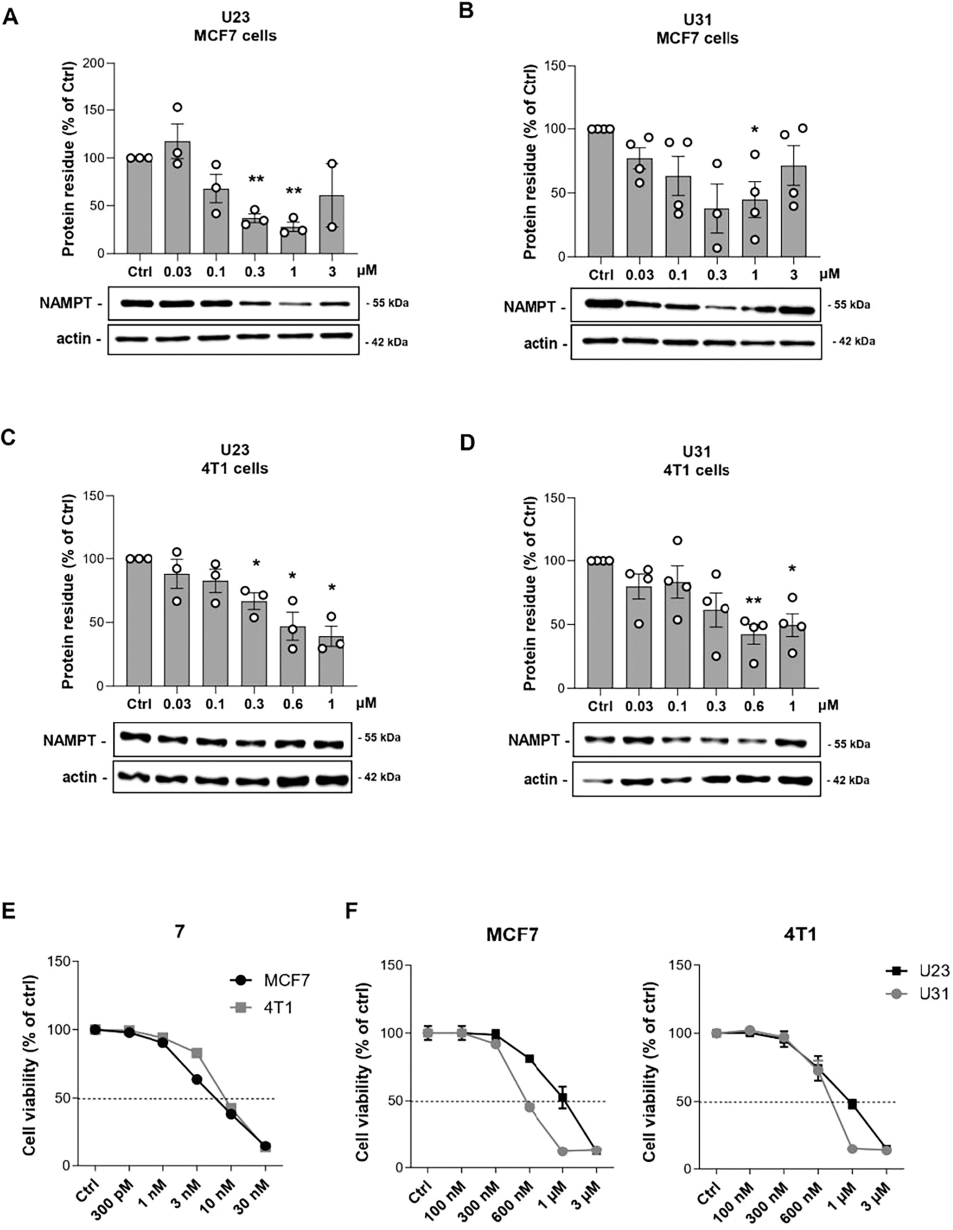
Representative images and quantification of NAMPT degradation on MCF7 cells using a dose curve of **U23** (**A**) and **U31** (**B**) for 18 h. Mean ± SEM of at least 3 independent experiments. * *p* < 0.05, ** *p* < 0.01 by unpaired parametric *t* test. Representative images and quantification of NAMPT degradation on 4T1 cells using a dose curve of **U23** (**C**) and **U31** (**D**) for 18 h. Mean ± SEM of at least 3 independent experiments. * *p* < 0.05, ** *p* < 0.01 by unpaired parametric *t* test. (**E**) Cell viability performed at 72 h on MCF7 and 4T1 cells treated with a dose curve of **7**. Mean ± SEM of at least 3 independent experiments. (**F**) Cell viability performed at 72 h on MC**F**7 (left) and 4T1 (right) cells treated with a dose curve of **U23** and **U31**. Mean ± SEM of at least 3 independent experiments.

As expected, both **U23** and **U31** retained cytotoxic activity but with lower potency compared to the parent compound **7** (IC_50_ of 7.0 ± 0.02 nM in MCF7 and of 9.6 ± 0.03 nM in 4T1, Figure 4E). The IC_50_ values in MCF7 cells were 1.03 ± 0.06 μM for **U23** and 550 ± 0.04 nM for **U31** (Figure 4F), while in 4T1 cells the values were 1.5 ± 0.94 μM and 687 ± 0.03 nM, respectively (Figure 4F). These data further confirm that **U31** is not only the most potent NAMPT degrader, but also the most effective cytotoxic agent among the synthesized compounds.

### Validation and Optimization of the Lead Compounds

To verify the E3-ligase-based mechanism of action of our PROTACs, we assessed **U31** in the presence or absence of the proteasome inhibitor bortezomib (300 nM), in both MCF7 and 4T1 cells. In both cell lines, proteasome inhibition prevented **U31**-mediated NAMPT degradation, thereby supporting our hypothesis (Figure 5A,B).

**Figure 5:**
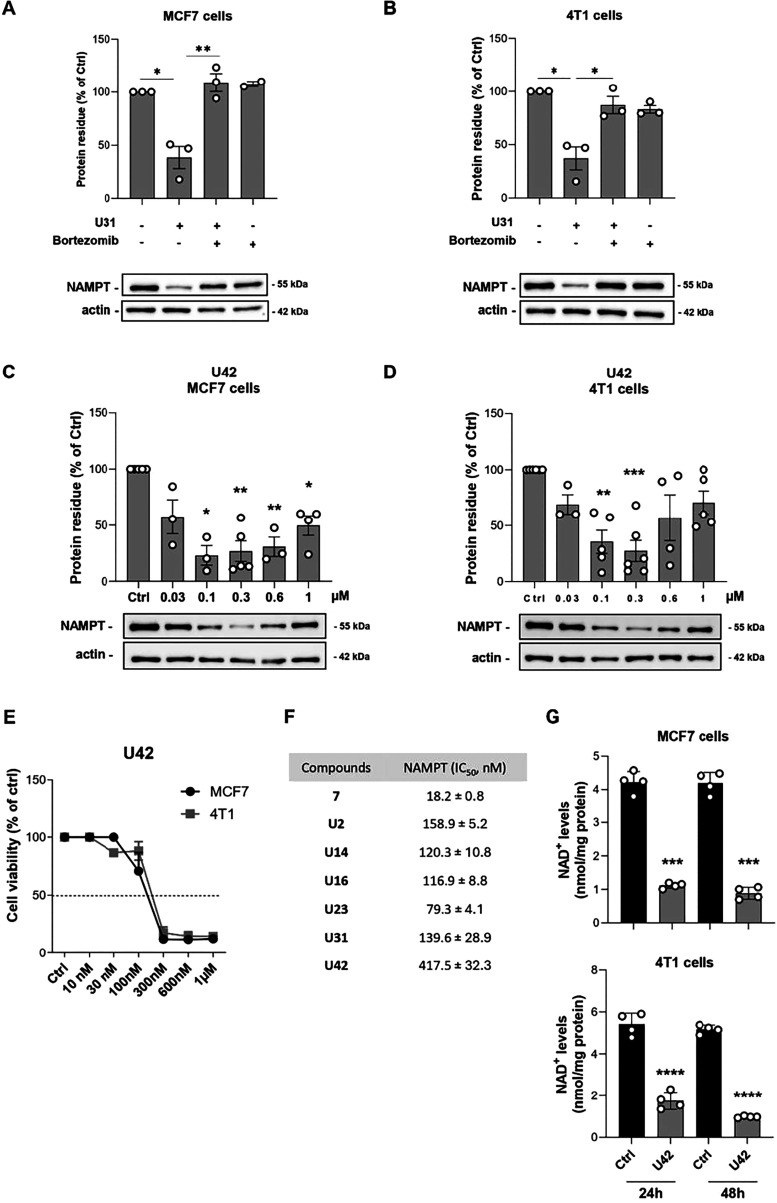
Representative image and quantification of NAMPT degradation in both MCF7 (**A**) and 4T1 cells (**B**) treated for 18 h respectively with **U31** (300 nM), bortezomib (300 nM) or the combination of the two. Mean ± SEM of 3 independent experiments. * *p* < 0.05, ** *p* < 0.01 by unpaired parametric *t* test. Representative image and quantification of NAMPT degradation in both MCF7 (**C**) and 4T1 cells (**D**) treated with a dose curve of **U42** for 18 h. Mean ± SEM of 3 independent experiments. * *p* < 0.05, ** *p* < 0.01, *** *p* < 0.001 by unpaired parametric *t* test. (**E**) Cell viability performed at 72 h of MCF7 and 4T1 cells treated with a dose curve of **U42**. Mean ± SEM of at least 3 independent experiments. (**F**) NAMPT inhibitory activity with the lead compound **7** and the most representative PROTACs: **U2**, **U14**, **U16**, **U23**, **U31**, **U42**. Data are expressed as the means of three independent experiments ± standard deviation. (**G**) Intracellular NAD^+^ levels in MCF7 (up) and 4T1 cells (down) treated for 24 and 48 h **U42** (300 nM). Mean ± SEM of 4 independent experiments. *** *p* < 0.001, **** *p* < 0.0001 by unpaired parametric *t* test.

It has been shown that the stereoselective introduction of the (*S*)-methyl group at the benzylic position of VHL ligand **10** significantly enhances its binding affinity to the VHL-E3 ligase.^31^ We have therefore prepared **U42**, the methylated derivative of **U31**, by coupling the carboxylic acid **98** with the (*S*)*-*methyl VHL ligand **103** (Scheme 6).

**Scheme 6:**
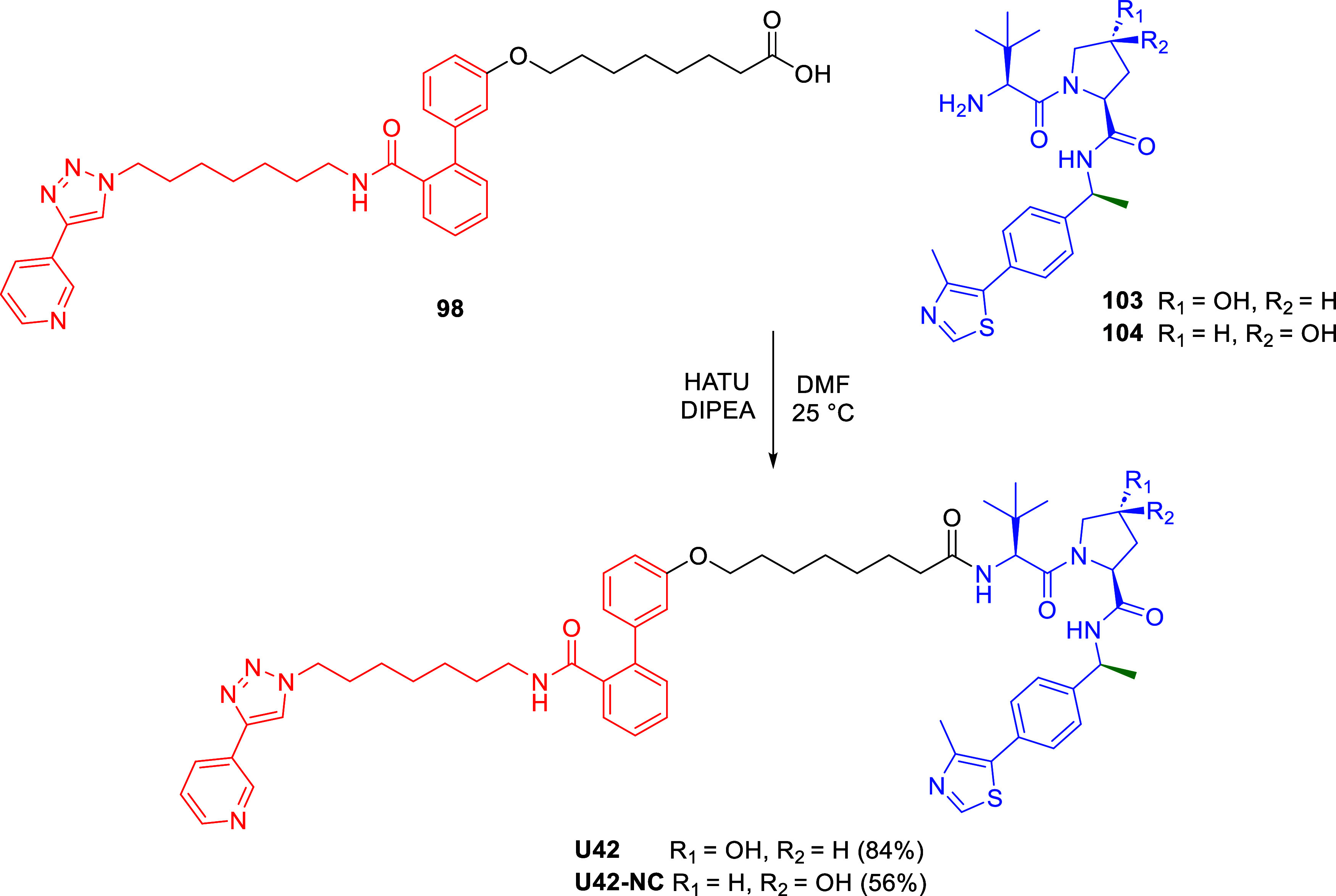
Synthesis of (*S*)-Methyl VHL Ligand-Based PROTAC **U42** and Negative Control **U42-NC**

The DC_50_ values obtained from the dose–response curve (30 nM-1 μM) confirmed the increase in degradation efficiency, **U42** was more potent than **U31** in degrading NAMPT. Specifically, the DC_50_ was 45 nM in MCF7 cells and 55 nM in 4T1 cells (Figure 5C,D). Consequently, **U42** also more effective in inducing cell death, with an IC_50_ of 110 ± 0.01 nM in MCF7 cells and 157 ± 0.04 nM in 4T1 cells (Figure 5E).

Overall, these data suggest that triazole-containing linkers compromise NAMPT degradation, as evidenced by the pronounced difference in activity between **U31/U42** and **U14**, which share the same linker length (nine atoms) but exhibit opposite degradation outcomes. In the absence of structural or biophysical insights into NAMPT PROTACs, we hypothesize that the triazole imparts excessive rigidity, thereby restricting ternary complex stabilization. These observations are therefore most appropriately interpreted within the empirical, trial-and-error framework that still characterizes PROTAC optimization. In this regard, we decided to investigate in depth the impact of triazole within the linker by integrating experimental evidence from cells and recombinant proteins with molecular modeling, as shown in the following sections.

First, to demonstrate that our best PROTAC worked by specifically inhibiting NAMPT, we evaluated the inhibitory activity of **U42** on recombinant NAMPT using an enzymatic coupled assay. In this analysis, we also included a selection of other representative PROTACs: **U2**, **U16**, **U14**, **U23**, and **U31**. **U2** is one of the most cytotoxic compounds at 1 μM, contains lenalidomide, and features a triazole within a 9-atom linker, but it does not induce NAMPT degradation. **U16** is also highly cytotoxic at 1 μM, contains a VHL ligand and a triazole within the linker, and does not induce degradation. **U14** is cytotoxic at 10 μM, contains the VHL ligand and a triazole within a 9-atom linker, does not degrade NAMPT, and serves as the structural negative control of **U31**/**U42**. In contrast, **U23**, **U31**, and **U42** have linkers of similar length to the previous compounds but lack a triazole and are able to recruit VHL. All these compounds showed nanomolar inhibitory potency on recombinant NAMPT (Figures 5F and S2), demonstrating that, on one hand, the cytotoxic but nondegrading compounds (**U2**, **U16**, **U14**) induce cell death through NAMPT inhibition and, on the other, that our best PROTACs effectively inhibit NAMPT.

NAMPT has a twin enzyme, the nicotinic acid phosphoribosyltransferase (NAPRT), another critical enzyme in NAD salvage pathway which is structurally and functionally related to NAMPT. Despite the fact that it has previously been demonstrated that FK866 does not inhibit NAPRT (likely due to steric clashes between the inhibitor and the enzyme tunnel region),^32^ we verified whether **U42** could exert off-target effects on NAPRT. Therefore, we first evaluated the inhibitory activity of compounds **7** and **U42** on recombinant NAPRT using an enzymatic coupled assay. As shown by Figure S3A neither compound exhibited inhibitory activity against NAPRT, even at high concentrations. Afterward, to assess whether this selectivity was maintained in cellular models, 4T1 and MCF7 cells were treated with **U42** and NAMPT and NAPRT protein levels were monitored via Western blot. Figure S3B shows that **U42** induced NAMPT degrades without affecting NAPRT levels in either cell line.

Translating NAMPT inhibition into the cellular context, we measured the NAD^+^ levels in both MCF7 and 4T1 cells after 24 and 48 h of 300 nM **U42** treatment, which were significantly reduced (Figure 5G).

Once ascertained the direct involvement of NAMPT inhibition in **U42** activity, we explored the VHL recruitment. To validate the role of VHL in mediating the binding to the E3 ligase, we generated a null version of **U42**. Since the stereochemistry of the hydroxyl group on proline is crucial for VHL binding,^33^ the negative control **U42-NC** was synthesized using VHL ligand **104**, a (*S*)-hydroxyproline diastereoisomer of VHL ligand **103**, to prevent VHL E3 ligase recruitment (Scheme 6). As shown in Figure 6A, **U42-NC** failed to degrade NAMPT in both cell lines, as expected.

**Figure 6:**
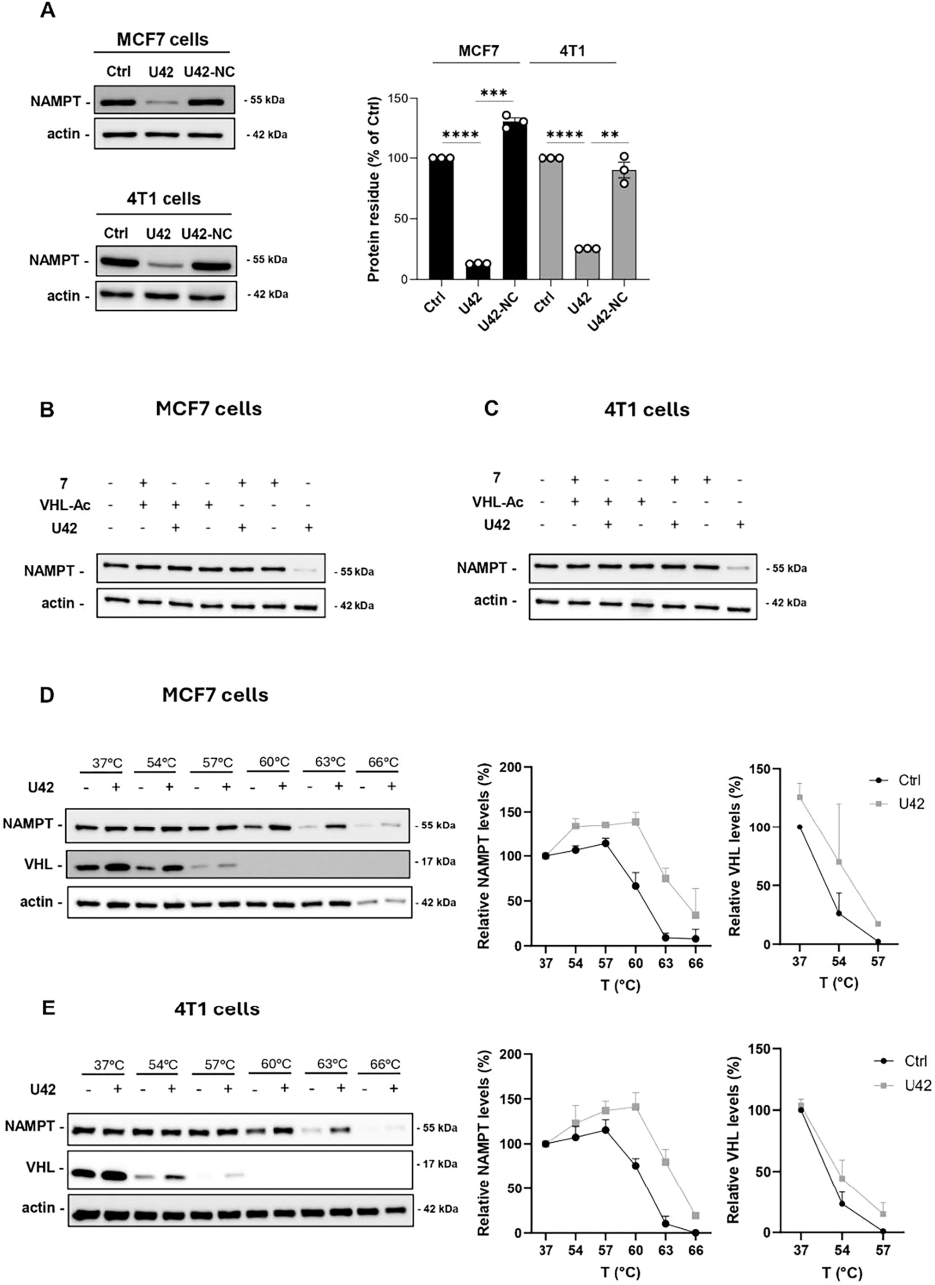
(**A**) Representative image and quantification of NAMPT degradation in both MCF7 (up) and 4T1 cells (down) treated for 18 h respectively with **U42** (100 nM) and **U42-NC** (100 nM). Mean ± SEM of 3 independent experiments. (**B, C**) NAMPT-degrading competition assay in MCF7 and 4T1 cells, respectively, treated with NAMPT inhibitor **7** (1 μM) or ligand **VHL-Ac** (100 μM), followed by **U42** (300 nM) or DMSO treatment for 18 h. Representative images are the results of 2 independent experiments. (**D, E**) NAMPT and VHL levels in MCF7 and 4T1 cells, respectively, treated with **U42** (10 μM) for 5 h by cellular thermal shift assay. Representative images are the results of 2 independent experiments.

Additionally, we synthesized the ligand **VHL-Ac** (VHL ligand **103**-Acetylated)^18^ to test it in a NAMPT-degradation competition assay. By treating cells with compound **7**, **VHL-Ac**, and **U42**, either alone or in combination, we demonstrated that only **U42** alone was capable to inducing NAMPT degradation, consistent with the formation of the ternary complex. On the other hand, when either the POI or the E3 ligase are saturated with an excess of ligand or inhibitor, **U42** could no longer form the ternary complex, and no degradation was observed (Figure 6B,C).

To further support our findings, we performed a cellular thermal shift assay (CETSA) to assess whether **U42** penetrates the cell membrane and effectively binds to both NAMPT and VHL. As shown in Figure 6D (MCF7 cells) and Figure 6E (4T1 cells), NAMPT exhibited increased thermal stability in **U42**-treated cells compared to the control, particularly at temperatures above 60 °C. Similarly, VHL showed enhanced stability upon **U42** treatment at temperatures above 54 °C. Collectively, these results indicate that **U42** is able to form a ternary complex by simultaneously engaging NAMPT and VHL, thereby promoting target degradation via UPS. On this bases, we asked whether the presence of the triazole group in the linker of **U14** effectively confers conformational rigidity by impeding binding to VHL. To verify this, we performed CETSA on 4T1 cells treated with **U14** in parallel with **U31**, its twin molecule (Figure S4A,B). **U31**, like **U42**, stabilized both NAMPT and VHL levels. Instead, **U14** only increased NAMPT levels stability without affecting VHL, suggesting an inability of **U14** to stabilize the ternary complex.

### Impact of NAMPT Degradation on Its Extracellular Moonlighting Localization

As mentioned above, NAMPT, besides being a cytosolic enzyme, also functions as an extracellular cytokine (eNAMPT) that binds to a receptor on the plasma membrane to exert its effects. We therefore evaluated whether **U42** could also reduce eNAMPT levels in the medium of breast cancer cells. Surprisingly, at the same time point as iNAMPT degradation (18 h in serum-free conditions), we did not appreciate any reduction in the extracellular form, but, on the contrary, we observed a trend of increasing eNAMPT levels under **U42** treatment (Figure S5A). In a previous study, Liu et al. developed FK866-based degraders aimed at selectively disrupting the nonenzymatic functions of NAMPT. They treated ovarian and colon cancer cells for 24 h, followed by a washing step and an additional 24-h incubation in serum-free medium containing the compounds.^16^ Based on this protocol, we treated MCF7 and 4T1 cells with **U42** for 18 h, washed the cells, and continued treatment in serum-free medium for additional 24 h. Under these conditions, in addition to degradation of iNAMPT (Figure S5B), we observed a dose-dependent degradation of eNAMPT (Figure 7A,B), with a DC_50_ of 50 nM, suggesting that a longer treatment duration is required to achieve complete depletion of the extracellular form of NAMPT. A plausible explanation is the presence of intracellular pools of pre-eNAMPT that must be depleted before a measurable reduction in the extracellular fraction occurs. Conversely, the transient increase in eNAMPT observed after only 18 h of **U42** treatment (Figure S5A) may reflect a compensatory or stress-induced secretory response triggered by intracellular NAMPT depletion. In this context, the time-dependent profile of eNAMPT release would be determined by both the rate of intracellular degradation and the activation of stress-regulated export pathways. Therefore, the temporal regulation of eNAMPT secretion is likely cell type–dependent and influenced by the cellular stress state induced by iNAMPT degradation.

**Figure 7:**
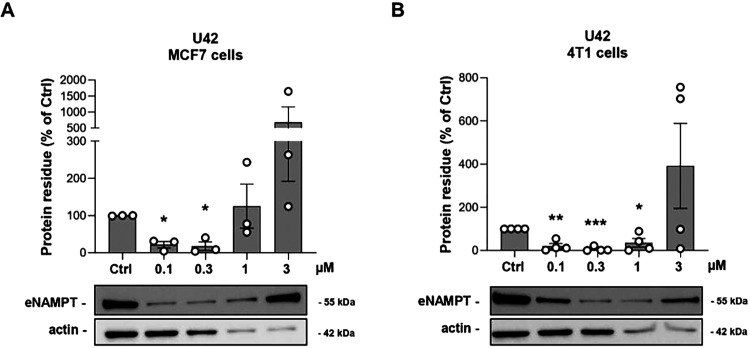
Representative image and quantifications of eNAMPT degradation in both MCF7 (**A**) and 4T1 cells (**B**) treated for 18 h in complete medium and then treated again for 24 h in serum-free conditions with a dose curve of **U42**. Mean ± SEM of 4 independent experiments. * *p* < 0.05, ** *p* < 0.01, *** *p* < 0.001 by unpaired parametric *t* test.

### Metabolic Stability and Pharmacokinetic Profile of NAMPT Degraders

The metabolic stability of **U31** and **U42** were assessed *in vitro* and both compounds demonstrated excellent resistance toward plasma and liver biotransformation with residual substrate greater than 90% after 60 min in the investigated settings, ruling out any noteworthy hydrolytic or oxidative degradation (Table 3).

**Table 3:** *In Vitro* Metabolic Stability Data of **U31** and **U42** in Mouse and Human and *In Vivo* PK Parameters of **U42** Administered in Mice

***In vitro*** **metabolic stability data** ^a^	**U31**	**U42**
% residue after 60 min incubation		
Phosphate buffer pH = 7.4	>99%	>99%
Mouse plasma	92%	92%
Human plasma	94%	>99%
MLM	95%	>99%

**U42** ^b^			
MLM *t*_1/2_ (min)	52.5	HLM *t*_1/2_ (min)	55.7
MLM CL_*in vitro*_ (μL min^–1^ mg^–1^)	13.2	HLM CL_*in vitro*_ (μL min^–1^ mg^–1^)	12.4

***In vivo*** **PK data** ^c^ **U31 U42**		
*t*_1/2_ (h)	1.6	1.6
*T*_max_ (h)	1.0	0.5
*C*_max_ (μg L^–1^)	217.0	178.9
AUC _0‑t_ (μg L^–1^ h^–1^)	382.4	412.1
CL/F (L h^–1^ kg^1–^)	52.3	48.5

a Incubated at the concentration of 50 μM.

b Incubated at the concentration of 5 μM.

c (*n* = 2 mice for each treatment) at a dose of 20 mg/kg (i.p.).

When incubated in liver microsomes, compound **U42** showed half-life ranging from 52.5 and 55.7 min in mouse liver microsomes (MLM) and human liver microsomes (HLM), respectively.

Based on these results, an explorative *in vivo* pharmacokinetic study was performed in mice to assess the PK parameters, following intraperitoneal administration at a dose of 20 mg/kg. The two compounds showed comparable profiles (Table 3 and Figures S6–S7), which is not surprising given their structural similarity.

Although **U42** was found to be stable in microsomes, its metabolic biotransformation was further investigated by high-resolution tandem mass spectrometry analysis, with data processed through Compound Discoverer 3.3. Molecular formulas of the detected metabolites were proposed based on accurate mass measurements of their protonated, biprotonated, and sodium adduct ions (Table S1), as well as their isotopic patterns. Metabolite structures were assigned by interpreting the fragment ions observed in the positive-mode MS^2^ spectra, as detailed in Section S112 in Supporting Information. In both mouse and human liver microsomes, **U42** underwent hepatic metabolism primarily through aliphatic hydroxylation (M1-M3) and desaturation (M4) reactions occurring at the hydrocarbon chain, as well pyridine *N*-oxidation (M5) on the MV78 moiety. Additional oxidative transformations included *O*-dealkylation (M6–M7) and *N*-dealkylation (M8–M10). Notably, no metabolic modifications were observed on the triazole group and VHL ligand portion and no amide hydrolysis products were detected (Figure 8), in agreement with the known robustness of hydroxyproline-based VHL ligands. The observed metabolic profile of **U42** aligns with common trends reported for PROTACs, with biotransformation events primarily occurring on the linker and POI ligand regions.

**Figure 8:**
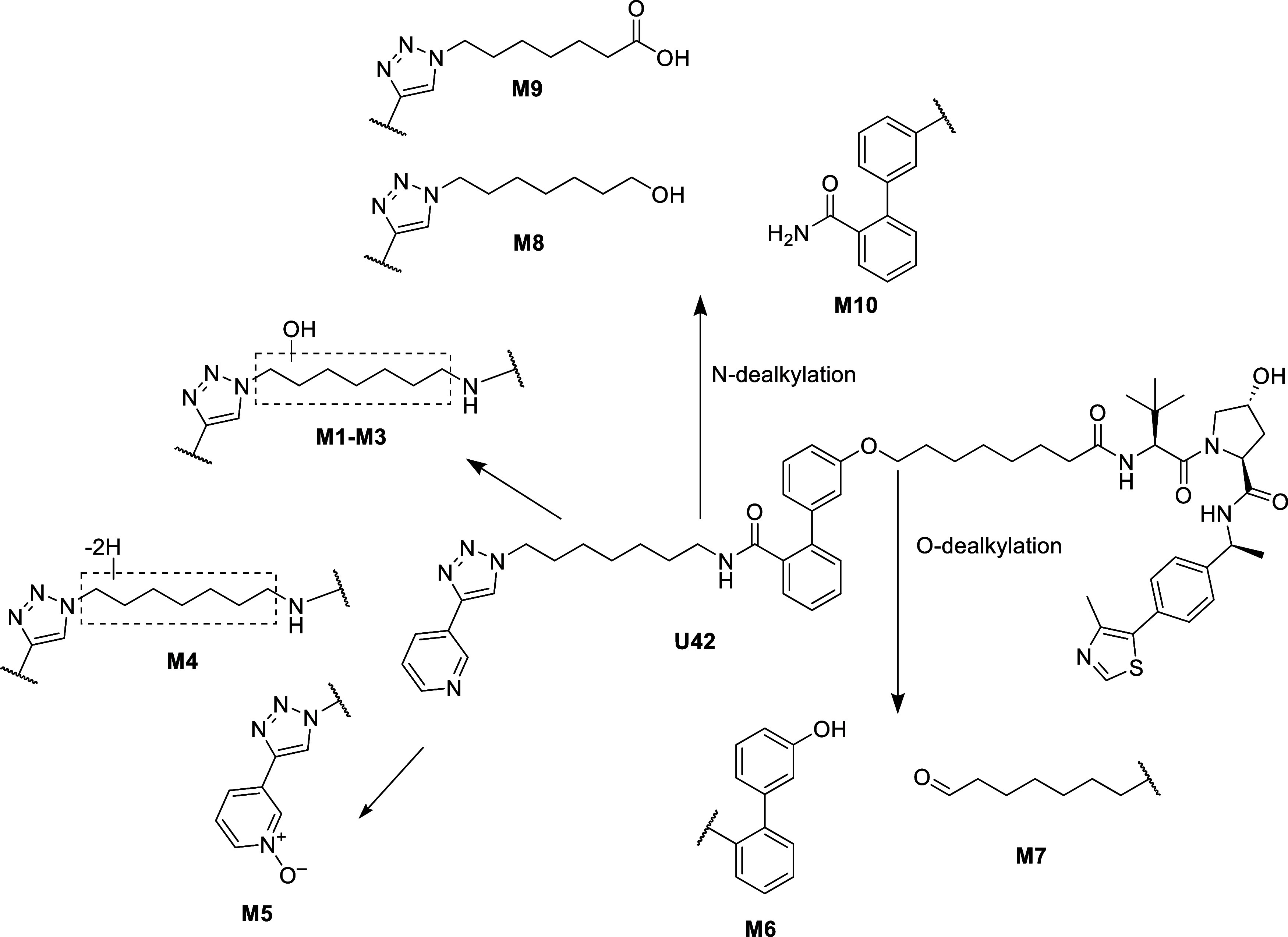
Proposed phase I metabolite profiling of compound **U42** in mouse and human liver microsomes.

### Targeted NAMPT Degradation Impairs Breast Cancer Mammosphere Viability

Finally, we evaluated the efficacy of **U42** in reducing tumor progression using a model that closely recapitulates three-dimensional tumor architecture *in vivo*. Both MCF7 and 4T1 cells were plated on polyHEMA-precoated plates and treated with a dose curve of **U42** for 8 days. Cells have been treated with **U42** at the time of plating and no additional treatment was performed during the 8 days in culture. This three-dimensional assay enables the evaluation of a PROTAC ability to inhibit the clonogenic potential of cancer cells. As shown in Figure 9A,B, treatment with **U42** at 3 μM resulted in a reduced number and area of mammospheres in both MCF7 and 4T1 cells. Additionally, **U42** effectively degraded NAMPT under mammosphere culture conditions following a single administration on day 1, further supporting the notion that NAMPT degradation impairs the tumorigenic potential of breast cancer cells (Figure 9C,D). Notably, eNAMPT levels measured in mammosphere culture medium after 8 days showed a significant decrease in 4T1 cells and a trend of decrease in MCF7 cells (Figure 9E,F). Data were obtained by calculating the ratio between eNAMPT levels from ELISA assay and the number of mammospheres in each independent experiment. These data mirror the efficiency of **U42** in degrading iNAMPT and, consequently, reducing eNAMPT levels, thereby contributing to its antitumor effect.

**Figure 9:**
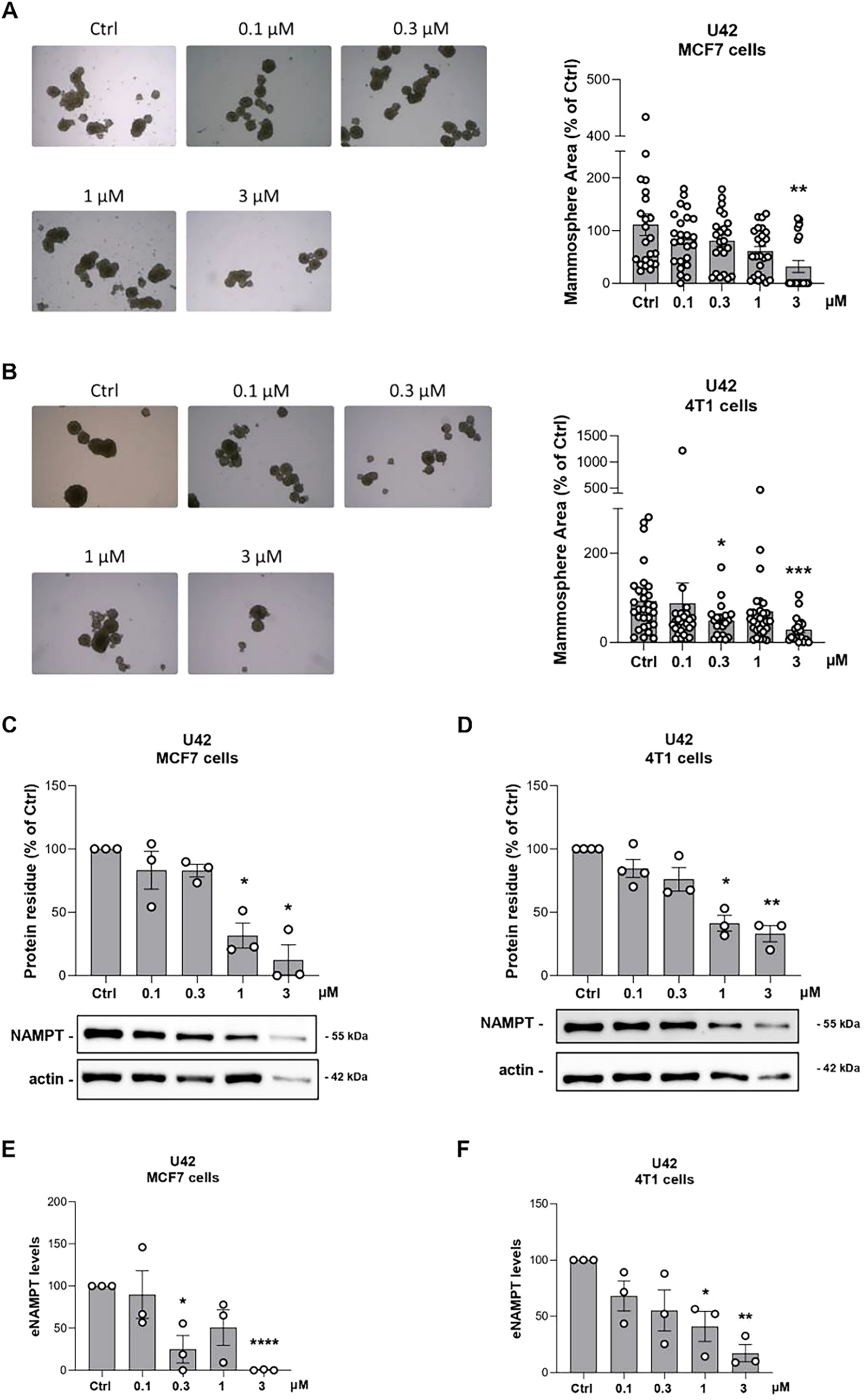
Image representation and quantification of mammosphere area in MCF7 (**A**) and 4T1 (**B**) cells on polyHEMA-precoated plates treated with a dose curve of **U42** for 8 days (one single treatment at the time of plating). Mean ± SEM of 5 independent experiments. * *p* < 0.05, ** *p* < 0.01 by unpaired parametric *t* test. Image representation and densitometry quantification of NAMPT degradation by Western blot analysis in MCF7 (**C**) and 4T1 (**D**) mammospheres. Mean ± SEM of 3 independent experiments. *p < 0.05, ** *p* < 0.01 by unpaired parametric *t* test. eNAMPT levels measured by ELISA assay in mammosphere culture medium after 8 days in MCF7 (**E**) and 4T1 (**F**) cells plated on polyHEMA-precoated plates and treated with a dose curve of **U42**. Mean ± SEM of 3 independent experiments. * *p* < 0.05, ** *p* < 0.01 by unpaired parametric *t* test.

### Mechanistic Interpretation of the Conformational Rigidity Imparted by Triazole Linkers

The SAR data highlighted a sharp dichotomy between triazole-free and triazole-containing linkers: while **U31** (and its optimized analogue **U42**) supported robust NAMPT degradation, closely related CuAAC-derived analogues failed to induce degradation despite retaining NAMPT inhibitory activity.

To rationalize this behavior at the structural level, we selected **U31** as the active reference compound and **U14** as its matched negative control, since the two PROTACs share the same overall architecture and comparable NAMPT inhibition but differ by the insertion of a triazole fragment within the linker, which correlates with loss of degradation (Figure 10).

**Figure 10:**
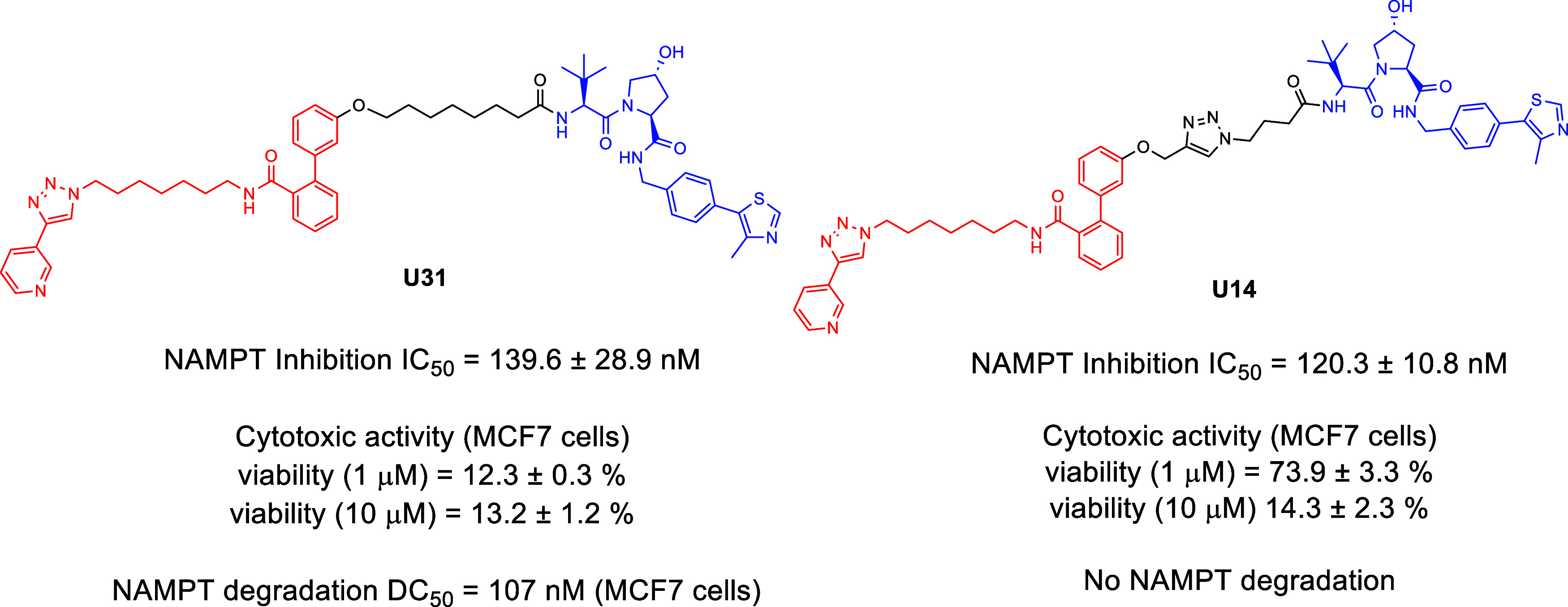
Chemical structure and biological activity of **U31** and **U14**.

We therefore performed a molecular modeling workflow aimed at (i) generating plausible NAMPT–PROTAC–VHL ternary complexes for both compounds and (ii) assessing, through molecular dynamics (MD) simulations, whether the triazole substitution alters linker flexibility and compromises the conformational adjustments required for stable ternary complex formation.

Initially, the docking poses of MV78 (**7**) were reproduced according to the previously reported protocol using FRED software.^25^ The obtained docking results confirmed the correct placement of the pyridine ring within the NAMPT active site, stabilized through π–π stacking interactions with Phe193 and Tyr18′, while the biphenyl moiety occupied the same solvent-exposed cavity described for previously studied analogs.

Based on these results, the PROTAC model was built by linking the **7** headgroup to the hydroxyproline-containing ligand (**10**) for VHL derived from the crystallographic structure (PDB ID: 7JTO). The coordinates of the VHL tail were preserved as in the experimental complex, maintaining its binding mode in the recognition pocket. The ternary complex (Figure 11) was generated using the PROTAC-Model platform reported by the authors of PROTAC-DB,^34^ the same procedure was used to generate the ternary complex with **U14**.

**Figure 11:**
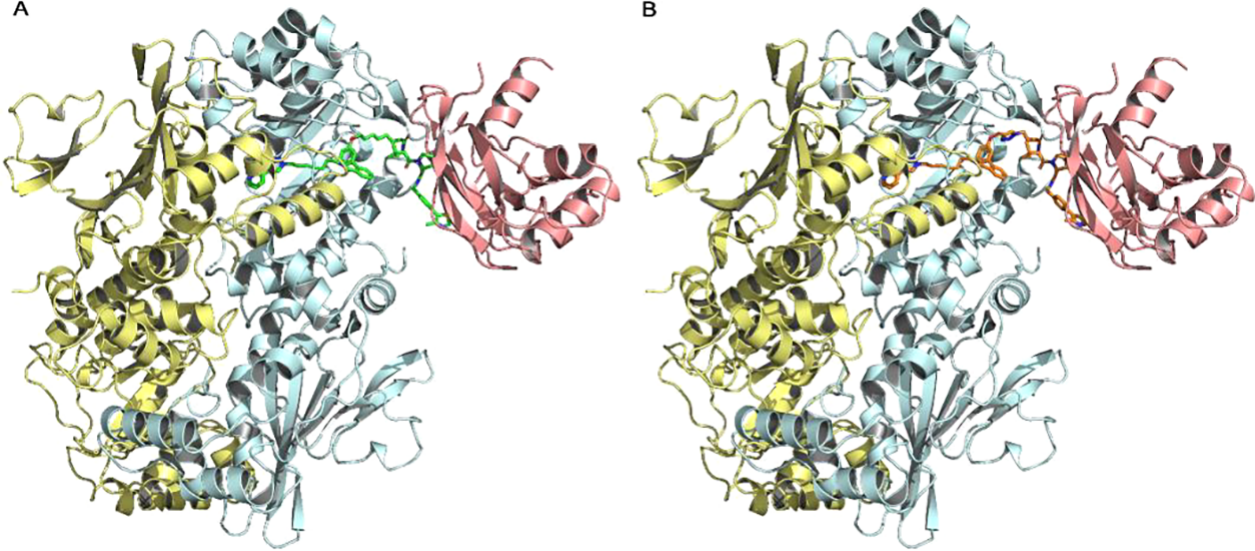
Complex structure of hNAMPT-VHL-ligands. The backbone of hNAMPT is shown as cartoon representation (yellow and cyan), the backbone of VHL is shown as cartoon (salmon). PROTAC compounds **U31** (A), and **U14** (B) are shown as sticks, green and orange, respectively.

To explore the dynamic behavior and stability of the ternary complexes, MD simulations were performed for both **U31**- and **U14**-containing assemblies, using the software Desmond. At the beginning of the simulations, the two systems exhibited very similar overall conformations, as expected due to their close structural resemblance. In both cases, the PROTAC molecules share a common architecture, differing mainly by the replacement of a linker fragment with a triazole in **U14**. This minor structural modification resulted in nearly identical binding arrangements in the initial docked complexes (Figure S8).

However, the MD trajectories revealed that the subsequent conformational evolution of the two systems diverged significantly over time. The **U31** complex achieved a stable conformation shortly after equilibration and maintained it consistently throughout the 100 ns simulation. The RMSD profiles of both the protein backbone and the ligand remained low and stable (Figures S9–S10), indicating limited structural drift during the trajectory. Analysis of protein–ligand interaction maps confirmed the persistence of key hydrogen bonds and π–π stacking interactions within the NAMPT binding site, as well as stable contacts between the VHL pocket and the PROTAC tail (Figure S11). The overall ternary assembly remained compact and well-ordered, supporting the ability of **U31** to maintain an effective interface between the two proteins.

In contrast, the **U14**-containing complex exhibited a progressive loss of structural integrity starting around the midpoint of the simulation (∼50 ns). This trend was evident from the increase in RMSD values (Figures S12–S13) and from contact map analysis, which showed a marked decrease in persistent interactions between the NAMPT pocket and the headgroup derived from MV78 (**7**) (Figure S14). Representative trajectory snapshots (Figure 12) illustrate the gradual disengagement of the NAMPT-binding moiety, leading to partial disruption of the ternary architecture.

**Figure 12:**
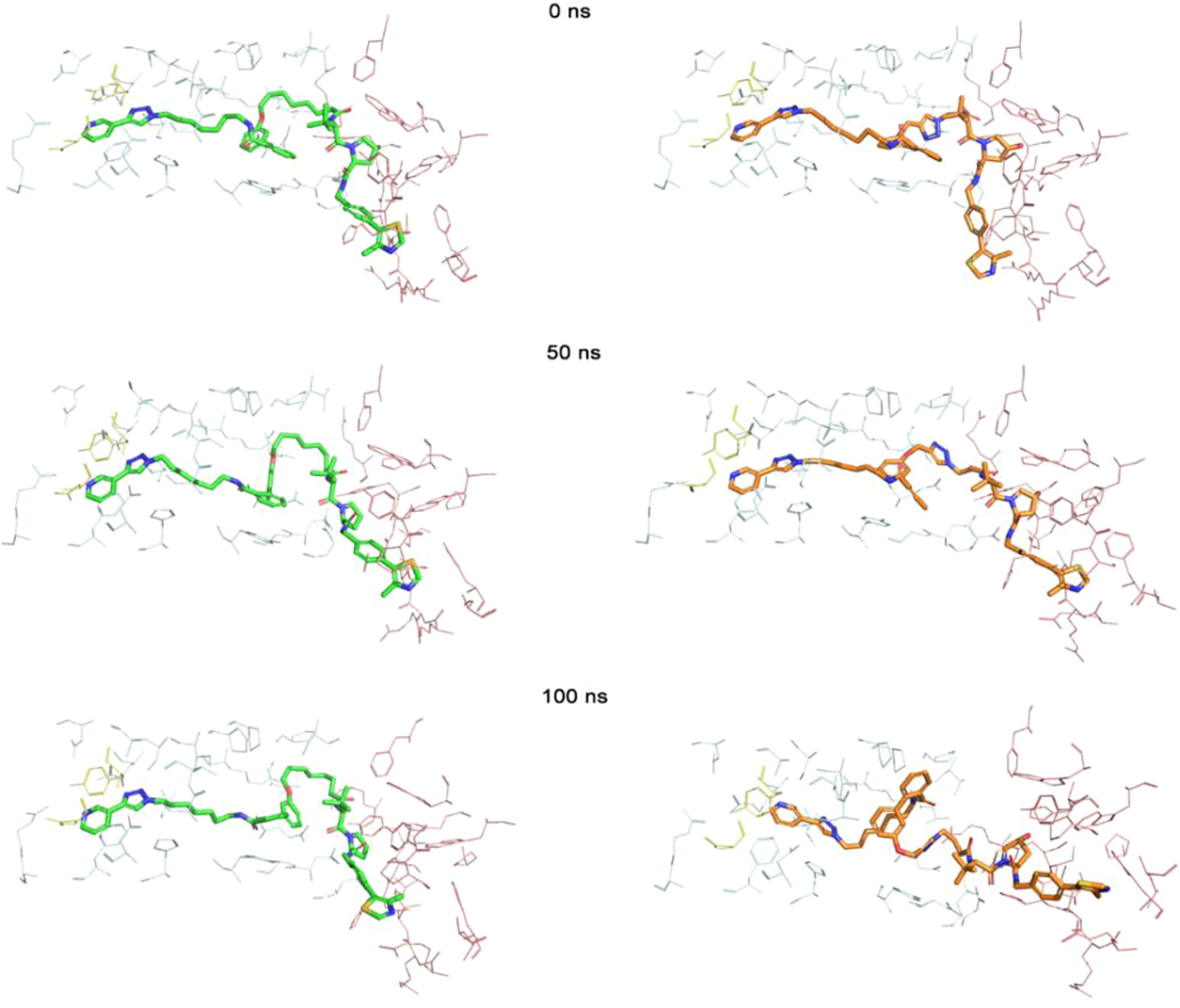
Representative trajectory snapshots of the ternary complex with **U31** (left) and **U14** (right).

All MD simulations were carried out in triplicate using different random seeds, consistently reproducing these trends and confirming the reliability of the observed structural divergence between the two complexes.

From a mechanistic perspective, the different behaviors of **U31** and **U14** likely arise from the impact of the triazole substitution within the linker region. In **U14**, the presence of the triazole ring reduces local flexibility and may alter the torsional dynamics required to maintain an optimal spatial arrangement between the two binding domains. This constrained geometry could hinder the fine conformational adjustments necessary to accommodate both proteins simultaneously, thereby weakening the cooperative interactions that stabilize the ternary complex. Conversely, the more flexible linker of **U31** appears to allow better alignment of the binding motifs, promoting sustained protein–protein association and overall complex stability.

## Conclusions

In summary, leveraging on a potent NAMPT inhibitor previously developed by our group, we designed, synthesized, and comprehensively evaluated a novel class of NAMPT-targeting PROTACs. A systematic SAR study was conducted, varying both the E3 ligase recruiter and linker structure. Unlike prior reports of NAMPT degraders,^16,18^ our CuAAC-derived PROTACs featuring a triazole moiety in the linker failed to induce degradation, highlighting the crucial role of linker design. Additionally, none of the lenalidomide-based compounds, regardless of triazole inclusion, showed degradation capability. Consistently, molecular modeling and MD simulations provided a mechanistic rationale for this SAR, suggesting that triazole insertion rigidifies the linker and destabilizes the NAMPT–PROTAC–VHL ternary complex, thereby impairing productive degradation. Notably, triazole removal restored degradation capability and led to the identification of **U31**, a VHL-recruiting PROTAC with nanomolar potency in MCF7 and 4T1 breast cancer cell lines. The introduction of an (*S*)-methyl group on the VHL ligand (the “magic methyl”) yielded **U42**, a compound with enhanced degradation potency (DC_50_ = 45 nM in MCF7, 55 nM in 4T1), and low nanomolar antiproliferative activity. The cytotoxic activity of **U42** was primarily attributed to its ability to bind NAMPT, inhibit its function, and promote its proteasomal degradation, leading to a reduction in NAD^+^ levels in both breast cancer cell lines. Mechanistic studies employing proteasome inhibition (bortezomib) and an inactive diastereomeric control (**U42-NC**) confirmed that the degradation was VHL-dependent. Moreover, we validated the formation of a ternary complex among the PROTAC, NAMPT, and VHL through NAMPT-degradation competition and CETSA assays. **U42** effectively promoted degradation of both intracellular and extracellular NAMPT, although full eNAMPT depletion required prolonged treatment, as shown using a modified assay protocol. Importantly, **U42** retained its activity in three-dimensional mammosphere cultures, reducing both iNAMPT and eNAMPT levels and impairing clonogenic growth—suggesting a potential to diminish tumorigenic capacity. Pharmacokinetic studies in mice demonstrated favorable *in vivo* properties for **U42**, supporting its progression into preclinical development.

Most published degraders employ NAMPT inhibitors such as FK866 or related analogues, which contain functional groups including acrylamides, thioureas, or ureas. These moieties have been associated with potential liabilities: the acrylamide of FK866 is electrophilic; thioureas have been reported as teratogenic,^18,35^ and ureas can inhibit CYP450 isoforms^36^ and have also been suggested to carry teratogenic potential. In contrast, our degraders employ MV78, a triazolylpyridine derivative as the warhead. The triazole unit is generally considered chemically and metabolically stable and shows minimal CYP450 inhibition except at very high concentrations.^37^ Metabolic profiling of our most active PROTAC confirmed a favorable stability profile, which may be partly attributed to the intrinsic robustness of the triazole in MV78. Taken together, these comparisons suggest potential advantages of MV78-based NAMPT PROTACs over previously reported scaffolds and provide a rationale for their further exploration.

The recent phase III success of vepdegestrant—an estrogen receptor (ER)–targeting PROTAC that improved progression-free survival in ER^+^/HER2^–^ breast cancer patients harboring ESR1 mutations—highlights the clinical promise of PROTAC-based therapies in oncology, despite their current applicability being limited to specific patient subgroups.^38^ In this context, the present study identifies **U42** as a promising NAMPT degrader with potent cellular activity and encouraging pharmacokinetics. These results provide a strong rationale for further development of NAMPT-targeting PROTACs as a therapeutic approach in breast cancer and potentially beyond. Given that systemic NAMPT inhibition is known to cause adverse effects, our future efforts will focus on tumor-selective delivery strategies.^6^ Specifically, we aim to develop activatable PROTACs designed to restrict NAMPT degradation to the breast cancer microenvironment.

## Experimental Section

### Chemistry

#### General Methods

All reagents and solvents were purchased from commercial sources and used without further purification. As needed, the reactions were performed in oven-dried glassware under a positive pressure of dry nitrogen. Melting points were determined in an open glass capillary with a Stuart scientific SMP3 apparatus and were uncorrected. Infrared spectra were acquired with a FT-IR Thermo-Nicolet Avatar or Bruker Alpha II spectrometer. ^1^H NMR and ^13^C NMR spectra were recorded on a JEOL ECP 300 MHz spectrometer or Bruker Avance Neo 400 MHz spectrometer. Chemical shifts (δ) are reported in parts per million (ppm) referenced to the residual solvent peak. The multiplicity of each signal is designated using the following abbreviations: s (singlet), d (doublet), t (triplet), q (quadruplet), quint (quintuplet), m (multiplet), br s (broad singlet), dd (doublet of doublets). Coupling constants (*J*) are reported in Hertz (Hz). High-Resolution ESI-MS spectra were acquired on a Thermo Scientific Q-Exactive Plus Hybrid Quadrupole-Orbitrap mass spectrometer. The spectra were recorded by infusion into the ESI source using methanol as the solvent. Flash column chromatography was performed on silica gel (Merck Kieselgel 60, 230–400 mesh ASTM). Thin layer chromatography (TLC) was carried out on plates with a layer thickness of 0.25 mm (Merck Silica gel 60 F_254_); when necessary, they were developed with KMnO_4_ reagent.

Compounds MV78 (**7**),^25^ VHL ligand **10**,^33^ VHL ligand **103**,^39^ and ligand **VHL-Ac** (VHL ligand **103**-Acetylated)^18^ were synthesized according to the literature.

Compounds **9** (lenalidomide), **18–30** (alkyne-alcohols), **63–64** (*tert-*butyl ester functionalized polyethylene glycols), **79–84** (bromo-carboxylic acids) and **104** (the *S*-hydroxyproline diastereoisomer of VHL ligand **103**) were commercially available.

Synthetic methods for the preparation of amine **16**, linkers **31–43**, **67–70**, **85–90** and ^1^H NMR, ^13^C NMR data of the intermediates were reported in Section S14 in Supporting Information.

The purity of final compounds was ≥95% and was determined by high performance liquid chromatography coupled with ultraviolet–visible detector (LC-UV) using the instrumentation and methods reported in Section S87 in Supporting Information.

### Preparation of NAMPT Ligand 3′-Hydroxy-*N*-(7-(4-(pyridin-3-yl)-1*H*-1,2,3-triazol-1-yl)­heptyl)-[1,1′-biphenyl]-2-carboxamide (**8**)

2-Iodobenzoic acid (0.374 g, 1.51 mmol, 1.1 equiv), EDCI (0.340 g, 1.78 mmol, 1.3 equiv), TEA (0.762 mL, 5.48 mmol, 4 equiv) and DMAP (0.0167 g, 0.137 mmol, 0.1 equiv) were added to a solution of amine **16** (0.355 g, 1.37 mmol, 1 equiv) in dry CH_2_Cl_2_ (5 mL) and the reaction mixture was stirred overnight at room temperature under nitrogen. Upon completion, the reaction was diluted with water and extracted with CH_2_Cl_2_ (×3). The combined organic layers were washed with a 2 M NaOH aqueous solution (×1), brine (×1), dried over anhydrous Na_2_SO_4_ and the solvent was evaporated under vacuum. The crude was purified by column chromatography using CH_2_Cl_2_/MeOH 98:2 as eluent. The product was triturated at 0 °C with EtOAc and subsequently filtered under vacuum. 2-Iodo-*N*-(7-(4-(pyridin-3-yl)-1*H*-1,2,3-triazol-1-yl)­heptyl)­benzamide (**17**) was collected as a white solid, 83% yield. IR (KBr) 3331, 3120, 2929, 2852, 1631, 1533, 1289, 806, 709 cm^–1^; ^1^H NMR (300 MHz, CDCl_3_) δ 8.99 (s, 1H), 8.56 (d, *J* = 4.9 Hz, 1H), 8.20 (d, *J* = 8.0 Hz, 1H), 7.86–7.83 (m, 2H), 7.37–7.33 (m, 3H), 7.11–7.05 (m, 1H), 5.79 (br s, 1H), 4.43 (t, *J* = 7.0 Hz, 2H), 3.45 (q, *J* = 6.7 Hz, 2H), 1.98 (quint, *J* = 7.0 Hz, 2H), 1.66–1.61 (m, 4H), 1.42 (br s, 4H) ppm; ^13^C NMR (75 MHz, CDCl_3_) δ 169.5, 148.9, 146.8, 144.4, 142.5, 139.5, 132.9, 130.7, 128.0 (2C), 126.8, 123.7, 120.2, 92.5, 50.4, 39.7, 30.1, 29.1, 28.4, 26.6, 26.2 ppm.

Amide **17** (1,14 g, 2.35 mmol, 1 equiv) was dissolved in 1,2-dimethoxyethane (14 mL), MeOH (8.6 mL) and H_2_O (2 mL) in a Schlenk tube. Then (3-hydroxyphenyl)­boronic acid (0.454 g, 3.28 mmol, 1.4 equiv), palladium tetrakis (0.407 g, 0.352 mmol, 0.15 equiv) and K_2_CO_3_ (1.29 g, 9.39 mmol, 4 equiv) were added. Subsequently the solution was degassed by bubbling a stream of nitrogen through the solvent for 10 min. The reaction was stirred at 90 °C under nitrogen atmosphere overnight. Upon completion, the reaction was filtered under vacuum and rinsed multiple times with MeOH. The filtrate was evaporated, the solid residue was suspended in water and extracted with EtOAc (×2). The combined organic layers were then washed with brine (×1), dried over anhydrous Na_2_SO_4_, filtered and the solvent was evaporated under vacuum. The crude was purified by column chromatography using EtOAc and EtOAc/MeOH 95:5 as eluents to afford MV78-OH (**8**) as a white solid (0.901 g, 84% yield). ^1^H NMR (400 MHz, CD_3_OD) δ 9.02 (br s, 1H), 8.51 (d, *J* = 4.2 Hz, 1H), 8.48 (s, 1H), 8.27 (dt, *J* = 8.0/1.8 Hz, 1H), 7.81 (t, *J* = 5.6 Hz, 1H), 7.52–7.35 (m, 5H), 7.17 (t, *J* = 8.0 Hz, 1H), 6.88–6.86 (m, 2H), 6.78–6.75 (m, 1H), 4.46 (t, *J* = 7.1 Hz, 2H), 3.14 (t, *J* = 6.8 Hz, 2H), 1.94 (t, *J* = 6.9 Hz, 2H), 1.33–1.28 (m, 6H), 1.07–1.03 (m, 2H) ppm; ^13^C NMR (101 MHz, CD_3_OD) δ 171.5, 157.1, 148.1, 145.8, 143.9, 141.7, 139.8, 136.4, 133.5, 129.7, 129.4, 129.0, 127.4, 127.3, 126.9, 124.2, 121.7, 119.6, 115.3, 114.1, 50.2, 39.3, 29.8, 28.4, 28.3, 26.2, 26.0 ppm; HRMS (ESI) *m*/*z* calcd for C_27_H_30_N_5_O_2_ [M + H]^+^ 456.23940, found 456.23889.

### Synthesis of NAMPT Targeting PROTACs **U1–13**

#### General Procedure A for the Williamson Etherification (**44–56**)

Under a nitrogen atmosphere MV78-OH (**8**) (1 equiv) was dissolved in dry acetonitrile (0.06 M) and K_2_CO_3_ (4 equiv) was added. The resulting suspension was heated to 85 °C and vigorously stirred for 15 min. Subsequently, the corresponding tosylated alkyne (**31–43**) (2 equiv) was added dropwise and the reaction mixture was heated to 85 °C for 5 h. The suspension was filtered and the solid was washed with CH_2_Cl_2_. The filtrate obtained was concentrated in vacuo and the crude was purified by column chromatography.

### Synthesis of 2-Azido-*N*-(2-(2,6-dioxopiperidin-3-yl)-1-oxoisoindolin-4-yl)­acetamide (**58**)

2-Bromoacetyl chloride (1.22 g, 7.72 mmol, 2 equiv) was added to a solution of commercial lenalidomide (**9**) (1.00 g, 3.86 mmol, 1 equiv) in THF (12 mL) and the reaction was heated to reflux for 2 h under nitrogen atmosphere. Upon completion, the solvent was evaporated under vacuum, the solid residue was suspended in EtOAc at 0 °C and then filtered under vacuum. 2-Bromo-*N*-(2-(2,6-dioxopiperidin-3-yl)-1-oxoisoindolin-4-yl)­acetamide (**57**) was collected as a white solid (1.37 g, 94% yield); IR (neat) 3302, 3237, 3167, 3083, 2968, 2839, 1695, 1669, 1546, 1300, 1193, 750 cm^–1^; ^1^H NMR (400 MHz, (CD_3_)_2_SO) δ 11.03 (s, 1H), 10.28 (s, 1H), 7.84 (d, *J* = 7.5 Hz, 1H), 7.56–7.52 (m, 2H), 5.15 (dd, *J* = 13.2/5.0 Hz, 1H), 4.41 (d, *J* = 17.5 Hz, 1H, AB system), 4.34 (d, *J* = 17.5 Hz, 1H, AB system), 4.11 (s, 2H), 2.92 (ddd, *J* = 17.6/13.3/4.8 Hz, 1H), 2.64–2.60 (m, 1H), 2.35 (qd, *J* = 13.1/4.3 Hz, 1H), 2.10–2.03 (m, 1H) ppm; ^13^C NMR (101 MHz, (CD_3_)_2_SO) δ 173.4, 171.5, 168.2, 165.6, 134.3, 133.5, 133.3, 129.4, 125.7, 120.2, 52.0, 46.8, 31.7, 30.2, 23.1 ppm; HRMS (ESI) *m*/*z* calcd for C_15_H_15_BrN_3_O_4_ [M + H]^+^ 380.02459, found 380.02373.

NaN_3_ (0.45 g, 6.91 mmol, 2 equiv) was added to a solution of bromo-derivative **57** (1.30 g, 3.46 mmol, 1 equiv) in acetone (15 mL) and the reaction was heated to reflux overnight. Upon completion, the solvent was evaporated under vacuum. The solid residue was suspended in water (6 mL) and CH_2_Cl_2_ (12 mL) and then filtered under vacuum. Azide **58** was obtained as a white solid (1.04 g, 88% yield); IR (neat) 3301, 3079, 2905, 2838, 2107, 1690, 1656, 1544, 1334. 1282, 1178, 749 cm^–1^; ^1^H NMR (400 MHz, (CD_3_)_2_SO) δ 11.03 (br s, 1H), 10.23 (br s, 1H), 7.86 (dd, *J* = 7.3/1.2 Hz, 1H), 7.57–7.51 (m, 2H), 5.15 (dd, *J* = 13.3/5.1 Hz, 1H), 4.45 (d, *J* = 17.6 Hz, 1H, AB system), 4.37 (d, *J* = 17.6 Hz, 1H, AB system), 4.14 (s, 2H), 2.92 (ddd, *J* = 17.7/13.2/4.9 Hz, 1H), 2.64–2.60 (m, 1H), 2.38 (qd, *J* = 9.0/4.3 Hz, 1H), 2.06–2.02 (m, 1H) ppm; ^13^C NMR (101 MHz, (CD_3_)_2_SO) δ 173.4, 171.5, 168.2, 167.1, 134.3, 133.4, 133.2, 129.3, 125.9, 120.0, 52.6, 52.1, 47.0, 31.7, 23.1 ppm. HRMS (ESI) *m*/*z* calcd for C_15_H_15_N_6_O_4_ [M + H]^+^ 343.11548, found 343.11457.

#### General Procedure B for the Click Reaction

Azide **58** (1.2 equiv), CuSO_4_·5H_2_O (0.2 equiv) and sodium ascorbate (1 equiv) were added to a solution of the corresponding alkyne (**44–56**) (1 equiv) in a mixture of THF/H_2_O 1:1 (0.08 M) and the reaction was stirred overnight at 55 °C. Upon completion, the solvent was evaporated under vacuum and the reaction crude was purified by column chromatography.

##### 3′-(2-(1-(2-((2-(2,6-Dioxopiperidin-3-yl)-1-oxoisoindolin-4-yl)­amino)-2-oxoethyl)-1*H*-1,2,3-triazol-4-yl)­ethoxy)-*N*-(7-(4-(pyridin-3-yl)-1*H*-1,2,3-triazol-1-yl)­heptyl)-[1,1′-biphenyl]-2-carboxamide (**U1**)

The compound was prepared from **44**. The crude was purified using EtOAc/MeOH 9:1 as eluent to give a white solid; yield 44%; IR (neat): 3350, 2922, 2852, 1626, 1603, 1409, 1363, 1201, 1051, 752 cm^–1^; ^1^H NMR (400 MHz, CD_3_OD) δ 9.02 (br s, 1H), 8.52 (br s, 1H), 8.44 (s, 1H), 8.25 (d, *J* = 8.0 Hz, 1H), 7.97 (s, 1H), 7.76 (d, *J* = 7.9 Hz, 1H), 7.67 (d, *J* = 7.5 Hz, 1H), 7.54–7.38 (m, 6H), 7.26 (t, *J* = 7.8 Hz, 1H), 7.0–6.98 (m, 2H), 6.92–6.89 (m, 1H), 5.40 (s, 2H), 5.14 (dd, *J* = 13.2/5.2 Hz, 1H), 4.50 (s, 2H), 4.44 (t, *J* = 7.0 Hz, 2H), 4.30 (t, *J* = 6.4 Hz, 2H), 3.21 (t, *J* = 6.4 Hz, 2H), 3.11 (t, *J* = 3.1 Hz, 2H), 2.95–2.76 (m, 2H), 2.48–2.43 (m, 1H), 2.20–2.17 (m, 1H), 1.92 (quint, *J* = 6.9 Hz, 2H), 1.31–1.24 (m, 6H), 1.04 (quint, *J* = 7.4 Hz, 2H) ppm; ^13^C NMR (101 MHz, CD_3_OD) δ 173.2, 171.4, 170.6, 169.6, 164.8, 158.6, 148.1, 147.5, 145.9, 143.9, 141.8, 139.4, 136.6, 134.5, 133.4, 133.6 (2C), 129.7, 129.4, 129.1, 128.8, 127.3 (2C), 127.0, 126.0, 124.3, 123.7, 121.7, 121.1, 120.3; 114.9, 113.5, 66.6, 52.3, 51.8, 50.2, 47.2, 39.3, 30.9, 29.7, 28.4, 28.3, 26.2, 25.9, 25.5, 22.7 ppm; HRMS (ESI), *m*/*z* [M + H]^+^ calcd for C_46_H_48_N_11_O_6_ 850,37890, found 850,37789.

##### 3′-(3-(1-(2-((2-(2,6-Dioxopiperidin-3-yl)-1-oxoisoindolin-4-yl)­amino)-2-oxoethyl)-1*H*-1,2,3-triazol-4-yl)­propoxy)-*N*-(7-(4-(pyridin-3-yl)-1*H*-1,2,3-triazol-1-yl)­heptyl)-[1,1′-biphenyl]-2-carboxamide (**U2**)

The compound was prepared from **45**. The crude was purified using EtOAc/MeOH 9:1 as eluent to give a white solid; yield 69%; IR (neat): 3055, 2922, 2853, 1687, 1637, 1602, 1545, 1433, 12.87, 1201, 1050, 751, 698 cm^–1^; ^1^H NMR (400 MHz, CD_3_OD) δ 9.01 (br s, 1H), 8.49 (br d, 1H), 8.44 (s, 1H), 8.23 (dt, *J* = 8.4/1.3 Hz, 1H), 7.87 (s, 1H), 7.76 (dd, *J* = 8.0/0.8 Hz, 1H), 7.63 (d, *J* = 6.9 Hz, 1H), 7.50–7.43 (m, 4H), 7.39–7.35 (m, 2H), 7.23 (t, *J* = 8.2 Hz, 1H), 6.97–6.95 (m, 2H), 6.88–6.86 (m, 1H), 5.39 (s, 2H), 5.12 (dd, *J* = 13.2/5.2 Hz, 1H), 4.49 (s, 2H), 4.43 (t, *J* = 7.1 Hz, 2H), 4.04 (t, *J* = 6.2 Hz, 2H), 3.11 (t, *J* = 6.9 Hz, 2H), 2.87 (t, *J* = 6.3 Hz, 2H), 2.79–2.77 (m, 1H), 2.74–2.72 (m, 1H), 2.42 (qd, *J* = 13,1/4.8 Hz, 1H), 2.18–2.11 (m, 3H), 1.91 (quint, *J* = 7.0 Hz, 2H), 1.30–1.23 (m, 6H), 1.04 (quint, *J* = 7.5 Hz, 2H) ppm; ^13^C NMR (101 MHz, CD_3_OD) δ 173.1, 171.4, 170.6, 169.5, 164.9, 158.9, 148.2, 147.1, 146.0, 144.0, 141.8, 139.6, 136.6, 134.7, 133.5, 132.7, 132.5, 129.7, 129.4, 129.0, 128.8, 127.3 (2C), 127.0, 126.1, 124.1, 123.8, 121.7, 120.9, 120.4, 114.8, 113.6, 66.8, 52.4, 52.0, 50.2, 47.2, 39.3, 31.0, 29.7, 28.6, 28.4, 28.3, 26.2, 25.9, 22.7, 21.6 ppm; HRMS (ESI), *m*/*z* [M + H]^+^ calcd for C_47_H_50_N_11_O_6_ 864.39455, found 864.39332.

##### 3′-(4-(1-(2-((2-(2,6-Dioxopiperidin-3-yl)-1-oxoisoindolin-4-yl)­amino)-2-oxoethyl)-1*H*-1,2,3-triazol-4-yl)­butoxy)-*N*-(7-(4-(pyridin-3-yl)-1*H*-1,2,3-triazol-1-yl)­heptyl)-[1,1′-biphenyl]-2-carboxamide (**U3**)

The compound was prepared from **46**. The crude was purified using EtOAc/MeOH 9.5:0.5 as eluent to give a white solid; yield 45%; IR (neat): 3232, 3130, 3065, 2923, 2853, 1687, 1630, 1602, 1548, 1433, 1287, 1200, 1049, 751, 699 cm^–1^; ^1^H NMR (400 MHz, CD_3_OD) δ 9.02 (br s, 1H), 8.51 (br s, 1H), 8.48 (s, 1H), 8.26 (d, *J* = 8.0 Hz, 1H), 7.86 (s, 1H), 7.77 (d, *J* = 7.9 Hz, 1H), 7.64 (d, *J* = 7.4 Hz, 1H), 7.50–7.38 (m, 6H), 7.24 (t, *J* = 8.2 Hz, 1H), 6.97–6.95 (m, 2H), 6.84 (dd, *J* = 7.7/2.0 Hz, 1H), 5.38 (s, 2H), 5.15 (dd, *J* = 13.3/5.1 Hz, 1H), 4.47 (s, 2H), 4.43 (t, *J* = 7.0 Hz, 2H), 3.99 (t, *J* = 5.8 Hz, 2H), 3.12 (t, *J* = 6.8 Hz, 2H), 2.94–2.73 (m, 4H), 2.41 (qd, *J* = 13.2/4.6 Hz, 1H), 2.18–2.14 (m, 1H), 1.90–1.85 (m, 1H), 1.31–1.23 (m, 6H), 1.01–0.99 (m, 2H) ppm; ^13^C NMR (101 MHz, CD_3_OD) δ 173.2, 171.6, 170.7, 169.5, 164.9, 158.9, 148.1, 147.6, 145.8, 143.9, 141.7, 139.6, 136.5, 134.4, 133.4, 132.7, 132.6, 129.7, 129.4, 129.0, 128.8, 127.3 (2C), 127.0, 125.9, 124.3, 123.8, 121.7, 120.7, 120.3, 114,5, 113.4, 67.2, 52.2, 51.8, 50.2, 47.2, 39.3, 30.9, 29.8, 28.4 (2C), 26.2, 25.9, 25.7, 24.6, 22.7 ppm; HRMS (ESI), *m*/*z* [M + H]^+^ calcd for C_48_H_52_N_11_O_6_ 878.41020, found 878.40988.

##### 3′-((5-(1-(2-((2-(2,6-Dioxopiperidin-3-yl)-1-oxoisoindolin-4-yl)­amino)-2-oxoethyl)-1*H*-1,2,3-triazol-4-yl)­pentyl)­oxy)-*N*-(7-(4-(pyridin-3-yl)-1*H*-1,2,3-triazol-1-yl)­heptyl)-[1,1′-biphenyl]-2-carboxamide (**U4**)

The compound was prepared from **47**. The crude was purified using EtOAc/MeOH 9.5:0.5 and EtOAc/MeOH 8:2 as eluents to give a white solid; yield 66%; IR (neat): 3232, 3129, 3064, 2925, 2854, 1687, 1637, 1602, 1544, 1433, 1287, 1200, 1050, 751, 699 cm^–1^; ^1^H NMR (400 MHz, CD_3_OD) δ 9.02 (br s, 1H), 8.51 (br s, 1H), 8.45 (s, 1H), 8.25 (d, *J* = 8.0 Hz, 1H), 7.83 (s, 1H), 7.77 (d, *J* = 7.8 Hz, 1H), 7.65 (d, *J* = 7.3 Hz, 1H), 7.51–7.44 (m, 4H), 7.39–7.36 (m, 2H), 7.23 (t, *J* = 8.0 Hz, 1H), 6.97–6.95 (m, 2H), 6.85 (dd, *J* = 7.6/1.8 Hz, 1H), 5.38 (s, 2H), 5.12 (dd, *J* = 13.2/5.2 Hz, 2H), 4.50 (s, 2H), 4.44 (t, *J* = 7.0 Hz, 2H), 3.98 (t, *J* = 6.3 Hz, 2H), 3.13 (t, *J* = 6.8 Hz, 2H), 2.88–2.73 (m, 3H), 2.44 (qd, *J* = 12.6/4.9 Hz, 1H), 2.20–2.15 (m, 1H), 1.93 (quint, *J* = 6.9 Hz, 2H), 1.81–1.74 (m, 4H), 1.54 (quint, *J* = 7.0 Hz, 2H), 1.30–1.27 (m, 6H), 1.08–1.03 (m, 2H) ppm; ^13^C NMR (101 MHz, CD_3_OD) δ 173.1, 171.4, 170.6, 169.5, 164.9, 159.1, 148.2, 147.8, 146.0, 144.1, 141.8, 139.6, 136.6, 134.6, 133.5, 132.7, 132.6, 129.7, 129.4, 125.0, 128.8, 127.3 (2C), 127.0, 126.1, 124.1, 123.6, 121.7, 120.8, 120.4, 114.8, 113.6, 67.6, 52.4, 52.0, 50.2, 47.2, 39.3, 31.0, 29.7, 28.6 (2C), 28.4, 28.3, 26.2, 25.9, 25.3, 24.8, 22.7 ppm; HRMS (ESI), *m*/*z* [M + H]^+^ calcd for C_49_H_54_N_11_O_6_ 892.42585, found 892.42511.

##### 3′-((6-(1-(2-((2-(2,6-Dioxopiperidin-3-yl)-1-oxoisoindolin-4-yl)­amino)-2-oxoethyl)-1*H*-1,2,3-triazol-4-yl)­hexyl)­oxy)-*N*-(7-(4-(pyridin-3-yl)-1*H*-1,2,3-triazol-1-yl)­heptyl)-[1,1′-biphenyl]-2-carboxamide (**U5**)

The compound was prepared from **48**. The crude was purified using EtOAc/MeOH 9.5:0.5 and EtOAc/MeOH 9:1 as eluents to give a white solid; yield 66%; IR (neat): 3232, 3131, 3063, 2930, 2856, 1694, 1641, 1603, 1548, 1434, 1288, 1202, 1050, 753, 699 cm^–1^; ^1^H NMR (400 MHz, CD_3_OD) δ 9.02 (br s, 1H), 8.51 (br s, 1H), 8.43 (s, 1H), 8.24 (d, *J* = 7.9 Hz, 1H); 7.81 (s, 1H), 7.76 (d, *J* = 7.9 Hz, 1H), 7.65 (d, *J* = 7.4 Hz, 1H), 7.51–7.36 (m, 6H), 7.24 (t, *J* = 8.2 Hz, 1H), 6.97–6.95 (m, 2H), 6.85 (d, *J* = 7.5 Hz, 1H), 5.36 (s, 2H), 5.12 (dd, *J* = 13.2/5.2 Hz, 1H), 4.48 (s, 2H), 4.44 (t, *J* = 7.0 Hz, 2H), 3.96 (t, *J* = 6.3 Hz, 2H), 3.13 (t, *J* = 6.8 Hz, 2H), 2.92–2.71 (m, 4H), 2.43 (qd, *J* = 13.1/4.7 Hz, 1H), 2.18–2.15 (m, 1H), 1.93 (quint, *J* = 6.8 Hz, 2H), 1.77–1.69 (m, 4H), 1.54–1.43 (m, 4H), 1.31–1.23 (m, 6H), 1.07–1.06 (m, 2H) ppm; ^13^C NMR (101 MHz, CD_3_OD) δ 173.2, 171.5, 170.7, 169.5, 164.9, 159.0, 148.1, 147.9, 145.8, 144.0, 141.7, 139.6, 136.5, 134.5, 133.4, 132.7, 132.6, 129.7, 129.4, 129.0, 128.8, 127.3 (2C), 127.0, 126.0, 124.3, 123.7, 121.7, 120.7, 120.3, 114.6, 113.5, 67.6, 52.3, 51.9, 50.2, 47.1, 39.3, 30.9, 29.8, 28.9, 28.8, 28.4 (2C), 28.3, 26.2, 25.9, 25.4, 24.8, 22.7 ppm; HRMS (ESI), *m*/*z* [M + H]^+^ calcd for C_50_H_56_N_11_O_6_ 906.44150, found 906.43929.

##### 3′-((7-(1-(2-((2-(2,6-Dioxopiperidin-3-yl)-1-oxoisoindolin-4-yl)­amino)-2-oxoethyl)-1*H*-1,2,3-triazol-4-yl)­heptyl)­oxy)-*N*-(7-(4-(pyridin-3-yl)-1*H*-1,2,3-triazol-1-yl)­heptyl)-[1,1′-biphenyl]-2-carboxamide (**U6**)

The compound was prepared from **49**. The crude was purified using EtOAc/MeOH 9.5:0.5 and EtOAc/MeOH 9:1 as eluents to give a white solid; yield 45%; IR (neat): 3233, 3130, 3055, 2928, 2854, 1689, 1627, 1602, 1547, 1433, 1286, 1202, 1051, 753, 699 cm^–1^; ^1^H NMR (400 MHz, CD_3_OD) δ 9.02 (br s, 1H), 8.50 (br s, 1H), 8.44 (s, 1H), 8.25 (dt, *J* = 8.0/1.7 Hz, 1H), 7.81 (s, 1H), 7.77 (dd, *J* = 7.9/0.7 Hz, 1H), 7.64 (d, *J* = 7.1 Hz, 1H), 7.51–7.44 (m, 4H), 7.40–7.36 (m, 2H), 7.24 (t, *J* = 8.2 Hz, 1H), 6.97–6.95 (m, 2H), 6.86–6.84 (m, 1H), 5.37 (s, 2H), 5.12 (dd, *J* = 13.2/5.2 Hz, 1H), 4.50 (s, 2H), 4.44 (t, *J* = 7.0 Hz, 2H), 3.95 (t, *J* = 6.4 Hz, 2H), 3.13 (t, *J* = 6.8 Hz, 2H), 2.92–2.83 (m, 1H), 2.79–2.78 (m, 1H), 2.71 (t, *J* = 7.5 Hz, 2H), 2.44 (qd, *J* = 13.0/4.8 Hz, 1H), 2.20–2.14 (m, 1H), 1.93 (quint, *J* = 6.9 Hz, 2H), 1.77–1.67 (m, 4H), 1.47–1.39 (m, 6H), 1.32–1.25 (m, 6H), 1.09–1.05 (m, 2H) ppm; ^13^C NMR (101 MHz, CD_3_OD) δ 173.1, 171.4, 170.6, 169.5, 164.9, 159.1, 148.2, 148.0, 146.0, 144.0, 141.7, 139.6, 136.5, 134.7, 133.4, 132.7, 132.6, 129.7, 129.4, 129.0, 128.3, 127.3 (2C), 126.9, 126.1, 124.1, 123.6, 121.6, 120.7, 120.4, 114.8, 113.6, 67.8, 52.4, 51.9, 50.2, 47.2, 39.3, 31.0, 29.7, 28.9, 28.8, 28.6 (2C), 28.4, 28.3, 26.2, 25.9, 25.6, 24.9, 22.7 ppm; HRMS (ESI), *m*/*z* [M + H]^+^ calcd for C_51_H_58_N_11_O_6_ 920.45715, found 920.45683.

##### 3′-((8-(1-(2-((2-(2,6-Dioxopiperidin-3-yl)-1-oxoisoindolin-4-yl)­amino)-2-oxoethyl)-1*H*-1,2,3-triazol-4-yl)­octyl)­oxy)-*N*-(7-(4-(pyridin-3-yl)-1*H*-1,2,3-triazol-1-yl)­heptyl)-[1,1′-biphenyl]-2-carboxamide (**U7**)

The compound was prepared from **50**. The crude was purified using EtOAc and EtOAc/MeOH 9:1 to give a white solid; yield 64%; IR (neat):3222, 3129, 3057, 2923, 2852, 1686, 1637, 1602, 1543, 1433, 1286, 1201, 1050, 756, 699 cm^–1^; ^1^H NMR (400 MHz, CD_3_OD) δ 9.02 (br s, 1H), 8.50 (br s, 1H), 8.46 (s, 1H), 8.25 (d, *J* = 7.2 Hz, 1H), 7.81 (s, 1H), 7.78 (d, *J* = 7.0 Hz, 1H), 7.64 (d, *J* = 7.5 Hz, 1H), 7.51–7.34 (m, 6H), 7.24 (t, *J* = 8.0 Hz, 1H), 6.97–6.92 (m, 2H), 6.86–6.84 (m, 1H), 5.38 (s, 2H), 5.13 (dd, *J* = 13.2/5.2 Hz, 1H), 4.50 (s, 2H), 4.45 (t, *J* = 7.1 Hz, 2H), 3.95 (t, *J* = 6.4 Hz, 2H), 3.13 (t, *J* = 6.9 Hz, 2H), 2.80–2.75 (m, 1H), 2.74 (t, *J* = 7.1 Hz, 2H), 2.50–2.39 (qd, *J* = 13.1/4.8 Hz, 1H), 2.19–2.14 (m, 1H), 1.93 (quint, *J* = 7.0 Hz, 2H), 1.77–1.66 (m, 4H), 1.44–1.28 (m, 14H), 1.07–1.06 (m, 2H) ppm; ^13^C NMR (101 MHz, CD_3_OD) δ 173.1, 171.4, 170.6, 169.5, 165.0, 159.1, 148.2, 148.0, 146.0, 144.0, 141.7, 139.6, 136.5, 134.7, 133.4, 132.7, 132.6, 129.7, 129.4, 129.0, 128.8, 127.3 (2C), 126.9, 126.1, 124.1, 123.5, 121,6, 120.7, 120.4, 114.7, 113.6, 67.8, 52.4, 51.9, 50.2, 47.2, 39.4, 30.9, 29.7, 28.9 (2C), 28.8 (2C), 28.6, 28.4, 28.3, 26.2, 25.9, 25.7, 24.9, 22.7 ppm; HRMS (ESI), *m*/*z* [M + H]^+^ calcd for C_52_H_60_N_11_O_6_ 934.47280, found 934.47196.

##### 3′-((9-(1-(2-((2-(2,6-Dioxopiperidin-3-yl)-1-oxoisoindolin-4-yl)­amino)-2-oxoethyl)-1*H*-1,2,3-triazol-4-yl)­nonyl)­oxy)-*N*-(7-(4-(pyridin-3-yl)-1*H*-1,2,3-triazol-1-yl)­heptyl)-[1,1′-biphenyl]-2-carboxamide (**U8**)

The compound was prepared from **51**. The crude was purified using EtOAc and EtOAc/MeOH 9:1 to give a white solid; yield 44%; IR (neat): 3232, 3131, 3056, 2924, 2852, 1689, 1636, 1602, 1545, 1433, 1286, 1201, 1050, 752, 699 cm^–1^; ^1^H NMR (400 MHz, CD_3_OD) δ 9.05 (br s, 1H), 8.53 (br s, 1H), 8.45 (s, 1H), 8.26 (d, *J* = 7.9 Hz, 1H), 7.81 (s, 1H), 7.79 (dd, *J* = 8.0/0.6 Hz, 1H), 7.66 (d, *J* = 7.0 Hz, 1H), 7.52–7.36 (m, 6H), 7.24 (t, *J* = 8.2 Hz, 1H), 6.97–6.95 (m, 2H), 6.87–6.84 (m, 1H), 5.38 (s, 2H), 5.13 (dd, *J* = 13.2/5.2 Hz, 1H), 4.51 (s, 2H), 4.45 (t, *J* = 7.1 Hz, 2H), 3.85 (t, *J* = 6.4 Hz, 2H), 3.14 (t, *J* = 6.9 Hz, 2H), 2.93–2.84 (m, 1H), 2.80–2.74 (m, 1H), 2.70 (t, *J* = 7.5 Hz, 2H), 2.45 (qd, *J* = 13.3/4.9 Hz, 1H), 2.20–2.15 (m, 1H), 1.94 (quint, *J* = 6.9 Hz, 2H), 1.73 (quint, *J* = 6.7 Hz, 2H), 1.70–1.64 (m, 2H), 1.46–1.27 (m, 16H), 1.06 (quint, *J* = 7.3 Hz, 2H) ppm; ^13^C NMR (101 MHz, CD_3_OD) δ 173.1, 171.4, 170.6, 169.5, 165.0, 159.1, 148.2, 148.0, 146.0, 144.0, 141.7, 139.6, 136.5, 134.7, 133.4, 132.7, 136.6, 129.7, 129.4, 129.0, 128.8, 127.3 (2C), 126.9, 126.1, 124.0, 123.5, 121.6, 120.7, 120.4, 114.7, 113.6, 67.8, 52.4, 51.9, 50.2, 47.2, 39.3, 31.0, 29.7, 29.0, 29.0 (2C), 28.9, 28.8, 28.7, 28.4, 28.3, 26.2, 25.9, 25.7, 24.9, 22.7 ppm; HRMS (ESI), *m*/*z* [M + H]^+^ calcd for C_53_H_62_N_11_O_6_ 948.48845, found 948.48849.

##### 3′-((10-(1-(2-((2-(2,6-Dioxopiperidin-3-yl)-1-oxoisoindolin-4-yl)­amino)-2-oxoethyl)-1*H*-1,2,3-triazol-4-yl)­decyl)­oxy)-*N*-(7-(4-(pyridin-3-yl)-1*H*-1,2,3-triazol-1-yl)­heptyl)-[1,1′-biphenyl]-2-carboxamide (**U9**)

The compound was prepared from **52**. The crude was purified using EtOAc/MeOH 9.5:0.5 and EtOAc/MeOH 8.5:1.5 to give a white solid; yield 70%; IR (neat): 3232, 3139, 3056, 2923, 2852, 1693, 1644, 1602, 1546, 1433, 1286, 1201, 1050, 752, 699 cm^–1^; ^1^H NMR (400 MHz, CD_3_OD) δ 9.06 (br s, 1H), 8.55 (br s, 1H), 8.45 (s, 1H), 8.26 (d, *J* = 7.9 Hz, 1H), 7.80 (s, 1H), 7.78 (dd, *J* = 8.0/0.8 Hz, 1H), 7.65 (d, *J* = 7.5 Hz, 1H), 7.52–7.36 (m, 6H), 7.24 (t, *J* = 8.1 Hz, 1H), 6.97–6.95 (m, 2H), 6.87–6.84 (dt, *J* = 9.2/1.8 Hz, 1H), 5.36 (s, 2H), 5.13 (dd, *J* = 13.2/5.2 Hz, 1H), 4.50 (s, 2H), 4.45 (t, *J* = 7.1 Hz, 2H), 3.96 (t, *J* = 6.4 Hz, 2H), 3.14 (t, *J* = 6.9 Hz, 2H), 2.89–2.79 (m, 1H), 2.78–2.74 (m, 1H), 2.70 (t, *J* = 7.5 Hz, 2H), 2.45 (qd, *J* = 13.1/4.6 Hz, 1H), 2.20–2.14 (m, 1H), 1.94 (quint, *J* = 7.0 Hz, 2H), 1.77–1.64 (m, 4H), 1.45 (quint, *J* = 7.0 Hz, 2H), 1.36–1.28 (m, 16H), 1.07 (quint, *J* = 7.4 Hz, 2H) ppm; ^13^C NMR (101 MHz, CD_3_OD) δ 173.2, 171.5, 170.7, 169.5, 164.9, 159.0, 148.1, 148.0, 145.9, 144.0, 141.7, 139.6, 136.5, 134.6, 133.4, 132.7, 132.6, 129.7, 129.4, 129.0, 128.8, 127.3 (2C), 127.0, 126.0, 124.3, 123.6, 121.7, 120.7, 120.3, 114.6, 113.5, 67.7, 52.3, 51.9, 50.2, 47.1, 39.3, 30.9, 29.8, 29.2, 29.1, 29.0 (3C), 28.9, 28.7, 28.4, 28.3, 26.2, 25.9, 25.8, 24.9, 22.7 ppm; HRMS (ESI), *m*/*z* [M + H]^+^ calcd for C_54_H_64_N_11_O_6_ 962.50410, found 962.50260.

##### 3′-(2-((1-(2-((2-(2,6-Dioxopiperidin-3-yl)-1-oxoisoindolin-4-yl)­amino)-2-oxoethyl)-1*H*-1,2,3-triazol-4-yl)­methoxy)­ethoxy)-*N*-(7-(4-(pyridin-3-yl)-1*H*-1,2,3-triazol-1-yl)­heptyl)-[1,1′-biphenyl]-2-carboxamide (**U10**)

The compound was prepared from alkyne **53**. The crude was purified using EtOAc and EtOAc/MeOH 9:1 as eluents. White solid; yield 64%; IR (neat) 3232, 3140, 3056, 2931, 2859, 1694, 1636, 1603, 1548, 1434, 1203, 1051, 753 cm^–1^; ^1^H NMR (400 MHz, CD_3_OD) δ 9.02 (s, 1H), 8.50–8.48 (m, 2H), 8.26 (dt, *J* = 8.0/1.9 Hz, 1H), 8.08 (s, 1H), 7.76 (d, *J* = 7.9 Hz, 1H), 7.63 (d, *J* = 7.3 Hz, 1H), 7.50–7.36 (m, 6H), 7.24 (t, *J* = 8.2 Hz, 1H), 6.98–6.97 (m, 2H), 6.87–6.85 (dd, *J* = 8.1/1.8 1H), 5.43 (s, 2H), 5.14 (dd, *J* = 13.3/5.1 Hz, 1H), 4.73 (s, 2H), 4.47 (s, 2H), 4.43 (t, *J* = 7.0 Hz, 2H), 4.14–4.13 (m, 2H), 3.90–3.87 (m, 2H), 3.12 (t, *J* = 6.8 Hz, 2H), 2.93–2.84 (m, 1H), 2.77–2.71 (m, 1H), 2.46–2.36 (m, 1H), 2.17–2.12 (m, 1H), 1.89 (quint, *J* = 6.9 Hz, 2H), 1.28–1.22 (m, 6H), 0.98 (quint, *J* = 6.9 Hz, 2H) ppm; ^13^C NMR (101 MHz, CD_3_OD) δ 173.1, 171.4, 170.6, 169.4, 164.7, 158.8, 148.1, 146.0, 144.8, 144.0, 141.8, 139.5, 136.6, 134.7, 133.4, 132.7, 132.5, 129.7, 129.4, 129.0, 128.8, 127.5, 127.3, 127.0, 126.1, 125.4, 124.1, 121.6, 121.1, 120.4, 114.9, 113.7, 68.8, 67.4, 63.9, 52.4, 52.0, 50.2, 47.1, 39.3, 30.9, 29.7, 28.4, 28.2, 26.1, 25.9, 22.7 ppm; HRMS (ESI) *m*/*z* calcd for C_47_H_50_N_11_O_7_ [M + H]^+^ 880.38947, found 880.38806.

##### 3′-(2-(2-((1-(2-((2-(2,6-Dioxopiperidin-3-yl)-1-oxoisoindolin-4-yl)­amino)-2-oxoethyl)-1*H*-1,2,3-triazol-4-yl)­methoxy)­ethoxy)­ethoxy)-*N*-(7-(4-(pyridin-3-yl)-1*H*-1,2,3-triazol-1-yl)­heptyl)-[1,1′-biphenyl]-2-carboxamide (**U11**)

The compound was prepared from alkyne **54**. The crude was purified using EtOAc and EtOAc/MeOH 9:1 as eluents. White solid; yield 64%; IR (neat): 3231, 3130, 3056, 2923, 2855, 1687, 1636, 1602, 1547, 1433, 1201, 1051, 752 cm^–1^; ^1^H NMR (400 MHz, CD_3_OD) δ 9.03 (s, 1H), 8.49 (s, 1H), 8.47 (s, 1H), 8.28 (d, *J* = 8.0 Hz, 1H), 8.06 (s, 1H), 7.77 (d, *J* = 7.9 Hz, 1H), 7.63 (d, *J* = 7.5 Hz, 1H), 7.53–7.35 (m, 6H), 7.22 (t, *J* = 8.1 Hz, 1H), 6.98–6.96 (m, 2H), 6.86 (d, *J* = 7.2 Hz, 2H), 5.36 (s, 2H), 5.13 (dd, *J* = 12.3/5.1 Hz, 1H), 4.66 (s, 2H), 4.46–4.43 (m, 4H), 4.10 (quint, *J* = 4.6 Hz, 2H), 3.81 (quint, *J* = 4.5 Hz, 2H), 3.70 (br s, 4H), 3.13 (t, *J* = 6.8 Hz, 2H), 2.92–2.83 (m, 1H), 2.76–2.72 (m, 1H), 2.40–2.33 (m, 1H), 2.15–2.11 (m, 1H), 1.91 (quint, *J* = 6.8 Hz, 2H), 1.30–1.24 (m, 6H), 1.01 (quint, *J* = 7.0 Hz, 2H) ppm; ^13^C NMR (101 MHz, CD_3_OD) δ 173.1, 171.4, 170.6, 169.4, 164.7, 158.8, 148.0, 145.8, 144.8, 143.9, 141.8, 139.5, 136.5, 134.5, 133.6, 132.7, 132.6, 129.7, 129.4, 129.1, 128.8, 127.5, 127.3, 127.0, 125.9, 125.5, 124.3, 121.8, 121.0, 120.3, 114.7, 113.6, 70.4, 69.5, 69.4, 67.4, 63.7, 52.3, 31.9, 50.2, 47.0. 39.3, 30.9, 29.7, 28.4, 28.3, 26.2, 25.9, 22.7 ppm; HRMS (ESI) *m*/*z* calcd for C_49_H_54_N_11_O_8_ [M + H]^+^ 924.41568, found 924.41534.

##### 3′-(2-(2-(2-((1-(2-((2-(2,6-Dioxopiperidin-3-yl)-1-oxoisoindolin-4-yl)­amino)-2-oxoethyl)-1*H*-1,2,3-triazol-4-yl)­methoxy)­ethoxy)­ethoxy)­ethoxy)-*N*-(7-(4-(pyridin-3-yl)-1*H*-1,2,3-triazol-1-yl)­heptyl)-[1,1′-biphenyl]-2-carboxamide (**U12**)

The compound was prepared from alkyne **55**. The crude was purified using EtOAc and EtOAc/MeOH 9:1 as eluents. White solid; yield 41%; IR (neat) 3218, 3129, 3049, 2920, 2856, 1686, 1637, 1602, 1543, 1432, 1201, 1051, 752 cm^–1^; ^1^H NMR (400 MHz, CD_3_OD) δ 9.02 (s, 1H), 8.51 (s, 1H), 8.44 (s, 1H), 8.26 (d, *J* = 8.0 Hz, 1H), 8.03 (s, 1H), 7.76 (d, *J* = 8.0 Hz, 1H), 7.65 (d, *J* = 7.4 Hz, 1H), 7.51–7.35 (m, 6H), 7.23 (t, *J* = 8.0 Hz, 1H), 6.98–6.96 (m, 2H), 6.87 (d, *J* = 7.4 Hz, 1H), 5.36 (s, 2H), 5.12 (dd, *J* = 13.2/5.2 Hz, 1H), 4.66 (s, 2H), 4.47–4.43 (m, 4H), 4.13–4.10 (m, 2H), 3.82 (t, *J* = 4.7 Hz, 2H), 3.68–3.65 (m, 8H), 3.14 (t, *J* = 6.8 Hz, 2H), 2.93–2.83 (m, 1H), 2.79–2.74 (m, 1H), 2.46–2.35 (m, 1H), 2.17–2.14 (m, 1H), 1.93 (quint, *J* = 6.8 Hz, 2H), 1.32–1–25 (m, 6H), 1.07 (quint, *J* = 6.8 Hz, 2H) ppm; ^13^C NMR (101 MHz, CD_3_OD) δ 173.1, 171.4, 170.6, 169.4, 164.7, 158.8, 148.2, 146.0, 144.9, 144.0, 141.8, 139.5, 136.5, 134.7, 133.4, 132.7, 132.5, 129.7, 129.4, 129.1, 128.8, 128.3, 127.3, 127.0 126.0, 125.4, 124.1, 121.6, 121.1, 120.4, 114.8, 113.7, 70.4, 70.2, 70.1, 69.5, 69.4, 67.5, 63.7, 52.3, 51.9, 50.2, 47.1, 39.3, 30.9, 29.7, 28.4, 28.3, 26.2, 25.9, 22.7 ppm; HRMS (ESI) *m*/*z* calcd for C_51_H_58_N_11_O_9_ [M + H]^+^ 968.44190, found 968.4411.

##### 3′-((1-(1-(2-((2-(2,6-Dioxopiperidin-3-yl)-1-oxoisoindolin-4-yl)­amino)-2-oxoethyl)-1*H*-1,2,3-triazol-4-yl)-2,5,8,11-tetraoxatridecan-13-yl)­oxy)-*N*-(7-(4-(pyridin-3-yl)-1*H*-1,2,3-triazol-1-yl)­heptyl)-[1,1′-biphenyl]-2-carboxamide (**U13**)

The compound was prepared from alkyne **56**. The crude was purified using EtOAc and EtOAc/MeOH 9:1 as eluents. White solid; yield 50%; IR (neat) 3232, 3139, 3057, 2921, 2854, 1693, 1634, 1602, 1550, 1433, 1201, 1099, 752 cm^–1^; ^1^H NMR (400 MHz, CD_3_OD) δ 9.02 (br s, 1H), 8.51 (d, *J* = 4.0 Hz, 1H), 8.44 (s, 1H), 8.26 (dt, *J* = 8.0, 1.9 Hz, 1H), 8.03 (s, 1H), 7.76 (d, *J* = 8.0 Hz, 1H), 7.64 (d, *J* = 7.5 Hz, 1H), 7.51–7.44 (m, 4H), 7.40–7.35 (m, 2H), 7.23 (t, *J* = 8.2 Hz, 1H), 6.98–6.96 (m, 2H), 6.88–6.85 (m, 1H), 5.37 (s, 2H), 5.12 (dd, *J* = 13.2/5.2 Hz, 1H), 4.64 (s, 2H), 4.47–4.43 (m, 4H), 4.10 (t, *J* = 4.9 Hz, 2H), 3.80 (t, *J* = 4.8 Hz, 2H), 3.68–3.60 (m, 12H), 3.14 (t, *J* = 6.9 Hz, 2H), 2.92–2.83 (m, 1H), 2.79–2.72 (m, 1H), 2.46–2.35 (m, 1H), 2.16–2.10 (m, 1H), 1.93 (quint, *J* = 7.0 Hz, 2H), 1.34–1.23 (m, 6H), 1.07 (quint, *J* = 7.2 Hz, 2H) ppm; ^13^C NMR (101 MHz, CD_3_OD) δ 173.1, 171.4, 170.6, 169.4, 164.7, 158.8, 148.2, 146.0, 144.9, 144.1, 141.8, 139.5, 136.5, 134.6, 133.4, 132.7, 132.6, 129.7, 129.4, 129.1, 128.8, 127.4 (2C), 127.0, 126.0, 125.4, 124.1, 121.7, 121.1, 120.4, 114.9, 113.7, 70.4, 70.2 (2C), 70.2, 70.1, 69.5, 69.4, 67.4, 63.7, 52.3, 51.9, 50.2, 47.1, 39.3, 30.9, 29.7, 28.4, 28.3, 26.2, 25.9, 22.7 ppm; HRMS (ESI) *m*/*z* calcd for C_53_H_62_N_11_O_10_ [M + H]^+^ 1012.46811, found 1012.46742.

### Synthesis of NAMPT Targeting PROTACs **U14–21**

#### Synthesis of (2*S*,4*R*)-1-((*S*)-2-(4-Azidobutanamido)-3,3-dimethylbutanoyl)-4-hydroxy-*N*-(4-(4-methylthiazol-5-yl)­benzyl)­pyrrolidine-2-carboxamide (**61**)

NaN_3_ (1.20 g, 15.4 mmol, 1.2 equiv) was added portionwise to a solution of ethyl 4-bromobutanoate (3.00 g, 15.4 mmol, 1 equiv) in DMF (28 mL) and the reaction was stirred overnight at 80 °C. Upon completion, the reaction was diluted with water and extracted with EtOAc (×3). The combined organic layers were washed with water (×2), brine (×1), dried over anhydrous Na_2_SO_4_ and the solvent was evaporated under vacuum to give ethyl 4-azidobutanoate (**59**) as a colorless oil (1.04 g 43% yield); ^1^H NMR (400 MHz, CDCl_3_) δ 4.03 (q, *J* = 4.1 Hz, 2H), 3.25 (t, *J* = 6.7 Hz, 2H), 2.30 (t, *J* = 7.3 Hz, 2H), 1.81 (q, *J* = 6.9 Hz, 2H), 1.16 (t, *J* = 7.2 Hz, 3H) ppm; ^13^C NMR (101 MHz, CDCl_3_) δ 172.5, 60.3, 50.5, 31.0, 24.1, 14.0 ppm.

LiOH (0.48 g, 19.9 mmol, 3 equiv) was added to a solution of **59** (1.04 g, 6.61 mmol, 1 equiv) in a solution of THF–water 1:1 (10 mL) and the reaction was stirred overnight at 40 °C. Upon completion, the reaction was quenched by adding 3 M HCl aqueous solution (pH ∼ 1–2) and then extracted with EtOAc (×4). The combined organic layers were then dried over anhydrous Na_2_SO_4_, filtered and the solvent was evaporated under vacuum to afford 4-azidobutanoic acid (**60**) as a colorless oil (0.83 g 98% yield); ^1^H NMR (400 MHz, CDCl_3_) δ 10.55 (br s, 1H), 3.36 (t, *J* = 6.72 Hz, 2H), 2.46 (t, *J* = 7.28 Hz, 2H), 1.90 (quint, *J* = 6.97 Hz, 2H) ppm; ^13^C NMR (101 MHz, CDCl_3_) δ 179.1, 50.4, 30.9, 23.9 ppm.

DIPEA (0.533 mL, 3.06 mmol, 4.5 equiv) and HATU (0.323 g, 0.850 mmol, 1.25 equiv) were added to a solution of **60** (0.0877 g, 0.680 mmol, 1 equiv) in DMF (10 mL) and it was stirred at room temperature for 5 min. Subsequently VHL ligand **10** (0.370 g, 0.680 mmol, 1 equiv) was added portionwise and the reaction was stirred at room temperature for 5 h under nitrogen atmosphere. Upon completion, the reaction was diluted with brine and extracted with EtOAc (×2). The combined organic layers were washed with brine (×1), dried over anhydrous Na_2_SO_4_ and the solvent was evaporated under vacuum. The reaction crude was then purified by column chromatography using CH_2_Cl_2_/MeOH 98:2 and CH_2_Cl_2_/MeOH 97:3 as eluents. Azide **61** was obtained as a pale yellow solid (0.266 g 72% yield); ^1^H NMR (400 MHz, CD_3_OD) δ 8.88 (s, 1H), 8.66 (t, *J* = 5.9 Hz, 1H), 7.92 (d, *J* = 8.8 Hz, 1H), 7.47 (d, *J* = 8.2 Hz, 2H), 7.42 (d, *J* = 8.2 Hz, 2H), 4.66–4.53 (m, 4H), 4.41–4.36 (m, 1H), 3.95–3.80 (m, 2H), 3.37–3.34 (m, 2H), 2.48 (s, 3H), 2.43–2.33 (m, 2H), 2.27–2.07 (m, 2H), 1.86 (quint, *J* = 7.2 Hz, 2H), 1.06 (s, 9H) ppm; ^13^C NMR (101 MHz, CD_3_OD) Chemical shifts were referred to the main rotamer δ 173.5, 173.0, 170.9, 151.5, 147.6, 138.9, 132.0, 130.1, 129.0, 127.6, 69.7, 59.4, 57.8, 56.6, 50.5, 42.3, 37.5, 35.1, 32.1, 25.7, 24.7, 14.5 ppm; HRMS (ESI) *m*/*z* calcd for C_26_H_36_N_7_O_4_S [M + H]^+^ 542.25495, found 542.25461.

#### General Procedure B1 for the Click Reaction

Azide **61** (1.2 equiv), sodium ascorbate (1 equiv), CuSO_4_·5H_2_O (0.2 equiv) were added to a solution of the corresponding alkyne (**62**, **44–46**, **53–56**) (1 equiv) in a mixture of THF-H_2_O 2:1 (0.08 M) and the reaction was stirred for 5 h at 25 °C. Upon completion, the reaction was diluted with water and then extracted with CH_2_Cl_2_ (×3). The combined organic layers were washed with 1% EDTA aqueous solution (basified to pH 10 with few drops of 2 M NaOH) (×1) and water (×1). The solvent was evaporated under vacuum and the crude was purified by column chromatography using CH_2_Cl_2_/MeOH 98:2 and CH_2_Cl_2_/MeOH 9:1 as eluents.

##### (2*S*,4*R*)-1-((*S*)-3,3-Dimethyl-2-(4-(4-(((2′-((7-(4-(pyridin-3-yl)-1*H*-1,2,3-triazol-1-yl)­heptyl)­carbamoyl)-[1,1′-biphenyl]-3-yl)­oxy)­methyl)-1*H*-1,2,3-triazol-1-yl)­butanamido)­butanoyl)-4-hydroxy-*N*-(4-(4-methylthiazol-5-yl)­benzyl)­pyrrolidine-2-carboxamide (**U14**)

The compound was prepared from alkyne **62**. White solid; yield 53%; IR (neat) 3293, 3066, 2929, 2858, 1630, 1526, 1434, 1194, 1010, 790, 759, 697 cm^–1^; ^1^H NMR (400 MHz, CDCl_3_) δ 9.00 (br s, 1H), 8.67 (s, 1H), 8.54 (br s, 1H), 8.25 (d, *J* = 7.5 Hz, 1H), 8.07 (s, 1H), 7.73 (s, 1H), 7.65 (d, *J* = 7.2 Hz, 1H), 7.48–7.28 (m, 10H), 7.02–6.94 (m, 3H), 6.73 (d, *J* = 8.3 Hz, 1H), 5.41 (br s, 1H), 5.16 (s, 2H), 4.76 (t, *J* = 7.8 Hz, 1H), 4.59–4.31 (m, 8H), 4.07 (d, *J* = 11.2 Hz, 1H), 3.62 (d, *J* = 8.4 Hz, 1H), 3.10 (q, *J* = 6.1 Hz, 2H), 2.51–2.48 (m, 4H), 2.30–2.18 (m, 5H), 1.92 (quint, *J* = 6.5 Hz, 2H), 1.22–1.12 (m, 6H), 1.00–0.94 (m, 11H) ppm; ^13^C NMR (101 MHz, CDCl_3_) δ 171.8 (2C), 170.9, 169.6, 158.2, 150.3, 148.8, 148.4, 146.8, 144.5, 143.6, 141.8, 139.0, 138.2, 136.0, 133.2, 131.6, 130.9, 130.0 (2C), 129.9, 129.5, 128.6, 128.0, 127.8, 127.1, 123.9, 123.8, 121.6, 120.4, 114.8, 114.6, 70.0, 61.7, 58.6, 57.9, 56.8, 50.4, 49.2, 43.2, 39.6, 36.4, 35.1, 32.3, 30.1, 28.8, 28.5, 26.4, 26.3, 26.1, 25.8, 16.0 ppm; HRMS (ESI) *m*/*z* calcd. for C_56_H_67_N_12_O_6_S [M + H]^+^ 1035.50272, found 1035.50167.

##### (2*S*,4*R*)-1-((*S*)-3,3-Dimethyl-2-(4-(4-(2-((2′-((7-(4-(pyridin-3-yl)-1*H*-1,2,3-triazol-1-yl)­heptyl)­carbamoyl)-[1,1′-biphenyl]-3-yl)­oxy)­ethyl)-1*H*-1,2,3-triazol-1-yl)­butanamido)­butanoyl)-4-hydroxy-*N*-(4-(4-methylthiazol-5-yl)­benzyl)­pyrrolidine-2-carboxamide (**U15**)

The compound was prepared from alkyne **44**. White solid; yield 47%; IR (neat) 3272, 3067, 2928, 2857, 1632, 1531, 1434, 1204, 1020, 759, 698 cm^–1^; ^1^H NMR (400 MHz, CDCl_3_) δ 9.00 (br s, 1H), 8.67 (br s, 2H), 8.25 (d, *J* = 6.9 Hz, 1H), 8.04 (s, 1H), 7.65 (d, *J* = 7.3 Hz, 1H), 7.54–6.98 (m, 11H), 6.92–6.85 (m, 3H), 6.78 (br s, 1H), 5.39 (br s, 1H), 4.76 (br s, 1H), 4.59–4.32 (m, 8H), 4.31 (br s, 2H), 4.08 (d, *J* = 11.1 Hz, 1H), 3.62 (d, *J* = 9.0 Hz, 1H), 3.18–3.09 (m, 4H), 2.50 (br s, 4H), 2.28–2.17 (m, 5H), 1.92 (quint, *J* = 6.7 Hz, 2H), 1.26–1.11 (m, 6H), 0.97–0.94 (m, 11H) ppm; ^13^C NMR (101 MHz, CDCl_3_) δ 171.9, 171.4, 171.0, 169.6, 158.6, 150.3, 148.8, 148.4, 146.8, 144.5, 144.3, 141.7, 139.0, 138.2, 135.9, 133.1, 131.6, 130.8, 130.0 (2C), 129.8, 129.4, 128.6, 128.2, 128.0, 127.7, 122.6 (2C), 121.2, 120.3, 115.2, 113.7, 70.0, 66.8, 58.6, 57.9, 56.8, 50.4, 49.0, 43.1, 39.7, 36.4, 35.1, 32.3, 30.1, 28.8, 28.5, 26.5, 26.4, 26.2, 26.1, 26.0, 16.0 ppm; HRMS (ESI) *m*/*z* calcd. for C_57_H_69_N_12_O_6_S [M + H]^+^ 1049.51837, found 1049.51786.

##### (2*S*,4*R*)-1-((*S*)-3,3-Dimethyl-2-(4-(4-(3-((2′-((7-(4-(pyridin-3-yl)-1*H*-1,2,3-triazol-1-yl)­heptyl)­carbamoyl)-[1,1′-biphenyl]-3-yl)­oxy)­propyl)-1*H*-1,2,3-triazol-1-yl)­butanamido)­butanoyl)-4-hydroxy-*N*-(4-(4-methylthiazol-5-yl)­benzyl)­pyrrolidine-2-carboxamide (**U16**)

The compound was prepared from alkyne **45**. White solid; yield 56%; IR (neat) 3291, 3067, 2930, 2860, 1630, 1530, 1435, 1202, 1052, 791, 760, 698 cm^–1^; ^1^H NMR (400 MHz, CDCl_3_) δ 8.67 (br s, 1H), 8.29 (br s, 1H), 8.03 (s, 1H), 7.67 (d, *J* = 7.3 Hz, 1H), 7.46–7.29 (m, 11H), 6.98 (d, *J* = 7.3 Hz, 1H), 6.89–6.86 (m, 3H), 5.41 (br s, 1H), 4.78–4.36 (m, 9H), 4.09 (d, *J* = 10.9 Hz, 1H), 4.00 (br s, 1H), 3.63 (d, *J* = 10.2 Hz, 1H), 3.12 (q, *J* = 6.0 Hz, 2H), 2.89 (br s, 2H), 2.50 (br s, 4H), 2.16 (br s, 7H), 1.94 (br s, 2H), 1.27–1.16 (m, 6H), 1.00–0.95 (m, 11H) ppm; ^13^C NMR (101 MHz, CDCl_3_) δ 171.9, 171.7, 171.1, 169.6, 158.9, 150.3, 148.4 (2C), 146.9, 144.8 (2C), 141.7, 139.2, 138.2, 135.9, 132.9, 131.8, 130.8, 130.0 (2C), 129.7, 129.4, 128.6, 128.2, 128.0, 127.7, 121.7 (2C), 121.0, 120.4, 114.5, 114.3, 70.0, 67.0, 58.7, 57.9, 56.8, 50.4, 49.0, 43.1, 39.7, 36.6, 35.3, 32.4, 30.1, 28.8 (2C), 28.5, 26.5, 26.4, 26.2, 25.9, 22.1, 16.0 ppm; HRMS (ESI) *m*/*z* calcd. for C_58_H_71_N_12_O_6_S [M + H]^+^ 1063.53402, found 1063.53299.

##### (2*S*,4*R*)-1-((*S*)-3,3-Dimethyl-2-(4-(4-(4-((2′-((7-(4-(pyridin-3-yl)-1*H*-1,2,3-triazol-1-yl)­heptyl)­carbamoyl)-[1,1′-biphenyl]-3-yl)­oxy)­butyl)-1*H*-1,2,3-triazol-1-yl)­butanamido)­butanoyl)-4-hydroxy-*N*-(4-(4-methylthiazol-5-yl)­benzyl)­pyrrolidine-2-carboxamide (**U17**)

The compound was prepared from alkyne **46**. White solid; yield 61%; IR (neat) 3291, 3066, 2929, 2860, 1629, 1527, 1434, 1201, 1051, 791, 759, 698 cm^–1^; ^1^H NMR (400 MHz, CDCl_3_) δ 8.65 (br s, 1H), 8.23 (d, *J* = 5.9, 1H), 7.98 (s, 1H), 7.66 (d, *J* = 7.4 Hz, 1H), 7.45–7.25 (m, 11H), 6.96–6.84 (m, 3H), 6.70 (br s, 1H), 5.41 (t, *J* = 5.5 Hz, 1H), 4.75 (br s, 1H), 4.52–4.33 (m, 8H), 4.06–3.97 (m, 3H), 3.65–3.61 (m, 1H), 3.12 (q, *J* = 6.3 Hz, 2H), 2.75 (br s, 2H), 2.49 (br s, 4H), 2.24–2.14 (m, 5H), 1.93–1.83 (m, 6H), 1.26–1.15 (m, 6H), 1.05–0.94 (m, 11H) ppm; ^13^C NMR δ 171.9, 171.6, 171.1, 169.5, 159.0, 150.3, 148.8, 148.4, 146.5, 144.7, 141.7, 139.2, 138.2, 135.8, 132.9, 131.7, 130.8, 130.0, 129.9, 129.7, 129.4, 128.7, 128.2, 128.0, 127.7, 121.4 (2C), 121.0, 120.4, 114.7, 114.0, 70.0, 67.6, 58.7, 57.9, 56.8, 50.4, 49.0, 43.1, 39.7, 36.6, 35.3, 32.4, 30.1, 28.8, 28.7, 28.5, 26.5, 26.4, 26.2, 26.0, 25.9, 25.3, 16.0 ppm. HRMS (ESI) *m*/*z* calcd. for C_59_H_73_N_12_O_6_S [M + H]^+^ 1077.54967, found 1077.54962.

##### (2*S*,4*R*)-1-((*S*)-3,3-Dimethyl-2-(4-(4-((2-((2′-((7-(4-(pyridin-3-yl)-1*H*-1,2,3-triazol-1-yl)­heptyl)­carbamoyl)-[1,1′-biphenyl]-3-yl)­oxy)­ethoxy)­methyl)-1*H*-1,2,3-triazol-1-yl)­butanamido)­butanoyl)-4-hydroxy-*N*-(4-(4-methylthiazol-5-yl)­benzyl)­pyrrolidine-2-carboxamide (**U18**)

The compound was prepared from alkyne **53**. White solid; yield 59%; IR (neat) 3293, 3065, 2921, 2853, 1631, 1531, 1436, 1201, 1089, 1051, 759, 698 cm^–1^; ^1^H NMR (400 MHz, CDCl_3_) δ 9.03 (br s, 1H), 8.66 (s, 1H), 8.55 (br s, 1H), 8.28 (d, *J* = 7.9 Hz, 1H), 8.05 (s, 1H), 7.66 (dd, *J* = 7.4/1.1 Hz, 1H), 7.63 (s, 1H), 7.49–7.26 (m, 10H), 6.99 (d, *J* = 7.6 Hz, 1H), 6.93 (s, 1H), 6.87 (dd, *J* = 8.2/1.9 Hz, 1H), 6.76 (d, *J* = 8.5 Hz, 1H), 5.42 (t, *J* = 5.6 Hz, 1H), 4.78–4.32 (m, 11H), 4.14–4.07 (m, 3H), 3.89–3.87 (m, 2H), 3.64–3.61 (m, 1H), 3.14 (q, *J* = 6.2 Hz, 2H), 2.66 (br s, 1H), 2.50–2.42 (m, 4H), 2.28–2.14 (m, 5H), 1.90 (quint, *J* = 7.0 Hz, 2H), 1.27–1.17 (m, 6H), 1.01–0.89 (m, 11H) ppm; ^13^C NMR (101 MHz, CDCl_3_) δ 171.9, 171.6, 171.1, 169.6, 158.7, 150.3, 148.5, 148.4, 146.4, 144.8, 144.3, 141.7, 139.1, 138.2, 135.9, 133.5, 131.6, 130.8, 130.0 (2C), 129.7, 129.4, 128.7, 128.0, 127.7, 127.2, 124.0, 123.4, 121.4, 120.5, 114.8, 114.1, 70.0, 68.8, 67.4, 64.6, 58.7, 57.9, 56.9, 50.4, 49.1, 43.1, 39.7, 36.5, 35.2, 32.3, 30.1, 28.8, 28.5, 26.5, 26.3, 26.2, 25.9, 16.0 ppm; HRMS (ESI) *m*/*z* calcd for C_58_H_71_N_12_O_7_S [M + H]^+^ 1079.52894, found 1079.52824.

##### (2*S*,4*R*)-1-((*S*)-3,3-Dimethyl-2-(4-(4-((2-(2-((2′-((7-(4-(pyridin-3-yl)-1*H*-1,2,3-triazol-1-yl)­heptyl)­carbamoyl)-[1,1′-biphenyl]-3-yl)­oxy)­ethoxy)­ethoxy)­methyl)-1*H*-1,2,3-triazol-1-yl)­butanamido)­butanoyl)-4-hydroxy-*N*-(4-(4-methylthiazol-5-yl)­benzyl)­pyrrolidine-2-carboxamide (**U19**)

The compound was prepared from alkyne **54**. White solid; yield 76%; IR (neat) 3293, 3068, 2926, 2859, 1630, 1529, 1436, 1201, 1087, 1056, 842 cm^–1^; ^1^H NMR (400 MHz, CDCl_3_) δ 8.98 (br s, 1H), 8.63 (s, 1H), 8.49 (br s, 1H), 8.20 (d, *J* = 6.8 Hz, 1H), 8.02 (s, 1H), 7.60–7.56 (m, 3H), 7.43–7.22 (m, 9H), 6.94 (d, *J* = 7.5 Hz, 1H), 6.88–6.83 (m, 3H), 5.66 (br t, 1H), 4.70 (*J* = 7.8 Hz, 1H), 4.57–4.25 (m, 11H), 4.08–3.57 (m, 10H), 3.11 (q, *J* = 6.1 Hz, 2H), 2.46 (s, 3H), 2.32–2.03 (m, 6H), 1.89 (quint, *J* = 6.4 Hz, 2H), 1.25–1.16 (m, 6H), 1.00–0.92 (m, 11H) ppm; ^13^C NMR (101 MHz, CDCl_3_) δ 172.2, 171.5, 171.3, 169.7, 158.7, 150.3, 148.5, 148.3, 146.5, 144.7, 144.3, 141.6, 139.1, 138.3, 135.9, 133.4, 131.6, 130.7, 130.0 (2C), 129.7, 129.4, 128.5, 128.0, 127.7, 127.2, 124.0, 123.4, 121.4, 120.6, 114.9, 113.9, 70.6, 70.1, 69.6, 69.6, 67.4, 64.3, 58.8, 58.2, 56.7, 50.4, 49.1, 43.0, 39.7, 36.8, 35.1, 32.2, 30.1, 28.8, 28.5, 26.5, 26.3, 26.1, 25.9, 16.0 ppm; HRMS (ESI) *m*/*z* calcd for C_60_H_75_N_12_O_8_S [M + H]^+^ 1123.55515, found 1123.55526.

##### (2*S*,4*R*)-1-((*S*)-3,3-Dimethyl-2-(4-(4-((2-(2-(2-((2′-((7-(4-(pyridin-3-yl)-1*H*-1,2,3-triazol-1-yl)­heptyl)­carbamoyl)-[1,1′-biphenyl]-3-yl)­oxy)­ethoxy)­ethoxy)­ethoxy)­methyl)-1*H*-1,2,3-triazol-1-yl)­butanamido)­butanoyl)-4-hydroxy-*N*-(4-(4-methylthiazol-5-yl)­benzyl)­pyrrolidine-2-carboxamide (**U20**)

The compound was prepared from alkyne **55**. White solid; yield 55%; IR (neat) 3294, 3067, 2924, 2857, 1633, 1531, 1435, 1202, 1087, 1054, 760, 708 cm^–1^; ^1^H NMR (400 MHz, CDCl_3_) δ 9.04 (br s, 1H), 8.68 (s, 1H), 8.56 (br s, 1H), 8.31 (d, *J* = 7.8 Hz, 1H), 8.03 (s, 1H), 7.68–7.63 (m, 2H), 7.48–7.26 (m, 10H), 6.98 (d, *J* = 7.6 Hz, 1H), 6.93 (s, 1H), 6.87 (dd, *J* = 8.2/1.9 Hz, 1H), 6.78 (d, *J* = 8.3 Hz, 1H), 5.47 (t, *J* = 5.6 Hz, 1H), 4.76 (t, *J* = 8.2 Hz, 1H), 4.60–4.32 (m, 11H), 4.12–4.07 (m, 3H), 3.85–3.82 (m, 2H), 3.72–3.62 (m, 9H), 3.15 (q, *J* = 6.2 Hz, 2H), 2.51–2.35 (m, 4H), 2.25–2.11 (m, 5H), 1.95–1.90 (m, 2H), 1.27–1.19 (m, 6H), 1.04–0.95 (m, 11H) ppm; ^13^C NMR (101 MHz, CDCl_3_) δ 172.1, 171.6, 171.2, 169.9, 158.8, 150.3, 148.7, 148.3, 146.6, 144.8, 144.4, 141.7, 139.1, 138.3, 135.9, 133.3, 131.6, 130.7, 130.0, 129.9, 129.6, 129.4, 128.6, 128.0, 127.7, 127.0, 124.0, 123.5, 121.4, 120.4, 114.8, 114.1, 70.7, 70.4 (2C), 70.0, 69.7 (2C), 67.4, 64.4, 58.8, 58.0, 56.9, 50.4, 49.0, 43.1, 39.7, 36.7, 35.2, 32.1, 30.1, 28.8, 28.5, 26.4, 26.4, 26.2, 25.9, 16.0 ppm; HRMS (ESI) *m*/*z* calcd for C_62_H_79_N_12_O_9_S [M + H]^+^ 1167.58137, found 1167.58039.

##### (2*S*,4*R*)-1-((*S*)-3,3-Dimethyl-2-(4-(4-(13-((2′-((7-(4-(pyridin-3-yl)-1*H*-1,2,3-triazol-1-yl)­heptyl)­carbamoyl)-[1,1′-biphenyl]-3-yl)­oxy)-2,5,8,11-tetraoxatridecyl)-1*H*-1,2,3-triazol-1-yl)­butanamido)­butanoyl)-4-hydroxy-*N*-(4-(4-methylthiazol-5-yl)­benzyl)­pyrrolidine-2-carboxamide (**U21**)

The compound was prepared from alkyne **56**. White solid; yield 61%; IR (neat) 3294, 3067, 2927, 2859, 1632, 1529, 1436, 1202, 1088, 1055, 760, 698 cm^–1^; ^1^H NMR (400 MHz, CDCl_3_) δ 8.99 (br s, 1H), 8.64 (s, 1H), 8.51 (br s, 1H), 8.19 (s, 1H), 7.97 (s, 1H), 7.60–7.32 (m, 12H), 6.91–6.85 (m, 4H), 5.54 (br s, 1H), 4.70–4.38 (m, 11H), 4.09–4.02 (m, 3H), 3.81–3.61 (m, 15H), 3.12 (br s, 2H), 2.47–2.12 (m, 9H), 1.90 (br s, 2H), 1.24 (br s, 6H), 0.93 (br s, 11H) ppm; ^13^C NMR (101 MHz, CDCl_3_) δ 171.8, 171.6, 171.2, 169.5, 158.8, 150.3, 148.8, 148.4, 146.7, 145.0, 144.4, 141.7, 139.1, 138.3, 135.9, 133.2, 131.6, 130.8, 130.0, 129.9, 129.6, 129.4, 128.7, 128.0, 127.7, 127.0, 123.9, 123.3, 121.3, 120.3, 114.8, 114.1, 70.7, 70.5 (2C), 70.4, 70.4, 70.0, 69.7 (2C), 67.5, 64.5, 58.7, 57.9, 56.8, 50.4, 49.0, 43.1, 39.6, 36.6, 35.2, 32.2, 30.1, 28.8, 28.5, 26.5, 26.4, 26.2, 25.9, 16.0 ppm; HRMS (ESI) *m*/*z* calcd for C_64_H_83_N_12_O_10_S [M + H]^+^ 1211.60758, found 1211.60853.

### Synthesis of NAMPT Targeting PROTACs **U22–29**

Ethers **71–74** were synthesized from phenol **8** and the corresponding tosylated poly­(ethylene glycol) *tert*-butyl ester (**67–70**) following the general procedure A. The crude were purified by column chromatography using CH_2_Cl_2_/MeOH 98:2 as eluent.

#### General Procedure C for the *tert*-Butyl Ester Hydrolysis (**75–78**)

The corresponding *tert*-butyl ester (**71–74**) (1 equiv) was dissolved in a 4 N HCl solution in dioxane (0.2 M), and the reaction was stirred at 25 °C for 1 h. Upon completion, the solvent was evaporated under vacuum and the reaction crude was purified by column chromatography using CH_2_Cl_2_/MeOH 95:5 and CH_2_Cl_2_/MeOH 9:1.

#### General Procedure D for the Amide Coupling

VHL ligand **10** or lenalidomide (**9**) (1.1 equiv) and DIPEA (6 equiv) were added to a solution of the corresponding carboxylic acid (**75–78**) (1 equiv) in DMF (0.033 M). The solution was cooled to 0 °C, then HATU (1.5 equiv) was added. The reaction mixture was stirred at 25 °C for 5 h under nitrogen atmosphere. Upon completion, the reaction was diluted with water and extracted with CH_2_Cl_2_ (×2). The combined organic layers were washed with water (×1), brine (×1), dried over anhydrous Na_2_SO_4_ and the solvent was evaporated under vacuum. The crude was purified by column chromatography using CH_2_Cl_2_/MeOH 98:2 and CH_2_Cl_2_/MeOH 9:1 as eluents.

##### (2*S*,4*R*)-1-((*S*)-3,3-Dimethyl-2-(3-(2-((2′-((7-(4-(pyridin-3-yl)-1*H*-1,2,3-triazol-1-yl)­heptyl)­carbamoyl)-[1,1′-biphenyl]-3-yl)­oxy)­ethoxy)­propanamido)­butanoyl)-4-hydroxy-*N*-(4-(4-methylthiazol-5-yl)­benzyl)­pyrrolidine-2-carboxamide (**U22**)

The compound was prepared from carboxylic acid **75**. White solid; yield: 78%; IR (neat) 3294, 3067, 2927, 2860, 1632, 1526, 1434, 1199, 1110, 1050, 759, 698 cm^–1^; ^1^H NMR (400 MHz, CDCl_3_) δ 9.02 (br s, 1H), 8.64 (s, 1H), 8.53 (br s, 1H), 8.28 (d, *J* = 8.0 Hz, 1H), 8.05 (s, 1H), 7.62 (dd, *J* = 7.4/1.4 Hz, 1H), 7.51–7.24 (m, 10H), 7.03–6.85 (m, 4H), 5.51 (t, *J* = 5.8 Hz, 1H), 4.66 (t, *J* = 8.0 Hz, 1H), 4.55–4.28 (m, 6H), 4.12–4.05 (m, 2H), 3.99 (d, *J* = 11.2 Hz, 1H), 3.81–3.59 (m, 6H), 3.16–3.10 (m, 2H), 2.50–2.36 (m, 6H), 2.12–2.05 (m, 1H), 1.95–1.88 (quint, *J* = 7.0 Hz, 2H), 1.26–1.15 (m, 6H), 1.04–0.93 (m, 11H) ppm; ^13^C NMR (101 MHz, CDCl_3_) δ 171.6, 171.4, 171.1, 169.6, 158.7, 150.3, 148.4, 148.3, 146.3, 144.3, 141.7, 139.2, 138.3, 135.9, 133.6, 131.6, 130.8, 130.0 (2C), 129.7, 129.4, 128.6, 128.0, 127.7, 127.3, 124.1, 121.3, 120.5, 144.5, 114.4, 70.0, 69.6, 67.3 (2C), 58.6, 57.6, 56.8, 50.4, 43.1, 39.7, 36.8, 36.3, 35.3, 30.1, 28.8, 28.5, 26.4, 26.3, 26.2, 16.0 ppm; HRMS (ESI) *m*/*z* calcd for C_54_H_66_N_9_O_7_S [M + H]^+^ 984.48059, found 984.47881.

##### (2*S*,4*R*)-1-((*S*)-3,3-Dimethyl-2-(3-(2-(2-((2′-((7-(4-(pyridin-3-yl)-1*H*-1,2,3-triazol-1-yl)­heptyl)­carbamoyl)-[1,1′-biphenyl]-3-yl)­oxy)­ethoxy)­ethoxy)­propanamido)­butanoyl)-4-hydroxy-*N*-(4-(4-methylthiazol-5-yl)­benzyl)­pyrrolidine-2-carboxamide (**U23**)

The compound was prepared from carboxylic acid **76**. White solid; yield 58%; IR (neat) 3295, 3065, 2925, 2859, 1632, 1528, 1435, 1200, 1088, 759, 697 cm^–1^; ^1^H NMR (400 MHz, CDCl_3_) δ 8.99 (br s, 1H), 8.63 (s, 1H), 8.53 (br s, 1H), 8.22 (d, *J* = 7.9 Hz, 1H), 7.98 (s, 1H), 6.64 (d, *J* = 7.4 Hz, 1H), 7.51–7.24 (m, 10H), 7.07–6.86 (m, 4H), 5.50 (t, *J* = 5.7 Hz, 1H), 4.69 (t, *J* = 7.9 Hz, 1H), 4.55–4.27 (m, 6H), 4.10–4.08 (m, 2H), 4.02 (d, *J* = 11.3 Hz, 1H), 3.81 (t, *J* = 4.8 Hz, 2H), 3.71–3.59 (m, 7H), 3.13 (q, *J* = 6.7 Hz, 2H), 2.48–2.40 (m, 6H), 2.13–2.08 (m, 1H), 1.92 (quint, *J* = 7.0 Hz, 2H), 1.26–1.16 (m, 6H), 1.05–0.94 (m, 11H) ppm; ^13^C NMR (101 MHz, CDCl_3_) δ 171.7, 171.6, 171.0, 169.5, 158.8, 150.3, 149.0, 148.4, 146.9, 144.6, 141.7, 139.1, 138.2, 135.9, 133.0, 131.6, 130.8, 130.0, 129.9, 129.6, 129.4, 128.7, 128.0, 127.7, 127.0, 123.9, 121.3, 120.2, 114.7, 114.2, 70.7, 70.5, 70.0, 69.7, 67.5, 67.2, 58.6, 57.6, 56.7, 50.4, 43.1, 39.7, 36.7, 36.2, 35.1, 30.2, 28.8, 28.5, 26.4 (2C), 26.2, 16.0 ppm; HRMS (ESI) *m*/*z* calcd for C_56_H_70_N_9_O_8_S [M + H]^+^ 1028.50681, found 1028.50459.

##### (2*S*,4*R*)-1-((*S*)-14-(*tert*-Butyl)-12-oxo-1-((2′-((7-(4-(pyridin-3-yl)-1*H*-1,2,3-triazol-1-yl)­heptyl)­carbamoyl)-[1,1′-biphenyl]-3-yl)­oxy)-3,6,9-trioxa-13-azapentadecan-15-oyl)-4-hydroxy-*N*-(4-(4-methylthiazol-5-yl)­benzyl)­pyrrolidine-2-carboxamide (**U24**)

The compound was prepared from carboxylic acid **77**. White solid; yield 51%; IR (neat) 3293, 3066, 2922, 2860, 1629, 1528, 1437, 1200, 1087, 841 cm^–1^; ^1^H NMR (400 MHz, CDCl_3_) δ 9.00 (br s, 1H), 8.64 (s, 1H), 8.52 (br s, 1H), 8.21 (d, *J* = 7.7 Hz, 1H), 7.99 (s, 1H), 7.63 (d, *J* = 7.4 Hz, 1H), 7.49–7.24 (m, 10H), 7.05–6.87 (m, 4H), 5.59 (t, *J* = 5.5 Hz, 1H), 4.66 (t, *J* = 8.0 Hz, 1H), 4.53–4.30 (m, 6H), 4.11 (t, *J* = 4.2 Hz, 2H), 3.98 (d, *J* = 11.2 Hz, 1H), 3.83–3.82 (m, 2H), 3.68–3.54 (m, 11H), 3.14 (q, *J* = 6.5 Hz, 2H), 2.49–2.33 (m, 6H), 2.11–2.04 (m, 1H), 1.90 (quint, *J* = 7.2 Hz, 2H), 1.26–1.18 (m, 6H), 1.04–1.03 (m, 2H), 0.93 (s, 9H) ppm; ^13^C NMR (101 MHz, CDCl_3_) δ 172.1, 171.6, 171.1, 169.7, 158.7, 150.3, 148.9, 148.4, 146.9, 144.5, 141.7, 139.1, 138.3, 135.9, 133.1, 131.6, 130.8, 130.1, 130.0, 129.7, 129.4, 128.6, 128.0, 127.7, 127.0, 123.9, 121.4, 120.4, 114.9, 114.2, 70.5, 70.2 (3C), 70.0, 69.6, 67.5, 67.1, 58.7, 57.9, 56.6, 50.4, 43.1, 39.7, 36.4, 36.3, 35.0, 30.1, 28.8, 28.5, 26.4 (2C), 26.2, 16.1 ppm; HRMS (ESI) *m*/*z* calcd for C_58_H_74_N_9_O_9_S [M + H]^+^ 1072.53302, found 1072.53095.

##### (2*S*,4*R*)-1-((*S*)-17-(*tert*-Butyl)-15-oxo-1-((2′-((7-(4-(pyridin-3-yl)-1*H*-1,2,3-triazol-1-yl)­heptyl)­carbamoyl)-[1,1′-biphenyl]-3-yl)­oxy)-3,6,9,12-tetraoxa-16-azaoctadecan-18-oyl)-4-hydroxy-*N*-(4-(4-methylthiazol-5-yl)­benzyl)­pyrrolidine-2-carboxamide (**U25**)

The compound was prepared from carboxylic acid **78**. White solid; yield 58%; IR (neat) 3306, 3065, 2923, 2857, 1632, 1530, 1435, 1201, 1088, 841 cm^–1^; ^1^H NMR (400 MHz, CDCl_3_) δ 9.01 (br s, 1H), 8.66 (s, 1H), 8.53 (br s, 1H), 8.21 (d, *J* = 7.9 Hz, 1H), 7.99 (s, 1H), 7.63 (d, *J* = 7.4 Hz, 1H), 7.46–7.24 (m, 10H), 6.99–6.87 (m, 4H), 5.52 (t, *J* = 5.6 Hz, 1H), 4.66 (t, *J* = 7.9 Hz, 1H), 4.54–4.30 (m, 6H), 4.14–4.11 (m, 2H), 4.02–4.00 (m, 1H), 3.87–3.84 (m, 2H), 3.73–3.70 (m, 2H), 3.65–3.58 (m, 13H), 3.14 (q, *J* = 6.7 Hz, 2H), 2.49 (s, 3H), 2.44–2.41 (m, 3H), 2.17–2.09 (m, 1H), 1.92 (quint, *J* = 6.9 Hz, 2H), 1.26–1.17 (m, 6H), 1.05–0.93 (m, 11H) ppm; ^13^C NMR (101 MHz, CDCl_3_) δ 172.2, 171.5, 171.0, 169.6, 158.8, 150.3, 149.0 148.4, 146.9, 144.5, 141.7, 139.1, 138.3, 135.9, 133.1, 131.6, 130.8, 130.1, 130.0, 129.7, 129.4, 128.6, 128.0, 127.7, 127.0, 123.9, 121.4, 120.3, 114.8, 114.2, 70.4, 70.2, 70.1 (4C), 70.0, 69.6, 67.5, 67.1, 58.6, 57.8, 56.6, 50.4, 43.1, 39.7, 36.4, 36.3, 35.0, 30.2, 28.8, 28.5, 26.4 (2C), 26.2, 16.1 ppm; HRMS (ESI) *m*/*z* calcd for C_60_H_78_N_9_O_10_S [M + H]^+^ 1116.55924, found 1116.55836.

##### 3′-(2-(3-((2-(2,6-Dioxopiperidin-3-yl)-1-oxoisoindolin-4-yl)­amino)-3-oxopropoxy)­ethoxy)-*N*-(7-(4-(pyridin-3-yl)-1*H*-1,2,3-triazol-1-yl)­heptyl)-[1,1′-biphenyl]-2-carboxamide (**U26**)

The compound was prepared from carboxylic acid **75**. White solid; yield 43%; IR (neat) 3270, 3064, 2925, 2855, 1683, 1637, 1539, 1431, 1201, 1121, 1052, 753 cm^–1^; ^1^H NMR (400 MHz, CDCl_3_) δ 9.42 (s, 1H), 9.08 (s, 1H), 8.99 (s, 1H), 8.53 (d, *J* = 4.0 Hz, 1H), 8.20 (d, *J* = 7.9 Hz, 1H), 7.94 (s, 1H), 7.67 (d, *J* = 6.7 Hz, 1H), 7.58 (t, *J* = 6.7 Hz, 2H), 7.44–7.18 (m, 6H), 6.94 (d, *J* = 7.5 Hz, 1H), 6.88 (s, 1H), 6.78 (d, *J* = 6.6 Hz, 1H), 5.66 (t, *J* = 5.4 Hz, 1H), 5.03 (dd, *J* = 13.0/4.8 Hz, 1H), 4.39–4.35 (m, 4H), 4.13 (br s, 2H), 3.89–3.84 (m, 4H), 3.12 (q, *J* = 6.2 Hz, 2H), 2.71–2.68 (m, 3H), 2.28–2.11 (m, 2H), 2.03–2.00 (m, 1H), 1.87 (quint, *J* = 7.0 Hz, 2H), 1.21–1.20 (m, 6H), 1.00–0.98 (m, 2H) ppm; ^13^C NMR (101 MHz, CDCl_3_) δ 171.8, 170.1, 170.0, 169.9, 169.0, 158.6, 148.8, 146.7, 144.5, 141.8, 139.1, 135.9, 134.0, 133.2, 133.0, 132.5, 130.1, 130.0, 129.7, 128.9, 128.4, 127.7, 127.0, 126.0, 123.9, 121.4, 120.6, 120.3, 114.9, 114.4, 69.7, 67.5, 67.3, 51.9, 50.4, 46.6, 39.7, 37.2, 31.5, 30.1, 28.8, 28.4, 26.3, 26.1, 23.2 ppm; HRMS (ESI) *m*/*z* calcd for C_45_H_49_N_8_O_7_ [M + H]^+^ 813.37242, found 813.37079.

##### 3′-(2-(2-(3-((2-(2,6-Dioxopiperidin-3-yl)-1-oxoisoindolin-4-yl)­amino)-3-oxopropoxy)­ethoxy)­ethoxy)-*N*-(7-(4-(pyridin-3-yl)-1*H*-1,2,3-triazol-1-yl)­heptyl)-[1,1′-biphenyl]-2-carboxamide (**U27**)

The compound was prepared from carboxylic acid **76**. White solid; yield 40%; IR (neat) 3270, 3052, 2920, 1683, 1636, 1537, 1431, 1200, 1104, 1053, 752 cm^–1^; ^1^H NMR (400 MHz, CD_3_OD) δ 9.03 (br s, 1H), 8.52–8.50 (m, 2H), 8.28 (d, *J* = 8.0 Hz, 1H), 7.72 (d, *J* = 7.9 Hz, 1H), 7.57–7.36 (m, 7H), 7.21 (t, *J* = 8.9 Hz, 1H), 7.00–6.95 (m, 1H), 6.90 (s, 1H), 6.79 (dd, *J* = 8.2/2.2 Hz, 1H), 5.13 (dd, *J* = 13.3/5.2 Hz, 1H), 4.49–4.44 (m, 4H), 3.12 (t, *J* = 6.7 Hz, 2H), 2.92–2.83 (m, 1H), 2.77–2.72 (m, 1H), 2.67 (t, *J* = 5.9 Hz, 2H), 2.42 (qd, *J* = 13.2/4.6 Hz, 1H), 2.16–2.12 (m, 1H), 1.92 (quint, *J* = 6.8 Hz, 2H), 1.30–1.24 (m, 6H), 1.03–1.02 (m, 2H) ppm; ^13^C NMR (101 MHz, CD_3_OD) δ 173.2, 171.5, 171.0, 170.6, 169.6, 158.6, 148.1, 145.8, 143.9, 141.7, 139.4, 136.5, 134.8, 135.5, 133.1, 132.4, 129.8, 129.4, 129.0, 128.6, 127.4, 127.3, 127.0, 126.2, 124.2, 121.7, 121.0, 120.0, 114.5, 113.4, 70.2, 70.0, 69.4, 67.2, 66.7, 52.2, 50.2, 47.0, 39.3, 36.6, 31.0, 29.8, 28.4, 28.3, 26.2, 25.9, 22.8 ppm; HRMS (ESI) *m*/*z* calcd for C_47_H_53_N_8_O_7_ [M + H]^+^ 857.39864, found 857.39694.

##### 3′-(2-(2-(2-(3-((2-(2,6-Dioxopiperidin-3-yl)-1-oxoisoindolin-4-yl)­amino)-3-oxopropoxy)­ethoxy)­ethoxy)­ethoxy)-*N*-(7-(4-(pyridin-3-yl)-1*H*-1,2,3-triazol-1-yl)­heptyl)-[1,1′-biphenyl]-2-carboxamide (**U28**)

The compound was prepared from carboxylic acid **77**. White solid; yield 9%; IR (neat) 3271, 3067, 2921, 2852, 1685, 1601, 1535, 1457, 1200, 1085, 1053, 750 cm^–1^; ^1^H NMR (400 MHz, CD_3_OD) δ 9.04 (br s, 1H), 8.52 (br s, 2H), 8.29 (d, *J* = 8.0 Hz, 1H), 7.76–7.72 (m, 2H), 7.63 (d, *J* = 7.5 Hz, 1H), 7.56–7.44 (m, 5H), 7.30–7.26 (m, 2H), 6.91–6.84 (m, 2H), 5.16 (dd, *J* = 13.5/5.1 Hz, 1H), 4.50–4.46 (m, 4H), 4.03–4.01 (m, 2H), 3.82 (t, *J* = 5.9 Hz, 2H), 3.73–3.71 (m, 2H), 3.64–3.61 (m, 8H), 3.15 (br s, 2H), 2.95–2.86 (m, 1H), 2.81–2.76 (m, 1H), 2.66 (t, *J* = 5.9 Hz, 2H), 2.45 (qd, *J* = 13.3/4.8 Hz, 1H), 2.20–2.16 (m, 1H), 1.95 (quint, *J* = 6.9 Hz, 2H), 1.33–1.30 (m, 6H), 1.14–1.12 (m, 2H) ppm; ^13^C NMR (101 MHz, CD_3_OD) δ 173.2, 171.1, 170.6, 170.3, 169.6, 157.3, 148.1, 145.8, 143.9, 140.0, 137.3, 136.7, 134.9, 133.5, 133.2, 132.5, 130.5, 129.8, 129.4, 128.7, 127.7, 127.4, 127.1, 126.3, 124.3, 121.7, 120.0 (2C), 117.2, 115.2, 70.3, 70.1, 70.0, 69.9, 69.3, 67.6, 66.7, 52.2, 50.2, 47.0, 39.1, 36.6, 31.0, 29.8, 28.7, 28.3, 26.2, 25.9, 22.8 ppm; HRMS (ESI) *m*/*z* calcd for C_49_H_57_N_8_O_9_ [M + H]^+^ 901.42485, found 901.42354.

##### N-(2-(2,6-Dioxopiperidin-3-yl)-1-oxoisoindolin-4-yl)-1-((2′-((7-(4-(pyridin-3-yl)-1*H*-1,2,3-triazol-1-yl)­heptyl)­carbamoyl)-[1,1′-biphenyl]-3-yl)­oxy)-3,6,9,12-tetraoxapentadecan-15-amide (**U29**)

The compound was prepared from carboxylic acid **78**. White solid; yield 14%; IR (neat) 3292, 3065, 2921, 2852, 1686, 1638, 1539, 1432, 1201, 1095, 1026, 753 cm^–1^; ^1^H NMR (400 MHz, CD_3_OD) δ 9.04 (br s, 1H), 8.54–8.52 (m, 2H), 8.31–8.28 (m, 1H), 7.76 (d, *J* = 7.9 Hz, 1H), 7.64 (d, *J* = 7.5 Hz, 1H), 7.55–7.38 (m, 6H), 7.23 (t, *J* = 7.1 Hz, 1H), 7.00–6.97 (m, 2H), 6.88–6.85 (m, 1H), 5.16 (dd, *J* = 13.3/5.2 Hz, 1H), 4.54–4.44 (m, 4H), 4.11–4.08 (m, 2H), 3.85–3.78 (m, 4H), 3.65–3.54 (m, 12H), 3.15–3.12 (m, 2H), 2.95–2.86 (m, 1H), 2.81–2.75 (m, 1H), 2.66 (t, *J* = 5.9 Hz, 2H), 2.51–2.40 (qd, *J* = 13.2/4.8 Hz, 1H), 2.21–2.15 (m, 1H), 1.94 (quint, *J* = 7.0 Hz, 2H), 1.31–1.27 (m, 6H), 1.04–1.01 (m, 2H) ppm; ^13^C NMR (101 MHz, CD_3_OD) δ 173.2, 171.5, 171.4, 170.6, 169.6, 158.7, 148.1, 145.9, 143.9, 141.8, 139.5, 136.5, 134.9, 135.5, 133.2, 132.5, 129.7, 129.4, 129.0, 128.7, 127.4, 127.3, 127.0, 126.3, 124.2, 121.8, 121.0, 120.0, 114.6, 113.5, 70.3, 70.1 (3C), 70.0, 69.9, 69.4, 67.2, 66.7, 52.2, 50.2, 47.0, 39.3, 36.6, 31.0, 29.8, 28.4, 28.3, 26.2, 25.9, 22.8 ppm; HRMS (ESI) *m*/*z* calcd for C_51_H_61_N_8_O_10_ [M + H]^+^ 945.45107, found 945.44842.

### Synthesis of NAMPT Targeting PROTACs **U30–42**

Ethers **91–96** were synthesized from phenol **8** and the corresponding bromo-ester (**85–90**) with addition of NaI (0.5 equiv) following the general procedure A.

#### General Procedure E for the Basic Hydrolysis of Esters (**97–102**)

To a stirred solution of the corresponding methyl ester (**91–96**) (1 equiv) in THF/H_2_O 1:1 (0.05 M), was added LiOH (4 equiv) and the mixture was heated to 45 °C for 4 h. After completion of the reaction, water was added and the resulting aqueous phase was acidified with 2 M HCl until pH ∼ 3. The product was extracted with CH_2_Cl_2_ (×3). The combined organic layers were washed with brine (×1), dried over anhydrous Na_2_SO_4_ and concentrated in vacuo. The crude was purified by column chromatography using CH_2_Cl_2_/MeOH 98:2 and CH_2_Cl_2_/MeOH 9:1 as eluents.

Finally, the carboxylic acids (**97–102**) were coupled with VHL ligand **10** or lenalidomide (**9**) overnight following the general procedure D.

##### (2*S*,4*R*)-1-((*S*)-3,3-Dimethyl-2-(7-((2′-((7-(4-(pyridin-3-yl)-1*H*-1,2,3-triazol-1-yl)­heptyl)­carbamoyl)-[1,1′-biphenyl]-3-yl)­oxy)­heptanamido)­butanoyl)-4-hydroxy-*N*-(4-(4-methylthiazol-5-yl)­benzyl)­pyrrolidine-2-carboxamide (**U30**)

The compound was prepared from **97**. White solid; yield 63%; IR (neat) 3303, 3066, 2930, 2858, 1630, 1528, 1434, 1201, 847, 759 cm^–1^; ^1^H NMR (400 MHz, CDCl_3_) δ 9.03 (br s, 1H), 8.69 (s, 1H), 8.55 (br s, 1H), 8.31 (d, *J* = 7.8 Hz, 1H), 8.10 (s, 1H), 7.70 (d, *J* = 7.4 Hz, 1H), 7.49–7.28 (m, 10H), 6.98–6.86 (m, 3H), 6.30 (d, *J* = 8.6 Hz, 1H), 5.32 (br s, 1H), 4.72 (t, *J* = 7.9 Hz, 1H), 4.61–4.54 (m, 3H), 4.44–4.33 (m, 3H), 4.08 (br d, 1H), 3.92 (t, *J* = 6.1 Hz, 2H), 3.63–3.60 (m, 1H), 3.16–3.15 (m, 2H), 2.57–2.52 (m, 4H), 2.24–2.12 (m, 3H), 1.95 (quint, *J* = 6.7 Hz, 2H), 1.75 (quint, *J* = 6.6 Hz, 2H), 1.64 (quint, *J* = 7.2 Hz, 2H), 1.53–1.15 (m, 10H), 1.00–0.94 (m, 11H) ppm; ^13^C NMR (101 MHz, CDCl_3_) δ 173.4, 171.8, 170.9, 169.5, 159.1, 150.3, 148.7, 148.4, 146.7, 144.5, 141.7, 139.3, 138.2, 135.8, 133.2, 131.6, 130.9, 130.0 (2C), 129.7, 129.5, 128.7, 128.1, 127.7, 127.2, 124.0, 120.8, 120.3, 114.5, 114.3, 69.9, 67.8, 58.7, 57.3, 56.9, 50.5, 43.2, 39.7, 36.3, 36.1, 35.2, 30.2, 29.0 (2C), 28.9, 28.8, 28.6, 26.4, 26.2, 25.8, 25.5, 16.0 ppm; HRMS (ESI) *m*/*z* calcd. for C_56_H_70_N_9_O_6_S [M + H]^+^ 996.51698, found 996.51558.

##### (2*S*,4*R*)-1-((*S*)-3,3-Dimethyl-2-(8-((2′-((7-(4-(pyridin-3-yl)-1*H*-1,2,3-triazol-1-yl)­heptyl)­carbamoyl)-[1,1′-biphenyl]-3-yl)­oxy)­octanamido)­butanoyl)-4-hydroxy-*N*-(4-(4-methylthiazol-5-yl)­benzyl)­pyrrolidine-2-carboxamide (**U31**)

The compound was prepared from **98**. White solid; yield 53%; IR (neat) 3291, 3066, 2927, 2855, 1629, 1529, 1435, 1201, 759, 697 cm^–1^; ^1^H NMR (400 MHz, CDCl_3_) δ 9.00 (br s, 1H), 8.66 (s, 1H), 8.54 (br s, 1H), 8.24 (d, *J* = 7.9 Hz, 1H), 8.00 (s, 1H), 7.67 (d, *J* = 7.2 Hz, 1H), 7.47–7.26 (m, 10H), 6.96–6.84 (m, 3H), 6.36 (d, *J* = 8.8 Hz, 1H), 5.40 (t, *J* = 5.4 Hz, 1H), 4.72 (t, *J* = 7.9 Hz, 1H), 4.59–4.42 (m, 3H), 4.41–4.31 (m, 4H), 4.06–4.03 (m, 1H), 3.91 (t, *J* = 6.4 Hz, 2H), 3.65–3.61 (m, 1H), 3.14 (q, *J* = 6.3 Hz, 2H), 2.50–2.40 (m, 4H), 2.34–2.11 (m, 3H), 1.92 (quint, *J* = 6.9 Hz, 2H), 1.73 (quint, *J* = 6.8 Hz, 2H), 1.59 (quint, *J* = 6.9 Hz, 2H), 1.42–1.14 (m, 12H), 1.04–0.94 (m, 11H) ppm; ^13^C NMR (101 MHz, CDCl_3_) δ 173.5, 171.8, 171.0, 169.5, 159.1, 150.3, 148.9, 148.4, 146.8, 144.6, 141.7, 139.3, 138.2, 135.8, 133.1, 131.6, 130.9, 130.0 (2C), 129.7, 129.5, 128.8, 128.1, 127.7, 127.1, 124.0, 120.8, 120.2, 114.6, 114.2, 69.9, 67.9, 58.7, 57.3, 56.9, 50.4, 43.2, 39.7, 36.4, 36.1, 35.2, 30.2, 29.1, 29.0, 28.9, 28.8, 28.5, 26.4, 26.3, 26.2, 25.8, 25.5, 16.0 ppm; HRMS (ESI) *m*/*z* calcd. for C_57_H_72_N_9_O_6_S [M + H]^+^ 1010.53263, found 1010.53113.

##### (2*S*,4*R*)-1-((*S*)-3,3-Dimethyl-2-(9-((2′-((7-(4-(pyridin-3-yl)-1*H*-1,2,3-triazol-1-yl)­heptyl)­carbamoyl)-[1,1′-biphenyl]-3-yl)­oxy)­nonanamido)­butanoyl)-4-hydroxy-*N*-(4-(4-methylthiazol-5-yl)­benzyl)­pyrrolidine-2-carboxamide (**U32**)

The compound was prepared from **99**. White solid; yield 40%; IR (neat) 3288, 3067, 2927, 2855, 1629, 1527, 1436, 1201, 759, 698 cm^–1^; ^1^H NMR (400 MHz, CDCl_3_) δ 9.00 (br s, 1H), 8.67 (s, 1H), 8.53 (d, *J* = 1.3 Hz, 1H), 8.25 (dt, *J* = 8.0/1.9 Hz, 1H), 8.00 (s, 1H), 7.69 (dd, *J* = 7.5/1.3 Hz, 1H), 7.48–7.26 (m, 10H), 6.97–6.86 (m, 3H), 6.32 (d, *J* = 8.8 Hz, 1H), 5.37 (t, *J* = 5.6 Hz, 1H), 4.72 (t, *J* = 7.9 Hz, 1H), 4.59–4.53 (m, 3H), 4.41 (t, *J* = 7.2 Hz, 2H), 4.37–4.32 (m, 1H), 4.27 (br s, 1H), 4.06 (br d, 1H), 3.92 (t, *J* = 6.4 Hz, 2H), 3.64–3.61 (m, 1H), 3.14 (q, *J* = 6.2 Hz, 2H), 2.51–2.45 (m, 4H), 2.20–2.11 (m, 3H), 1.93 (quint, *J* = 7.0 Hz, 2H), 1.74 (quint, *J* = 6.8 Hz, 2H), 1.59 (quint, *J* = 6.2 Hz, 2H), 1.40 (quint, *J* = 7.7 Hz, 2H), 1.29–1.15 (m, 12H), 1.05–0.94 (m, 11H) ppm; ^13^C NMR (101 MHz, CDCl_3_) δ 173.6, 171.8, 170.9, 169.4, 159.2, 150.3, 148.9, 148.4, 146.8, 144.6, 141.7, 139.3, 138.1, 135.7, 133.1, 131.6, 130.9, 130.0 (2C), 129.7, 129.5, 128.8, 128.1, 127.7, 127.0, 123.9, 120.8, 120.1, 114.6, 114.2, 69.9, 68.0, 58.7, 57.3, 56.9, 50.5, 43.2, 39.7, 36.5, 36.0, 35.2, 30.2, 29.2 (3C), 29.1, 29.0, 28.8, 28.5, 26.4, 26.2, 26.0, 25.6, 16.0 ppm; HRMS (ESI) *m*/*z* calcd. for C_58_H_74_N_9_O_6_S [M + H]^+^ 1024.54828, found 1024.54812.

##### (2*S*,4*R*)-1-((*S*)-3,3-Dimethyl-2-(10-((2′-((7-(4-(pyridin-3-yl)-1*H*-1,2,3-triazol-1-yl)­heptyl)­carbamoyl)-[1,1′-biphenyl]-3-yl)­oxy)­decanamido)­butanoyl)-4-hydroxy-*N*-(4-(4-methylthiazol-5-yl)­benzyl)­pyrrolidine-2-carboxamide (**U33**)

The compound was prepared from **100**. White solid; yield 67%; IR (neat) 3290, 3065, 2925, 2854, 1628, 1528, 1434, 1201, 1028, 759, 697 cm^–1^; 1H NMR (400 MHz, CDCl_3_) δ 9.00 (br s, 1H), 8.67 (s, 1H), 8.54 (br s, 1H), 8.25 (d, *J* = 7.9 Hz, 1H), 7.98 (s, 1H), 7.69 (d, *J* = 7.4 Hz, 1H), 7.47–7.26 (m, 10H), 6.97–6.87 (m, 3H), 6.32 (d, *J* = 8.5 Hz, 1H), 5.37 (br s, 1H), 4.72 (t, *J* = 7.8 Hz, 1H), 4.58–4.53 (m, 3H), 4.41 (t, *J* = 7.1 Hz, 2H), 4.37–4.32 (m, 1H), 4.08–4.05 (m, 1H), 3.93 (t, *J* = 6.4 Hz, 2H), 3.65–3.61 (m, 1H), 3.14 (q, *J* = 6.4 Hz, 2H), 2.51 (br s, 4H), 2.20–2.14 (m, 3H), 1.93 (quint, *J* = 6.9 Hz, 2H), 1.75 (quint, *J* = 7.0 Hz, 2H), 1.58–1.51 (m, 2H), 1.44–1.15 (m, 16H), 1.04–0.94 (m, 11H) ppm; ^13^C NMR (101 MHz, CDCl_3_) δ 173.7, 171.8, 170.9, 169.4, 159.2, 150.3, 148.7, 148.4, 146.6, 144.5, 141.7, 139.3, 138.1, 135.7, 133.3, 131.6, 130.9, 130.1, 130.0, 129.7, 129.5, 128.8, 128.1, 127.7, 127.1, 124.0, 120.9, 120.2, 114.6, 114.2, 69.9, 68.0, 58.7, 57.3, 56.9, 50.5, 43.2, 39.7, 36.5, 36.0, 35.1, 30.2, 29.7, 29.3, 29.2, 29.2, 29.2, 29.2, 28.8, 28.5, 26.4, 26.3, 26.0, 25.6, 16.0 ppm; HRMS (ESI) *m*/*z* calcd. for C_59_H_76_N_9_O_6_S [M + H]^+^ 1038.56393, found 1038.56175.

##### (2*S*,4*R*)-1-((*S*)-3,3-Dimethyl-2-(11-((2′-((7-(4-(pyridin-3-yl)-1*H*-1,2,3-triazol-1-yl)­heptyl)­carbamoyl)-[1,1′-biphenyl]-3-yl)­oxy)­undecanamido)­butanoyl)-4-hydroxy-*N*-(4-(4-methylthiazol-5-yl)­benzyl)­pyrrolidine-2-carboxamide (**U34**)

The compound was prepared from **101**. White solid; yield 51%; IR (neat) 3294, 3066, 2922, 2852, 1628, 1528, 1434, 1201, 844, 759, 698 cm^–1^; ^1^H NMR (400 MHz, CDCl_3_) δ 9.01 (br s, 1H), 8.68 (s, 1H), 8.57 (d, *J* = 4 Hz, 1H), 8.25 (d, *J* = 7.8 Hz, 1H), 7.96 (s, 1H), 7.72 (d, *J* = 7.4 Hz, 1H), 7.49–7.28 (m, 10H), 6.98–6.89 (m, 3H), 6.19 (d, *J* = 8.6 Hz, 1H), 5.30 (br s, 1H), 4.73 (t, *J* = 7.9 Hz, 1H), 4.61–4.53 (m, 3H), 4.43 (t, *J* = 7.1 Hz, 2H), 4.38–4.33 (m, 1H), 4.13–4.11 (m, 1H), 3.95 (t, *J* = 6.4 Hz, 2H), 3.63–3.60 (m, 1H), 3.18–3.13 (q, *J* = 6.4 Hz, 2H), 2.60–2.53 (m, 4H), 2.21–2.12 (m, 3H), 1.97–1.92 (quint, *J* = 6.9 Hz, 2H), 1.77 (quint, *J* = 7.1 Hz, 2H), 1.59 (br s, 2H), 1.50–1.44 (m, 4H), 1.28–1.19 (m, 14H), 1.06–0.95 (m, 11H) ppm; ^13^C NMR (101 MHz, CDCl_3_) δ 173.8, 171.9, 170.8, 169.4, 159.2, 150.3, 149.0, 148.5, 146.8, 144.6, 141.7, 139.3, 138.1, 135.7, 133.1, 131.6, 131.0, 130.0 (2C), 129.7, 129.5, 128.8, 128.1, 127.7, 127.0, 123.9, 120.8, 120.1, 114.6, 114.2, 70.0, 68.0, 58.5, 57.4, 56.8, 50.5, 43.3, 39.7, 36.5, 35.8, 35.0, 30.2, 29.4, 29.3 (2C), 29.2 (3C), 29.1, 28.8, 28.5, 26.4, 26.2, 26.0, 25.6, 16.0 ppm; HRMS (ESI) *m*/*z* calcd. for C_60_H_78_N_9_O_6_S [M + H]^+^ 1052.57958, found 1052.57738.

##### (2*S*,4*R*)-1-((*S*)-3,3-Dimethyl-2-(12-((2′-((7-(4-(pyridin-3-yl)-1*H*-1,2,3-triazol-1-yl)­heptyl)­carbamoyl)-[1,1′-biphenyl]-3-yl)­oxy)­dodecanamido)­butanoyl)-4-hydroxy-*N*-(4-(4-methylthiazol-5-yl)­benzyl)­pyrrolidine-2-carboxamide (**U35**)

The compound was prepared from **102**. White solid; yield 35%; IR (neat) 3285, 3069, 2922, 2853, 1630, 1528, 1435, 1201, 759, 697 cm^–1^; ^1^H NMR (400 MHz, CDCl_3_) δ 9.01 (br s, 1H), 8.68 (s, 1H), 8.57 (br s, 1H), 8.25 (d, *J* = 7.9 Hz, 1H), 7.96 (s, 1H), 7.71 (d, *J* = 7.5 Hz, 1H), 7.49–7.28 (m, 10H), 6.98–6.88 (m, 3H), 6.20 (br d, 1H), 5.31 (br s, 1H), 4.74 (t, *J* = 7.8 Hz, 1H), 4.61–4.54 (m, 3H), 4.42 (t, *J* = 7.1 Hz, 2H), 4.38–4.33 (m, 1H), 4.11–4.08 (br d, 1H), 3.95 (t, *J* = 6.5 Hz, 2H), 3.64–3.60 (m, 1H), 3.15 (q, *J* = 6.4 Hz, 2H), 2.57–2.52 (m, 4H), 2.21–2.15 (m, 3H), 1.94 (quint, *J* = 7.0 Hz, 4H), 1.77 (quint, *J* = 7.0 Hz, 2H), 1.59 (br s, 2H), 1.44 (quint, *J* = 6.8 Hz, 2H), 1.26–1.18 (m, 16H), 1.06–0.94 (m, 11H) ppm; ^13^C NMR (101 MHz, CDCl_3_) δ 173.7, 172.0, 170.7, 169.4, 159.2, 150.3, 149.0, 148.5, 146.9, 144.6, 141.7, 139.3, 138.1, 135.7, 133.1, 131.6, 131.0, 130.1, 130.0, 129.7, 129.5, 128.9, 128.1, 127.7, 127.0, 123.9, 120.8, 120.0, 114.6, 114.2, 70.0, 68.0, 58.5, 57.4, 56.8, 50.5, 43.3, 39.7, 36.5, 35.8, 35.0, 30.2, 29.5, 29.4, 29.4, 29.3, 29.2 (3C), 29.1, 28.9, 28.5, 26.4, 26.3, 26.0, 25.5, 16.1 ppm; HRMS (ESI) *m*/*z* calcd. for C_61_H_80_N_9_O_6_S [M + H]^+^ 1066.59523, found 1066.59612.

##### 3′-((7-((2-(2,6-Dioxopiperidin-3-yl)-1-oxoisoindolin-4-yl)­amino)-7-oxoheptyl)­oxy)-*N*-(7-(4-(pyridin-3-yl)-1*H*-1,2,3-triazol-1-yl)­heptyl)-[1,1′-biphenyl]-2-carboxamide (**U36**)

The compound was prepared from **97**. White solid; yield 49%; IR (neat) 3269, 3068, 2928, 2856, 1686, 1638, 1529, 1429, 1200, 752, 726, 698 cm^–1^; ^1^H NMR (400 MHz, CDCl_3_) δ 9.23 (s, 1H), 8.99 (br s, 2H), 8.55 (br s, 1H), 8.19 (d, *J* = 7.6 Hz, 1H), 7.95 (s, 1H), 7.71–7.63 (m, 3H), 7.47–7.52 (m, 6H), 6.96–6.83 (m, 3H), 5.53 (br s, 1H), 5.07 (dd, *J* = 12.9/4.8 Hz, 1H), 4.41–4.33 (m, 4H), 3.90 (t, *J* = 5.7 Hz, 2H), 3.14 (q, *J* = 6.0 Hz, 2H), 2.78–2.66 (m, 2H), 2.42 (t, *J* = 7.0 Hz, 2H), 2.19–2.09 (m, 2H), 1.91–1.89 (m, 2H), 1.74 (br s, 4H), 1.43 (br s, 4H), 1.23–1.19 (m, 6H), 1.00 (br d, 2H) ppm; ^13^C NMR (101 MHz, CDCl_3_) δ 172.2, 175.6, 170.0, 169.8, 169.1, 159.1, 148.8, 146.6, 144.5, 141.6, 139.3, 135.8, 134.1, 133.3, 133.3, 132.6, 130.1 (2C), 129.7, 128.9, 128.5, 127.7, 126.9, 126.2, 124.0, 120.7, 120.5, 120.3, 114.9, 114.1, 67.9, 51.8, 50.5, 46.8, 39.7, 36.6, 31.5, 30.1, 28.8 (2C), 28.5, 26.3, 26.1, 25.6, 25.4, 23.2 ppm; HRMS (ESI) *m*/*z* calcd. for C_47_H_53_N_8_O_6_ [M + H]^+^ 825.40881, found 825.40767.

##### 3′-((8-((2-(2,6-Dioxopiperidin-3-yl)-1-oxoisoindolin-4-yl)­amino)-8-oxooctyl)­oxy)-*N*-(7-(4-(pyridin-3-yl)-1*H*-1,2,3-triazol-1-yl)­heptyl)-[1,1′-biphenyl]-2-carboxamide (**U37**)

The compound was prepared from **98**. White solid; yield 39%; IR (neat) 3256, 3066, 2923, 2853, 1682, 1639, 1601, 1534, 1430, 1201, 1051, 793, 751, 698 cm^–1^; ^1^H NMR (400 MHz, CDCl_3_) δ 9.01 (s, 2H), 8.90 (s, 1H), 8.57 (br s, 1H), 8.21 (d, *J* = 7.9 Hz, 1H), 7.93 (s, 1H), 7.68–7.63 (m, 3H), 7.49–7.27 (m, 6H), 6.96 (d, *J* = 7.7 Hz, 1H), 6.89–6.86 (m, 2H), 5.43 (t, *J* = 5.7 Hz, 1H), 5.13 (dd, *J* = 13.1/5.2 Hz, 1H), 4.46–4.36 (m, 4H), 3.92 (t, *J* = 6.4 Hz, 2H), 3.15 (q, *J* = 6.2 Hz, 2H), 2.83–2.69 (m, 2H), 2.43 (t, *J* = 7.3 Hz, 2H), 2.29–2.18 (m, 1H), 2.14–2.11 (m, 1H), 1.92 (quint, *J* = 7.0 Hz, 2H), 1.74 (t, *J* = 6.6 Hz, 2H), 1.46–1.38 (m, 6H), 1.27–1.15 (m, 6H), 1.00 (quint, *J* = 7.0 Hz, 2H) ppm; ^13^C NMR (101 MHz, CDCl_3_) δ 172.1, 171.4, 169.8, 169.7, 169.1, 159.1, 148.9, 146.6, 144.6, 141.6, 139.3, 135.7, 134.4, 133.3, 133.2, 132.7, 130.1, 130.0, 129.8, 128.9, 128.6, 127.7, 127.0, 126.2, 124.0, 120.7, 120.6, 120.2, 114.7, 114.3, 68.0, 51.8, 50.5, 46.8, 39.8, 36.6, 31.5, 30.2, 28.9, 28.8, 28.8, 28.7, 28.5, 26.4, 26.2, 25.9, 25.4, 23.3 ppm; HRMS (ESI) *m*/*z* calcd. for C_48_H_55_N_8_O_6_ [M + H]^+^ 839.42446, found 839.42275.

##### 3′-((9-((2-(2,6-Dioxopiperidin-3-yl)-1-oxoisoindolin-4-yl)­amino)-9-oxononyl)­oxy)-*N*-(7-(4-(pyridin-3-yl)-1*H*-1,2,3-triazol-1-yl)­heptyl)-[1,1′-biphenyl]-2-carboxamide (**U38**)

The compound was prepared from **99**. Off-white solid; yield 53%; IR (neat) 3270, 3065, 2925, 2852, 1677, 1638, 1534, 1430, 1200, 1051, 751, 698 cm^–1^; ^1^H NMR (400 MHz, CDCl_3_) δ 9.01 (br s, 1H), 8.89 (s, 1H), 8.71 (s, 1H), 8.57 (br s, 1H), 8.22 (d, *J* = 7.7 Hz, 1H), 7.94 (s, 1H), 7.68–7.65 (m, 3H), 7.49–7.28 (m, 6H), 6.97 (d, *J* = 7.3 Hz, 1H), 6.89–6.87 (m, 2H), 5.40 (br t, 1H), 5.14 (dd, *J* = 13.1/4.9 Hz, 1H), 4.45–4.40 (m, 4H), 3.93 (t, *J* = 6.2 Hz, 2H), 3.15 (q, *J* = 6.3 Hz, 2H), 2.85–2.70 (m, 2H), 2.42 (t, *J* = 7.1 Hz, 2H), 2.28–2.16 (m, 2H), 1.93 (quint, *J* = 6.6 Hz, 2H), 1.79–1.74 (m, 4H), 1.43–1.17 (m, 14H), 1.02–1.00 (m, 2H) ppm; ^13^C NMR (101 MHz, CDCl_3_) δ 172.3, 171.7, 170.1, 169.7, 169.1, 159.1, 148.9, 146.7, 144.5, 141.6, 139.3, 135.7, 134.2, 133.4, 133.2, 132.6, 130.1 (2C), 129.7, 128.9, 128.6, 127.7, 126.9, 126.2, 124.0, 120.7, 120.5, 120.3, 114.8, 114.1, 68.0, 51.8, 50.5, 46.8, 39.7, 36.8, 31.5, 30.2, 29.0 (2C), 29.0 (2C), 28.8, 28.5, 26.4, 26.2, 25.8, 25.6, 23.2 ppm; HRMS (ESI) *m*/*z* calcd. for C_49_H_57_N_8_O_6_ [M + H]^+^ 853.44011, found 853.43877.

##### 3′-((10-((2-(2,6-Dioxopiperidin-3-yl)-1-oxoisoindolin-4-yl)­amino)-10-oxodecyl)­oxy)-*N*-(7-(4-(pyridin-3-yl)-1*H*-1,2,3-triazol-1-yl)­heptyl)-[1,1′-biphenyl]-2-carboxamide (**U39**)

The compound was prepared from **100**. Off-white solid; yield 35%; IR (neat) 3260, 3065, 2926, 2854, 1686, 1639, 1601, 1534, 1430, 1201, 1050, 793, 752, 698 cm^–1^; ^1^H NMR (400 MHz, CDCl_3_) δ 9.19 (br s, 1H), 9.00 (s, 1H), 8.80 (br s, 1H), 8.55 (d, *J* = 3.9 Hz, 1H), 8.20 (dt, *J* = 6.1/1.8 Hz, 1H), 7.94 (s, 1H), 7.69–7.63 (m, 3H), 7.48–7.27 (m, 6H), 6.95 (d, *J* = 7.7 Hz, 1H), 6.89–6.86 (m, 2H), 5.47 (t, *J* = 5.6 Hz, 1H), 5.09 (dd, *J* = 13.1/5.2 Hz, 1H), 4.42–4.37 (m, 4H), 3.92 (t, *J* = 6.4 Hz, 2H), 3.14 (q, *J* = 6.2 Hz, 2H), 2.80–2.70 (m, 2H), 2.40 (t, *J* = 7.4 Hz, 2H), 2.24–2.08 (m, 2H), 1.92 (quint, *J* = 7.0 Hz, 2H), 1.77–1.68 (m, 4H), 1.41–1.15 (m, 16H), 1.01 (quint, *J* = 6.8 Hz, 2H) ppm; ^13^C NMR (101 MHz, CDCl_3_) δ 172.3, 171.6, 170.0, 169.6, 169.1, 159.2, 148.8, 146.6, 144.5, 141.6, 139.3, 135.6, 134.2, 133.3, 133.2, 132.6, 130.1 (2C), 129.7, 128.9, 128.7, 127.7, 127.0, 126.2, 124.0, 120.8, 120.6, 120.2, 114.8, 114.1, 68.0, 51.8, 50.5, 46.8, 39.7, 38.8, 31.5, 30.2, 29.2, 29.1, 29.0, 29.0, 28.9, 28.8, 28.5, 26.4, 26.2, 25.9, 25.6, 23.2 ppm; HRMS (ESI) *m*/*z* calcd. for C_50_H_59_N_8_O_6_ [M + H]^+^ 867.45576, found 867.45320.

##### 3′-((11-((2-(2,6-Dioxopiperidin-3-yl)-1-oxoisoindolin-4-yl)­amino)-11-oxoundecyl)­oxy)-*N*-(7-(4-(pyridin-3-yl)-1*H*-1,2,3-triazol-1-yl)­heptyl)-[1,1′-biphenyl]-2-carboxamide (**U40**)

The compound was prepared from **101**. Off-white solid; yield 28%; IR (neat) 3269, 3058, 2923, 2852, 1686, 1639, 1535, 1453, 1430, 1201, 1050, 751, 698 cm^–1^; ^1^H NMR (400 MHz, CDCl_3_) δ 9.07 (s, 1H), 9.00 (s, 1H), 8.64 (s, 1H), 8.56 (br s, 1H), 8.19 (d, *J* = 7.9 Hz, 1H), 7.91 (s, 1H), 7.69–7.65 (m, 3H), 7.48–7.27 (m, 6H), 6.97–6.87 (m, 3H), 5.42 (t, *J* = 5.5 Hz, 1H), 5.10 (dd, *J* = 13.0/4.9 Hz, 1H), 4.42–4.38 (m, 4H), 3.93 (t, *J* = 6.3 Hz, 2H), 3.15 (q, *J* = 6.5 Hz, 2H), 2.80–2.66 (m, 2H), 2.40 (t, *J* = 7.3 Hz, 2H), 2.25–2.20 (m, 1H), 2.18–2.09 (m, 1H), 1.90 (quint, *J* = 7.1 Hz, 2H), 1.76–1.69 (m, 4H), 1.42–1.18 (m, 18H), 1.04–1.02 (m, 2H) ppm; ^13^C NMR (101 MHz, CDCl_3_) δ 172.2, 171.5, 169.9, 169.5, 169.1, 159.2, 149.0, 146.8, 144.6, 141.7, 139.3, 135.6, 134.2, 133.2, 133.1, 132.6, 130.1 (2C), 129.7, 128.9, 128.7, 127.7, 126.9, 126.2, 123.9, 120.8, 120.7, 120.1, 114.8, 114.2, 68.0, 51.8, 50.5, 46.8, 39.7, 36.8, 31.5, 30.1, 29.7, 29.3, 29.2, 29.1, 29.1 (2C), 28.8, 28.5, 26.4, 26.2, 26.0, 25.6, 23.2 ppm; HRMS (ESI) *m*/*z* calcd. for C_51_H_61_N_8_O_6_ [M + H]^+^ 881.47141, found 861.46948.

##### 3′-((12-((2-(2,6-Dioxopiperidin-3-yl)-1-oxoisoindolin-4-yl)­amino)-12-oxododecyl)­oxy)-*N*-(7-(4-(pyridin-3-yl)-1*H*-1,2,3-triazol-1-yl)­heptyl)-[1,1′-biphenyl]-2-carboxamide (**U41**)

The compound was prepared from **102**. Off-white solid; yield 35%; IR (neat) 3269, 3066, 2923, 2852, 1684, 1639, 1602, 1533, 1430, 1297, 1200, 1051, 751, 698 cm^–1^; ^1^H NMR (400 MHz, CDCl_3_) δ 9.02 (s, 1H), 8.96 (s, 1H), 8.57 (br s, 1H), 8.52 (s, 1H), 8.22 (d, *J* = 7.5 Hz, 1H), 7.92 (s, 1H), 7.70–7.67 (m, 3H), 7.49–7.28 (m, 6H), 6.97–6.88 (m, 3H), 5.39 (br t, 1H), 5.13 (dd, *J* = 13.0/5.0 Hz, 1H), 4.43–4.40 (m, 4H), 3.94 (t, *J* = 6.2 Hz, 2H), 3.15 (q, *J* = 6.3 Hz, 2H), 2.84–2.70 (m, 2H), 2.41 (t, *J* = 7.3 Hz, 2H), 2.30–2.15 (m, 2H), 1.93 (quint, *J* = 6.6 Hz, 2H), 1.77–1.70 (m, 4H), 1.43–1.16 (m, 20H), 1.04 (quint, *J* = 6.7 Hz, 2H) ppm; ^13^C NMR (101 MHz, CDCl_3_) δ 172.1, 171.4, 169.9, 169.5, 169.0, 159.2, 148.9, 146.7, 144.6, 141.6, 139.3, 135.6, 134.3, 133.2, 133.1, 132.7, 130.1 (3C), 129.7, 129.0, 128.8, 127.7, 127.0, 126.2, 124.0, 120.8, 120.1, 114.8, 114.1, 68.0, 51.8, 50.5, 46.7, 39.7, 36.9, 31.5, 30.2, 29.3 (3C), 29.2 (4C), 28.8, 28.5, 26.4, 26.2, 26.0, 25.6, 23.3 ppm; HRMS (ESI) *m*/*z* calcd. for C_52_H_63_N_8_O_6_ [M + H]^+^ 895.48706, found 895.48523.

##### (2*S*,4*R*)-1-((*S*)-3,3-Dimethyl-2-(8-((2′-((7-(4-(pyridin-3-yl)-1*H*-1,2,3-triazol-1-yl)­heptyl)­carbamoyl)-[1,1′-biphenyl]-3-yl)­oxy)­octanamido)­butanoyl)-4-hydroxy-*N*-((*S*)-1-(4-(4-methylthiazol-5-yl)­phenyl)­ethyl)­pyrrolidine-2-carboxamide (**U42**)

The compound was prepared from VHL ligand **103** and carboxylic acid **98** following the general procedure D. White solid, yield 84%; IR (neat) 3292, 3065, 2929, 2858, 1627, 1528, 1436, 1300, 1201, 759, 698 cm^–1^; ^1^H NMR (400 MHz, CDCl_3_) δ 8.99 (s, 1H), 8.65 (s, 1H), 8.53 (d, *J* = 3.8 Hz, 1H), 8.24 (dt, *J* = 8.0, 1.9 Hz, 1H), 8.00 (s, 1H), 7.68 (dd, *J* = 7.5, 1.6 Hz, 1H), 7.46–7.25 (m, 11H), 6.95 (dt, *J* = 7.5, 1.2 Hz, 1H), 6.89 (t, *J* = 2.0 Hz, 1H), 6.85 (ddd, *J* = 8.3, 2.6, 1.0 Hz, 1H), 6.30 (d, *J* = 8.7 Hz, 1H), 5.30 (t, *J* = 6.0 Hz, 1H), 5.07 (quint, *J* = 7.1 Hz, 1H), 4.72 (t, *J* = 7.9 Hz, 1H), 4.59 (d, *J* = 8.8 Hz, 1H), 4.51 (brs, 1H), 4.40 (t, *J* = 7.2 Hz, 2H), 4.06 (dt, *J* = 11.5, 1.9 Hz, 1H), 3.91 (t, *J* = 6.4 Hz, 2H), 3.59 (dd, *J* = 11.3, 3.8 Hz, 1H), 3.13 (q, *J* = 6.7 Hz, 2H), 2.54–2.48 (m, 4H), 2.21–2.17 (m, 2H), 2.09–2.03 (m, 1H), 1.92 (quint, *J* = 7.2 Hz, 2H), 1.73 (quint, *J* = 6.6 Hz, 2H), 1.61 (q, *J* = 7.3 Hz, 2H), 1.46 (d, *J* = 6.9 Hz, 3H), 1.43–1.38 (m, 2H), 1.34–1.21 (m, 8H), 1.17 (quint, *J* = 7.3 Hz, 2H), 1.03–0.97 (m, 11H) ppm; ^13^C NMR (101 MHz, CDCl_3_) δ 173.6, 172.2, 169.8, 169.5, 159.3, 150.4, 149.1, 148.6, 147.0, 144.7, 143.3, 141.8, 139.4, 135.9, 133.2, 131.7, 131.0, 130.2, 130.1, 129.8, 129.7, 128.9, 127.8, 127.2, 126.6, 124.0, 121.0, 120.3, 114.7, 114.3, 70.0, 68.0, 58.6, 57.6, 56.9, 50.6, 49.0, 39.8, 36.5, 35.6, 35.3, 30.4, 29.2, 29.2, 29.1, 29.0, 28.7, 26.6, 26.5, 26.4, 26.0, 25.6, 22.3, 16.2 ppm; HRMS (ESI) *m*/*z* calcd. for C_58_H_74_N_9_O_6_S [M + H]^+^ 1024.54828, found 1024.54811.

##### (2*S*,4*S*)-1-((*S*)-3,3-Dimethyl-2-(8-((2′-((7-(4-(pyridin-3-yl)-1*H*-1,2,3-triazol-1-yl)­heptyl)­carbamoyl)-[1,1′-biphenyl]-3-yl)­oxy)­octanamido)­butanoyl)-4-hydroxy-*N*-((*S*)-1-(4-(4-methylthiazol-5-yl)­phenyl)­ethyl)­pyrrolidine-2-carboxamide (**U42-NC**)

The compound was prepared from the commercially available (2*S*,4*S*)-1-((*S*)-2-amino-3,3-dimethylbutanoyl)-4-hydroxy-*N*-((*S*)-1-(4-(4-methylthiazol-5-yl)­phenyl)­ethyl)­pyrrolidine-2-carboxamide (**104**), and carboxylic acid **98** following the general procedure D. White solid, yield 56%; ^1^H NMR (400 MHz, CDCl_3_) δ 9.02 (s, 1H), 8.69 (s, 1H), 8.57 (s, 1H), 8.22 (d, *J* = 6.4 Hz, 1H), 7.90 (s, 1H), 7.71–7.65 (m, 2H), 7.48–7.27 (m, 10H), 6.98–6.87 (m, 3H), 6.19 (d, *J* = 8.8 Hz, 1H), 5.31 (brt, 1H), 5.09 (quint, *J* = 7.1 Hz, 1H), 4.75 (d, *J* = 8.8 Hz, 1H), 4.59 (d, *J* = 8.9 Hz, 1H), 4.47–4.41 (m, 3H), 3.95–3.82 (m, 4H), 3.15 (q, *J* = 6.3 Hz, 2H), 2.54 (s, 3H), 2.36–2.15 (m, 4H), 1.93 (quint, *J* = 6.9 Hz, 2H), 1.76 (quint, *J* = 6.4 Hz, 2H), 1.63 (brs, 2H), 1.52–1.18 (m, 15H), 1.07–1.04 (m, 11H) ppm; ^13^C NMR (101 MHz, CDCl_3_) δ 173.1, 172.5, 171.5, 169.4, 159.2, 150.4, 149.1, 148.6, 147.0, 144.7, 142.3, 141.7, 139.2, 135.8, 133.0, 131.4, 131.2, 130.1, 130.0, 129.7 (2C), 128.8, 127.7, 126.9, 126.5, 123.8, 120.9, 119.9, 114.6, 114.2, 71.0, 68.0, 59.9, 58.6, 57.0, 50.4, 49.3, 39.6, 36.4, 35.1, 34.7, 30.2, 29.2 (2C), 29.0, 28.9, 28.5, 26.4, 26.3, 26.2, 25.9, 25.4, 21.9, 16.1 ppm; HRMS (ESI) *m*/*z* calcd. for C_58_H_74_N_9_O_6_S [M + H]^+^ 1024.54828, found 1024.54576.

### Cell Culturing

MCF7 human breast cancer cell line (ATCC HTB-22) and 4T1cl5 murine triple-negative breast cancer cell line^30^ were cultured in Minimum Essential Medium Eagle (MEM; Sigma-Aldrich) supplemented with 10% fetal bovine serum (FBS; Gibco), 1% glutamine (Sigma-Aldrich) and 1% penicillin/streptomycin (Sigma-Aldrich). All cells were maintained at 37 °C + 5% CO_2_.

### Western Blot Analysis

0.4 × 10^6^ or 1.5 × 10^5^ MCF7 and 4T1cl5 cells were seeded respectively into a 6 or 12-well plate to be exposed, the day after plating, to different concentrations of the synthesized compounds, as detailed in the text. For experiments shown in Figure 5A,B, bortezomib (300 nM, Sigma-Aldrich; #5043140001) was added 30 min before the treatment with PROTAC compounds. For experiments shown in Figure 6B,C, cells were treated with the lead compound **7** and ligand **VHL-Ac**. One hour later **U42** was added.

Cells were lysed in RIPA buffer containing protease inhibitors (100 nM PMSF, Sigma-Aldrich; PIC, Merck-Millipore) and phosphatase inhibitors (1 M NaF; 1 M Na_3_VO_4_, Sigma-Aldrich). Lysates were centrifuged at 13,000 rpm for 15 min at 4 °C, and protein concentration was measured using the Bradford assay (Sigma-Aldrich). Proteins were resolved via SDS-PAGE and transferred to nitrocellulose membranes using the TurboBlot system (Bio-Rad, Hemel Hempstead, UK). Immunoblotting was performed with primary antibodies mouse anti-NAMPT mAb (AdipoGen; Cat# AG-20A-0034, RRID:AB_2490117), anti-VHL mAb (Santa Cruz; Cat# sc-135657), NAPRT (Proteintech; Cat# 13549-1-AP) and mouse anti-Actin mAb (Sigma-Aldrich; #MAB1501), followed by HRP-conjugated antimouse secondary antibodies (Bio-Rad Laboratories). Protein levels were assessed using chemiluminescence detection with ECL solution (Thermo Fisher Scientific). The representative images and densitometry histograms were obtained by at least three independent experiments.

### MTT Assay

1.5 × 10^4^ MCF7 or 1.0 × 10^4^ 4T1cl5 were seeded into 24-multiwell. The day after plating, cells were treated with compounds for 72 h. After the exposure to synthesize compounds, cells were washed with 1X PBS and incubated with 250 μg/mL of 3-(4,5-dimethylthiazol-2-yl)-2,5-diphenyltetrazolium bromide (MTT; Sigma-Aldrich) in Locke buffer for 1 h at 37 °C + 5% CO_2_. Isopropanol-HCl 0.1 M was used to solubilize formazan crystals, and the optic absorbance was assessed at 570 nm using a plate reader (Victor3 V, PerkinElmer Life Sciences). The represented data were obtained by at least three independent experiments.

### Mammosphere Cultures in Poly-HEMA

A total of 2 × 10^3^ cells were seeded into 6-well plates precoated with 1.2% w/v poly­(2-hydroxyethyl methacrylate) (poly-HEMA, Sigma-Aldrich) in 95% ethanol heated at 60 °C and prepared the day before on 6-multiwell to let it dry overnight. Cells were maintained for 8 days in MEM without FBS which has been completed with B27 supplement (InvitrogenTM Life technology, CA, USA), basic fibroblast growth factor-2 (bFGF-2, 10 ng/mL, PeproTech, Rocky Hill, NJ, USA), epidermal growth factor (EGF, 20 ng/mL, Sigma) and heparin sodium salt (0.0004%, Sigma).^40^

At the time of plating, cells were treated with a dose curve of **U42** without any additional treatment during the time course of the experiment. Cultures were maintained at 37 °C + 5% CO_2_ for 8 days. Inverted microscope was used to capture images. For every experimental condition at least five images of the well have been taken to have a more representative overview of the well. The data of every single replicate were percentageised with the means of the control images for each independent experiment. To calculate the number and area of the mammospheres, ImageJ software was used by selecting every single mammosphere in each of the five images for every experimental condition. A total of four independent experiments were conducted, and data were pulled in the histograms.

For Western blot analysis, mammospheres were harvested by centrifuging the medium at 1,200 rpm for 5 min, followed by the lysis of the pellet. Lysates were then quantified. Proteins were resolved in SDS-PAGE and transferred to nitrocellulose membranes using the TurboBlot system, as described above for Western blot analysis.

### eNAMPT Detection Assays

#### Western Blot

MCF7 and 4T1 cells were plated at a concentration of 0.4 × 10^6^ in 6-well plate. The day after cells were treated for 18 h with a dose-dependent curve of **U42**. After this period, cells were washed in 1X PBS and cultured for 18 h in serum-free conditions treating again with **U42**. Media were then harvested and concentrated with cutoff (as described by Grolla et al.).^40^ Concentrated media were then loaded for SDS-PAGE as described above. To monitor eNAMPT levels directly after 18 h of treatment, as shown in Figure S5B, the day after plating cells were washed in 1X PBS and cultured for 24 h in serum-free conditions treating with **U42**.

#### ELISA Assay

After 8 days of culture, mammospheres have been harvested by centrifugating at 1200 rpm for 5 min. The pellets were lysed in RIPA lysis buffer as described above for Western blot assay. Media were harvested and were concentrated with cutoff (as described by Grolla et al.).^40^ eNAMPT levels were then evaluated with a commercially available sandwich enzyme-linked immunosorbent assay kit (ELISA kit from AdipoGen Inc.; Seoul Korea). Indeed, mammospheres could not be starved as it occurred for cells in 2D conditions, and to evaluate eNAMPT levels ELISA assay was used. eNAMPT levels reported in histograms are represented as a ratio between eNAMPT values from ELISA assay and the number of mammospheres for each experimental condition. The data of every single experiment were percentageised with the means of control condition. Finally, all the data from three independent experiments were pulled.

### NAMPT Inhibitory Activity Measurement

Wild type murine NAMPT and murine NMNAT isoform 2 were expressed and purified as described previously.^41,42^ For IC_50_ evaluation, the reaction mixtures contained 35 nM NAMPT (∼2 μg/mL), 1 μM NMNAT2 (∼40 μg/mL), 12.5 U/mL ADH, 0.55 mg/mL BSA, 25 mM MgCl_2_, 75 mM ethanol, 30 mM semicarbazide, 0.6 mM PRPP, 0.3 mM Nam, ATP 1.25 mM dissolved in 30 mM HEPES/NaOH, pH 7.5. Assays were carried out in 96-well plates using 200 μL per well. Each PROTAC was diluted in DMSO and added to the above reaction mixtures ranging from 5 nM to 4 μM, always at 1% DMSO final. Fluorescence readings (excitation at 340 nm and emission at 455 nm) were recorded for 1 h using a Sinergy HT microplate. Temperature control was set at 37 °C. Data outputs were processed by the integrated software and taken only under linearity (coeff reg > 0.985, usually 15–60 min of records). IC_50_ values were next calculated via best fitting in Excel to the eq 1 below

1 $$\mathrm{activity}\%=1/\left(1+\left[I\right]/{\mathrm{IC}}_{50}\right)$$

where “activity %” represents the NAMPT rate relative vs no inhibitor control (arbitrary fixed to 100%). After each assay, individual mixtures with higher concentrations of PROTACs were added with 0.25 mM NMN to see rapidly increasing rates in all cases; this excluded any interference by PROTACs on both ancillary enzymes NMNAT2 and ADH.

### NAPRT Inhibitory Activity Measurement

Recombinant human NAPRT (PMID: 21742010) was assayed in the presence of pure recombinant NadD, NadE obtained as described (PMID: 25223558). Assay mixtures contained the reagents listed above for NAMPT but NMNAT2 and Nam and, in addition, 20 mM K_2_HPO_4_, pH 7.5, 4.5 mM NH_4_Cl, ∼40 μg/mL NadD (0.6 milliU/mL), ∼30 μg/mL NadE (0.3 milliU/mL), and 0.3 mM Na that was used to start the reactions. DMSO control alone at 1% final or mixed to 10 μM final of either **7** or **U42** was included. Incubation was at 37 °C. Fluorescence measurements from the Sinergy HT microplate were handled as described above. The reaction mixtures were finally added with 0.25 mM NaMN to see rapidly increasing rates and thus exclude interference by PROTACs on the ancillary enzymes NadD, NadE, and ADH.

### Intracellular NAD^+^ Level Measurement

Cellular NAD^+^ levels were determined after perchlorate extraction and C18-HPLC analysis as previously described.^43^

### NAMPT-Degrading Competition Assay

After seeding MCF7 and 4T1 cells in 12-well plate, and following overnight incubation in complete MEM culture medium, cells were treated with the lead compound **7** (1 μM) and/or ligand **VHL-Ac** (300 μM). One hour later, 300 nM of **U42** was added to the pretreated cells following the treatment scheme represented in Figure 6B,C. After 18 h cells were lysed as described above and Western blot assay was performed. Data are represented by two independent experiments.

### Cellular Thermal Shift Assay

10^6^ MCF7 and 4T1 cells were plated in 60 mm Petri dish overnight. The day after, cells were treated with DMSO or 10 μM **U14**, **U31**, or **U42** for 5 h. After 5 h, cells were harvested, followed by washing twice with PBS and divided into two aliquots, two for control cells and two for **U42**-treated cells. One aliquot for control and **U42**-treated cells was divided into 6 aliquots, 25 μL each. Two aliquots for every cell line of control **U42**-treated cells were heated at 37 °C. The remaining aliquots were heated at the indicated temperatures (54, 57, 60, 63, 66 °C) for 3 min. After heating, aliquots were immediately put on ice and then RIPA lysis buffer was added in ratio 1:1. After 30 min in ice, aliquots were then subjected to two snap-freeze cycles in liquid nitrogen. After thawing at room temperature, samples were centrifuged at 13000 rcf for 15 min at 4 °C. The resulting supernatants were collected, mixed with loading buffer, and analyzed by Western blot as described above. Data are represented by two independent experiments.

### Statistical Analysis

Graphical representations and statistical analyses were performed using GraphPad Prism software, with data presented as mean ± SEM *t* test parametric (using Welch’s *t* test) was used to compare two column samples and a *p*-value <0.05 was considered statistically significant. For multiple comparisons one way ANOVA (Brown-Forsythe and Welch ANOVA) was used.

### *In Vitro* Metabolic Stability and Pharmacokinetics

#### Materials

Mouse and human liver microsomes, pooled Wistar Han, male MLM, 20 mg/mL and pooled mixed gender donors HLM, 20 mg/mL; CD1Mouse Whole Blood K2EDTA; human recovered plasma pooled K3EDTA were purchased from Tebubio Srl (Magenta, Italy).

#### Incubation in Mouse and Human Plasma

The standard incubation mixture (100 μL final volume) was carried out by dissolving the tested substrate (50 μM) in DMSO (5% final volume) in preincubated plasma at 37 °C. The mixture was shaken for 60 min at 37 °C. Control incubations were carried out without substrate. Each incubation was stopped by addition of 200 μL of ice-cold acetonitrile, vortexed, and centrifuged at 13,000 rpm for 10 min. The supernatants were analyzed by LC-UV.

#### Incubation in Liver Microsomes

The standard incubation mixture (250 μL final volume) was carried out in a 50 mM Tris (tris­[hydroxymethyl]­aminomethane) buffer (pH 7.4) containing 150 mM KCl, 1.5 mM, 3.3 mM MgCl_2_, 1.3 mM NADPNa_2_, 3.3 mM glucose 6-phosphate, 0.4 units/mL glucose 6-phosphate dehydrogenase, acetonitrile as cosolvent (1% of total volume), and the substrate (50 μM). After pre-equilibration of the mixture, an appropriate volume of microsomal suspension was added to give a final protein concentration of 1.0 mg/mL. The mixture was incubated for 60 min at 37 °C in a thermomixer with 700 rpm shaking. Control incubations were carried out without the presence of substrate, or NADPH-regenerating system, or microsomes. Each incubation was stopped by the addition of 250 μL of ice-cold acetonitrile, vortexed, and centrifuged at 13,000 rpm for 5 min prior analysis. For compound **U42** time-dependent substrate depletion experiments were carried out at the concentration of 5 μM in the presence of 1 mM βNADPH in both MLM and HLM. Aliquots (50 μL) were withdrawn at 0–5–10–20–40–60 min and stopped by addition of 100 μL of ice-cold acetonitrile, vortexed, and centrifuged at 13,000 rpm for 5 min. The supernatants were analyzed by HPLC-HRMS. The natural log of the percent remaining is plotted against time and the slope (*k*) of the line determined. Half-life (minutes) and the clearance *in vitro* (μL min^–1^ mg^–1^) were calculated as follow

$$t_{1/2}=\frac{\ln2}{k}$$

$${\mathrm{CL}}_{\mathrm{in}\;\mathrm{vitro}}=\frac{\ln2}{t_{1/2}}\times \frac{1}{\mathrm{microsomes}\;\left(\frac{\mathrm{mg}}{\mathrm{mL}}\right)}\times 1000$$

#### Data Processing

The metabolic stability of the compounds **U31** and **U42** was determined *in vitro* by measuring the residual peak area after incubation by LC-UV analysis (Section S108 in Supporting Information). Samples from compound **U42** were further processed by LC-HRMS equipment for metabolite profiling. Raw data files were processed using both Xcalibur and Compound Discoverer 3.3 software (Thermo Scientific) using a customized workflow for detection and identification of the expected and unknown metabolites (Section S110 in Supporting Information).

#### *In Vivo* PK Studies

Animals were managed in accordance with Italian regulation D.L. 26/2014 on use of animals for scientific purposes and experiments were authorized by the Italian version of Institutional Animal Care and Use Committee Statement (Ministry of Health, 81/2024-PR DB064.86) following strictly three Rs principles. 8-weeks old Balb/c female mice were dosed with **U42** and **U31** i.p. at 20 mg/kg. The compounds were dissolved using a formulation of 50% PEG and 50% saline. Compounds were then heated at 60 °C for 1 h and put in a rotary agitator overnight. After a single administration, 0.1 mL of blood was collected at 5, 15, 30 min and at 1, 2, 4, 8, 24, 48 h form the drug administration (2 mice for each time point). Blood samples collected with EDTA were centrifuged for 5 min at 8000 rpm at 4 °C to obtain plasma for the subsequent analysis.

#### Sample Preparation for LC-HRMS Analysis

Aliquot (50 μL) of each sample was diluted with 100 μL of acetonitrile containing 250 μg/L of **U30** as internal standard. Samples were centrifuged at 13,000 rpm for 10 min and the supernatant injected into the LC-HRMS system (Section S109 in Supporting Information for full instrumental set up). If the levels of analytes were over range, plasma samples were preventively diluted with blank plasma.

#### Data Analysis

Quantification compounds **U31** and **U42** was performed by using the calibration curves obtained with the corresponding working standards of the analytes and using **U30** as internal standard. The pharmacokinetic parameters were calculated by plotting the plasma concentration curves versus time obtained from the average values of two animals, using PK Solver 2.0.^44^

#### Molecular Modeling

Docking poses of compound **7** were reproduced following the previously described protocol. Conformer generation and docking were performed using OMEGA^45,46^ and FRED,^47–49^ respectively, within the active site of NAMPT (PDB ID: 2GVJ). The crystallographic water molecule was omitted, as it was not required to reproduce the reference ligand orientation. Binding interactions were analyzed to assess the π–π stacking of the pyridine ring with Phe193 and Tyr18′ and the potential hydrogen bonding of the triazole moiety with Ser275. The VHL structure was taken from the crystal complex (PDB ID: 7JTO), preserving the coordinates of the bound ligand **10** tail within the recognition pocket. Ternary complex models were generated using the PROTAC-model procedure, which integrates FRODOCK for protein–protein docking,^50^ RosettaDock for structural refinement,^51^ and RDKit for conformational sampling and linker generation.^52^

Molecular Dynamics simulations were conducted with the Desmond module of the Schrödinger Suite (version 2025-1).^53^ Each ternary complex was solvated in a 10 × 10 × 10 Å orthorhombic box of TIP3P water, neutralized with Na^+^ and Cl^–^ ions to 0.15 M ionic strength. Systems were minimized (2000 steps; 50 kcal mol^–1^ Å^–2^ restraints) and gradually equilibrated under NPT conditions through (i) a 12 ps run at 10 K, (ii) heating to 300 K over 24 ps, and (iii) a 24 ps equilibration at 300 K without restraints. Production MD was carried out for 100 ns at 300 K and 1 atm. Trajectories from three independent replicas were analyzed for RMSD, RMSF, and protein–ligand contact profiles using Desmond Simulation Interaction Diagram tools.

## Supplementary Material









## Notes

Representative images of Western blot analysis of PROTAC_S_ **U1–41** and NAMPT degradation by **U23** at different time points (**Figure S1**); the inhibitory activity of selected compounds against the catalytic activity of recombinant NAMPT (**Figure S2**); NAPRT inhibition assays of compound **7** and **U42** (**Figure S3)**; CETSA on 4T1 cells treated with **U31** and **U14** (**Figure S4)**; protein levels of iNAMPT and eNAMPT after treatment with **U42** (**Figure S5**); drug concentration–time curve of **U31** and **U42** (mice, 20 mg/kg, i.p.) (**Figures S6**–**S7**); phase I metabolites of **U42** incubated in mouse and human liver microsomes (**Table S1**); molecular modeling section (**Figure S8**–**S14**); synthetic methods and ^1^H NMR and ^13^C NMR data of the intermediates; ^1^H NMR, ^13^C NMR spectra and HRMS spectra of representative PROTACs; purity evaluation of selected PROTACs by HPLC-UV analysis; LC-UV method for metabolic stability evaluation; LC-HRMS method for pharmacokinetic analysis of **U31** and **U42**; LC-HRMS method for metabolites identification of **U42** in mouse and human liver microsomes, and related data (PDF)
SMILES molecular formula strings (CSV)
MD trajectory movies of the NAMPT–VHL ternary complexes containing **U14** (Movie S1)
VHL ternary complexes containing **U31** (Movie S2)

## Abbreviations Used

AUC: area under the concentration–time curve
CDCl_3_: deuterated chloroform
CD_3_OD: deuterated methanol
(CD_3_)_2_SO: deuterated dimethyl sulfoxide
CRBN: cereblon
*C*_max_: maximum concentration
DBU: 1,8-diazabicycloundec-7-ene
DC_50_: concentration causing 50% protein degradation
DIPEA: diisopropylethylamine
*D*_max_: maximal levels of degradation
DME: 1,2-dimethoxyethane
DMAP: dimethylaminopyridine
DMF: *N,N-*dimethylformamide
DMSO: dimethyl sulfoxide
DPPA: diphenylphosphoryl azide
EDCl: *N*-(3-(dimethylamino)­propyl)-*N*′-ethylcarbodimide hydrochloride
EDTA: ethylenediaminetetraacetic acid
EtOAc: ethyl acetate
eNAMPT: extracellular NAMPT
HATU: 1-[bis­(dimethylamino)-methylene]-1*H*-1,2,3-triazolo­[4,5-*b*]-pyridinium 3-oxid hexafluorophosphate
HLM: human liver microsomes
HPLC: high-performance liquid chromatography
IC_50_: half-maximal inhibitory concentration
iNAMPT: intracellular NAMPT
i.p.: intraperitoneal injection
LC-UV: liquid chromatography coupled to ultraviolet detector
LC-HRMS: liquid chromatography coupled to high resolution mass spectrometry
MeOH: methanol
MLM: mouse liver microsomes
NAD^+^: nicotinamide adenine dinucleotide
NAMPT: nicotinamide phosphoribosyltransferase
PROTACs: proteolysis-targeting chimeras
PK: pharmacokinetic
PE: petroleum ether
PEG: poly­(ethylene glycol)
*T*_1/2_: elimination half-life
TEA: triethylamine
THF: tetrahydrofuran
TsCl: tosyl chloride
UPS: ubiquitin-proteasome system
VHL: von Hippel-Lindau
