# Supplementary material for: Development of PROTACs Targeting the Moonlighting Enzyme Nicotinamide Phosphoribosyltransferase (NAMPT) for Breast Cancer Therapy
Source: J Med Chem. 2026 Feb 19;69(5):5259–90. doi: 10.1021/acs.jmedchem.5c01827 (PMC12990041; doi:10.1021/acs.jmedchem.5c01827)
Supplement: Supplementary file 1 [file jm5c01827_si_001.pdf]

SUPPORTING INFORMATION

Development of PROTACs Targeting the Moonlighting Enzyme Nicotinamide Phosphoribosyltransferase (NAMPT) for Breast Cancer Therapy

Ubaldina Galli,[1,†,*] Marianna Moro,[1,†] Federica Carolina Balestrero,[1] Giorgia Colombo,[1,2] Marco Koten,[1] Benedetta Roncaglio,[1] Armando A. Genazzani,[1,3] Silvio Aprile,[1,4] Alberto Massarotti,[1] Giuseppe Orsomando,[5] Tracey Pirali,[1,*] Ambra A. Grolla[1]

[1] Department of Pharmaceutical Sciences, Università degli Studi del Piemonte Orientale, Largo Donegani 2, 28100, Novara, Italy

[2] Division of Haematology/Oncology Department of Medicine, Weill Cornell Medicine, Cornell University, 413 E 69th St, New York, NY 10021, USA

[3] Department of Drug Science and Technology, Università degli Studi di Torino, Via Pietro Giuria 9, 10125, Torino, Italy

[4] Novanalitica Srls., Corso Trieste 15/a, 28100, Novara, Italy

[5] Department of Clinical Sciences (DISCO), Section of Biochemistry, Polytechnic University of Marche, Via Ranieri 67, Ancona 60131, Italy

* Correspondence: Ubaldina Galli (ubaldina.galli@uniupo.it); Tracey Pirali (tracey.pirali@uniupo.it)

† These authors contributed equally to this work.

Table of Contents

Figure S1.	p. S3
Figure S2.	P. S4
Figure S3.	P. S5
Figure S4.	p. S6
Figure S5.	p. S7
Figure S6.	p. S8
Figure S7.	p. S8
Table S1.	p. S9
Figure S8.	p. S10
Figure S9.	p. S11
Figure S10.	p. S11
Figure S11.	p. S12
Figure S12.	p. S12
Figure S13.	p. S13
Figure S14.	p. S13
Synthetic methods and ^{1}H-NMR and ^{13}C-NMR data of the intermediates.	p. S14
NMR spectra of PROTACs U14-25, U30-35, U42.	p. S49
HRMS spectra of PROTACs U14-25, U30-35, U42.	p. S68
Purity evaluation of selected PROTACs by HPLC-UV analysis.	p. S87
LC-UV method for metabolic stability evaluation.	p. S108
LC-HRMS method for pharmacokinetic analysis of PROTACs U31 and U42.	p. S109
LC-HRMS method for metabolites identification of PROTAC U42.	p. S110
LC-HRMS data of U42 and its metabolites in MLM and HLM.	p. S112

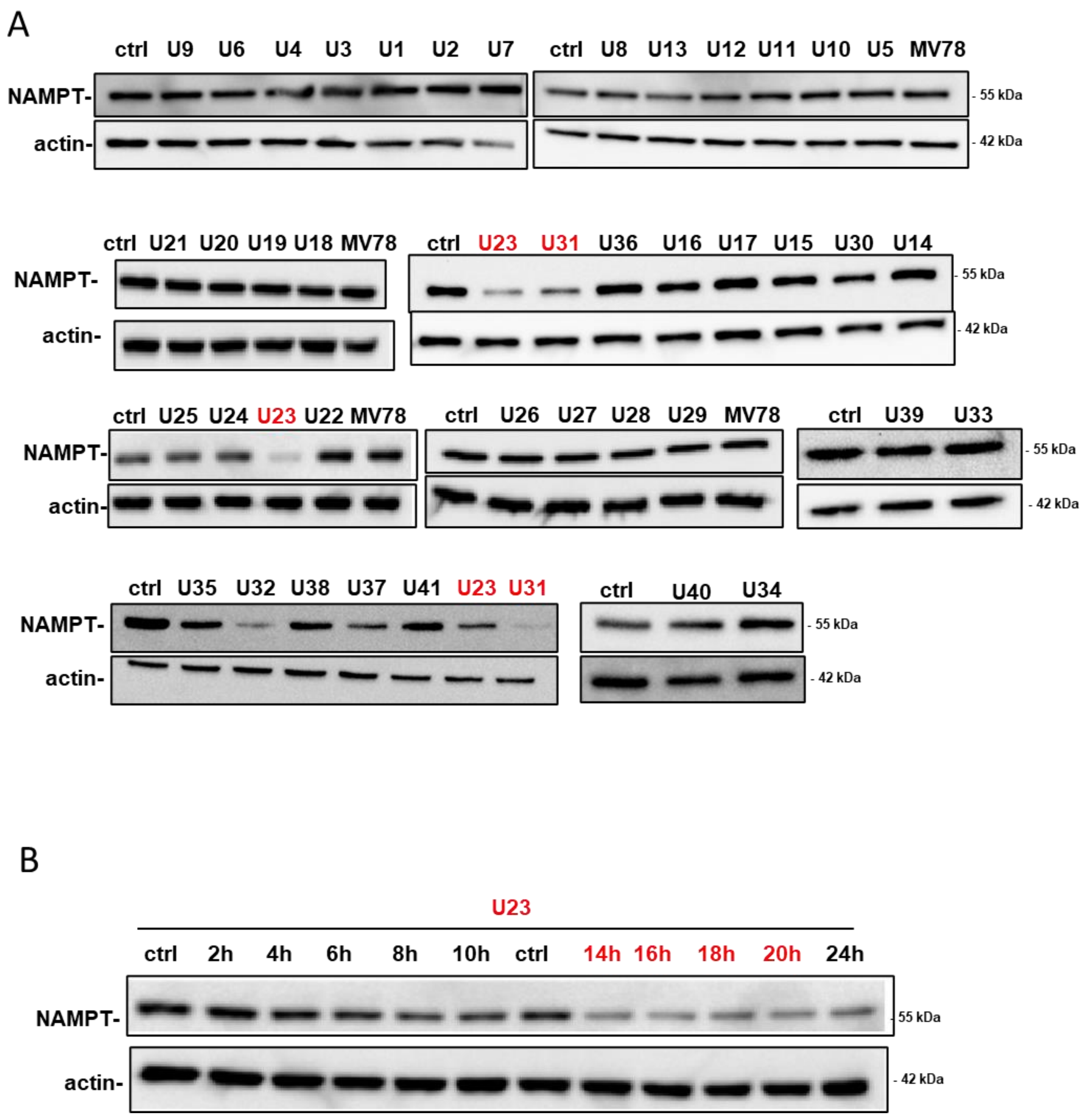

Figure S1. (**A**) Representative images of Western blot analysis performed after 18 hours on MCF7 cells treated with **MV78** (**7**) and **U1-41** at the dose of 1 μM. Only compounds with Dmax > 50% have been taken in consideration (red labelled). (**B**) NAMPT degradation by **U23** at different time points. After 14 hours of incubation, degradation exceeded 50%.

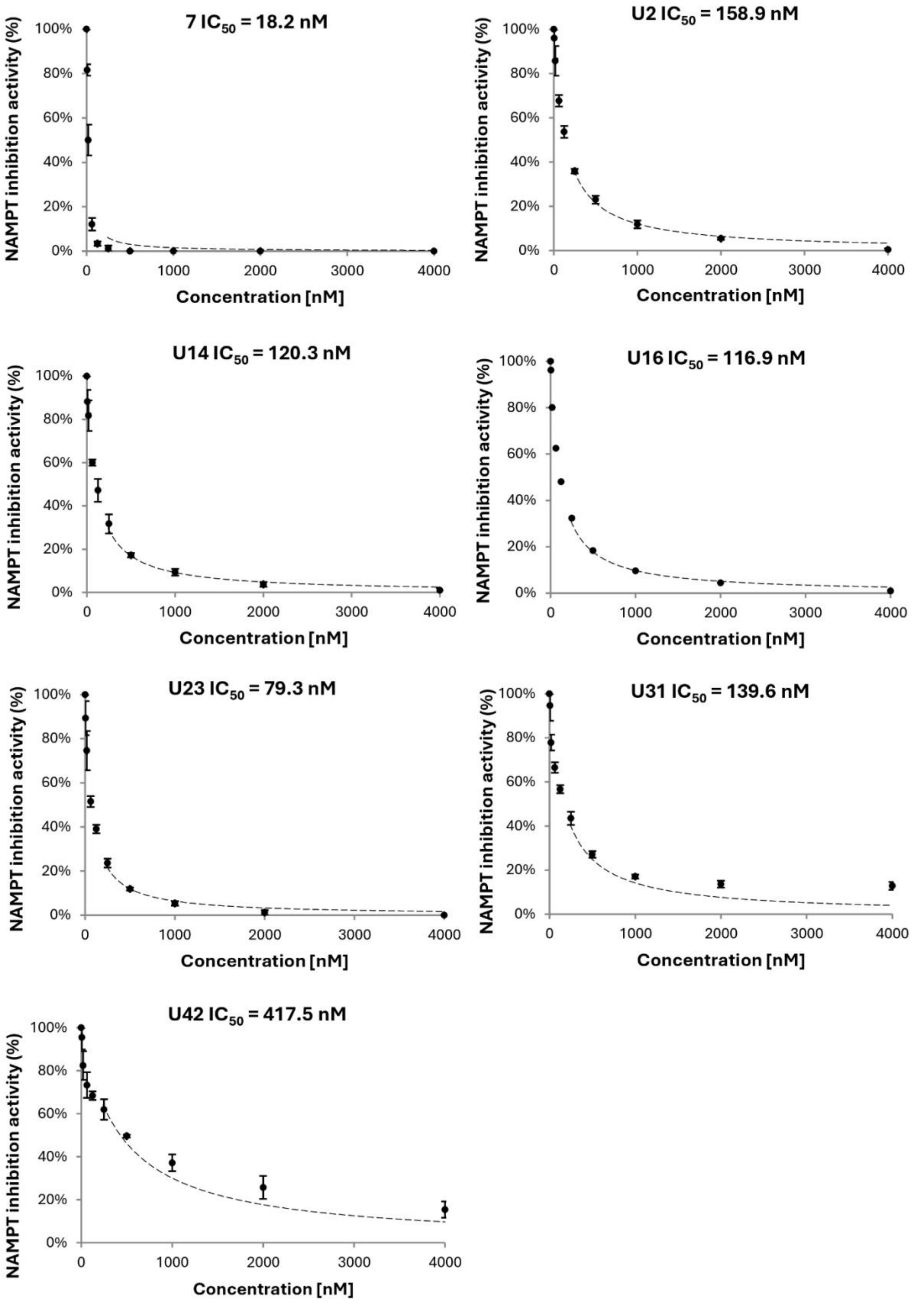

Figure S2. Screening of the NAMPT inhibitory activity of compounds **7**, **U2**, **U14**, **U16**, **U23**, **U31**, **U42.**

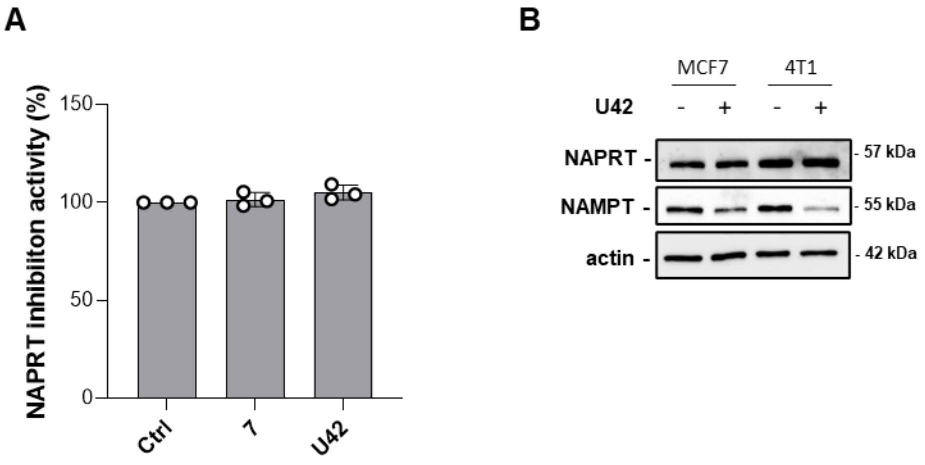

Figure S3. (**A**) NAPRT inhibitory activity of compounds **7** and **U42** at 10 µM. Results are expressed by the mean of 3 independent experiments. (**B**) Protein levels of NAPRT and NAMPT after 18 hours of treatment with 300 nM **U42** in both MCF7 and 4T1 cells.

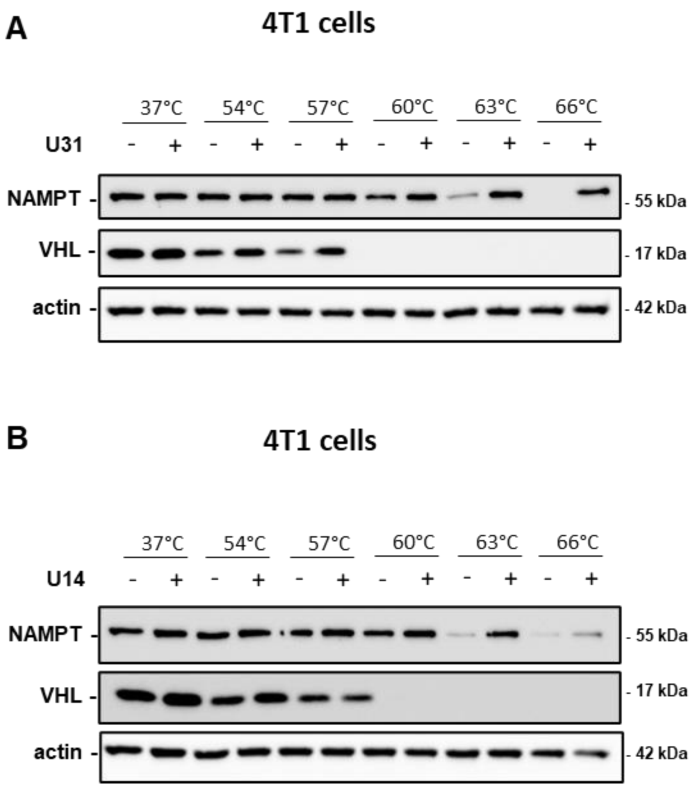

Figure S4. NAMPT and VHL levels in 4T1 cells treated with **U31** (**A**) and **U14** (**B**) (10 μM) for 5 hours by cellular thermal shift assay.

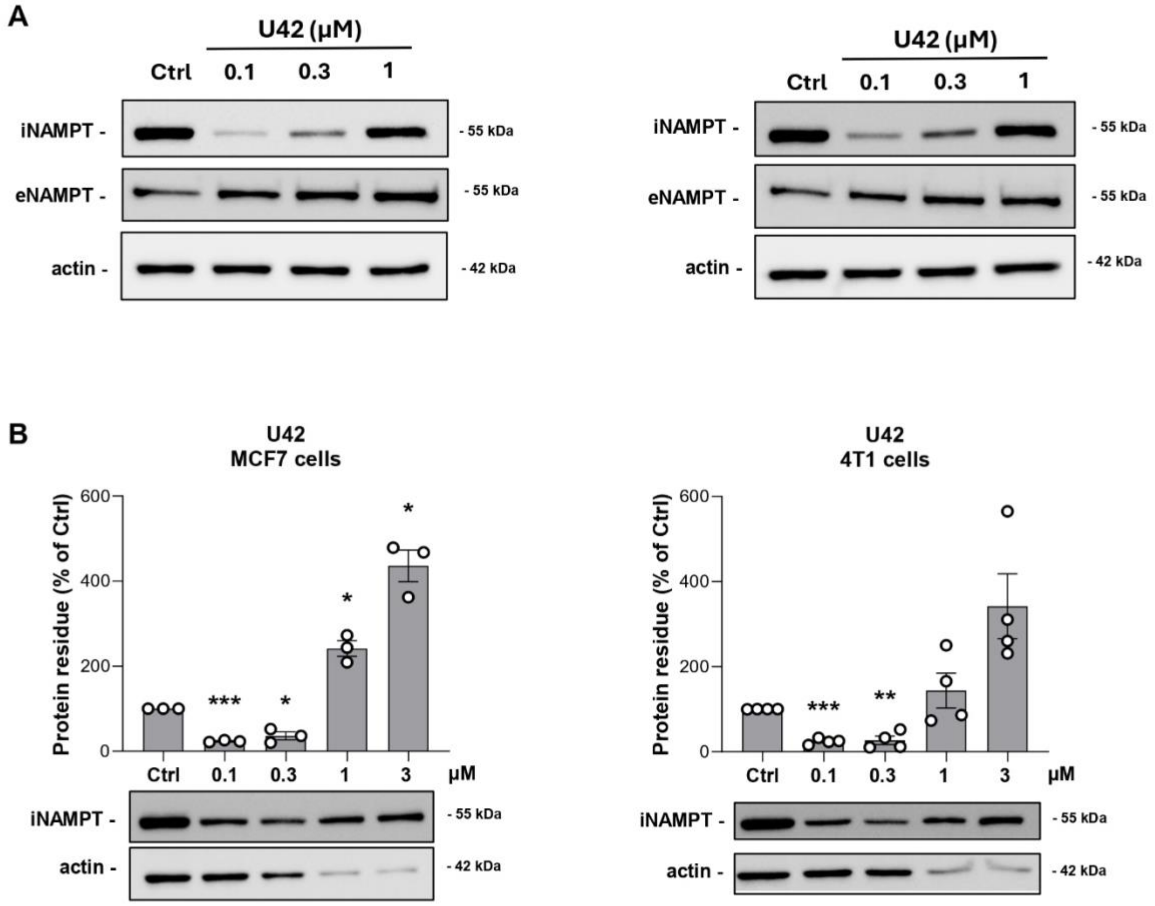

Figure S5. (**A**) Protein levels of iNAMPT and eNAMPT after 18 hours of treatment with **U42** at different concentrations in serum-free conditions in MCF7 (left) and 4T1 (right) cells. (**B**) iNAMPT protein levels in both MCF7 and 4T1 treated with **U42** for 18 hours, washed, and continued treatment in serum-free medium for additional 24 hours.

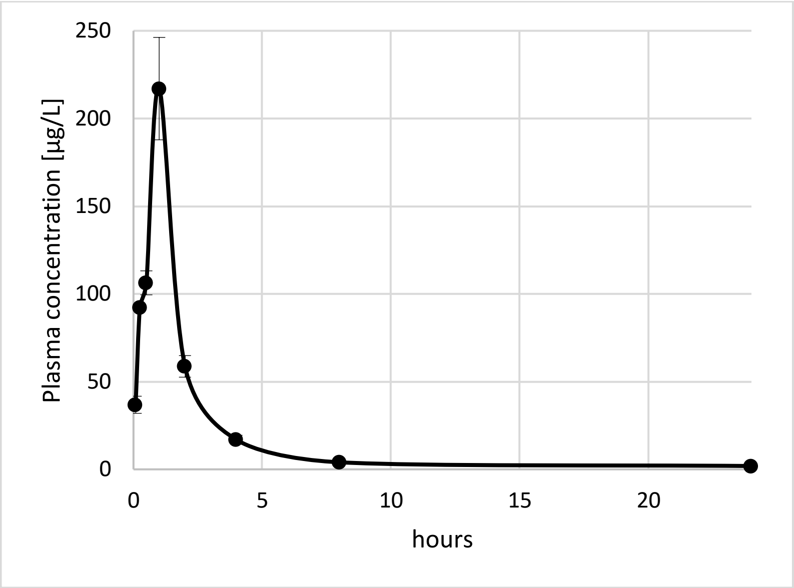

Figure S6. Drug concentration−time curve (±SD) of **U31** (mice, 20 mg/Kg, i.p.).

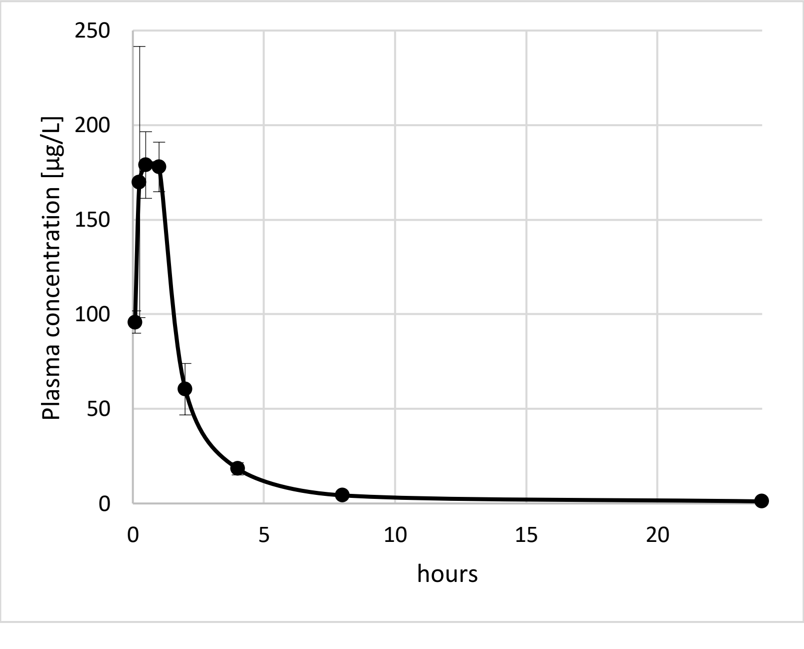

Figure S7. Drug concentration−time curve (±SD) of **U42** (mice, 20 mg/Kg, i.p.).

	Retention time (min)	Theoretical $[M+H]^+$	Measured $[M+H]^+$	Measured $[M+Na]^+$	Measured $[M+2H]^{+2}$	Δ Da ppm
**U42**	18.24	1024.54773	1024.54657	1046.52869	512.77820	1.13
**M1**	15.36	1040.54267	1040.54319	1062.52393	520.77531	0.50
**M2**	15.72	1040.54267	1040.54297	1062.52649	520.77545	0.29
**M3**	15.99	1040.54267	1040.54065	1062.52100	520.77551	1.94
**M4**	16.58	1022.53206	-	-	511.77004	0.52
**M5**	17.88	1040.54267	1040.54292	1062.52502	520.77521	0.24
**M6**	9.89	456.23940	456.23958	478.22159	-	0.39
**M7**	12.89	585.31052	585.31073	607.29260	-	0.36
**M8**	3.82	261.17099	261.17105	-	-	0.23
**M9**	3.68	275.15025	275.15033	-	-	0.29
**M10**	17.25	782.39458	782.39447	804.37643	-	0.14

Table S1. Phase I metabolites of compound **U42** incubated in mouse and human liver microsomes and detected by LC-HRMS analysis.

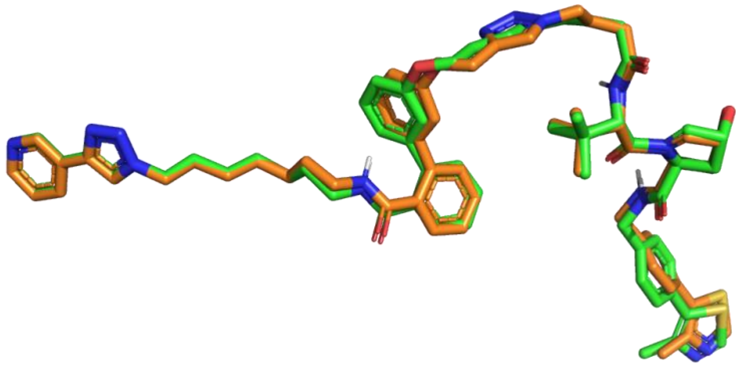

Figure S8. Superimposition of docking poses of **U14** (orange) and **U31** (green).

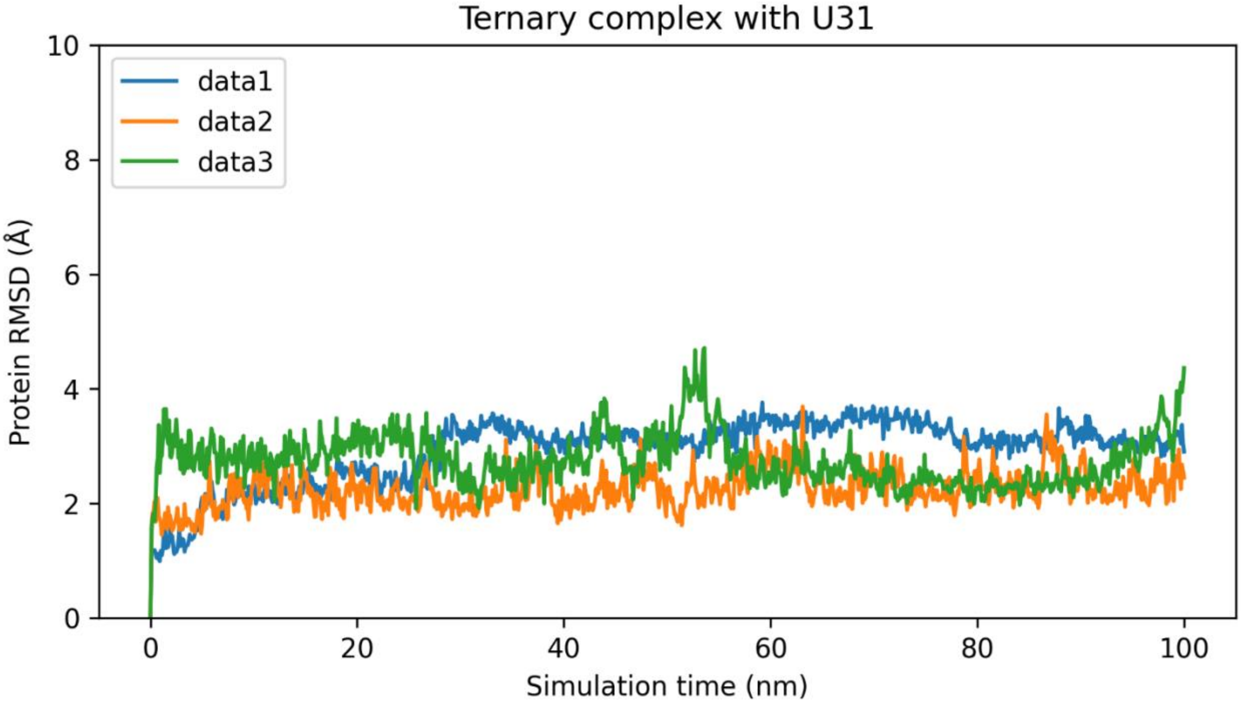

Figure S9. The RMSD for Cα atoms (Å) with respect to the initial structures as functions of simulation time (ns) for protein in the ternary complex with **U31** (three replica were performed).

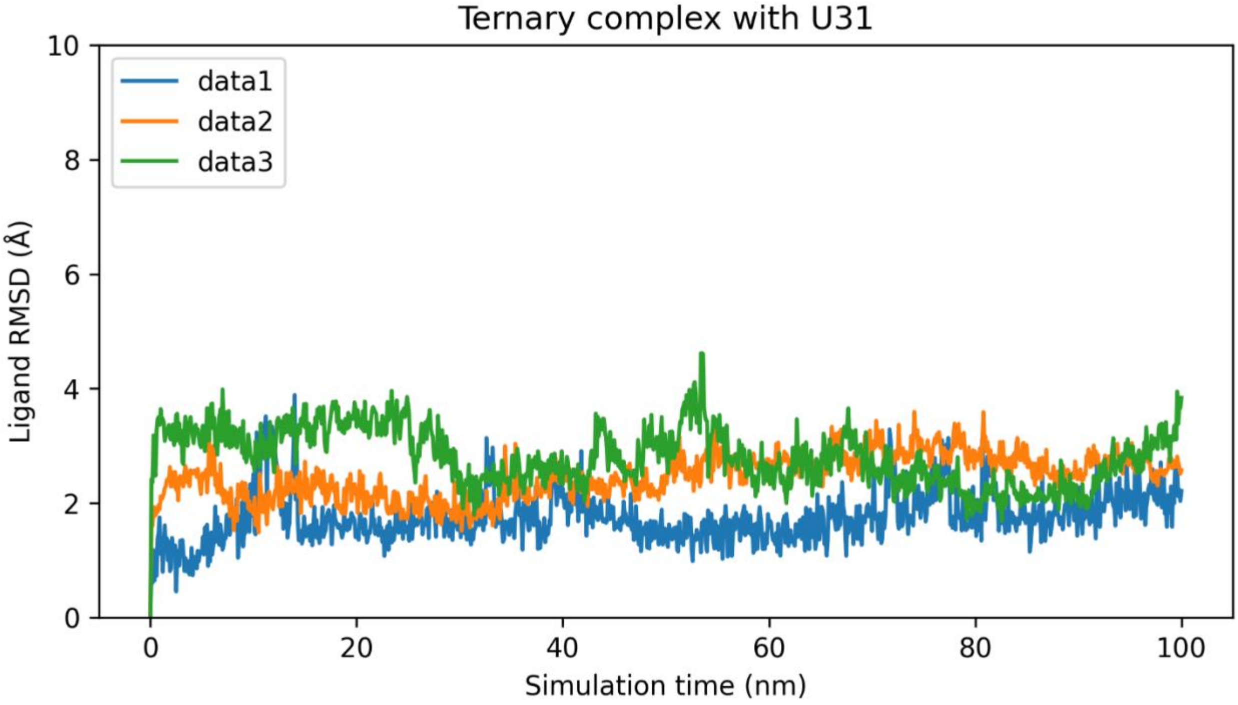

Figure S10. The RMSD for heavy atoms (Å) with respect to the initial structures as functions of simulation time (ns) for the ligands in the ternary complex with **U31** (three replica were performed).

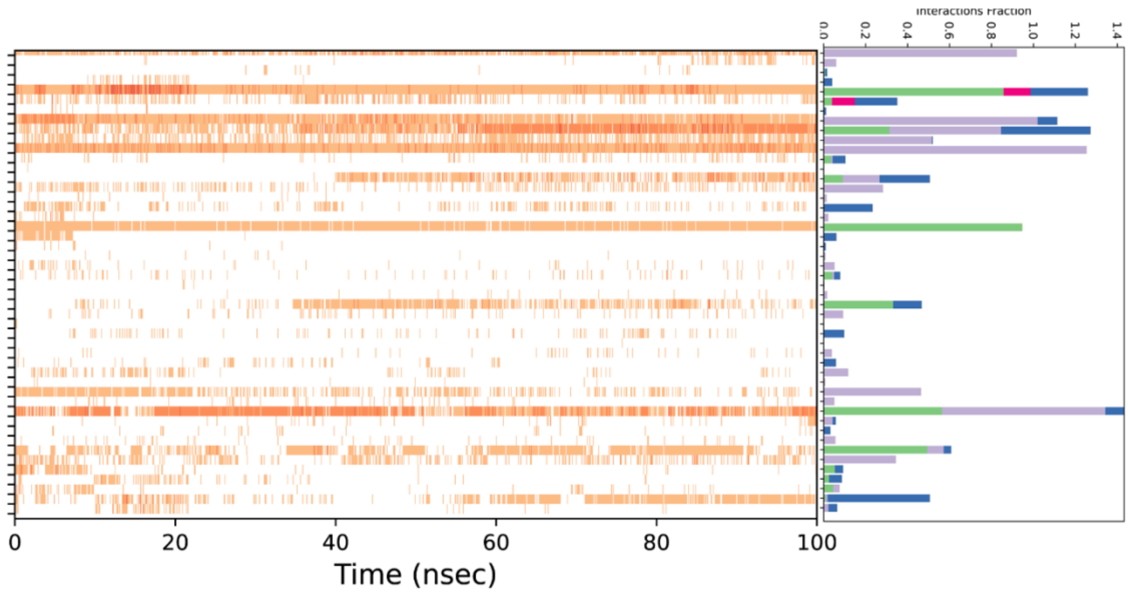

Figure S11. A timeline representation of the interactions and protein-ligand contacts of ternary complex with **U31**. The left side shows which residues interact with the ligand in each trajectory frame. Some residues make more than one specific contact with the ligand, which is represented by a darker shade of orange. The right side shows the interactions categorized by type: Hydrogen Bonds (green), Hydrophobic (violet), Ionic (purple) and Water Bridges (blue).

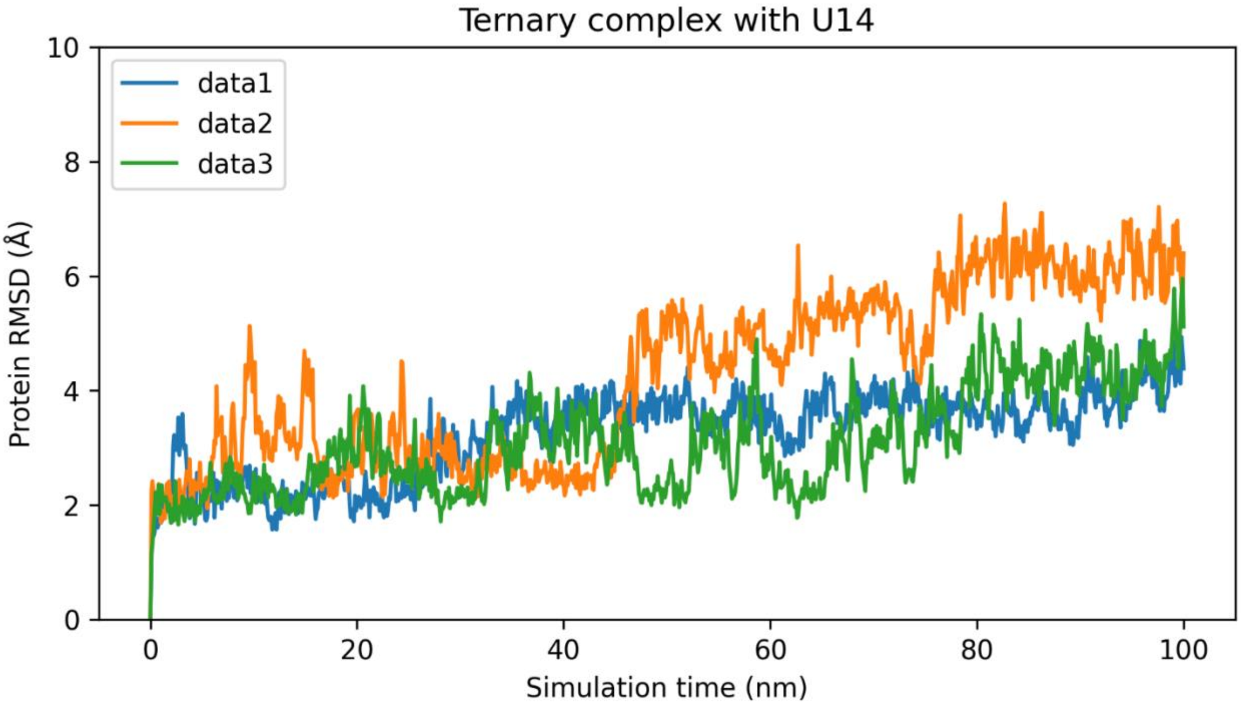

Figure S12. The RMSD for Cα atoms (Å) with respect to the initial structures as functions of simulation time (ns) for protein in the ternary complex with **U14** (three replica were performed).

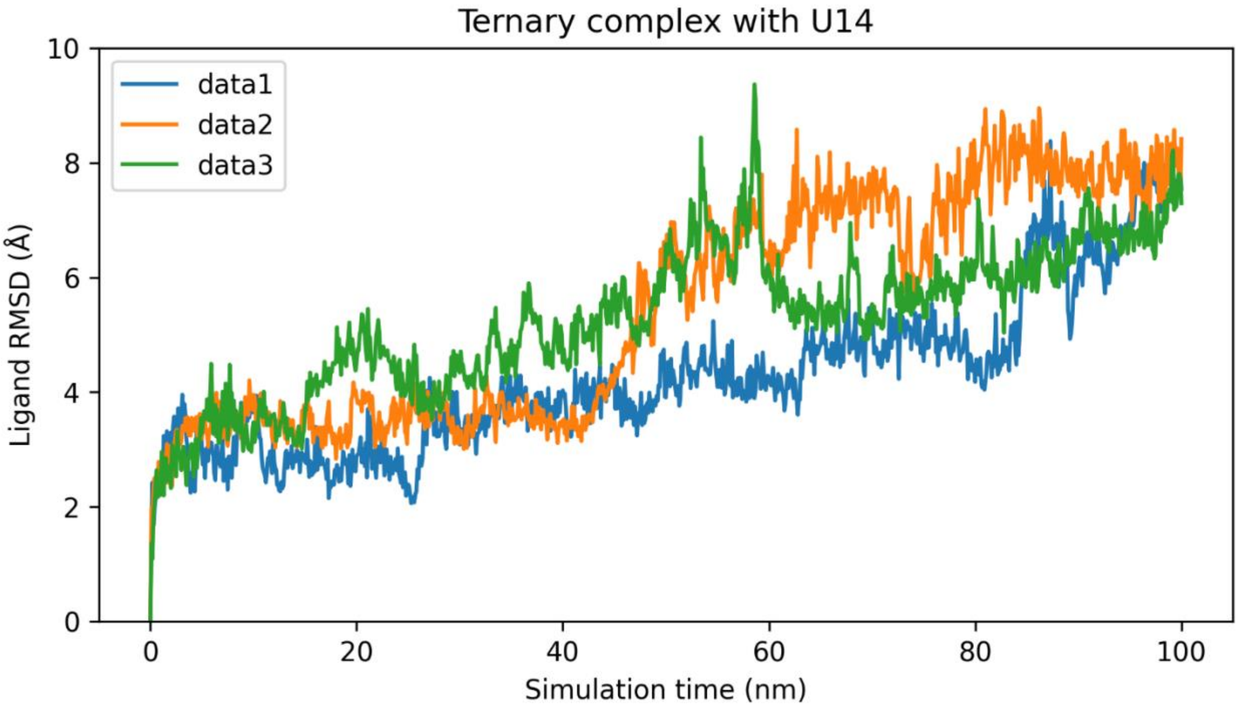

Figure S13. The RMSD for heavy atoms (Å) with respect to the initial structures as functions of simulation time (ns) for the ligands in the ternary complex with **U14** (three replica were performed).

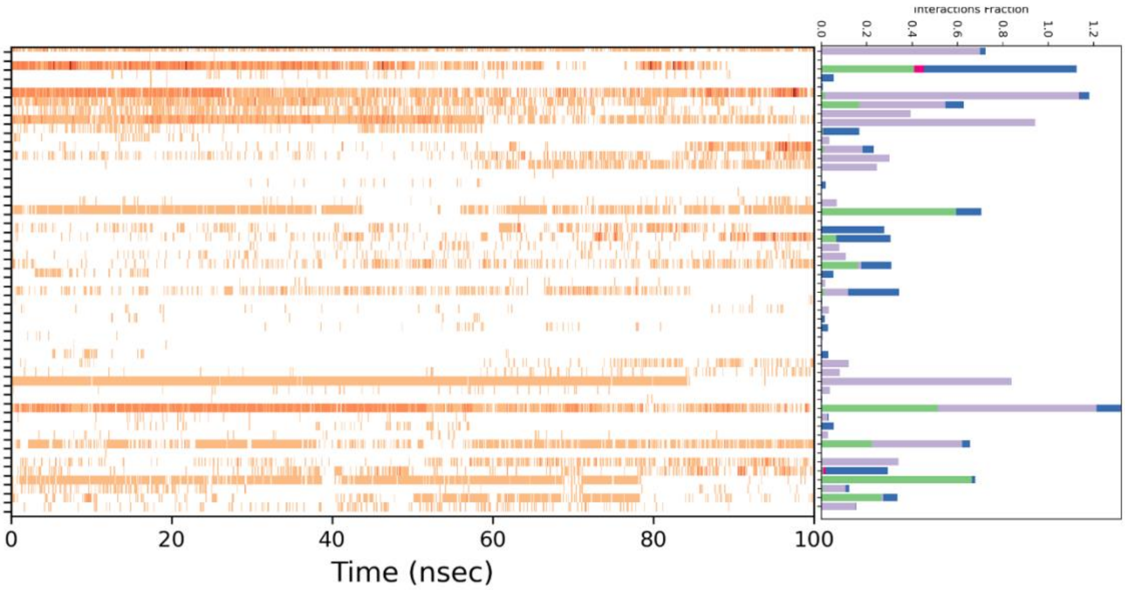

Figure S14. A timeline representation of the interactions and protein-ligand contacts of ternary complex with **U14**. The left side shows which residues interact with the ligand in each trajectory frame. Some residues make more than one specific contact with the ligand, which is represented by a darker shade of orange. The right side shows the interactions categorized by type: Hydrogen Bonds (green), Hydrophobic (violet), Ionic (purple) and Water Bridges (blue).

Synthetic methods and ^{1}H-NMR and ^{13}C-NMR data of the intermediates.

^{1}H-NMR, ^{13}C-NMR and IR data of the intermediates for the synthesis of the NAMPT ligand (8)

7-Azidoheptanoic acid (11)

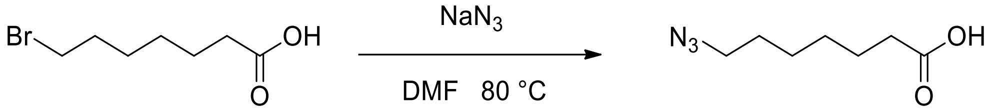

7-Bromoheptanoic acid (1 equiv) was dissolved in DMF (0.4 M). While stirring at room temperature sodium azide was added (1.2 equiv) and the reaction was heated at 80 °C overnight. After completion of the reaction, water was added, and the desired product was extracted with diethyl ether (x3). The combined organic layers were washed with water (x3), brine (x1), dried over anhydrous sodium sulfate and concentrated *in vacuo* to give 7-azidoheptanoic acid (**11**) as a colorless oil, yield 94%, which was used in the next step without further purification. IR (KBr) 2950, 2870, 2095, 1712, 1424, 1258, 665 cm^{-1}; ^{1}H NMR (300 MHz, $CDCl_3$) δ 11.55 (br s, 1H), 3.14 (t, J = 6.8 Hz, 2H), 2.22 (t, J = 7.2 Hz, 2H), 1.56-1.45 (m, 4H), 1.30-1.24 (m, 4H) ppm; ^{13}C NMR (75 MHz, $CDCl_3$) δ 178.3, 51.1, 33.7, 28.5, 28.4, 26.2, 24.4 ppm.

7-(4-(Pyridin-3-yl)-1*H*-1,2,3-triazol-1-yl)heptanoic acid (12)

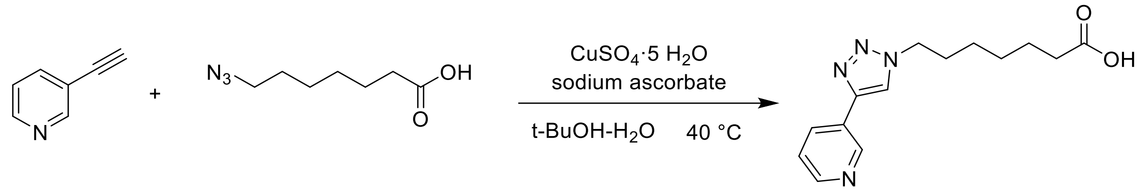

3-Ethynylpyridine (1 equiv) and azide **11** (1 equiv) were suspended in a mixture of water/*tert*-butanol (1:1), then sodium ascorbate (0.1 equiv), of a freshly prepared 1 M solution in water, was added, followed by the addition of $CuSO_4{\cdot}5H_2O$ (0.01 equiv). The resulting mixture was heated to 40 °C and vigorously stirred for 24 h. The reaction mixture was then diluted with water, cooled to a 0 °C, and the precipitate was collected by vacuum filtration, washed with diethyl ether and dried *in vacuo*. The triazole **12** was obtained as a yellowish amorphous solid, yield 90%; IR (KBr) 3560, 2975, 1715, 1420, 1055, 1030, 815 cm^{-1}; ^{1}H NMR (300 MHz, $(CD_3)_2SO$) δ 11.80 (br s, 1H), 9.03 (br s, 2H), 8.60 (s, 1H), 8.25 (d, J = 7.3 Hz, 1H), 7.60 (br s, 1H), 4.40 (t, J = 6.7 Hz, 2H), 2.30 (br s, 2H), 1.85 (m, 2H), 1.53 (br s, 2H), 1.25 (br s, 4H) ppm; ^{13}C NMR (75 MHz, $(CD_3)_2SO$) δ 182.5, 147.9, 147.5, 145.8, 134.3, 133.2, 130.3, 124.2, 51.9, 37.6, 31.6, 29.9, 27.8, 25.6 ppm.

Methyl 7-(4-(pyridin-3-yl)-1*H*-1,2,3-triazol-1-yl)heptanoate (13)

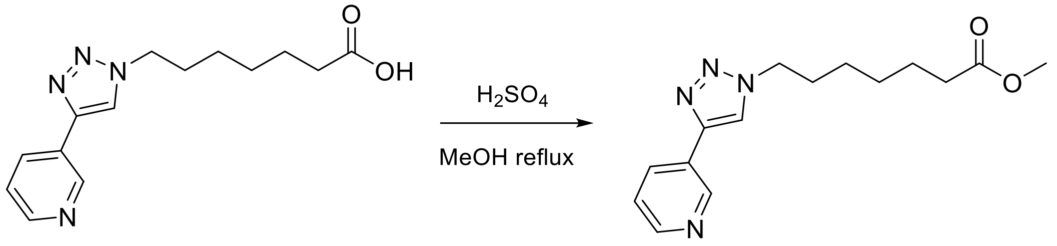

H_2SO_4 96% w/w (1.1 equiv) was added to a solution of **12** (1 equiv) in methanol (0.4 M) and the reaction mixture was heated under reflux for 2 h. Subsequently the solvent was evaporated under vacuum, the solid residue was diluted with a saturated aqueous $NaHCO_3$ solution and extracted with EtOAc (x3). The combined organic layers were washed with brine (x1), dried over anhydrous Na_2SO_4, and concentrated *in vacuo* to give the methyl ester **13**, as a white solid, yield 85%, which was used in the next step without further purification. IR (KBr) 2942, 1732, 1442, 1053, 1032, 806 cm^{-1}; ^1H NMR (300 MHz, $CDCl_3$) δ 8.90 (d, J = 2.2 Hz, 1H), 8.43 (d, J = 3.6 Hz, 1H), 8.06 (d, J = 7.7 Hz, 1H), 7.84 (s, 1H), 7.24 (dd, J = 8.0/5.0 Hz, 1H), 4.30 (t, J = 7.1 Hz, 2H), 3.52 (s, 3H), 2.17 (t, J = 7.2 Hz, 2H), 1.84 (br quint, 2H), 1.49 (br quint, 2H), 1.27-1.22 (m, 4H) ppm; ^{13}C NMR (75 MHz, $CDCl_3$) δ 175.2, 150.6, 148.4, 146.1, 134.5, 128.4, 125.3, 121.6, 53.0, 51.9, 35.3, 31.6, 29.9, 27.6, 26.1 ppm.

7-(4-(Pyridin-3-yl)-1*H*-1,2,3-triazol-1-yl)heptan-1-ol (14)

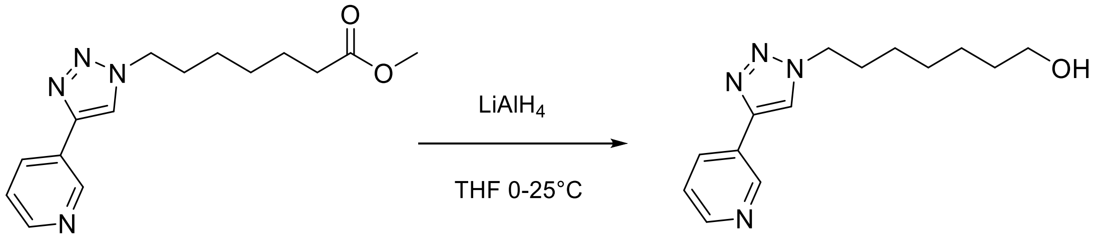

To a solution of methyl ester **13** (1 equiv) in dry THF (0.2 M), cooled to 0 °C, under magnetic stirring and nitrogen, $LiAlH_4$ (1.5 equiv) was carefully added. The reaction mixture was left to reach room temperature and stirred for 30 minutes. Upon completion, the reaction was cooled down to 0 °C and quenched by dropwise addition of 2M NaOH aqueous solution under stirring and a white precipitate was formed. The suspension was then filtered under vacuum and the precipitate was rinsed with MeOH. The filtrate obtained was concentrated *in vacuo* and the residue was purified by column chromatography using CH_2Cl_2/MeOH 98:2 and CH_2Cl_2/MeOH 95:5 as eluants to give alcohol **14** as a white solid, yield 98%; m.p. 96-97 °C; IR (KBr) 3268, 2933, 1455, 1056, 705 cm^{-1}; ^1H NMR (300 MHz, $CDCl_3$) δ 8.93 (d, J = 1.5 Hz, 1H), 8.48 (dd, J = 4.6/1.5 Hz, 1H), 8.13 (dt, J = 8.0/1.8 Hz, 1H),

7.85 (s, 1H), 7.31 (dd, *J* = 8.0/4.9 Hz, 1H), 4.35 (t, *J* = 7.4 Hz, 2H), 3.57 (t, *J* = 6.4 Hz, 2H), 2.94 (br s, 1H), 1.90 (br quint 2H), 1.49 (br quint, 2H) 1.31 (br s, 6H) ppm; ^{13}C NMR (75 MHz, $CDCl_3$) δ 150.5, 148.4, 146.2, 134.6, 128.5, 125.4, 121.6, 64.0, 52.1, 34.1, 31.7, 30.3, 27.9, 27.1 ppm.

3-(1-(7-Azidoheptyl)-1*H*-1,2,3-triazol-4-yl)pyridine (15)

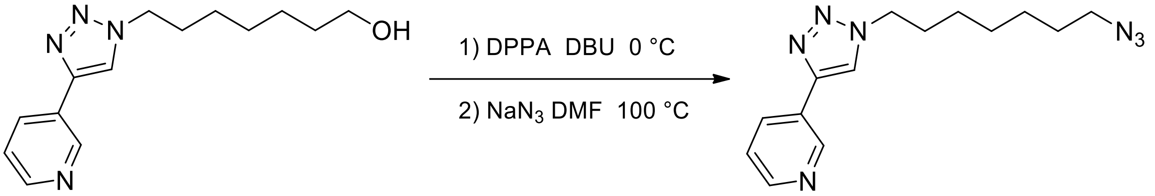

To a solution of **14** (1 equiv) in DMF (0.4 M) cooled down to 0 °C and under nitrogen atmosphere, DPPA (2 equiv) and DBU (2 equiv) were added, and the reaction was stirred for 30 minutes. Then NaN_3 (2 equiv) was added, and the reaction mixture was heated to 100 °C and stirred for 3 h. After cooling to room temperature, the reaction was worked up by dilution with water and extracted with diethyl ether (x5). The combined organic layers were then washed with water (x2), brine (x1), dried over anhydrous Na_2SO_4, and concentrated *in vacuo*. Finally, the crude material was purified by column chromatography using PE/EtOAc 2:8 as eluent to give the azide **15** as a pale yellow oil, yield 90%; IR (KBr) 2934, 2858, 2094, 1589, 1488, 1187, 970 cm^{-1}; ^{1}H NMR (300 MHz, $CDCl_3$) δ 8.96 (d, *J* = 1.5 Hz, 1H), 8.48 (dd, *J* = 3.4/1.2 Hz, 1H), 8.14 (dt, *J* = 8.0/1.8 Hz, 1H), 7.88 (s, 1H), 7.30 (dd, *J* = 8.0/4.9 Hz,1H), 4.33 (t, *J* = 7.4 Hz, 2H), 3.15 (t, *J* = 6.7 Hz, 2H), 1.87 (quint, *J* = 6.7 Hz, 2H), 1.48 (quint, *J* = 7.0 Hz, 2H), 1.28 (br s, 6H) ppm; ^{13}C NMR (75 MHz, $CDCl_3$) δ 150.1, 148.1, 145.9, 134.9, 128.7, 125.5, 121.8, 52.8, 52.0, 31.7, 30.2, 30.0, 28.0, 27.8 ppm.

7-(4-(Pyridin-3-yl)-1*H*-1,2,3-triazol-1-yl)heptan-1-amine (16)

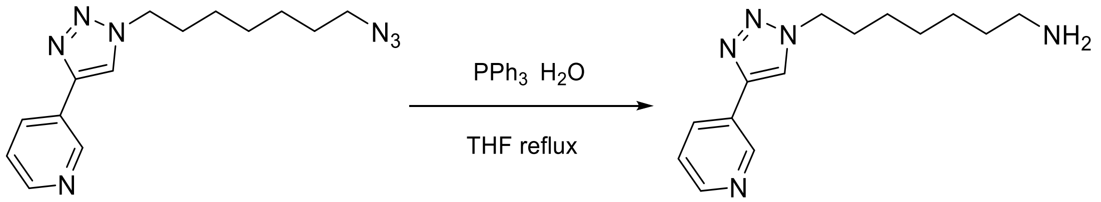

Water (6 equiv) and triphenylphosphine (1.5 equiv) were added to a solution of azide **15** (1 equiv) in THF (0.3 M) and the reaction mixture was heated at reflux for 3 h. Upon completion, the solvent was evaporated under vacuum and the crude was purified by column chromatography using EtOAc/MeOH 7:3 + 2% NH_3 and EtOAc/MeOH 5:5 + 2% NH_3 as eluents. The resulting product was suspended in CH_2Cl_2 and then filtered under vacuum. The filtrate was evaporated under vacuum and the amine **16** was obtained as a white solid, yield 64%; m.p. 89-91 °C; IR (KBr) 2926, 2849, 1560, 1474, 1027, 808 cm^{-1}; ^{1}H-NMR (300 MHz, $CDCl_3$) δ 8.96 (d, *J* = 0.9 Hz, 1H), 8.51 (dd, *J* = 4.9/1.5

Hz, 1H), 8.16 (dt, J = 8.0/1.5 Hz, 1H), 7.84 (s, 1H), 7.34-7.31 (m, 1H), 4.37 (t, J = 7.4 Hz, 2H), 2.64 (t, J = 7.0 Hz, 2H), 2.34 (br s, 2H), 1.91 (br quint, 2H), 1.40 (br quint, 2H), 1.30 (br s, 6H) ppm; ^{13}C-NMR (75 MHz, $CDCl_3$) δ 148.9, 146.8, 144.5, 132.9, 126.8, 123.7, 120.1, 50.4, 41.6, 32.6, 30.1, 28.6, 26.5, 26.3 ppm.

General procedure for the tosylation of the alkyne-ols (31-39)

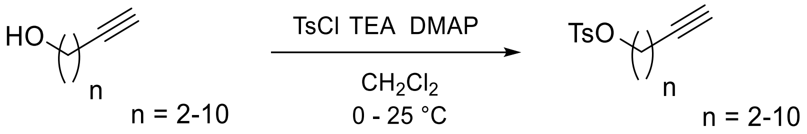

TEA (1.5 equiv) and DMAP (0.05 equiv) were added to a solution of the corresponding alkyne (**18-26**) (1 equiv) in anhydrous CH_2Cl_2 (0.45 M). The solution was cooled to 0 °C, then TsCl (1.2 equiv) was added portionwise and the reaction was stirred overnight under nitrogen atmosphere at 25 °C. Upon completion, the reaction was diluted with water and extracted with CH_2Cl_2 (x2). The combined organic layers were then washed with a saturated $NaHCO_3$ aqueous solution (x1), 2 M HCl aqueous solution (x1) and brine (x1). The organic layer was dried over anhydrous Na_2SO_4, filtered and the solvent was evaporated under vacuum. The reaction crude was then purified by flash chromatography.

But-3-yn-1-yl 4-methylbenzenesulfonate (31)

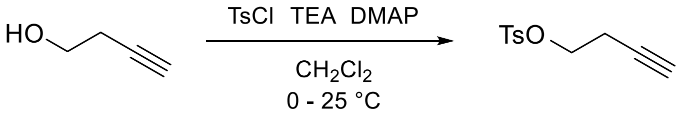

The compound was prepared from the commercially available but-3-yn-1-ol (**18**), according to general procedure. The crude was purified by flash column chromatography using PE/EtOAc 9.5:0.5 and PE/EtOAc 9:1 as eluents to give a pale yellow oil; yield 66%; ^{1}H NMR (400 MHz, $CDCl_3$) δ 7.77 (d, J = 8.3 Hz, 2H), 7.33 (d, J = 8.0 Hz, 2H), 4.07 (t, J = 6.9 Hz, 2H), 2.52 (td, J = 6.9/2.7 Hz, 2H), 2.41 (s, 3H), 1.97 (t, J = 2.7 Hz, 1H) ppm; ^{13}C NMR (101 MHz, $CDCl_3$) δ 143.0, 132.7, 130.0, 127.9, 78.5, 70.9, 67.6, 21.6, 19.4 ppm.

Pent-4-yn-1-yl 4- methylbenzenesulfonate (32)

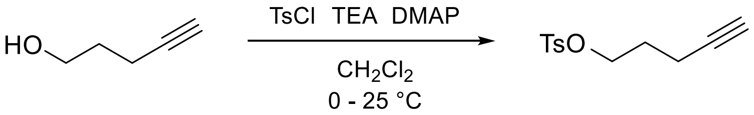

The compound was prepared from the commercially available pent-4-yn-1-ol (**19**), according to general procedure. The crude was purified by flash column chromatography using PE/EtOAc 9.5:0.5

and PE/EtOAc 9:1 as eluents to give a pale yellow oil; yield 90%; ^{1}H NMR (400 MHz, $CDCl_3$) δ 7.69 (d, J = 7.0 Hz, 2H), 7.28 (d, J =7.3 Hz, 2H), 4.05 (t, J = 6.1 Hz, 2H), 2.35 (br s, 3H), 2.16 (t, J = 6.1 Hz, 2H), 1.84 (t, J = 2.5 Hz, 1H), 1.76 (quint, J = 6.1 Hz, 2H) ppm; ^{13}C NMR (101 MHz, $CDCl_3$) δ 144.7, 132.6, 129.7, 127.6, 81.9, 69.4, 68.6, 27.5, 21.4, 14.4 ppm.

Hex-5-yn-1-yl 4- methylbenzenesulfonate (33)

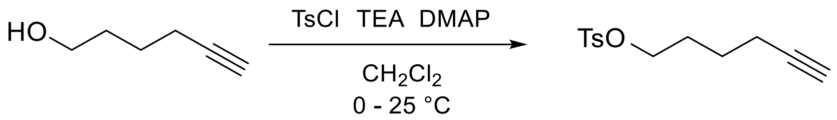

The compound was prepared from the commercially available hex-5-yn-1-ol (**20**), according to general procedure. The crude was purified by flash column chromatography using PE/EtOAc 9.5:0.5 and PE/EtOAc 9:1 as eluents to give a colorless oil; yield 40%; ^{1}H NMR (400 MHz, $CDCl_3$) δ 7.77 (d, J = 8.3 Hz, 2H), 7.34 (d, J = 8.0 Hz, 2H), 4.04 (t, J = 6.3 Hz, 2H), 2.44 (s, 3H), 2.15 (td, J = 7.0/2.6 Hz, 2H), 1.92 (t, J = 2.6 Hz, 1H), 1.76 (quint, J = 6.6 Hz, 2H), 1.54 (quint, J = 7.0 Hz, 2H) ppm; ^{13}C NMR (101 MHz, $CDCl_3$) δ 144.8, 133.0, 129.9, 127.8, 83.4, 70.0, 69.0, 27.7, 24.2, 21.6, 17.7 ppm.

Hept-6-yn-1-yl 4-methylbenzenesulfonate (34)

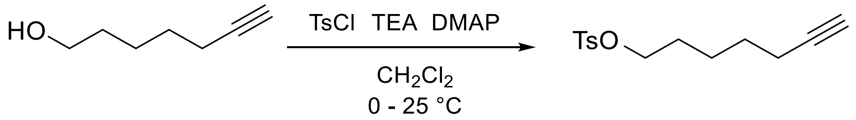

The compound was prepared from the commercially available hept-6-yn-1-ol (**21**), according to general procedure. The crude was purified by flash column chromatography using PE/EtOAc 9.5:0.5 and PE/EtOAc 9:1 as eluents to give a colorless oil; yield 94%; ^{1}H NMR (400 MHz, $CDCl_3$) δ 7.79 (d, J = 8.3 Hz, 2H), 7.35 (d, J = 8.0 Hz, 2H), 4.03 (t, J = 6.4 Hz, 2H), 2.45 (s, 3H), 2.15 (td, J = 6.7/2.6 Hz, 2H), 1.93 (t, J = 2.6 Hz, 1H), 1.67 (quint, J = 7.2 Hz, 2H), 1.50-1.42 (m, 4H) ppm; ^{13}C NMR (101 MHz, $CDCl_3$) δ 144.7, 133.1, 129.9, 127.9, 84.0, 70.4, 68.6, 28.3, 27.7, 24.5, 21.6, 18.2 ppm.

Oct-7-yn-1-yl 4-methylbenzenesulfonate (35)

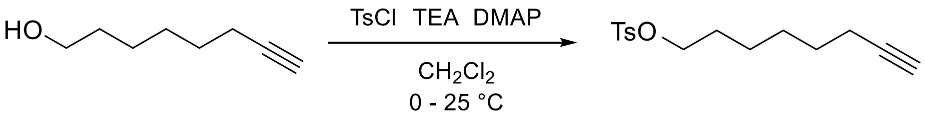

The compound was prepared from the commercially available ott-7-yn-1-ol (**22**), according to general procedure. The crude was purified by flash column chromatography using PE/EtOAc 9.5:0.5 and PE/EtOAc 9:1 as eluents to give a colorless oil; yield 80%; ^{1}H NMR (400 MHz, $CDCl_3$) δ 7.72 (d, J = 8.3 Hz, 2H), 7.30 (d, J = 8.1 Hz, 2H), 3.97 (t, J = 6.4 Hz, 2H), 2.39 (s, 3H), 2.07 (td, J = 6.9/2.5

Hz, 2H), 1.89 (t, J = 2.4 Hz, 1H), 1.58 (quint, J = 6.4 Hz, 2H), 1.40 (quint, J = 6.5 Hz, 2H), 1.37-1.28 (m, 4H) ppm; ^{13}C NMR (101 MHz, $CDCl_3$) δ 144.7, 133.1, 129.8, 127.8, 84.3, 70.5, 68.4, 28.6, 28.1, 27.9, 24.8, 21.5, 18.2 ppm.

Non-8-yn-1-yl 4-methylbenzenesulfonate (36)

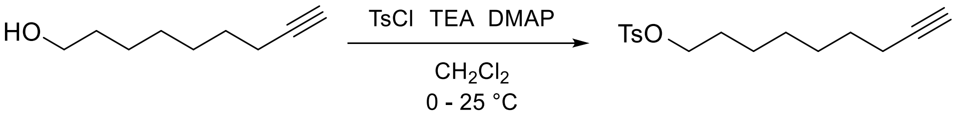

The compound was prepared from the commercially available non-8-yn-1-ol (**23**), according to general procedure. The crude was purified by flash column chromatography using PE/EtOAc 9.8:0.2 as eluent to give a colorless oil; yield 41%; ^{1}H NMR (400 MHz, $CDCl_3$) δ 7.78 (d, J = 8.1 Hz, 2H), 7.35 (d, J = 8.1 Hz, 2H), 4.01 (t, J = 6.4 Hz, 2H), 2.45 (s, 3H), 2.15 (td, J = 7.0/2.6 Hz, 2H), 1.93 (t, J = 2.6 Hz, 1H), 1.64 (quint, J = 6.5 Hz, 2H), 1.45 (quint, J = 6.9 Hz, 2H), 1.32-1.23 (m, 6H) ppm; ^{13}C NMR (101 MHz, $CDCl_3$) δ 144.7, 133.2, 129.8, 127.9, 84.5, 70.6, 68.2, 28.7, 28.4, 28.3, 28.2, 25.2, 21.6, 18.3 ppm.

Dec-9-yn-1-yl 4-methylbenzenesulfonate (37)

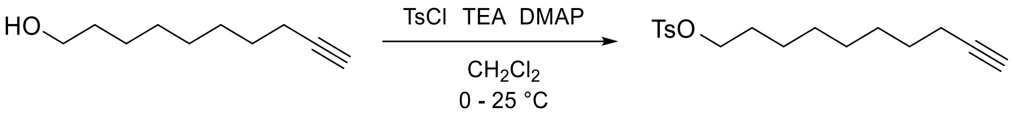

The compound was prepared from the commercially available dec-9-yn-1-ol (**24**), according to general procedure. The crude was purified by flash column chromatography using PE/EtOAc 9.8:0.2 as eluent to give a colorless oil; yield 23%; ^{1}H NMR (400 MHz, $CDCl_3$) δ 7.80 (d, J = 8.3 Hz, 2H), 7.33 (d, J = 8.0 Hz, 2H), 4.00 (t, J = 6.5 Hz, 2H), 2.43 (s, 3H), 2.15 (td, J = 7.0/2.6 Hz, 2H), 1.93 (t, J = 2.6 Hz, 1H), 1.60 (quint, J = 6.7 Hz, 2H), 1.48 (quint, J = 6.7 Hz, 2H), 1.36-1.20 (m, 8H) ppm; ^{13}C NMR (101 MHz, $CDCl_3$) δ 144.7, 133.2, 129.8, 127.8, 84.6, 70.6, 68.2, 28.8, 28.7 (2C), 28.5, 28.3, 25.2, 21.6, 18.3 ppm.

Undec-10-yn-1-yl 4-methylbenzenesulfonate (38)

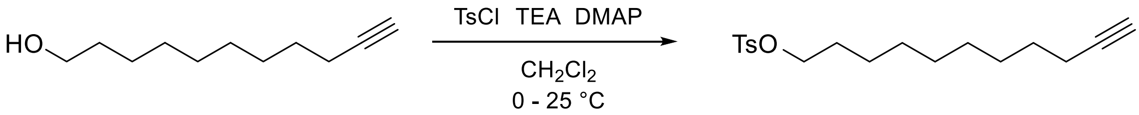

The compound was prepared from the commercially available undec-10-yn-1-ol (**25**), according to general procedure. The crude was purified by flash column chromatography using PE/EtOAc 9.8:0.2 as eluent to give a colorless oil; yield 72%; ^{1}H NMR (400 MHz, $CDCl_3$) δ 7.77 (d, J = 8.3 Hz, 2H),

7.34 (d, J = 8.0 Hz, 2H), 4.01 (t, J = 6.5 Hz, 2H), 2.44 (s, 3H), 2.16 (td, J = 7.0/2.6 Hz, 2H), 1.93 (t, J = 2.6 Hz, 1H), 1.62 (quint, J = 6.5 Hz, 2H), 1.50 (quint, J = 6.9 Hz, 2H), 1,37-1.23 (m, 10H) ppm; ^{13}C NMR (101 MHz, $CDCl_3$) δ 144.6, 133.2, 129.8, 127.8, 84.6, 70.7, 68.2, 29.2, 28.9, 28.8, 28.7, 28.6, 28.4, 25.3, 21.6, 18.3 ppm.

Dodec-11-yn-1-yl 4-methylbenzenesulfonate (39)

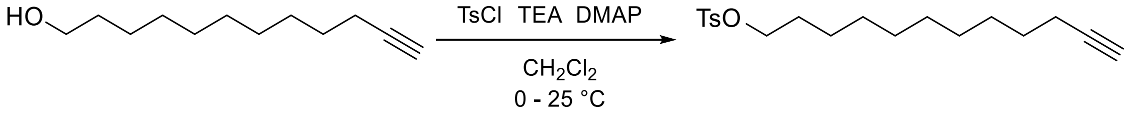

The compound was prepared from the commercially available dodec-11-yn-1-ol (**26**), according to general procedure. The crude was purified by flash column chromatography using PE/EtOAc 9.8:0.2 as eluent to give a colorless oil; yield 73%; ^{1}H NMR (400 MHz, $CDCl_3$) δ 7.73 (d, J = 8.3 Hz, 2H), 7.30 (d, J = 8.1 Hz, 2H), 3.97 (t, J = 6.5 Hz, 2H), 2,40 (s, 3H), 2.12 (td, J = 7.0/2.7 Hz, 2H), 1.90 (t, J = 2.6 Hz, 1H), 1.58 (quint, J = 6.7 Hz, 2H), 1.46 (quint, J = 7.5 Hz, 2H), 1.34-1.18 (m, 12H) ppm; ^{13}C NMR (101 MHz, $CDCl_3$) δ 144.6, 133.2, 129.8, 127.8, 84.6, 70.6, 68.2, 29.3 (2C), 29.0, 28.8, 28.7, 28.6, 28.4, 25.3, 21.5, 18.3 ppm.

General procedure for the tosylation of the alkyne functionalized polyethylene glycols (40-43)

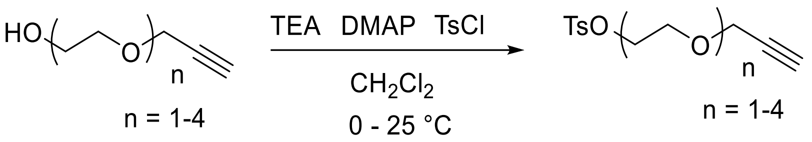

TEA (1.5 equiv) and DMAP (0.05 equiv) were added to a solution of the corresponding alkyne (**27-30**) (1 equiv) in anhydrous CH_2Cl_2 (0.45 M). The solution was cooled to 0 °C, then TsCl (1.2 equiv) was added portionwise and the reaction was stirred overnight under nitrogen atmosphere at room temperature. Upon completion, the reaction was diluted with water and extracted with CH_2Cl_2 (x2). The combined organic layers were then washed with a saturated $NaHCO_3$ aqueous solution (x1), 2 M HCl aqueous solution (x1) and brine (x1). The organic layer was dried over anhydrous Na_2SO_4, filtered and the solvent was evaporated under vacuum. The crude was purified by column chromatography.

2-(Prop-2-yn-1-yloxy)ethyl 4-methylbenzenesulfonate (40)

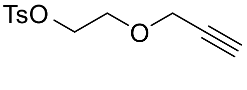

The compound was prepared from the commercially available 2-(prop-2-yn-1-yloxy)ethanol (**27**) according to general procedure. The crude was purified using PE/EtOAc 9.5:0.5 and PE/EtOAc 8:2 as eluents to give a yellow oil; yield 56%; ^{1}H NMR (400 MHz $CDCl_3$) δ 7.80 (d, J = 8.4 Hz, 2H), 7.35 (d, J = 8.0 Hz, 2H), 4.19 (t, J = 4.6 Hz, 2H), 4.11 (d, J = 2.4 Hz, 2H), 3.72 (t, J = 4.6 Hz, 2H), 2.44 (m, 4H) ppm; ^{13}C NMR (101 MHz, $CDCl_3$) δ 145.0, 132.8, 129.9, 127.9, 79.0, 75.1, 69.0, 67.1, 58.3, 21.6 ppm.

2-(2-(Prop-2-yn-1-yloxy)ethoxy)ethyl 4-methylbenzenesulfonate (41)

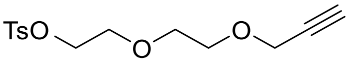

The compound was prepared from the commercially available 2-(2-(prop-2-yn-1-yloxy)ethoxy)ethanol (**28**) according to general procedure. The crude was purified using PE/EtOAc 95:5 and PE/EtOAc 8:2 as eluents to give a yellow oil; yield 99%; ^{1}H NMR (400 MHz, $CDCl_3$) δ 7.77 (d, J = 8.4 Hz, 2H), 7.33 (d, J = 8.5 Hz, 2H), 4.15-4.12 (m, 4H), 3.67-3.65 (m, 2H), 3.61-3.56 (m, 4H), 2.44-2.42 (m, 4H) ppm; ^{13}C NMR (101 MHz, $CDCl_3$) δ 144.9, 132.9, 129.9, 127.9, 79.5, 74.7, 70.5, 69.3, 69.0, 68.6, 58.4, 21.6 ppm.

2-(2-(2-(Prop-2-yn-1-yloxy)ethoxy)ethoxy)ethyl 4-methylbenzenesulfonate (42)

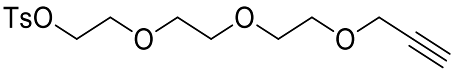

The compound was prepared from the commercially available 2-(2-(2-(prop-2-yn-1-yloxy)ethoxy)ethoxy)ethanol (**29**) according to general procedure. The crude was purified using PE/EtOAc 7:3 and PE/EtOAc 6:4 as eluents to give a yellow oil; yield 69%; ^{1}H NMR (400 MHz, $CDCl_3$) δ 7.80 (d, J = 6.7 Hz, 2H), 7.34 (d, J = 8.0 Hz, 2H), 4.18 (d, J =2.4 Hz, 2H), 4.16 (t, J = 4.4 Hz, 2H), 3.70-3.59 (m, 10H), 2.44-2.43 (m, 4H) ppm; ^{13}C NMR (101 MHz, $CDCl_3$) δ 144.8, 133.0, 129.8, 128.0, 79.6, 74.6, 70.7, 70.5, 70.4, 69.3, 69.1, 68.7, 58.4. 21.6 ppm.

3,6,9,12-Tetraoxapentadec-14-yn-1-yl 4-methylbenzenesulfonate (43)

The compound was prepared from the commercially available 3,6,9,12-tetraoxapentadec-14-yn-1-ol (**30**) according to general procedure. The crude was purified by using PE/EtOAc 7:3 and PE/EtOAc 5:5 as eluents to give a yellow oil; yield 93%; ^{1}H NMR (400 MHz, $CDCl_3$) δ 7.80 (d, J = 8.3 Hz, 2H), 7.35 (d, J = 8.0 Hz, 2H), 4.20 (d, J = 2.4 Hz, 2H), 4.16 (t, J = 3.9 Hz, 2H), 3.70-3.59 (m, 14H), 2.45 (s, 3H), 2.44 (t, J = 2.4 Hz, 1H) ppm; ^{13}C NMR (101 MHz, $CDCl_3$) δ 144.8, 133.0, 130.0, 128.0, 79.7, 74.6, 70.7, 70.6 (2C), 70.5, 70.4, 69.3, 69.1, 68.7, 58.4, 21.6 ppm.

^{1}H-NMR, ^{13}C-NMR data of the intermediates synthesized by Williamson etherification (44-52, 62)

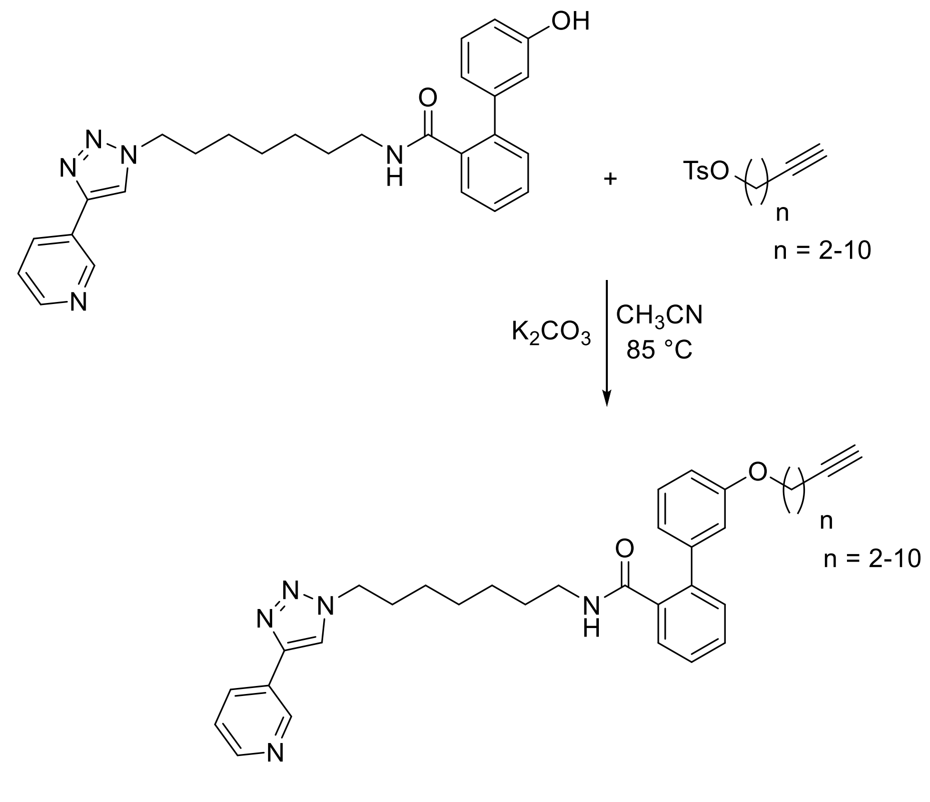

3'-(But-3-yn-1-yloxy)-*N*-(7-(4-(pyridin-3-yl)-1*H*-1,2,3-triazol-1-yl)heptyl)-[1,1'-biphenyl]-2-carboxamide (44)

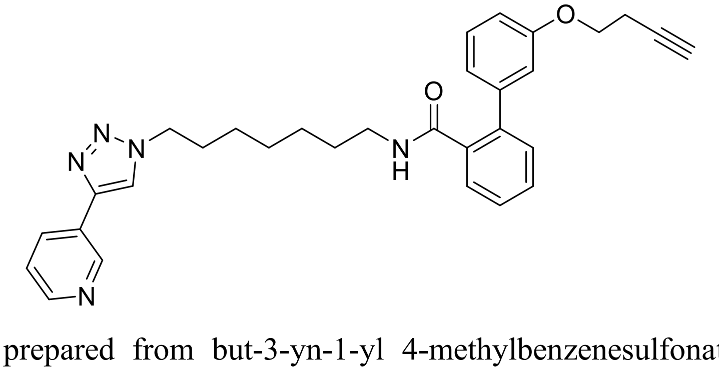

The compound was prepared from but-3-yn-1-yl 4-methylbenzenesulfonate (**31**), according to general procedure A. The crude was purified using CH_2Cl_2/MeOH 99:1 as eluent to give a pale yellow

oil; yield 33%; ^{1}H NMR (400 MHz, $CDCl_3$) δ 9.05 (br s, 1H), 8.59 (d, J = 4.8 Hz, 1H), 8.30 (dt, J = 8.0/1.9 Hz, 1H), 7.94 (s, 1H), 7.70 (dt, J = 6.2/1.8 Hz, 1H), 7.50 – 7.29 (m, 5H), 7.02-6.87 (m, 3H), 5.32 (t, J = 5.7 Hz, 1H), 4.43 (t, J = 7.3 Hz, 2H), 4.10 (t, J = 6.9 Hz, 2H), 3.15 (q, J = 6.7 Hz, 2H), 2.69 (td, J = 6.9/2.7 Hz, 2H), 2.05 (t, J = 2.7 Hz, 1H), 1.94 (quint, J = 7.0 Hz, 2H), 1.32-1.18 (m, 6H), 1.09-1.00 (m, 2H) ppm; ^{13}C NMR (101 MHz, $CDCl_3$) δ 169.4, 158.6, 148.2, 146.1, 144.3, 141.8, 139.1, 135.8, 133.8, 132.1, 130.0, 129.7, 128.8, 127.8, 127.3, 124.1, 121.5, 120.2, 114.8, 114.4, 80.4, 70.0, 66.1, 50.5, 39.7, 30.2, 28.9, 28.5, 26.4, 26.2, 19.6 ppm.

3'-(Pent-4-yn-1-yloxy)-*N*-(7-(4-(pyridin-3-yl)-1*H*-1,2,3-triazol-1-yl)heptyl)-[1,1'-biphenyl]-2-carboxamide (45)

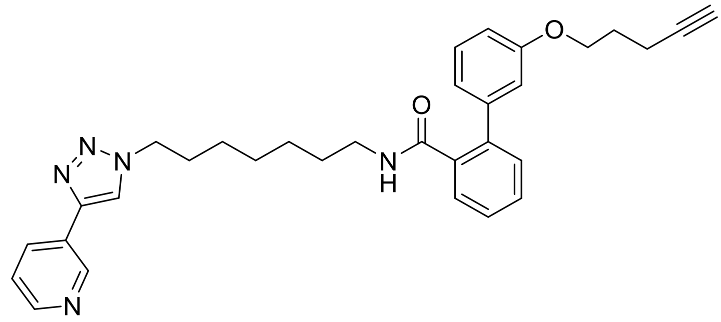

The compound was prepared from pent-4-yn-1-yl 4- methylbenzenesulfonate (**32**), according to general procedure A. The crude was purified using CH_2Cl_2/MeOH 98:2 and CH_2Cl_2/MeOH 97:3 as eluents to give a pale yellow oil; yield 90%; ^{1}H NMR (400 MHz, $CDCl_3$) δ 9.01 (br s, 1H), 8.56 (br d, 1H), 8.22 (dt, J = 8.0/1.8 Hz, 1H), 7.9 (s, 1H), 7.68 (dd, J = 7.5/1.3 Hz, 1H), 7.48-7.33 (m, 4H), 7.28 (t, J = 8.4 Hz, 1H), 6.98-6.88 (m, 3H), 5.37 (t, J = 5.6 Hz, 1H), 4.41 (t, J = 7.1 Hz, 2H), 4.07 (t, J = 6.1 Hz, 2H), 3.15 (q, J = 6.1 Hz, 2H), 2.40 (td, J = 7.0/2.6 Hz, 2H), 2.04-1.90 (m, 5H), 1.31-1.17 (m, 6H), 1.04 (quint, J = 7.1 Hz, 2H) ppm; ^{13}C NMR (101 MHz, $CDCl_3$) δ 169.4, 159.0, 148.8, 146.7, 144.5, 141.7, 139.2, 135.8, 133.2, 130.0, 130.0, 129.7, 128.8, 127.7, 127.0, 123.9, 121.1, 120.0, 114.7, 114.1, 83.4, 69.0, 66.2, 50.5, 39.6, 30.2, 28.9, 28.5, 28.2, 26.4, 26.2, 15.2 ppm.

3'-(Hex-5-yn-1-yloxy)-*N*-(7-(4-(pyridin-3-yl)-1*H*-1,2,3-triazol-1-yl)heptyl)-[1,1'-biphenyl]-2-carboxamide (46)

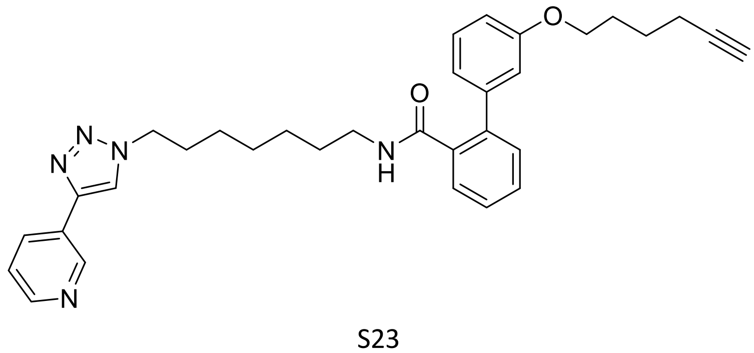

The compound was prepared from hex-5-yn-1-yl 4- methylbenzenesulfonate (**33**), according to general procedure A. The crude was purified using CH_2Cl_2/MeOH 98:2 and CH_2Cl_2/MeOH 95:5 as eluents to give a pale yellow oil; yield 69%; ^{1}H NMR (400 MHz, $CDCl_3$) δ 9.00 (br s, 1H), 8.55 (br d, 1H), 8.22 (dt, J = 8.0/1.9 Hz, 1H), 7.91 (s, 1H), 7.69 (dd, J = 7.4/1.2 Hz, 1H), 7.47-7.26 (m, 5H), 6.97-6.88 (m, 3H); 5.37 (t, J = 5.2 Hz, 1H), 4.41 (t, J = 7.2 Hz, 2H), 3.98 (t, J = 6.2 Hz, 2H), 3.14 (q, J = 6.1 Hz, 2H), 2.27 (td, J = 7.1/2.6 Hz, 2H), 1.97 (t, J = 2.6 Hz, 1H), 1.95-1.87 (m, 4H), 1.71 (quint, J = 7.8 Hz, 2H), 1.31-1.17 (m, 6H), 1.04 (quint, J = 7.0 Hz, 2H) ppm; ^{13}C NMR (101 MHz, $CDCl_3$) δ 169.4, 159.1, 148.8, 146.7, 144.5, 141.7, 139.2, 135.8, 133.2, 130.0, 130.0, 129.6, 128.8, 127.7, 127.0, 123.9, 121.0, 120.0, 114.6, 114.1, 84.0, 68.8, 67.4, 50.5, 39.6, 30.2, 28.9, 28.5, 28.3, 26.4, 26.2, 25.1, 18.2 ppm.

3'-(Hept-6-yn-1-yloxy)-*N*-(7-(4-(pyridin-3-yl)-1*H*-1,2,3-triazol-1-yl)heptyl)-[1,1'-biphenyl]-2-carboxamide (47)

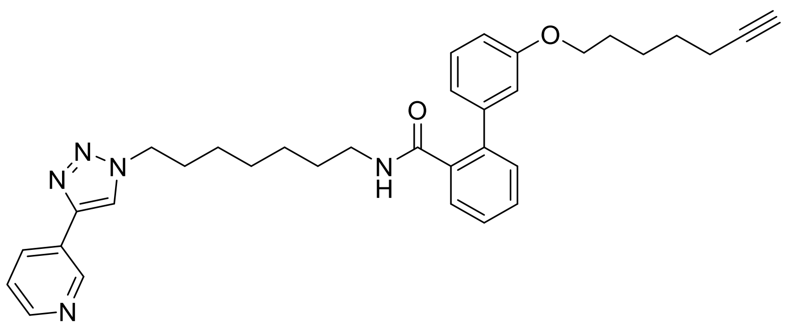

The compound was prepared from hept-6-yn-1-yl 4-methylbenzenesulfonate (**34**) according to general procedure A. The crude was purified using EtOAc as eluent to give a pale yellow oil; yield 64%; ^{1}H NMR (400 MHz, $CDCl_3$) δ 8.97 (s, 1H), 8.50 (d, J = 4.2 Hz, 1H), 8.17 (d, J = 7.9 Hz, 1H), 7.89 (s, 1H), 7.65 (d, J = 7.0 Hz, 1H); 7.44 7.28 (m, 4H), 7.25 (t, J = 7.8 Hz, 1H), 6.93-6.89 (m, 2H), 6.85 (d, J = 8.2 Hz, 1H), 5.46 (t, J = 5.5 Hz, 1H), 4.38 (t, J = 7.1 Hz, 2H), 3.93 (t, J = 6.4 Hz, 2H), 3.12 (q, J = 4.4 Hz, 2H), 2.19 (t, J = 3.5 Hz, 2H), 1.94-1.87 (m, 3H), 1.76 (t, J = 6.5 Hz, 2H), 1.57-1.53 (m, 4H), 1.25-1.15 (m, 6H), 1.05-0.99 (m, 2H) ppm; ^{13}C NMR (101 MHz, $CDCl_3$) δ 169.4, 159.1, 148.9, 146.8, 144.5, 141.7, 139.2, 135.9, 133.0, 130.0, 129.9, 129.6, 128.7, 127.6, 126.9, 123.8, 120.9, 120.0, 114.6, 114.1, 84.29, 68.5, 67.8, 50.4, 39.6, 30.2, 28.8, 28.7, 28.5, 28.1, 26.4, 26.2, 25.2, 18.3 ppm.

3'-(Oct-7-yn-1-yloxy)-*N*-(7-(4-(pyridin-3-yl)-1*H*-1,2,3-triazol-1-yl)heptyl)-[1,1'-biphenyl]-2-carboxamide (48)

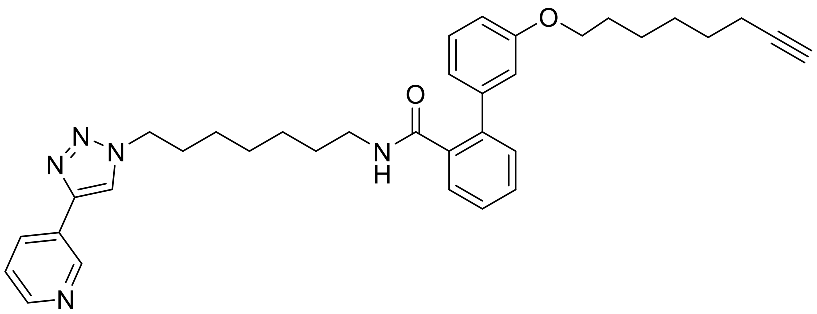

The compound was prepared from oct-7-yn-1-yl 4-methylbenzenesulfonate (**35**) according to general procedure A. The crude was purified using EtOAc as eluent to give a pale yellow oil; yield 79%; ^{1}H NMR (400 MHz, $CDCl_3$) δ 9.02 (s, 1H), 8.58 (d, J = 4.7 Hz, 1H), 8.25 (dt, J = 8.0/2.0 Hz, 1H), 7.91 (s, 1H); 7.71 (dd, J = 7.4/1.2 Hz, 1H), 7.49-7.27 (m, 5H), 6.98-6.88 (m, 3H), 5.31 (t, J = 4.2 Hz, 1H), 4.43 (t, J = 7.2 Hz, 2H), 3.96 (t, J = 6.4 Hz, 2H), 3.15 (q, J = 6.0 Hz, 2H), 2.21 (td, J = 6.9/2.6 Hz, 2H), 1.96-1.93 (m, 3H), 1.80 (quint, J = 6.6 Hz, 2H), 1.58-1.46 (m, 6H), 1.34-1.17 (m, 6H), 1.05 (quint, J = 7.0 Hz, 2H) ppm; ^{13}C NMR (101 MHz, $CDCl_3$) δ 169.3, 159.2, 148.7, 146.6, 144.5, 141.7, 139.3, 135.8, 133.3, 130.1, 130.0, 129.6, 128.8, 127.7, 127.1, 123.9, 120.9, 119.9, 114.6, 114.2, 84.5, 68.3, 67.9, 50.5, 39.6, 30.2, 29.1, 28.9, 28.5, 28.5, 28.4, 26.4, 26.2, 25.6, 18.3 ppm.

3'-(Non-8-yn-1-yloxy)-*N*-(7-(4-(pyridin-3-yl)-1*H*-1,2,3-triazol-1-yl)heptyl)-[1,1'-biphenyl]-2-carboxamide (49)

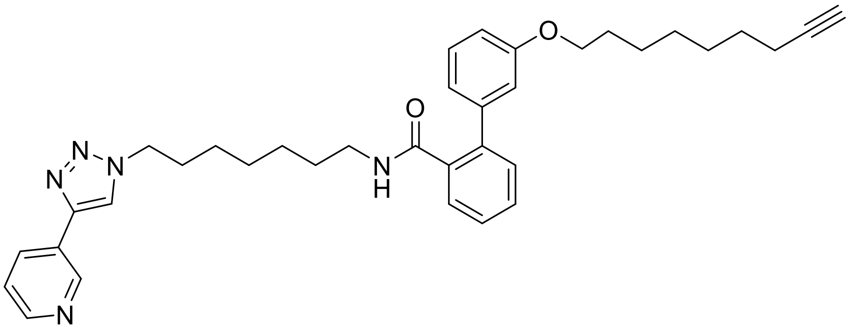

The compound was prepared from non-8-yn-1-yl 4-methylbenzenesulfonate (**36**) according to general procedure A. The crude was purified using PE/EtOAc 1:9 as eluent to give a pale yellow oil; yield 43%; ^{1}H NMR (400 MHz, $CDCl_3$) δ 9.00 (s, 1H), 8.65 (d, J = 4.7 Hz, 1H), 8.22 (dt, J = 8.0/1.9 Hz, 1H), 7.90 (s, 1H), 7.68 (dd, J = 7.4/1.4 Hz, 1H), 7.47-7.25 (m, 5H), 6.96-6.86 (m, 3H), 5.35 (t, J = 5.2 Hz, 1H), 4.41 (t, J = 7.2 Hz, 2H), 3.95 (t, J = 6.5 Hz, 2H), 3.14 (q, J = 6.2 Hz, 2H), 2.20-2.13 (m, 2H), 1.96-1.91 (m, 3H), 1.89-1.76 (m, 2H), 1.55-1.39 (m, 8H), 1.38-1.22 (m, 6H), 1.20-1.1 (m, 2H) ppm; ^{13}C NMR (101 MHz, $CDCl_3$) δ 169.4, 159.2, 148.9, 146.8, 144.6, 141.7, 139.3, 135.8, 133.2, 130.0, 129.9, 129.6, 128.8, 127.7, 127.0, 123.8, 120.9, 120.0, 114.6, 114.2, 84.6, 68.2, 68.0, 50.4, 39.6, 30.2, 29.2, 29.0, 28.9, 26.6, 28.5, 28.3, 26.4, 26.2, 25.9, 18.4 ppm.

3'-(Dec-9-yn-1-yloxy)-*N*-(7-(4-(pyridin-3-yl)-1*H*-1,2,3-triazol-1-yl)heptyl)-[1,1'-biphenyl]-2-carboxamide (50)

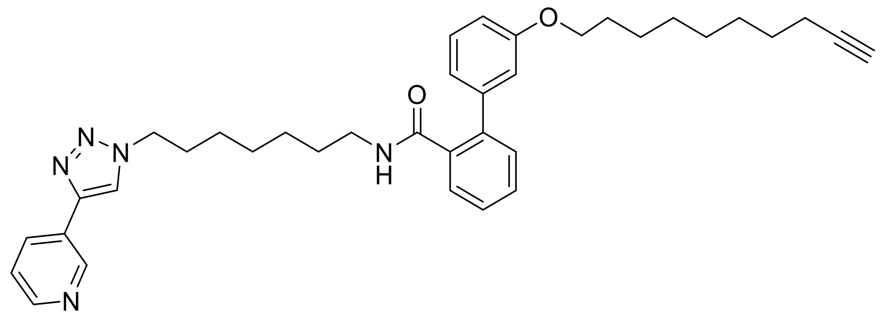

The compound was prepared from dec-9-yn-1-yl 4-methylbenzenesulfonate (**37**) according to general procedure A. The crude was purified using PE/EtOAc 2:8 as eluent to give a pale yellow oil; yield 54%; ^{1}H NMR (400 MHz, $CDCl_3$) δ 9.03 (br s, 1H), 8.57 (br s, 1H), 8.21 (d, J = 7.9 Hz, 1H), 7.90 (s, 1H), 7.68 (dd, J = 7.5/2.5 Hz, 1H), 7.47-7.25 (m, 5H), 6.95-6.86 (m, 3H), 5.38 (t, J = 5.6 Hz, 1H), 4.40 (t, J = 7.2 Hz, 2H), 3.93 (t, J = 6.5 Hz, 2H), 3.13 (q, J = 6.2 Hz, 2H), 2,17 (td, J = 7.0/2.6 Hz, 2H), 1.96-1.89 (m, 3H), 1.76 (quint, J = 6.6 Hz, 2H), 1.51 (quint, J = 7.6 Hz, 2H), 1.46-1.23 (m, 12H), 1.20 (quint, J = 7.6 Hz, 2H), 1.03 (quint, J = 7.4 Hz, 2H) ppm; ^{13}C NMR (101 MHz, $CDCl_3$) δ 169.4, 159.2, 148.8, 146.7, 144.6, 141.7, 139.3, 135.8, 133.1, 130.0, 129.9, 129.6, 128.8, 127.7, 127.3, 123.8, 120.9, 120.0, 114.6, 111.2, 84.7, 68.2, 68.0, 50.4, 39.6, 30.2, 29.2 (2C), 29.0, 28.9, 28.6, 28.5, 28.4, 26.4, 26.2, 26.0, 18.4 ppm.

3'-(Undec-10-yn-1-yloxy)-*N*-(7-(4-(pyridin-3-yl)-1*H*-1,2,3-triazol-1-yl)heptyl)-[1,1'-biphenyl]-2-carboxamide (51)

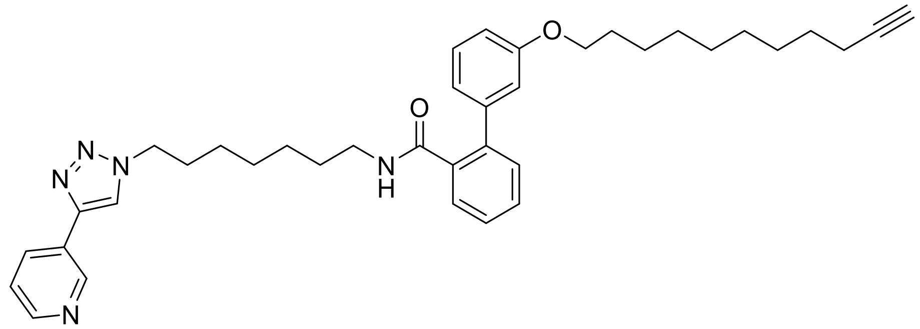

The compound was prepared from unced-10-yn-1-yl 4-methylbenzenesulfonate (**38**) according to general procedure A. The crude was purified using PE/EtOAc 3:7 and PE/EtOAc 1:9 as eluents to give a pale yellow oil; yield 58%; ^{1}H NMR (400 MHz, $CDCl_3$) δ 8.98 (br s, 1H), 8.52 (d, J = 4.4 Hz, 1H), 8.18 (d, J = 6.6 Hz, 1H), 7.9 (s, 1H), 7.66 (d, J = 7.9 Hz, 1H), 7.40-7.28 (m, 4H), 7.25 (t, J = 7.8 Hz, 1H), 6.94-6.84 (m, 3H), 5.43 (br s, 1H), 4.39 (t, J = 7.2 Hz, 2H), 3.92 (t, J = 6.5 Hz, 2H), 3.00 (q,

J = 6.2 Hz, 2H), 2.15 (td, J = 6.6/2.6 Hz, 2H), 1.94-1.89 (m, 3H), 1.75 (quint, J = 7.1 Hz, 2H), 1.50 (quint, J = 7.1 Hz, 2H), 1.42-1.24 (m, 14H), 1.19 (quint, J = 7.3 Hz, 2H), 1.01 (quint, J = 6.9 Hz, 2H) ppm; ^{13}C NMR (101 MHz, $CDCl_3$) δ 169.4, 159.2, 149.0, 146.8, 144.6, 141.7, 139.3, 135.6, 133.0, 130.0, 129.9, 129.6, 128.8, 127.6, 127.0, 123.8, 120.8, 120.0, 114.6, 114.1, 84.8, 68.2, 68.0, 50.4, 39.6, 30.2, 29.4, 29.3, 29.2, 29.0, 28.8, 28.7, 28.5, 28.4, 26.4, 26.2, 26.0, 18.4 ppm.

3'-(Dodec-11-yn-1-yloxy)-*N*-(7-(4-(pyridin-3-yl)-1*H*-1,2,3-triazol-1-yl)heptyl)-[1,1'-biphenyl]-2-carboxamide (52)

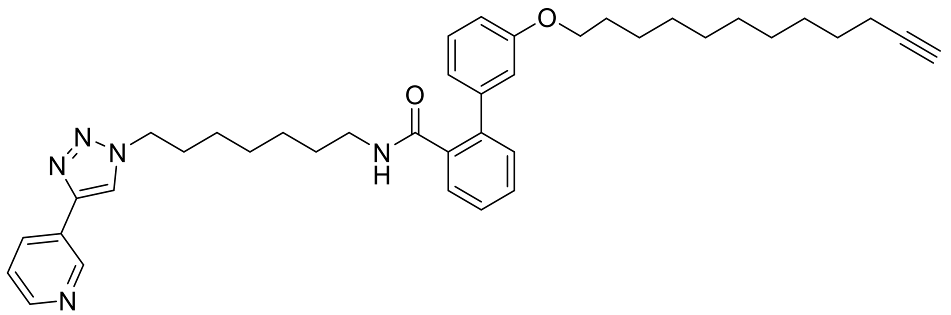

The compound was prepared from dodec-11-yn-1-yl 4-methylbenzenesulfonate (**39**) according to general procedure A. The crude was purified using PE/EtOAc 3:7 and PE/EtOAc 1:9 as eluents to give a pale yellow oil; yield 63%; ^{1}H NMR (400 MHz, $CDCl_3$) δ 8.99 (br s, 1H), 8.54, (br s, 1H), 8.24-8.20 (m, 1H), 7.89 (s, 1H), 7.69-7.67 (m, 1H), 7.46-7.24 (m, 5H), 6.95-6.88 (m, 3H), 5.83 (br s, 1H), 4.40 (t, J = 6.7 Hz, 2H), 3.93 (t, J = 6.3 Hz, 2H), 3.13 (q, J = 6.6 Hz, 2H), 2.18-2.15 (m, 2H), 1.93-1.91 (m, 3H), 1.76 (quint, J = 6.6 Hz, 2H), 1.54-1.18 (m, 20H), 1.16-1.03 (m, 2H) ppm; ^{13}C NMR (101 MHz, $CDCl_3$) δ 169.4, 159.2, 148.9, 146.8, 144.6, 141.7, 139.3, 135.8, 133.1, 130.0, 129.9, 129.6, 128.8, 127.6, 127.0, 123.8, 120.9, 120.0, 114.6, 114.2, 84.8, 68.1, 68.0, 50.4, 39.8, 30.2, 29.5, 29.4, 29.3, 29.2, 29.1, 28.9, 28.7, 28.5, 28.4, 26.4, 26.2, 26.0, 18.4 ppm.

3'-(prop-2-yn-1-yloxy)-*N*-(7-(4-(pyridin-3-yl)-1*H*-1,2,3-triazol-1-yl)heptyl)-[1,1'-biphenyl]-2-carboxamide (62)

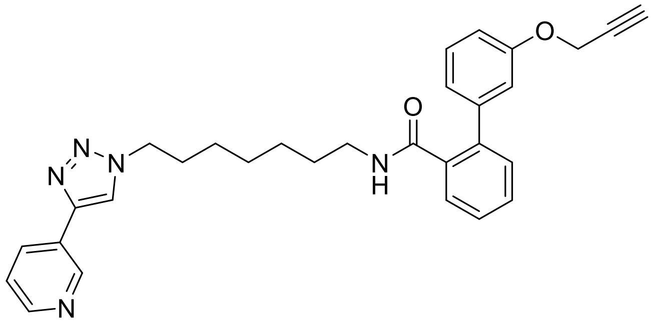

The compound was prepared from propargyl bromide, according to general procedure A. The crude was purified using CH_2Cl_2/MeOH 99:1 and CH_2Cl_2/MeOH 95:5 as eluents to give a yellow oil; yield

13 %; ^{1}H NMR (400 MHz, $CDCl_3$) δ 9.01 (s, 1H), 8.57 (d, J = 2.8 Hz, 1H), 8.22 (d, J = 7.9 Hz, 1H), 7.89 (s, 1H), 7.70 (dd, J = 7.4/1.2 Hz, 1H), 7.49-7.28 (m, 5H), 7.04-6.96 (m, 3H), 5.31 (br s, 1H), 4.71 (d, J = 2.3 Hz, 2H), 4.43 (t, J = 7.2 Hz, 2H), 3.15 (q, J = 6.1 Hz, 2H), 2.55 (t, J = 2.2 Hz, 1H), 1.94 (quint, J = 7.0 Hz, 2H), 1.29-1.17 (m, 6H), 1.06 (quint, J =7.4 Hz, 2H) ppm; ^{13}C NMR (101 MHz, $CDCl_3$) δ 169.3, 157.7, 149.1, 146.9, 144.6, 141.8, 139.0, 135.8, 133.0, 130.0 (2C), 129.7, 128.8, 127.8, 126.9, 123.8, 122.0, 119.9, 115.0, 114.4, 78.3, 75.8, 55.9, 50.5, 39.7, 30.2, 28.9, 28.5, 26.4, 26.2 ppm.

^{1}H-NMR, ^{13}C-NMR data of the intermediates synthesized by Williamson etherification (53-56)

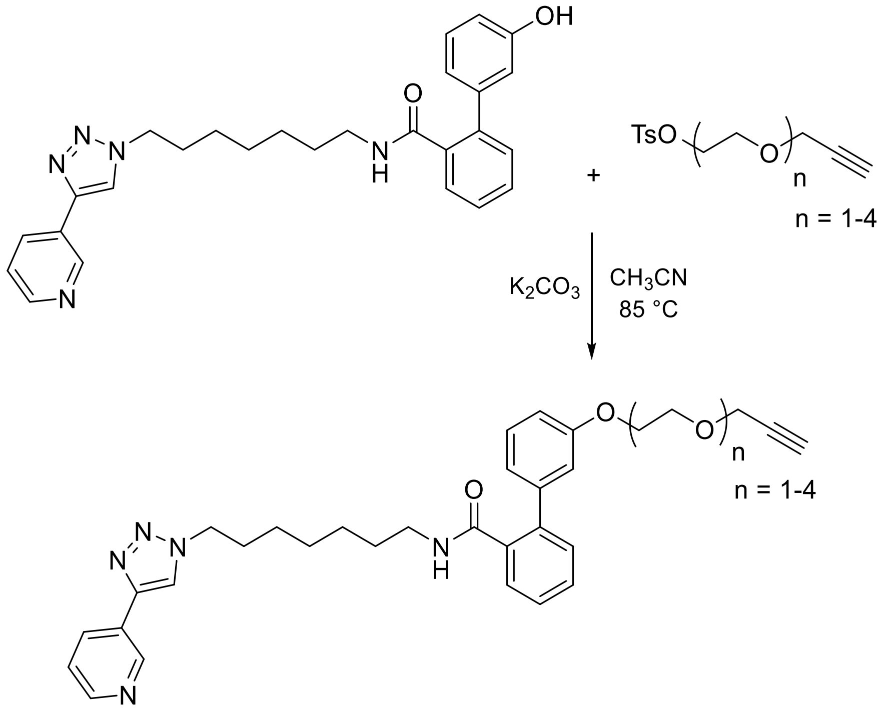

The compounds **53-56** were synthesized from the corresponding tosylated polyethylene glycol alkynes (**40-43**) and MV-78-OH (**8**) according to general procedure A

3'-(2-(Prop-2-yn-1-yloxy)ethoxy)-*N*-(7-(4-(pyridin-3-yl)-1*H*-1,2,3-triazol-1-yl)heptyl)-[1,1'-biphenyl]-2-carboxamide (53)

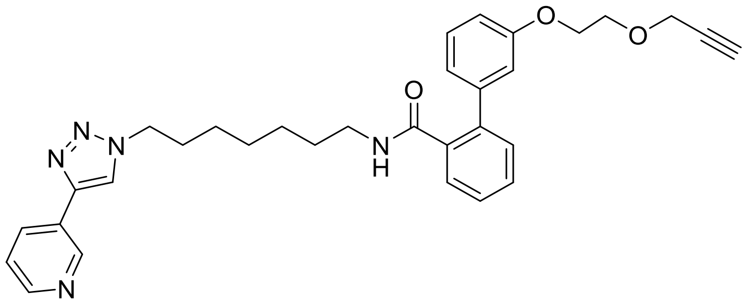

The compound was prepared from 2-(prop-2-yn-1-yloxy)ethyl 4-methylbenzenesulfonate (**40**), according to general procedure A. The crude was purified using CH_2Cl_2/MeOH 98:2 as eluent. Yellow oil; yield 71%; ^{1}H NMR (400 MHz, $CDCl_3$) δ 9.00 (s, 1H), 8.54 (d, *J* = 4.6 Hz, 1H), 8.21 (dd, *J* = 7.8/1.7 Hz, 1H), 7.92 (s, 1H), 7.67 (d, *J* = 7.4 Hz, 1H), 7.46-7.25 (m, 5H), 6.98-6.95 (m, 2H), 6.50 (dd, *J* = 8.3/2.4 Hz, 1H), 5.38 (br s, 1H), 4.40 (t, *J* = 7.1 Hz, 2H), 4.25 (d, *J* = 2.4 Hz, 2H), 4.14 (t, *J* = 4.4 Hz, 2H), 3.89 (t, *J* = 4.9 Hz, 2H), 3.14 (q, *J* = 6.4 Hz, 2H), 2.46 (t, *J* = 2.4 Hz, 1H), 1.92 (quint, *J* = 7.1 Hz, 2H), 1.27-1.16 (m, 6H), 1.02 (quint, *J* =7.1 Hz, 2H) ppm; ^{13}C NMR (101 MHz, $CDCl_3$) δ 169.4, 158.8, 148.9, 146.8, 144.5, 141.7 139.1, 135.8, 133.1, 130.0, 129.9, 129.6, 128.8, 127.7, 127.0, 123.8, 121.4, 120.1, 114.7, 114.2, 79.4, 74.9, 68.2, 67.3, 58.6, 50.4, 39.6, 30.2, 28.8, 28.5, 26.4, 26.2 ppm.

3'-(2-(2-(Prop-2-yn-1-yloxy)ethoxy)ethoxy)-*N*-(7-(4-(pyridin-3-yl)-1*H*-1,2,3-triazol-1-yl)heptyl)-[1,1'-biphenyl]-2-carboxamide (54)

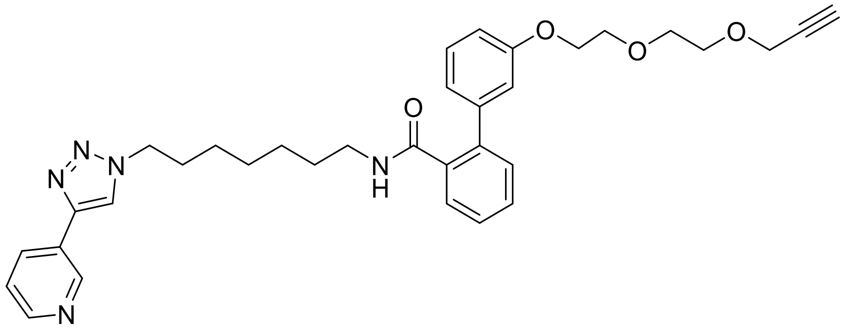

The compound was prepared from 2-(2-(prop-2-yn-1-yloxy)ethoxy)ethyl 4-methylbenzenesulfonate (**41**), according to general procedure A. The crude was purified using CH_2Cl_2/MeOH 98:2 as eluent. Yellow oil; yield 63%; ^{1}H NMR (400 MHz, $CDCl_3$) δ 9.04 (d, *J* = 1.5 Hz, 1H), 8.58 (dd, *J* = 4.8/1.4 Hz, 1H), 8.28 (dt, *J* = 8.1/1.8 Hz, 1H), 7.95 (s, 1H), 7.70 (dd, *J* = 7.4/1.4 Hz, 1H), 7.49-7.27 (m, 5H), 7.00-6.96 (m, 2H), 6.92-6.91 (m, 1H), 5.29 (t, *J* = 5.7 Hz, 1H), 4.43 (t, *J* = 7.2 Hz, 2H), 4.20 (d, *J* = 2.4 Hz, 2H), 4.15 (t, *J* = 4.6 Hz, 2H), 3.88 (t, *J* = 4.6 Hz, 2H), 3.78-3.72 (m, 4H), 3.15 (q, *J* = 6.9 Hz, 2H), 2.44 (t, *J* = 2.4 Hz, 1H), 1.95 (quint, *J* = 7.1 Hz, 2H), 1.32-1-18 (m, 6H), 1.05 (quint, *J* = 7.1 Hz, 2H) ppm; ^{13}C NMR (101 MHz, $CDCl_3$) δ 169.4, 159.0, 148.5, 146.4, 144.4, 141.7, 139.1, 135.8, 133.5, 130.1, 130.0, 129.6, 128.8, 127.7, 127.2, 124.0, 121.4, 120.1, 114.8, 114.2, 79.6, 74.6, 70.7, 69.8, 69.1, 67.5, 58.4, 50.5, 39.7, 30.2, 28.9, 28.5, 26.4, 26.2 ppm.

3'-(2-(2-(2-(Prop-2-yn-1-yloxy)ethoxy)ethoxy)ethoxy)-*N*-(7-(4-(pyridin-3-yl)-1*H*-1,2,3-triazol-1-yl)heptyl)-[1,1'-biphenyl]-2-carboxamide (55)

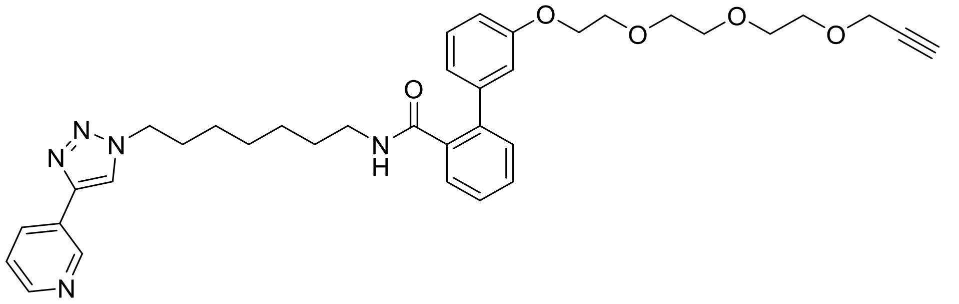

The compound was prepared from 2-(2-(2-(prop-2-yn-1-yloxy)ethoxy)ethoxy)ethyl 4-methylbenzenesulfonate (**42**), according to general procedure A. The crude was purified using CH_2Cl_2/MeOH 98:2 as eluent. Yellow oil; yield 37%; ^{1}H NMR (400 MHz, $CDCl_3$) δ 9.06 (s, 1H), 8.58 (s, 1H), 8.31 (d, J = 7.9 Hz, 1H), 7.98 (s, 1H), 7.70 (d, J = 7.5 Hz, 1H), 7.49-7.27 (m, 5H), 7.00-6.96 (m, 2H), 6.91 (d, J = 8.1 Hz, 1H), 5.29 (t, J = 5.3 Hz, 1H), 4.44 (t, J = 7.1 Hz, 2H), 4.19 (d, J = 2.2 Hz, 2H), 4.14 (t, J = 5.0 Hz, 2H), 3.87 (t, J = 4.8 Hz, 2H), 3.76-3.69 (m, 8H), 3.16 (q, J = 6.6 Hz, 2H), 2.43 (t, J = 2.2 Hz, 1H), 1.95 (quint, J = 6.8 Hz, 2H), 1.30-1.18 (m, 6H), 1.07 (quint, J = 7.4 Hz, 2H) ppm; ^{13}C NMR (101 MHz, $CDCl_3$) δ 171.4, 158.8, 148.0, 145.8, 143.9, 141.7, 139.5, 136.6, 133.6, 129.7, 129.4, 129.0, 127.4, 127.3, 127.0, 124.2, 121.7, 121.1, 114.9, 113.7, 78.6, 74.4, 70.4, 70.2, 70.0, 69.5, 68.8, 67.4, 57.6, 50.2, 39.3, 29.7, 28.4, 28.3, 26.2, 25.9 ppm.

3'-(3,6,9,12-Tetraoxapentadec-14-yn-1-yloxy)-*N*-(7-(4-(pyridin-3-yl)-1*H*-1,2,3-triazol-1-yl)heptyl)-[1,1'-biphenyl]-2-carboxamide (56)

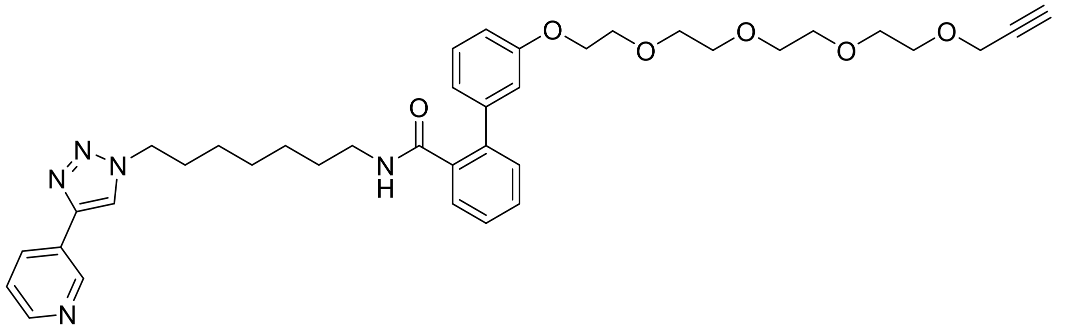

The compound was prepared from 3,6,9,12-tetraoxapentadec-14-yn-1-yl 4-methylbenzenesulfonate (**43**), according to general procedure A. The crude was purified using CH_2Cl_2/MeOH 98:2 as eluent. Yellow oil; yield 66%; ^{1}H NMR (400 MHz, $CDCl_3$) δ 8.99 (br s, 1H), 8.53 (br s, 1H), 8.21-8.18 (m, 1H), 7.93 (s, 1H), 7.65 (d, J = 7.4 Hz, 1H), 7.45-7.23 (m, 5H), 6.96-6.93 (m, 2H), 6.87 (d, J = 8.2 Hz, 1H), 5.40 (br d, 1H), 4.40 (t, J = 7.2 Hz, 2H), 4.16 (d, J = 2.3 Hz, 2H), 4.10 (t, J = 4.0 Hz, 2H), 3.83 (t, J = 5.2 Hz, 2H), 3.70-3.63 (m, 12H), 3.12 (q, J = 6.7 Hz, 2H), 2.41 (t, J = 2.3 Hz, 1H), 1.91 (quint, J = 6.4 Hz, 2H), 1.25-1.15 (m, 6H), 1.03 (quint, J = 7.4 Hz, 2H) ppm; ^{13}C NMR (101 MHz,

$CDCl_3$) δ 169.4, 158.9, 148.9, 146.8, 144.5, 141.7, 139.1, 135.8, 133.1, 130.0, 130.0, 129.6, 128.7, 127.7, 127.0, 123.8, 121.3, 120.1, 114.7, 114.1, 79.6, 74.6, 70.8, 70.6, 70.6 (2C), 70.4, 69.7, 69.1, 67.5, 58.4, 50.4, 39.6, 30.2, 28.9, 28.5, 26.4, 26.2 ppm.

General procedure for the tosylation of the polyethylene glycol *tert*-butylesters (67-70)

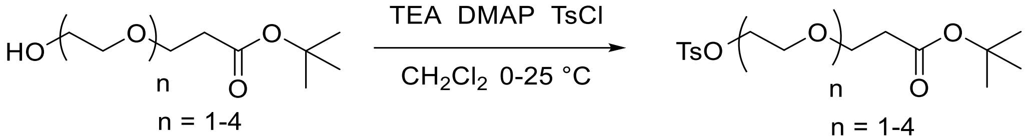

TEA (1.5 equiv) and DMAP (0.05 equiv) were added to a solution of the corresponding *tert*-buthylester functionalized polyethylene glycol (**63-66**) (1 equiv) in dry CH_2Cl_2 (0.45 M) and it was cooled down to 0 °C. Then TsCl (1.2 equiv) was added portionwise and the reaction was stirred overnight at room temperature. Upon completion, the reaction was diluted with water and extracted with CH_2Cl_2 (x2). The combined organic layers were washed with a saturated $NaHCO_3$ aqueous solution (x1), 2 M HCl aqueous solution (x1) and brine (x1). The organic phase was dried over anhydrous Na_2SO_4, filtered and the solvent was evaporated under vacuum. The resulting product was pure enough to be used in the following step.

***tert*-Butyl 3-(2-(tosyloxy)ethoxy)propanoate (67)**

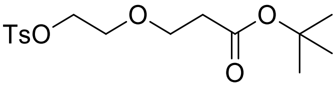

The compound was prepared from the commercially available tert-butyl 3-(2-hydroxyethoxy)propanoate (**63**) according to general procedure**.** Pale-yellow oil, yield 94%; ^{1}H NMR (400 MHz, $CDCl_3$) δ 7.68 (d, J = 8.4 Hz, 2H), 7.26 (d, J = 8.2 Hz, 2H), 4.04 (t, J = 4.7 Hz, 2H), 3.55-3.52 (m, 4H), 2.34 (s, 3H), 2.31 (t, J = 6.3 Hz, 2H), 1.34 (s, 9H) ppm; ^{13}C NMR (101 MHz, $CDCl_3$) δ 170.5, 144.8, 132.9, 129.8, 127.8, 80.4, 69.2, 68.2, 66.8, 36.0, 27.9, 21.5 ppm.

***tert*-Butyl 3-(2-(2-(tosyloxy)ethoxy)ethoxy)propanoate (68)**

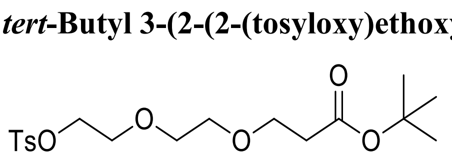

The compound was prepared from the commercially available tert-butyl 3-(2-(2-hydroxyethoxy)ethoxy)propanoate (**64**) according to general procedure. Pale-yellow oil; yield 95%; ^{1}H NMR (400 MHz, $CDCl_3$) δ 7.78 (d, J = 8.3 Hz, 2H), 7.33 (d, J = 8.0 Hz, 2H), 4.14 (t, J = 4.8 Hz, 2H), 3.68-3.64 (m, 4H), 3.55-3.51 (m, 4H), 2.46 (t, J = 6.5 Hz, 2H), 2.43 (s, 3H), 1.43 (s, 9H) ppm;

^{13}C NMR (101 MHz, $CDCl_3$) δ 170.6, 144.7, 132.9, 129.8, 127.8, 80.2, 70.4, 70.1, 69.3, 68.5, 66.7, 36.1, 27.9, 21.4 ppm.

***tert*-Butyl 3-(2-(2-(2-(tosyloxy)ethoxy)ethoxy)ethoxy)propanoate (69)**

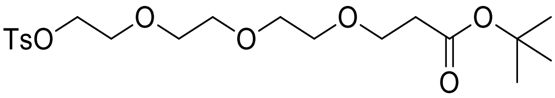

The compound was prepared from the commercially available tert-butyl 3-(2-(2-(2-hydroxyethoxy)ethoxy)ethoxy)propanoate (**65**) according to general procedure. Pale-yellow oil; yield 92%; ^{1}H NMR (400 MHz, $CDCl_3$) δ 7.55 (d, J = 7.7 Hz, 2H), 7.14 (d, J = 7.8 Hz, 2H), 3.92 (s, 2H), 3.48-3.32 (m, 12H), 2.26-2.21 (m, 5H), 1.22 (s, 9H) ppm; ^{13}C NMR (101 MHz, $CDCl_3$) δ 170.5, 144.6, 132.9, 129.7, 127.7, 80.0, 70.4, 70.2 (2C), 70.1, 69.3, 68.4, 66.6, 36.0, 27.8, 21.3 ppm.

***tert*-Butyl 1-(tosyloxy)-3,6,9,12-tetraoxapentadecan-15-oate (70)**

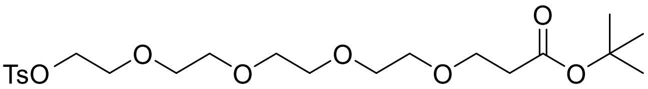

The compound was prepared from the commercially available tert-butyl 1-hydroxy-3,6,9,12-tetraoxapentadecan-15-oate (**66**) according to general procedure. pale-yellow oil; yield: 87%; ^{1}H NMR (400 MHz, $CDCl_3$) δ 7.76 (dd, J = 6.7/1.6 Hz, 2H), 7.31 (d, J = 8.0 Hz, 2H), 4.12 (t, J = 4.8 Hz, 2H), 3.68-3.63 (m, 4H), 3.60-3.54 (m, 12H), 2.46 (t, J = 6.6 Hz, 2H), 2.41 (s, 9H) ppm; ^{13}C NMR (101 MHz, $CDCl_3$) δ 170.8, 144.8, 133.0, 129.8, 127.9, 80.4, 70.7, 70.6, 70.5, 70.5, 70.4, 70.3, 69.3, 68.6, 66.8, 36.2, 28.1, 21.6 ppm.

^{1}H-NMR, ^{13}C-NMR data of the intermediates synthesized by Williamson etherification (71-74)

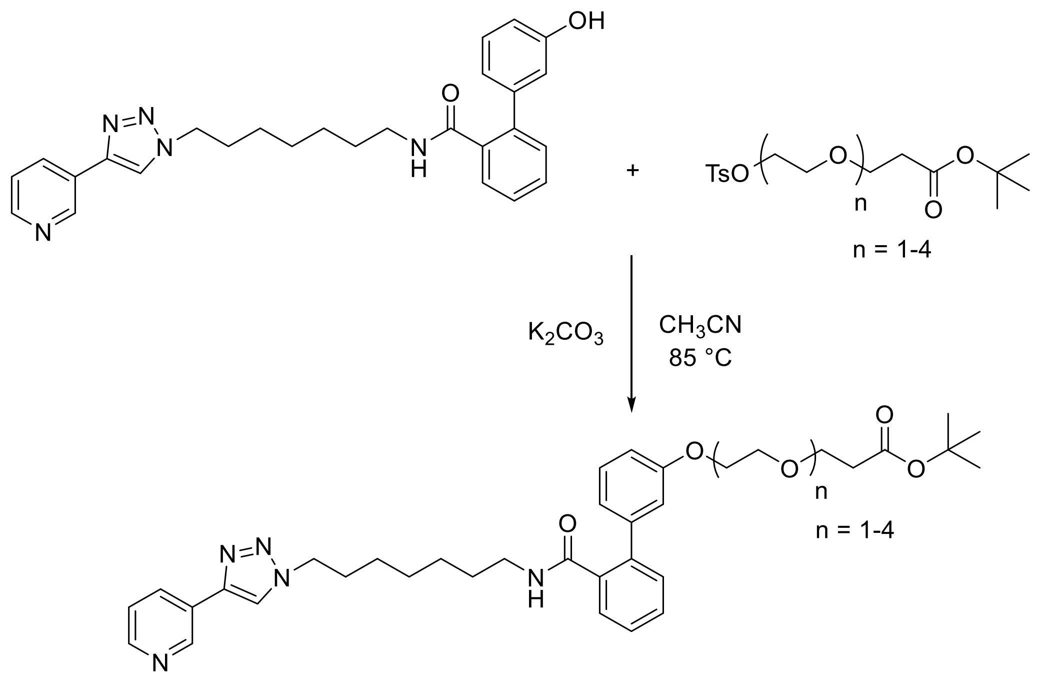

***tert*-Butyl 3-(2-((2'-((7-(4-(pyridin-3-yl)-1*H*-1,2,3-triazol-1-yl)heptyl)carbamoyl)-[1,1'-biphenyl]-3-yl)oxy)ethoxy)propanoate (71)**

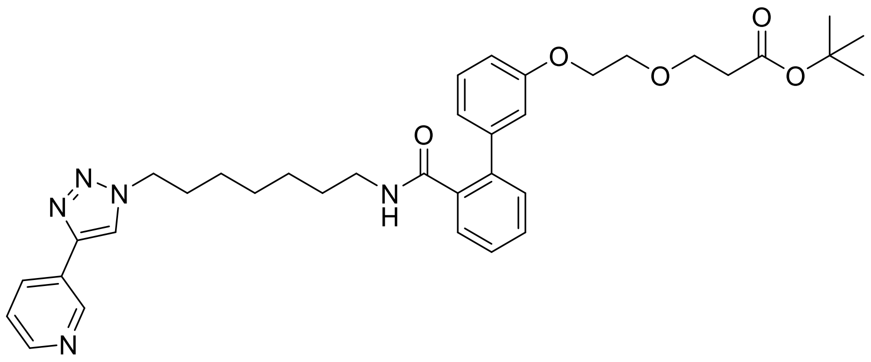

The compound was prepared from *tert*-butyl 3-(2-(tosyloxy)ethoxy)propanoate (**67**), according to general procedure A1. Pale-yellow oil; yield 94%; ^{1}H NMR (400 MHz, $CDCl_3$) δ 8.99 (s, 1H), 8.53 (d, J = 4.5 Hz, 1H), 8.18 (d, J = 7.9 Hz, 1H), 7.91 (s, 1H), 7.66 (d, J = 7.5 Hz, 1H), 7.45-7.24 (m, 5H), 6.96-6.87 (m, 3H), 5.40 (t, J = 5.5 Hz, 1H), 4.40 (t, J = 7.2 Hz, 2H), 4.09 (t, J = 4.5 Hz, 2H), 3.81-3.75 (m, 4H), 3.12 (q, J = 6.4 Hz, 2H), 2.51 (t, J = 6.4 Hz, 2H), 1.92 (quint, J = 7.0 Hz, 2H), 1.43 (s, 9H), 1.27-1.16 (m ,6H), 1.02 (quint, J = 7.0 Hz, 2H) ppm; ^{13}C NMR (101 MHz, $CDCl_3$) δ 170.8, 169.4, 158.9, 149.1, 147.0, 144.6, 141.7, 139.1, 135.8, 132.9, 130.0, 130.0, 129.6, 128.8, 127.7, 126.9, 123.7, 121.4, 120.0, 114.7, 114.2, 80.6, 69.4, 67.4, 67.1, 50.4, 39.6, 36.3, 30.2, 28.9, 28.5, 28.1, 26.4, 26.2, ppm.

***tert*-Butyl 3-(2-(2-((2'-((7-(4-(pyridin-3-yl)-1*H*-1,2,3-triazol-1-yl)heptyl)carbamoyl)-[1,1'-biphenyl]-3-yl)oxy)ethoxy)ethoxy)propanoate (72)**

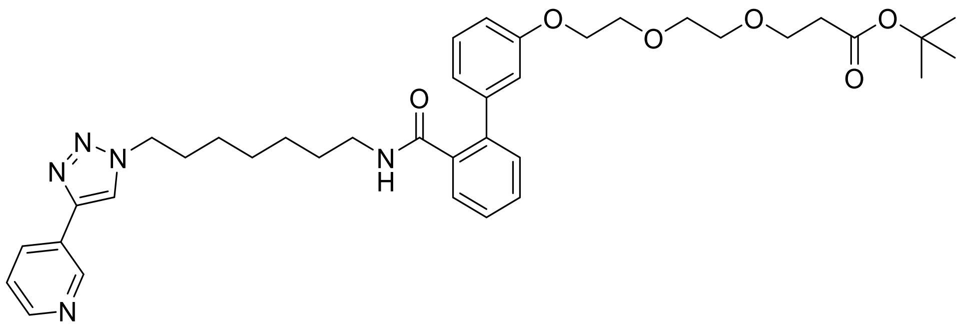

The compound was prepared from *tert*-butyl 3-(2-(2-(tosyloxy)ethoxy)ethoxy)propanoate (**68**), according to general procedure A1. Pale-yellow oil; yield 68%; ^{1}H NMR (400 MHz, $CDCl_3$) δ 9.00 (d, J = 1.6 Hz, 1H), 8.55 (dd, J = 4.8/1.6 Hz, 1H), 8.20 (dt, J = 8.0/1.9 Hz, 1H), 7.91 (s, 1H), 7.68 (dd, J = 7.4/1.2 Hz, 1H), 7.47-7.25 (m, 5H), 6.98-6.94 (m, 2H), 6.91-6.88 (m, 1H), 5.35 (t, J = 5.7 Hz, 1H), 4.41 (t, J = 7.2 Hz, 2H), 4.12 (t, J = 4.6 Hz, 2H), 3.72-3.62 (m, 6H), 3.13 (q, J = 6.2 Hz, 2H), 2.49 (t, J = 6.5 Hz, 2H), 1.93 (quint, J = 7.1 Hz, 2H), 1.43 (s, 9H), 1.32-1.16 (m, 6H), 1.04 (quint, J = 7.0 Hz, 2H) ppm; ^{13}C NMR (101 MHz, $CDCl_3$) δ 170.9, 169.4, 159.0, 149.1, 147.0, 144.6, 141.7, 139.1, 135.8, 133.0, 130.0, 130.0, 129.6, 128.8, 127.7, 126.9, 123.8, 121.4, 120.0, 114.8, 114.1, 80.5, 70.7, 70.4, 69.7, 67.5, 66.9, 50.4, 39.6, 36.2, 30.2, 28.9, 28.5, 28.1, 26.4, 26.2 ppm.

***tert*-Butyl 3-(2-(2-(2-((2'-((7-(4-(pyridin-3-yl)-1*H*-1,2,3-triazol-1-yl)heptyl)carbamoyl)-[1,1'-biphenyl]-3-yl)oxy)ethoxy)ethoxy)ethoxy)propanoate (73)**

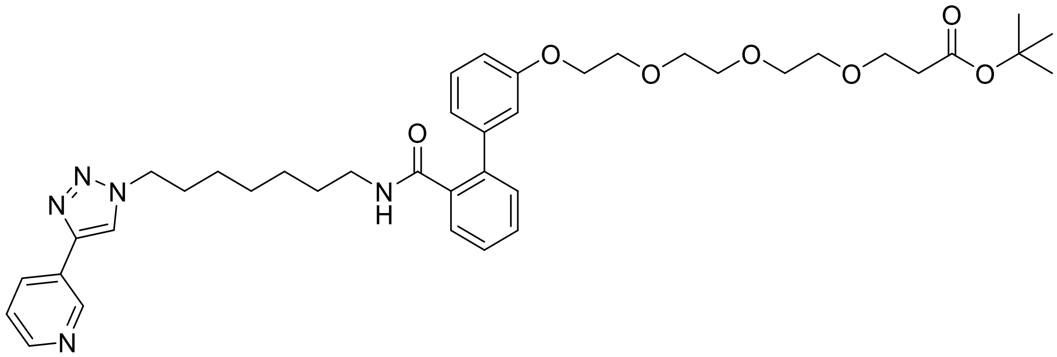

The compound was prepared from *tert*-butyl 3-(2-(2-(2-(tosyloxy)ethoxy)ethoxy)ethoxy)propanoate (**69**), according to general procedure A1. Yellow oil; yield 81%; ^{1}H NMR (400 MHz, $CDCl_3$) δ 8.97 (d, J = 1.6 Hz, 1H), 8.51 (dd, J = 4.8/1.6 Hz, 1H), 8.17 (dt, J = 8.0/2.0 Hz, 1H), 7.92 (s, 1H), 7.64 (dd, J = 7.4/1.2 Hz, 1H), 7.44-7.22 (m, 5H), 6.94-6.92 (m, 2H), 6.87-6.85 (m, 1H), 5.44 (t, J = 5.7 Hz, 1H), 4.38 (t, J = 7.2 Hz, 2H), 4.09 (t, J = 5.0 Hz, 2H), 3.82 (t, J = 4.9 Hz, 2H), 3.71-3.55 (m, 10H), 3.11 (q, J = 6.6 Hz, 2H), 2.45 (t, J = 6.6 Hz, 2H), 1.90 (quint, J = 7.0 Hz, 2H), 1.40 (s, 9H),

1.27-1.14 (m, 6H), 1.01 (quint, J = 7.1 Hz, 2H) ppm; ^{13}C NMR (101 MHz, $CDCl_3$) δ 170.9, 169.4, 158.9, 149.0, 146.9, 144.6, 141.7, 139.1, 135.9, 132.9, 130.0, 130.0, 129.6, 128.7, 127.6, 126.9, 123.7, 121.3, 120.1, 114.7, 114.1, 80.5, 70.8, 70.6, 70.5, 70.3, 69.7, 67.5, 66.9, 50.4, 39.6, 36.2, 30.1, 28.8, 28.5, 28.1, 26.4, 26.2 ppm.

***tert*-Butyl 1-((2'-((7-(4-(pyridin-3-yl)-1*H*-1,2,3-triazol-1-yl)heptyl)carbamoyl)-[1,1'-biphenyl]-3-yl)oxy)-3,6,9,12-tetraoxapentadecan-15-oate (74)**

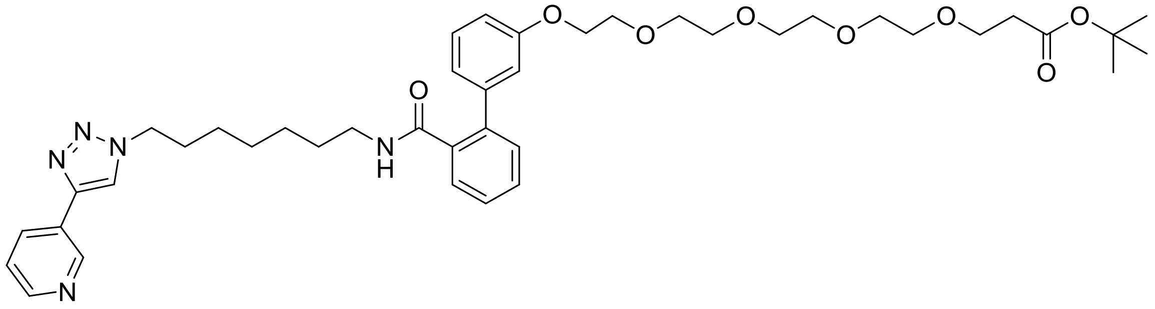

The compound was prepared from *tert*-butyl 1-(tosyloxy)-3,6,9,12-tetraoxapentadecan-15-oate (**70**), according to general procedure A1. Yellow oil; yield 97%; ^{1}H NMR (400 MHz, $CDCl_3$) δ 9.02 (d, J = 1.64 Hz, 1H), 8.57 (dd, J = 4.8/1.5 Hz, 1H), 8.22 (dt, J = 8.0/1.8 Hz, 1H), 7.92 (s, 1H), 7.70 (dd, J = 7.5/1.3 Hz, 1H), 7.48-7.26 (m, 5H), 6.99-6.95 (m, 2H), 6.91-6.89 (m, 1H), 5.31 (t, J = 5.8, 1H), 4.43 (t, J = 7.2 Hz, 2H), 4.14 (t, J = 4.6 Hz, 2H), 3.86 (t, J = 5.0 Hz, 2H), 3.74-3.59 (m, 14H), 3.15 (q, J = 6.8 Hz, 2H), 2.49 (t, J = 6.6 Hz, 2H), 1.94 (quint, J = 7.1 Hz, 2H), 1.44 (s, 9H), 1.31-1.17 (m, 6H), 1.05 (quint, J = 7.4 Hz, 2H) ppm; ^{13}C NMR (101 MHz, $CDCl_3$) δ 170.9, 169.4, 159.0, 149.1, 147.0, 144.6, 141.7, 139.1, 135.8, 133.0, 130.1, 130.0, 130.0, 129.0, 127.7, 126.9, 123.8, 121.7, 120.0, 114.8, 114.2, 80.5, 70.8, 70.6 (2C), 70.6, 70.5, 70.3, 69.7, 67.5, 66.7, 50.4, 39.7, 36.3, 30.2, 28.9, 28.5, 28.1, 26.4, 26.2 ppm.

^{1}H-NMR, ^{13}C-NMR data of the intermediates synthesized by *tert*-butylester hydrolysis (75-78)

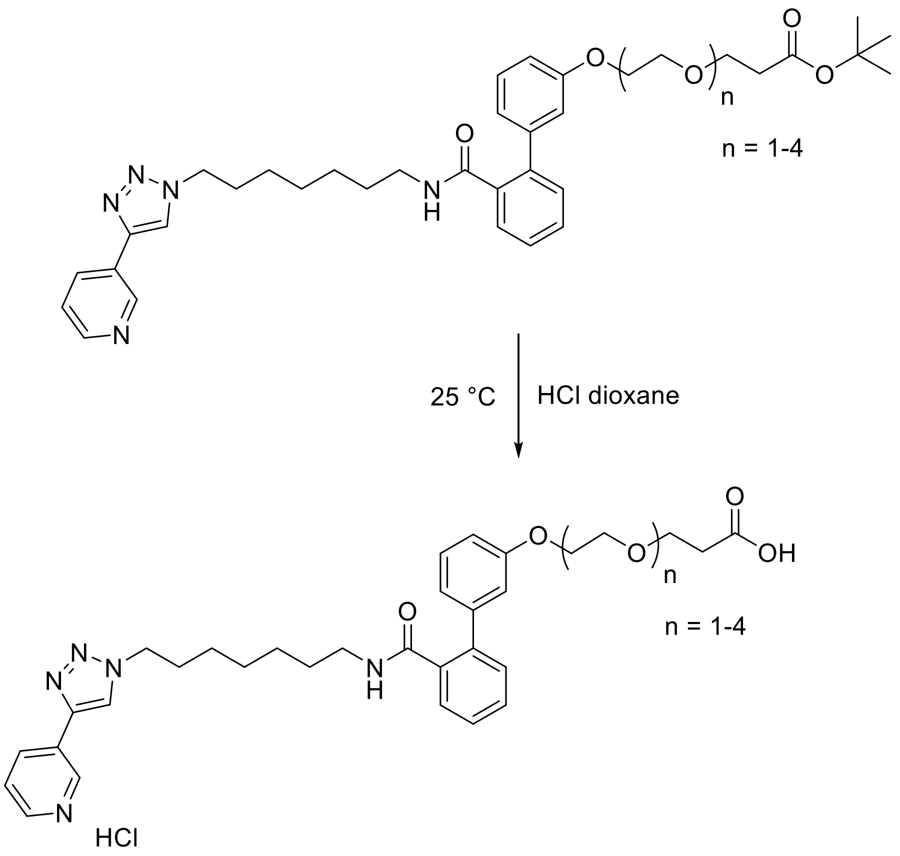

3-(2-((2'-((7-(4-(Pyridin-3-yl)-1*H*-1,2,3-triazol-1-yl)heptyl)carbamoyl)-[1,1'-biphenyl]-3-yl)oxy)ethoxy)propanoic acid hydrochloride (75)

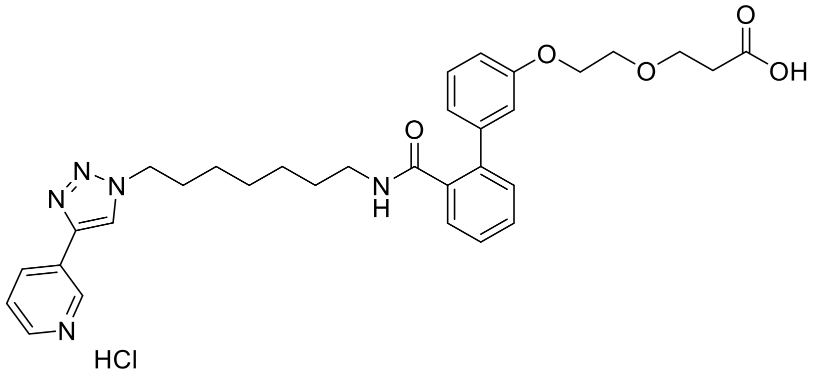

The compound was prepared from *tert*-butyl ester **71** according to general procedure C. White solid; yield quantitative; ^{1}H NMR (400 MHz, $CDCl_3$) δ 9.04 (br s, 1H), 8.50 (br s, 1H), 8.31 (br s, 1H), 8.13 (br s, 1H), 7.60 (d, J = 6.5 Hz, 1H), 7.39-7.21 (m, 5H), 6.92-6.81 (m, 3H), 5.67 (br s, 1H), 4.37 (br s, 2H), 4.05 (br s, 2H), 3.79 (br s, 4H), 3.12 (br s, 2H), 2.60 (br s, 2H), 1.88 (br s, 2H), 1.25-1.20 (m, 6H), 0.97 (br s, 2H) ppm**;** ^{13}C NMR (101 MHz, $CDCl_3$) δ 175.5, 169.7, 158.8, 147.1, 145.3, 143.8,

141.6, 139.1, 135.7, 134.5, 130.0 (2C), 129.6, 128.6, 127.8, 127.6, 124.5, 121.3, 121.0, 114.9, 114.1, 69.3, 67.5, 67.2, 50.5, 39.8, 35.5, 30.1, 28.7, 28.5, 26.3, 26.1 ppm.

3-(2-(2-((2'-((7-(4-(Pyridin-3-yl)-1*H*-1,2,3-triazol-1-yl)heptyl)carbamoyl)-[1,1'-biphenyl]-3-yl)oxy)ethoxy)ethoxy)propanoic acid hydrochloride (76)

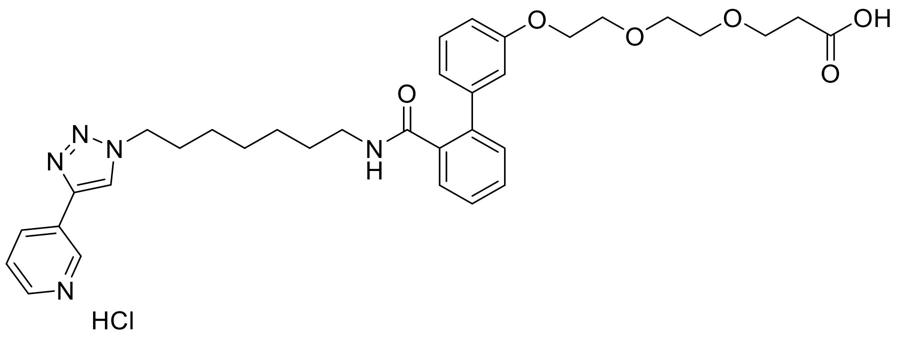

The compound was prepared from *tert*-butyl ester **72** according to general procedure C. White solid; yield quantitative; ^{1}H NMR (400 MHz, $CDCl_3$) δ 9.04 (br s, 1H), 8.53 (br s, 1H), 8.32 (br s, 1H), 8.07 (br s, 1H), 7.62 (d, J = 7.3 Hz, 1H), 7.44-7.21 (m, 5H), 6.95-6.84 (m, 3H), 5.56 (br s, 1H), 4.40 (br s, 2H), 4.08 (br s, 2H), 3.83-3.54 (m, 8H), 3.13-3.10 (m, 2H), 2.57 (br s, 2H), 1.90 (br s, 2H), 1.44-1.18 (m, 6H), 0.99 (br s, 2H) ppm; ^{13}C NMR (101 MHz, $CDCl_3$) δ 174.3, 169.7, 158.9, 147.4, 145.5, 144.0, 141.6, 139.2, 135.7, 134.2, 130.0 (2C), 129.6, 128.7, 127.7 (2C), 124.6, 121.2, 120.8, 114.8, 114.2, 70.6, 70.4, 69.7, 67.5, 66.9, 50.5, 39.6, 35.2, 30.1, 28.7, 28.5, 26.3, 26.1 ppm.

3-(2-(2-(2-((2'-((7-(4-(Pyridin-3-yl)-1*H*-1,2,3-triazol-1-yl)heptyl)carbamoyl)-[1,1'-biphenyl]-3-yl)oxy)ethoxy)ethoxy)ethoxy)propanoic acid hydrochloride (77)

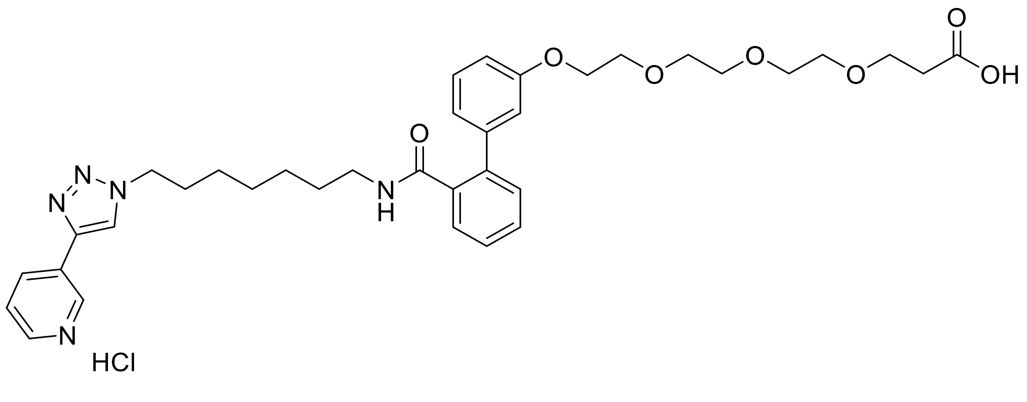

The compound was prepared from *tert*-butyl ester **73** according to general procedure C. White solid; yield quantitative; ^{1}H NMR (400 MHz, CD_3OD) δ 9.09 (br s, 1H), 8.64 (s, 1H), 8.62 (br s, 1H), 8.56 (br s, 1H), 8.34 (d, J = 7.9 Hz, 1H), 7.59-7.38 (m, 5H), 7.26 (t, J = 8.1 Hz, 1H), 6.99-6.87 (m, 3H), 4.50 (t, J = 7.0 Hz, 2H), 4.13 (t, J = 4.2 Hz, 2H), 4.84 (t, J = 4.3 Hz, 2H), 3.72-3.61 (m, 10H), 3.14

(t, J = 6.6 Hz, 2H), 2.53 (t, J = 5.9 Hz, 2H), 1.96 (quint, J = 6.8 Hz, 2H), 1.33-1.28 (m, 6H), 1.03 (br s, 2H) ppm; ^{13}C NMR (101 MHz, CD_3OD) δ 174.2, 171.5, 158.8, 147.9, 145.5, 143.9, 141.7, 139.5, 136.9, 134.0, 129.7, 129.5, 129.1, 127.3 (2C), 127.1, 124.5, 122.1, 121.0, 114.7, 113.5, 70.3, 70.2, 70.1, 70.0, 69.4, 67.3, 66.4, 50.3, 39.3, 34.5, 29.8, 28.4 (2C), 26.2, 25.9 ppm.

1-((2'-((7-(4-(Pyridin-3-yl)-1*H*-1,2,3-triazol-1-yl)heptyl)carbamoyl)-[1,1'-biphenyl]-3-yl)oxy)-3,6,9,12-tetraoxapentadecan-15-oic acid hydrochloride (78)

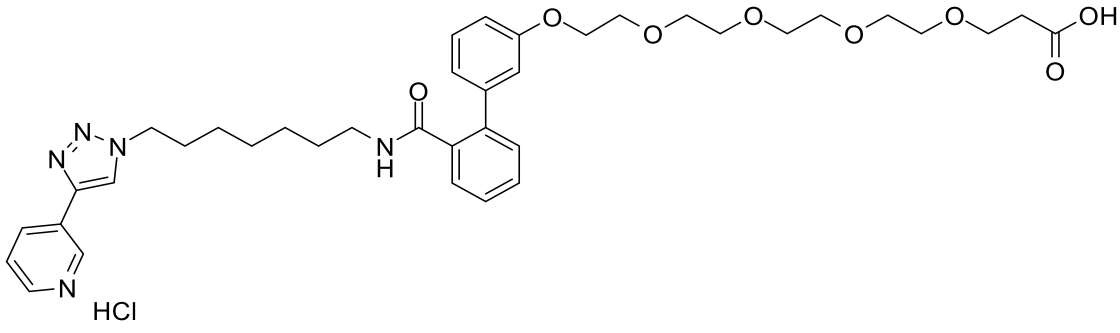

The compound was prepared from *tert*-butyl ester **74** according to general procedure C. White solid; yield: quantitative; ^{1}H NMR (400 MHz, CD_3OD) δ 9.07 (br s, 1H), 8.63 (s, 1H), 8.54 (br s, 1H), 8.32 (d, J = 7.8 Hz, 1H), 7.93 (br t, 1H), 7.56-7.38 (m, 5H), 7.26 (t, J = 8.0 Hz, 1H), 6.99-6.88 (m, 3H), 4.50 (t, J = 6.8 Hz, 2H), 4.13 (br s, 2H), 3.85 (br s, 2H), 3.76-3.59 (m, 14H), 3.15-3.13 (m, 2H), 2.53 (t, J = 6.0 Hz, 2H), 1.95-1.94 (m, 2H), 1.28 (br s, 2H), 1.03 (br s, 2H), ppm; ^{13}C NMR (101 MHz, CD_3OD) δ 174.5, 171.5, 158.7, 148.0, 145.7, 143.9, 141.8, 139.5, 136.5, 133.8, 129.8, 129.5, 129.1, 127.4, 127.1, 126.9, 124.4, 122.1, 121.1, 114.7, 113.5, 70.3, 70.1, 70.1, 70.1, 70.0, 69.9, 69.4, 67.3, 66.5, 50.3, 39.3, 34.6, 29.8, 28.4 (2C), 26.2, 25.9 ppm.

General procedure for the preparation of methyl esters (85-90)

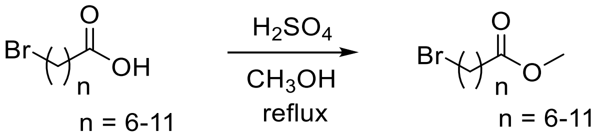

The corresponding carboxylic acid (**79-84**) (1 equiv) dissolved in MeOH (0.4 M), and H_2SO_4 96% w/w (1.1 equiv) were heated at reflux. After 4 h the solvent was evaporated under reduced pressure. The crude was diluted with a saturated $NaHCO_3$ aqueous solution (x1) and extracted with EtOAc (x3). The combined organic layers were washed with brine(x1), dried over anhydrous Na_2SO_4 and concentrated *in vacuo* to give the desired methyl ester, which was used in the next step without further purification.

Methyl 7-bromoheptanoate (85)

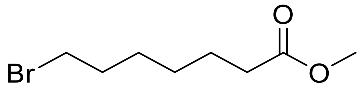

The compound was prepared from 7-bromoheptanoic acid according to general procedure. Colorless oil; yield 91%; ^{1}H NMR (400 MHz, $CDCl_3$) δ 3.63 (s, 3H), 3.37 (t, J = 6.7 Hz, 2H), 2.28 (t, J = 7.2 Hz, 2H), 1.81 (quint, J = 6.6 Hz, 2H), 1.59 (quint, J = 7.4 Hz, 2H), 1.44-1.31 (m, 4H) ppm; ^{13}C NMR (101 MHz, $CDCl_3$) δ 174.0, 51.4, 33.8, 33.7, 32.5, 28.2, 27.7, 24.6 ppm.

Methyl 8-bromooctanoate (86)

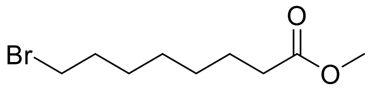

The compound was prepared from 8-bromoctanoic acid according to general procedure. Colorless oil; yield 90%; ^{1}H NMR (400 MHz, $CDCl_3$) δ 3.63 (s, 3H), 3.35 (t, J = 6.8 Hz, 2H), 2.26 (t, J = 7.4 Hz, 2H), 1.80 (quint, J = 7.5 Hz, 2H), 1.58 (quint, J = 6.9 Hz, 2H), 1.40-1.27 (m, 6H) ppm; ^{13}C NMR (101 MHz, $CDCl_3$) δ 174.0, 51.4, 33.9, 33.8, 32.6, 28.8, 28.3, 27.9, 24.7 ppm.

Methyl 9-bromononanoate (87)

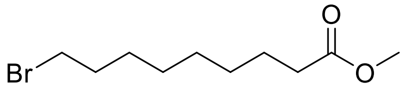

The compound was prepared from 9-bromononanoic acid according to general procedure. Colorless oil; yield 90%; ^{1}H NMR (400 MHz, $CDCl_3$) δ 3.64 (s, 3H), 3.38 (t, J = 6.8 Hz, 2H), 2.28 (t, J = 7.4 Hz, 2H), 1.83 (quint, J = 7.6 Hz, 2H), 1.60 (quint, J = 6.9 Hz, 2H), 1.40 (quint, J = 6.8 Hz, 2H), 1.30 (br s, 6H) ppm; ^{13}C NMR (101 MHz, $CDCl_3$) δ 174.2, 51.4, 34.0, 33.9, 32.7, 29.0, 29.0, 28.5, 28.0, 24.8 ppm.

Methyl 10-bromodecanoate (88)

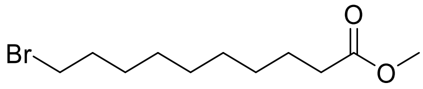

The compound was prepared from 10-bromodecanoic acid according to general procedure. The crude was purified by column chromatography using PE/EtOAc 98:2 and PE/EtOAc 95:5 as eluents to give a colorless oil; yield 88%; ^{1}H NMR (400 MHz, $CDCl_3$) δ 3.56 (s, 3H), 3.30 (t, J = 6.9 Hz, 2H), 2.21 (t, J = 7.4 Hz, 2H), 1.76 (quint, J = 6.8 Hz, 2H), 1.72-1.50 (m, 2H), 1.35-1.33 (m, 2H), 1.21 (br s,

8H) ppm; ^{13}C NMR (101 MHz, $CDCl_3$) δ 173.9, 51.2, 33.9, 33.7, 32.7, 29.1, 29.0, 29.0, 28.6, 28.0, 24.8 ppm.

Methyl 11-bromoundecanoate (89)

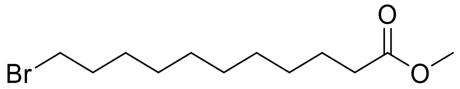

The compound was prepared from 11-bromoundecanoic acid according to general procedure. Colorless oil; yield 95%; ^{1}H NMR (400 MHz, $CDCl_3$) δ 3.66 (s, 3H), 3.40 (t, J = 6.8 Hz, 2H), 2.30 (t, J = 7.6 Hz, 2H), 1.83 (quint, J = 7.7 Hz, 2H), 1.61-1.60 (m, 2H), 1.41-1.40 (m, 2H), 1.28 (br s, 10H) ppm; ^{13}C NMR (101 MHz, $CDCl_3$) δ 173.9, 51.2, 33.9, 33.7, 32.7, 29.3, 29.2, 29.1, 29.0, 28.6, 28.1, 24.8 ppm.

Methyl 12-bromododecanoate (90)

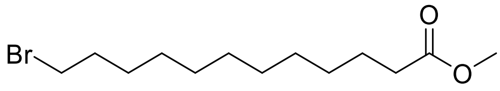

The compound was prepared from 12-bromododecanoic acid according to general procedure. Colorless oil; yield 88%; ^{1}H NMR (400 MHz, $CDCl_3$) δ 3.64 (s, 3H), 3.38 (t, J = 6.8 Hz, 2H), 2.28 (t, J = 7.4 Hz, 2H), 1.83 (quint, J = 7.6 Hz, 2H), 1.60 (t, J = 6.8 Hz, 2H), 1.42-1.40 (m, 2H), 1.26 (br s, 12H) ppm; ^{13}C NMR (101MHz, $CDCl_3$) δ 174.2, 51.4, 34.0, 33.9, 32.8, 29.4, 29.4, 29.3, 29.2, 29.1, 28.7, 28.1, 24.9 ppm.

^{1}H-NMR, ^{13}C-NMR data of the intermediates synthesized by Williamson etherification (91-96)

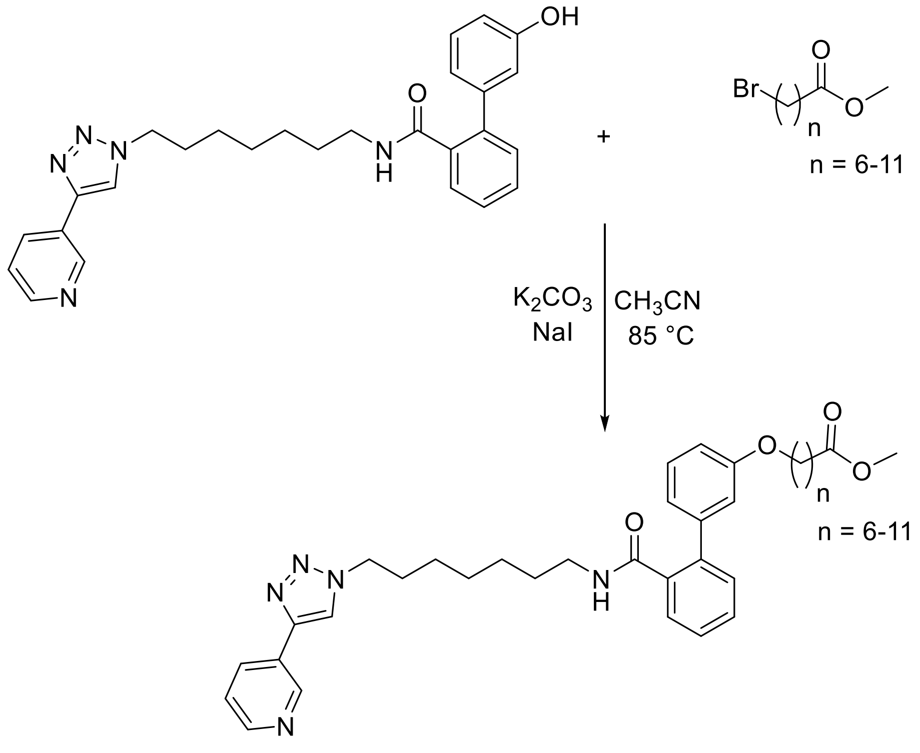

Methyl 7-((2'-((7-(4-(pyridin-3-yl)-1H-1,2,3-triazol-1-yl)heptyl)carbamoyl)-[1,1'-biphenyl]-3-yl)oxy)heptanoate (91)

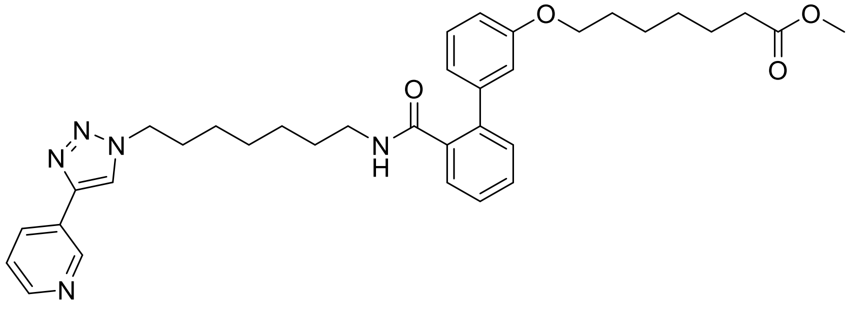

The compound was prepared from **85**, the crude was purified using CH_2Cl_2/MeOH 99:1 and CH_2Cl_2/MeOH 98:2 as eluents. Yellow oil; yield 73%; ^{1}H NMR (400 MHz, $CDCl_3$), *δ* 9.02 (br s, 1H), 8.59 (br s, 1H), 8.24 (d, *J* = 7.9 Hz, 1H), 7.90 (s, 1H), 7.71 (d, *J* = 7.6 Hz, 1H), 7.49-7.27 (m = 5H), 6.98-6.88 (m, 3H), 5.29 (t, *J* = 5.3 Hz, 1H), 4.43 (t, *J* = 7.2 Hz, 2H), 3.96 (t, *J* = 6.4 Hz, 2H), 3.67 (s, 3H), 3.16 (q, *J* = 6.4 Hz, 2H), 2.33 (t, *J* = 7.4 Hz, 2H), 1.95 (quint, *J* = 7.0 Hz, 2H), 1.79 (quint, *J* = 6.8 Hz, 2H), 1.67 (quint, *J* = 7.5 Hz, 2H), 1.52-1.17 (m, 10H), 1.05 (quint, *J* = 7.3 Hz, 2H) ppm; ^{13}C NMR *δ* (101 MHz, $CDCl_3$), δ 174.2, 169.3, 159.2, 149.0, 146.9, 144.6, 141.7, 139.3, 135.7, 133.1, 130.0, 130.0, 129.6, 128.8, 127.7, 126.9, 123.8, 120.9, 119.9, 114.6, 114.2, 67.9, 51.5, 50.5, 39.6, 34.0, 30.2, 29.1, 28.9 (2C), 28.5, 26.4, 26.2, 25.8, 24.8 ppm.

Methyl 8-((2'-((7-(4-(pyridin-3-yl)-1H-1,2,3-triazol-1-yl)heptyl)carbamoyl)-[1,1'-biphenyl]-3-yl)oxy)octanoate (92)

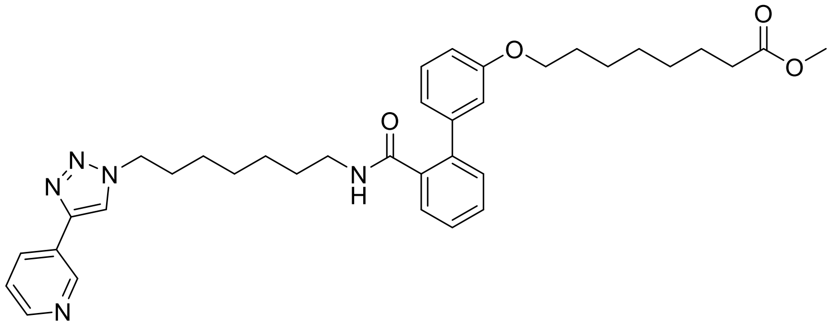

The compound was prepared from **86**, the crude was purified using CH_2Cl_2/MeOH 98:2 and CH_2Cl_2/MeOH 97:3 as eluents. Yellow oil; yield 75%; ^{1}H NMR (400 MHz, $CDCl_3$), δ 9.01 (s, 1H), 8.56 (dd, J = 4.8/1.6 Hz, 1H), 8.22 (dt, J = 8.0/2.1 Hz, 1H), 7.88 (s, 1H), 7.72-7.70 (m, 1H), 7.48-7.27 (m = 5H), 6.98-6.87 (m, 3H), 5.31 (br s, 1H), 4.41 (t, J = 7.2 Hz, 2H), 3.95 (t, J = 6.5 Hz, 2H), 3.67 (s, 3H), 3.15 (q, J = 6.1 Hz, 2H), 2.30 (t, J = 7.4 Hz, 2H), 1.94 (quint, J = 7.2 Hz, 2H), 1.78 (quint, J = 7.2 Hz, 2H), 1.64 (quint, J = 7.1 Hz, 2H), 1.47-1.17 (m, 12H), 1.05 (quint, J = 6.7 Hz, 2H) ppm; ^{13}C NMR (101 MHz, $CDCl_3$) δ 174.2, 169.3, 159.2, 149.2, 147.0, 144.7, 141.7, 139.3, 135.8, 133.0, 130.0, 130.0, 129.6, 128.8, 127.7, 126.9, 123.7, 120.9, 119.8, 114.6, 114.2, 68.0, 51.4, 50.4, 39.6, 34.0, 30.2, 29.2, 29.0, 29.0, 28.9, 28.5, 26.4, 26.2, 25.9, 24.8 ppm.

Methyl 9-((2'-((7-(4-(pyridin-3-yl)-1H-1,2,3-triazol-1-yl)heptyl)carbamoyl)-[1,1'-biphenyl]-3-yl)oxy)nonanoate (93)

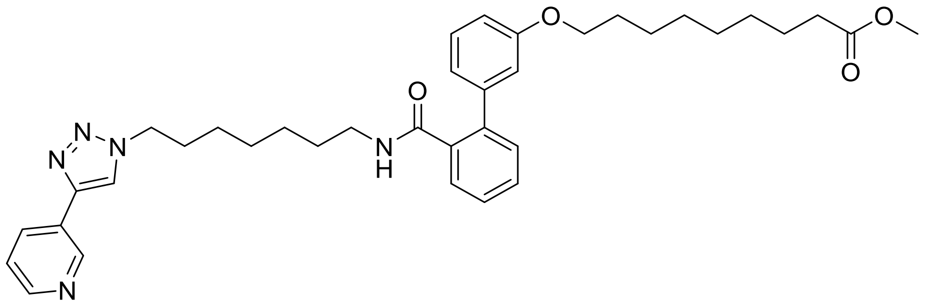

The compound was prepared from **87**, the crude was purified using CH_2Cl_2/MeOH 99:1 and CH_2Cl_2/MeOH 95:5 as eluents. Yellow oil; yield 71%; ^{1}H NMR (400 MHz, $CDCl_3$), δ 8.96 (s, 1H), 8.50 (d, J = 4.7 Hz, 1H), 8.16 (dt, J = 7.9/1.9 Hz, 1H), 7.89 (s, 1H), 7.64 (dd, J = 7.4/1.2 Hz, 1H), 7.43-7.22 (m, 5H), 6.92-6.83 (m, 3H), 5.46 (t, J = 5.6 Hz, 1H), 4.37 (t, J = 7.2 Hz, 2H), 3.91 (t, J = 6.5 Hz, 2H), 3.62 (s, 3H), 3.12 (q, J = 6.8 Hz, 2H), 2.27 (t, J = 7.4 Hz, 2H), 1.90 (quint, J = 7.0 Hz, 2H), 1.73 (quint, J = 6.9 Hz, 2H), 1.60-1.14 (m, 14H), 1.01 (quint, J = 6.8 Hz, 2H) ppm; ^{13}C NMR

(101 MHz, $CDCl_3$) δ 174.2, 169.4, 159.2, 149.0, 146.9, 144.6, 141.7, 139.3, 135.8, 132.9, 130.0, 129.9, 129.5, 128.7, 127.6, 126.9, 123.7, 120.9, 120.0, 114.6, 114.1, 68.0, 51.4, 50.4, 39.6, 34.0, 30.1, 29.2, 29.1 (2C), 29.0, 28.8, 28.5, 26.4, 26.2, 26.0, 24.9 ppm.

Methyl 10-((2'-((7-(4-(pyridin-3-yl)-1H-1,2,3-triazol-1-yl)heptyl)carbamoyl)-[1,1'-biphenyl]-3-yl)oxy)decanoate (94)

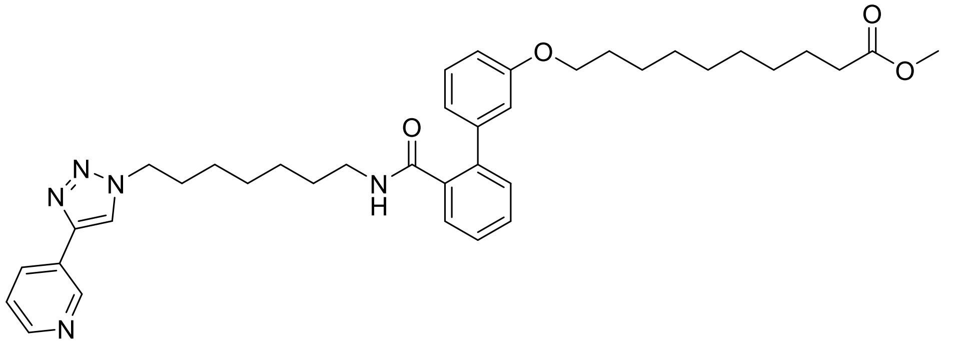

The compound was prepared from **88**, the crude was purified using CH_2Cl_2/MeOH 98:2 and CH_2Cl_2/MeOH 95:5 as eluents. Yellow oil; yield 68%; ^{1}H NMR (400 MHz, $CDCl_3$), δ 9.01 (d, J = 1.6 Hz, 1H), 8.58 (dd, J = 4.8/1.4 Hz, 1H), 8.23 (dt, J = 8.0/1.9 Hz, 1H), 7.89 (s, 1H), 7.71 (dd, J = 7.5/1.3 Hz, 1H), 7.49-7.27 (m, 5H), 6.97-6.88 (m, 3H), 5.30 (t, J = 5.6 Hz, 1H), 4.42 (t, J = 7.2 Hz, 2H), 3.95 (t, J = 6.5 Hz, 2H), 3.67 (s, 3H), 3.15 (q, J = 6.1 Hz, 2H), 2.31 (t, J = 7.5 Hz, 2H), 1.96 (quint, J = 7.1 Hz, 2H), 1.78 (quint, J = 6.9 Hz, 2H), 1.62 (quint, J = 7.2 Hz, 2H), 1.45 (quint, J = 6.7 Hz, 2H), 1.32-1.19 (m, 14H), 1.05 (quint, J = 6.9 Hz, 2H) ppm; ^{13}C NMR (101 MHz, $CDCl_3$) δ 174.3, 169.3, 159.3, 149.0, 146.9, 144.6, 141.7, 139.3, 135.7, 133.1, 130.0, 130.0, 129.6, 128.8, 127.7, 126.9, 123.8, 120.9, 119.8, 114.6, 114.2, 68.1, 51.4, 50.4, 39.6, 34.1, 30.2, 29.3, 29.2, 29.2, 29.1, 28.9, 28.5, 26.4, 26.2, 26.0, 24.9 ppm.

Methyl 11-((2'-((7-(4-(pyridin-3-yl)-1H-1,2,3-triazol-1-yl)heptyl)carbamoyl)-[1,1'-biphenyl]-3-yl)oxy)undecanoate (95)

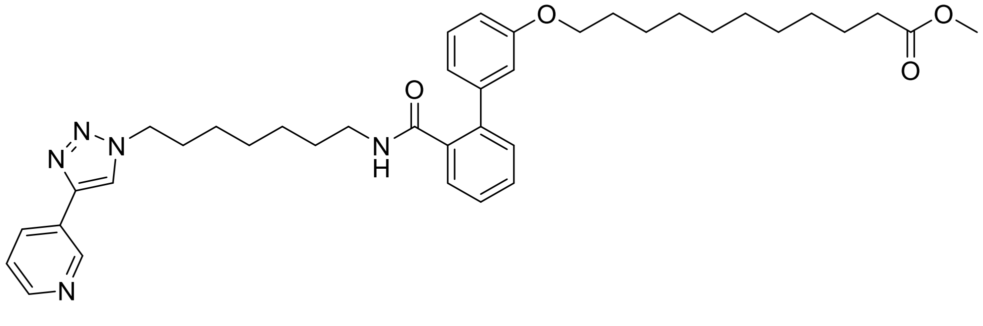

The compound was prepared from **89**, the crude was purified using CH_2Cl_2/MeOH 98:2 and CH_2Cl_2/MeOH 96:4 as eluents. Yellow oil; yield 72%; ^{1}H NMR (400 MHz, $CDCl_3$), δ 8.98 (d, J =

1.8 Hz, 1H), 8.53 (dd, J = 4.8/1.5 Hz, 1H), 8.18 (dt, J = 8.0/1.8 Hz, 1H), 7.89 (s, 1H), 7.66 (dd, J = 7.4/0.8 Hz, 1H), 7.45-7.24 (m, 5H), 6.94-6.85 (m, 3H), 5.42 (br s, 1H), 4.39 (t, J = 7.2 Hz, 2H), 3.92 (t, J = 6.5 Hz, 2H), 3.64 (s, 3H), 3.12 (q, J = 6.3 Hz, 2H), 2.28 (t, J = 7.5 Hz, 2H), 1.91 (quint, J = 7.2 Hz, 2H), 1.75 (quint, J = 7.0 Hz, 2H), 1.57 (quint, J = 7.0 Hz, 2H), 1.40 (quint, J = 7.0 Hz, 2H), 1.22-1.15 (m, 16H), 1.02 (quint, J = 7.1 Hz, 2H) ppm; ^{13}C NMR (101 MHz, $CDCl_3$) δ 174.3, 169.4, 159.2, 149.1, 146.9, 144.6, 141.7, 139.3, 135.8, 132.9, 130.0, 129.9, 129.6, 128.7, 127.6, 126.8, 123.7, 120.8, 119.9, 114.6, 114.1, 68.0, 51.4, 50.4, 39.6, 34.1, 30.2, 29.5, 29.3 (2C), 29.2, 29.2, 29.1, 28.8, 28.5, 26.4, 26.2, 26.0, 24.9 ppm.

Methyl 12-((2'-((7-(4-(pyridin-3-yl)-1H-1,2,3-triazol-1-yl)heptyl)carbamoyl)-[1,1'-biphenyl]-3-yl)oxy)dodecanoate (96)

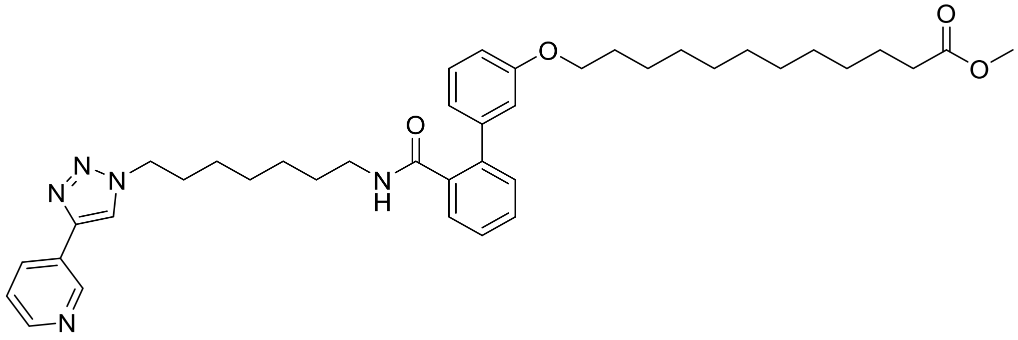

The compound was prepared from **90**, the crude was purified using CH_2Cl_2/MeOH 98:2 as eluent. Yellow oil; yield 27%; ^{1}H NMR (400 MHz, $CDCl_3$), δ 9.00 (d, J = 2.2 Hz, 1H), 8.56 (dd, J = 4.8/1.6 Hz, 1H), 8.21 (dt, J = 8.0/1.9 Hz, 2H), 7.89 (s, 1H), 7.70 (dd, J = 7.5/1.5 Hz, 1H), 7.48-7.26 (m, 5H), 6.96-6.87 (m, 3H), 5.33 (t, J = 5.7 Hz, 1H), 4.41 (t, J = 7.2 Hz, 2H), 3.49 (t, J = 6.6 Hz, 2H), 3.66 (s, 3H), 3.14 (q, J = 6.1 Hz, 2H), 2.30 (t, J = 7.4 Hz, 2H), 1.94 (quint, J = 7.1 Hz, 2H), 1.77 (quint, J = 6.9 Hz, 2H), 1.61 (quint, J = 7.2 Hz, 2H), 1.45 (quint, J = 6.5 Hz, 2H), 1.40-1.16 (m, 18H), 1.04 (quint, J = 7.5 Hz, 2H) ppm; ^{13}C NMR (101 MHz, $CDCl_3$) δ 174.3, 169.4, 159.2, 149.1, 147.0, 144.7, 141.7, 139.3, 135.8, 132.9, 130.0, 130.0, 129.6, 128.8, 127.7, 126.8, 123.7, 120.9, 119.9, 114.6, 114.2, 68.1, 51.4, 50.4, 39.6, 34.1, 30.2, 29.5, 29.5, 29.4, 29.4, 29.3, 29.2, 29.1, 28.9, 28.5, 26.4, 26.2, 26.1, 24.9 ppm.

^{1}H-NMR, ^{13}C-NMR data of the intermediates synthesized by basic hydrolysis of esters (97-102)

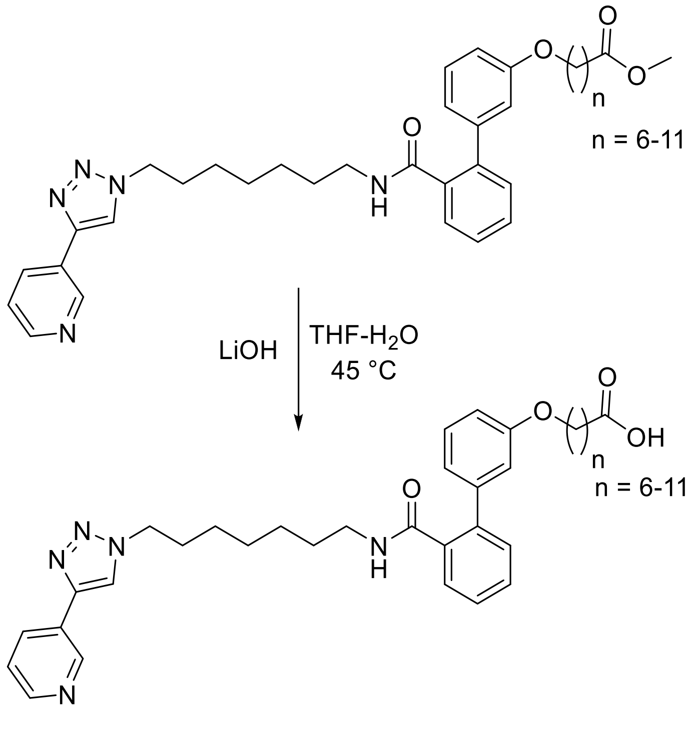

7-((2'-((7-(4-(Pyridin-3-yl)-1H-1,2,3-triazol-1-yl)heptyl)carbamoyl)-[1,1'-biphenyl]-3-yl)oxy)heptanoic acid hydrochloride (97)

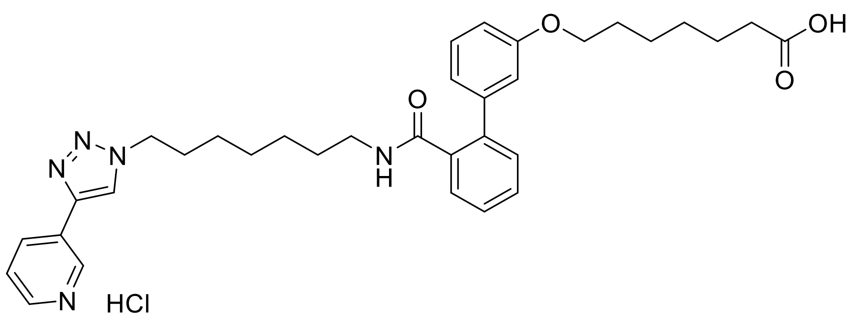

The compound was prepared from **91**. White solid; yield 82%; ^{1}H NMR (400 MHz, $CDCl_3$) δ 9.01 (s, 1H), 8.52 (d, J = 4.1 Hz, 1H), 8.29 (dt, J = 8.0/1.8 Hz, 1H), 7.98 (s, 1H), 7.67 (dd, J = 7.5/1.2 Hz, 1H), 7.47-7.25 (m, 5H), 6.95-6.85 (m, 3H), 5.43 (t, J = 5.7 Hz, 1H), 4.40 (t, J = 7.2 Hz, 2H), 3.94 (t, J = 6.4 Hz, 2H), 3.14 (q, J = 6.2 Hz, 2H), 2.36 (t, J = 7.3 Hz, 2H), 1.92 (quint, J = 7.0 Hz, 2H), 1.78 (quint, J = 6.7 Hz, 2H), 1.68 (quint, J = 7.4 Hz, 2H), 1.52-1.38 (m, 4H), 1.28-1.17 (m, 6H), 1.01 (quint, J = 6.9 Hz, 2H) ppm; ^{13}C NMR (101 MHz, $CDCl_3$) δ 177.1, 169.6, 159.2, 148.0, 146.1, 144.2, 141.6, 139.3, 135.7, 133.7, 130.0 (2C), 129.6, 128.8, 127.7, 127.3, 124.2, 120.9, 120.3, 114.6, 114.2, 67.9, 50.5, 39.7, 34.1, 30.2, 29.0, 28.8 (2C), 28.5, 26.4, 26.2, 25.8, 24.8 ppm.

8-((2'-((7-(4-(Pyridin-3-yl)-1H-1,2,3-triazol-1-yl)heptyl)carbamoyl)-[1,1'-biphenyl]-3-yl)oxy)octanoic acid hydrochloride (98)

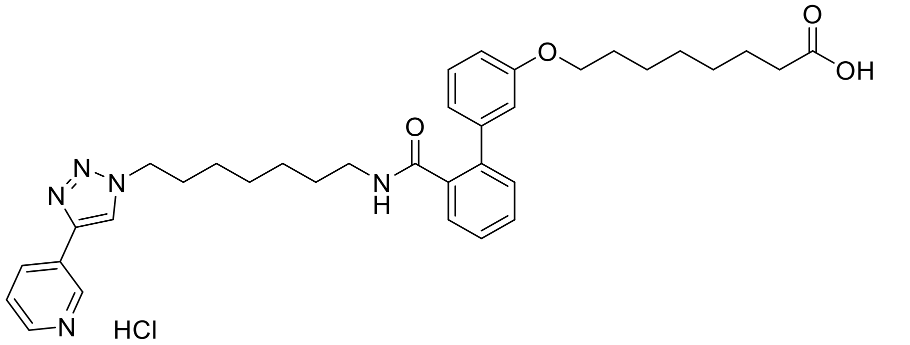

The compound was prepared from **92**. White solid; yield 78%; ^{1}H NMR (400 MHz, $CDCl_3$) δ 9.02 (br s, 1H), 8.55 (br s, 1H), 8.28 (d, J = 7.9 Hz, 1H), 7.95 (s, 1H), 7.68 (dd, J = 7.5/1.1 Hz, 1H), 7.46-7.25 (m, 5H), 6.96-6.86 (m, 3H), 5.41 (t, J = 5.7 Hz, 1H), 4.40 (t, J = 7.2 Hz, 2H), 3.94 (t, J = 6.5 Hz, 2H), 3.14 (q, J = 6.2 Hz, 2H), 2.35 (t, J = 7.3 Hz, 2H), 1.92 (quint, J = 7.0 Hz, 2H), 1.77 (quint, J = 6.8 Hz, 2H), 1.66 (quint, J = 7.0 Hz, 2H), 1.48-1.39 (m, 6H), 1.38-1.16 (m, 6H), 1.03 (quint, J = 7.0 Hz, 2H) ppm; ^{13}C NMR (101 MHz, $CDCl_3$) δ 177.0, 169.6, 159.2, 148.0, 146.0, 144.2, 141.6, 139.3, 135.6, 133.7, 130.0, 130.0, 129.6, 128.7, 127.6, 127.4, 124.2, 120.9, 120.3, 114.6, 114.1, 67.9, 50.4, 39.7, 34.4, 30.1, 29.1, 29.0, 28.9, 28.8, 28.5, 26.4, 26.2, 25.8, 24.8 ppm.

9-((2'-((7-(4-(Pyridin-3-yl)-1H-1,2,3-triazol-1-yl)heptyl)carbamoyl)-[1,1'-biphenyl]-3-yl)oxy)nonanoic acid hydrochloride (99)

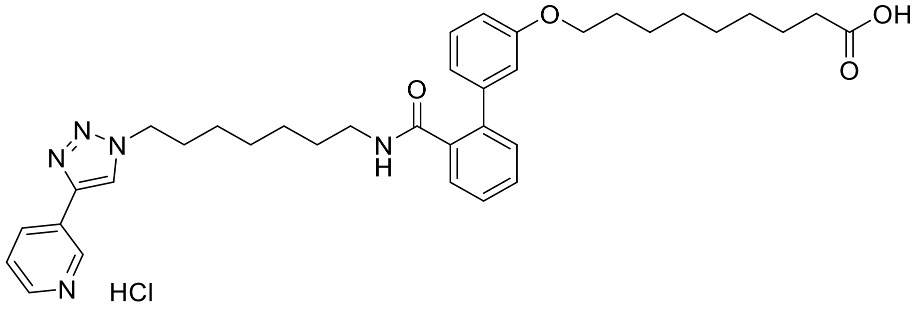

The compound was prepared from **93**. White solid; yield 81%; ^{1}H NMR (400 MHz, $CDCl_3$) δ 8.98 (br s, 1H), 8.51 (d, J = 4.1 Hz, 1H), 8.25 (dt, J = 8.0/1.8 Hz, 1H), 7.95 (s, 1H), 7.65 (dd, J = 7.5/1.2 Hz, 1H), 7.44-7.23 (m, 5H), 6.93-6.84 (m, 3H), 5.45 (t, J = 5.8 Hz, 1H), 4.38 (t, J = 7.1 Hz, 2H), 3.91 (t, J = 6.5 Hz, 2H), 3.13 (q, J = 6.2 Hz, 2H), 2.32 (t, J = 7.4 Hz, 2H), 1.90 (quint, J = 7.0 Hz, 2H), 1.74 (quint, J = 6.8 Hz, 2H), 1.62 (quint, J = 6.7 Hz, 2H), 1.44-1.14 (m, 14H), 1.00 (quint, J = 7.0 Hz, 2H) ppm; ^{13}C NMR (101 MHz, $CDCl_3$) δ 177.3, 169.6, 159.2, 148.0, 146.0, 144.2, 141.6, 139.3, 135.7, 133.7, 130.0, 129.8, 129.6, 128.7, 127.6, 127.2, 124.1, 120.8, 120.3, 114.6, 114.1, 68.0, 50.5, 39.7, 34.3, 30.1, 29.2, 29.1, 29.1, 29.0, 28.8, 28.5, 26.4, 26.2, 25.9, 24.9 ppm.

10-((2'-((7-(4-(Pyridin-3-yl)-1H-1,2,3-triazol-1-yl)heptyl)carbamoyl)-[1,1'-biphenyl]-3-yl)oxy)decanoic acid hydrochloride (100)

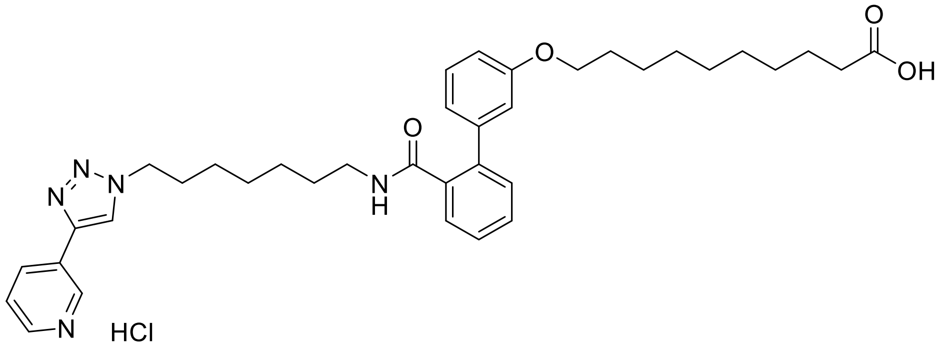

The compound was prepared from **94**. White solid; yield 72%; ^{1}H NMR (400 MHz, CDCl$_3$) δ 9.03 (br s, 1H), 8.56 (d, J = 4.2 Hz, 1H), 8.33 (dt, J = 8.0/1.8 Hz, 1H), 7.95 (s, 1H), 7.70 (dd, J = 7.5/1.2 Hz, 1H), 7.48-7.27 (m, 5H), 6.97-6.87 (m, 3H), 5.34 (t, J = 5.6 Hz, 1H), 4.42 (t, J = 7.2 Hz, 2H), 3.94 (t, J = 6.5 Hz, 2H), 3.15 (q, J = 6.2 Hz, 2H), 2.35 (t, J = 7.3 Hz, 2H), 1.94 (quint, J = 7.0 Hz, 2H), 1.77 (quint, J = 6.8 Hz, 2H), 1.65 (quint, J = 7.1 Hz, 2H), 1.47-1.16 (m, 16H), 1.04 (quint, J = 7.0 Hz, 2H) ppm; ^{13}C NMR (101 MHz, CDCl$_3$) δ 177.2, 169.5, 159.3, 148.1, 146.1, 144.3, 141.7, 139.3, 135.6, 133.7, 130.0 (2C), 129.6, 128.8, 127.7, 127.3, 124.1, 120.8, 120.1, 114.5, 114.3, 68.1, 50.5, 39.7, 34.1, 30.2, 29.2, 29.2, 29.1, 29.0, 28.9, 28.8, 28.5, 26.4, 26.2, 26.0, 24.8 ppm.

11-((2'-((7-(4-(Pyridin-3-yl)-1H-1,2,3-triazol-1-yl)heptyl)carbamoyl)-[1,1'-biphenyl]-3-yl)oxy)undecanoic acid hydrochloride (101)

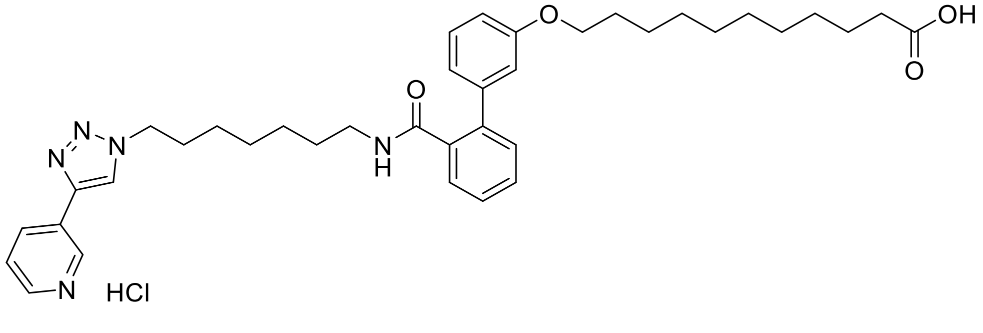

The compound was prepared from **95**. White solid; yield 77%; ^{1}H NMR (400 MHz, CDCl$_3$) δ 9.02 (br s, 1H), 8.55 (br s, 1H), 8.28 (d, J = 7.9 Hz, 1H), 7.94 (s, 1H), 7.67 (dd, J = 7.0/1.2 Hz, 1H), 7.46-7.24 (m, 5H), 6.94-6.85 (m, 3H), 5.44 (br s, 1H), 4.40 (t, J = 7.5 Hz, 2H), 3.93 (t, J = 6.5 Hz, 2H), 3.13 (q, J = 6.3 Hz, 2H), 2.33 (t, J = 7.4 Hz, 2H), 1.91 (quint, J = 6.9 Hz, 2H), 1.75 (quint, J = 6.9 Hz, 2H), 1.63 (quint, J = 7.0 Hz, 2H), 1.42-1.15 (m, 18H), 1.01 (quint, J = 7.0 Hz, 2H) ppm; ^{13}C NMR (101 MHz, CDCl$_3$) δ 177.4, 169.6, 159.2, 148.1, 146.1, 144.3, 141.6, 139.3, 135.6, 133.6,

130.0, 130.0, 129.6, 128.8, 127.6, 127.1, 124.2, 120.8, 120.2, 114.6, 114.2, 68.0, 50.5, 39.7, 34.3, 30.1, 29.4, 29.3 (2C), 29.2, 29.2, 29.1, 28.8, 28.5, 26.4, 26.2, 26.0, 24.9 ppm.

12-((2'-((7-(4-(Pyridin-3-yl)-1H-1,2,3-triazol-1-yl)heptyl)carbamoyl)-[1,1'-biphenyl]-3-yl)oxy)dodecanoic acid hydrochloride (102)

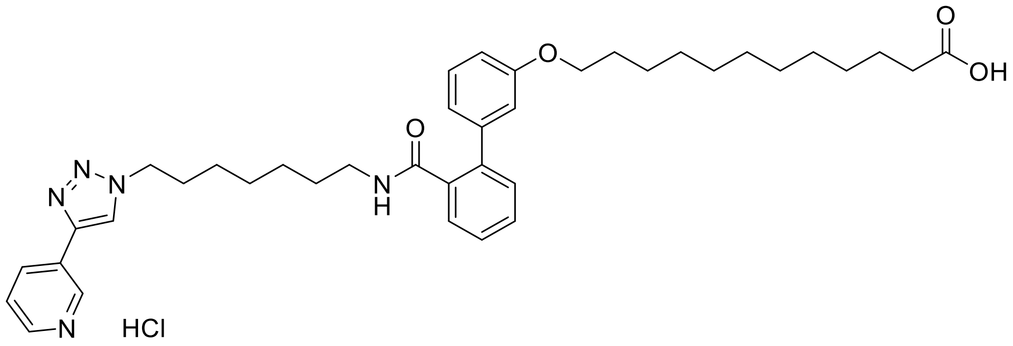

The compound was prepared from **96**. White solid; yield 72%; ^{1}H NMR (400 MHz, $CDCl_3$) δ 9.01 (br s, 1H), 8.55 (br s, 1H), 8.29 (d, J = 8.0 Hz, 1H), 7.94 (s, 1H), 7.69 (dd, J = 7.6/1.0 Hz, 1H), 7.47-7.26 (m, 5H), 6.96-6.87 (m, 3H), 5.38 (t, J = 5.7 Hz, 1H), 4.41 (t, J = 7.2 Hz, 2H), 3.94 (t, J = 6.5 Hz, 2H), 3.14 (q, J = 6.3 Hz, 2H), 2.34 (t, J = 7.4 Hz, 2H), 1.93 (quint, J = 7.0 Hz, 2H), 1.76 (quint, J = 6.9 Hz, 2H), 1.64 (quint, J = 7.0 Hz, 2H), 1.45-1.16 (m, 20H), 1.03 (quint, J = 7.0 Hz, 2H) ppm; ^{13}C NMR (101 MHz, $CDCl_3$) δ 177.5, 169.5, 159.3, 148.2, 146.2, 144.3, 141.7, 139.3, 135.6, 133.6, 130.0, 130.0, 129.6, 128.8, 127.6, 127.2, 124.1, 120.8, 120.1, 114.6, 114.3, 68.1, 50.5, 39.7, 34.2, 30.2, 29.4, 29.4, 29.3 (2C), 29.2, 29.2, 29.0, 28.8, 28.5, 26.4, 26.2, 26.0, 24.9 ppm.

NMR spectra of PROTACs U14-25, U30-35, U42.

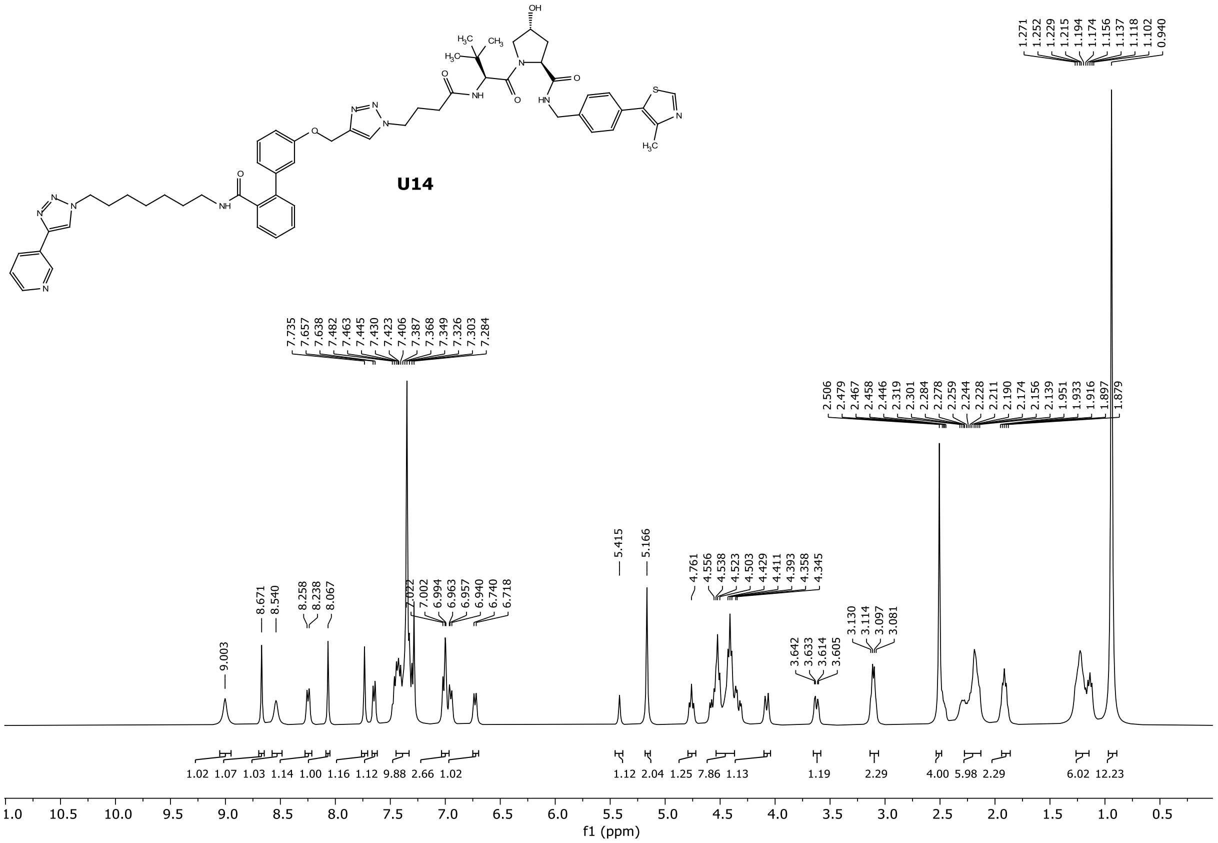

Figure S1. ^{1}H NMR spectrum of compound **U14**.

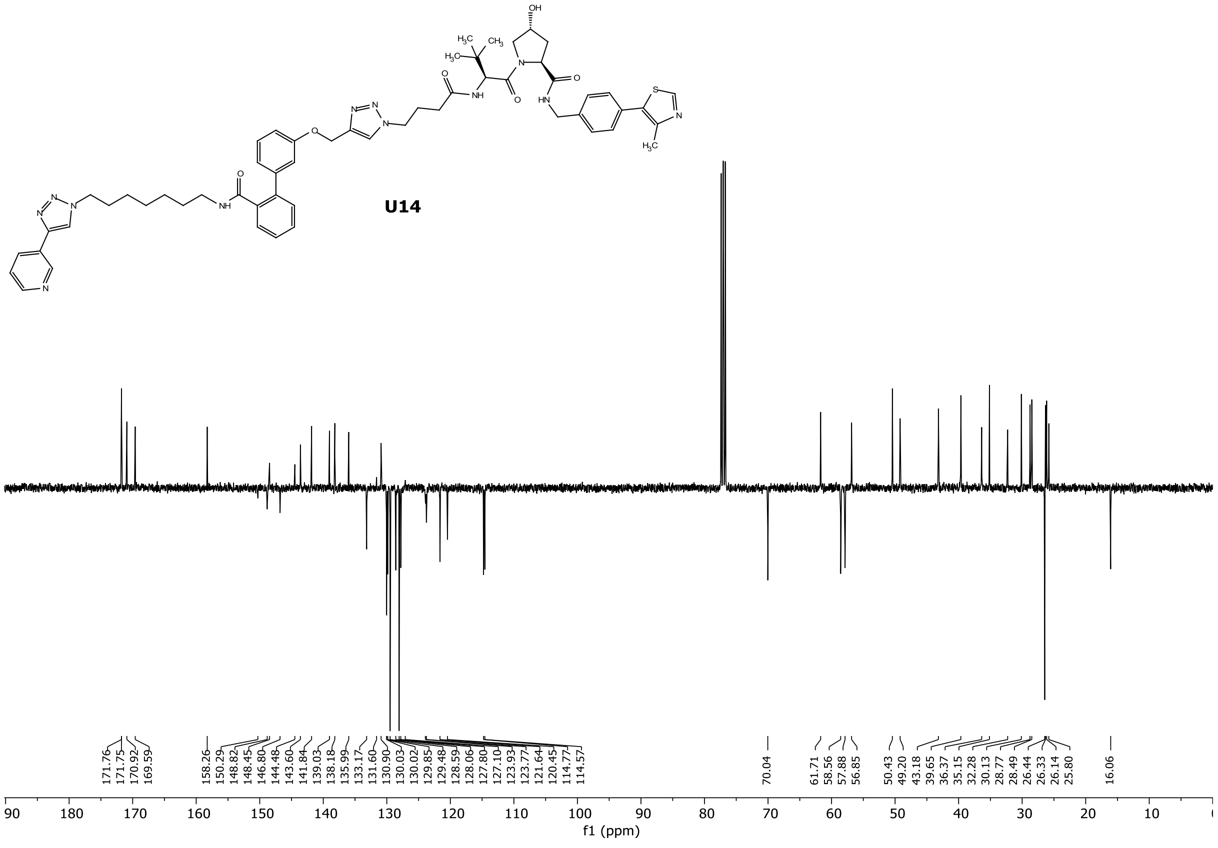

Figure S2. ^{13}C APT NMR spectrum of compound **U14**.

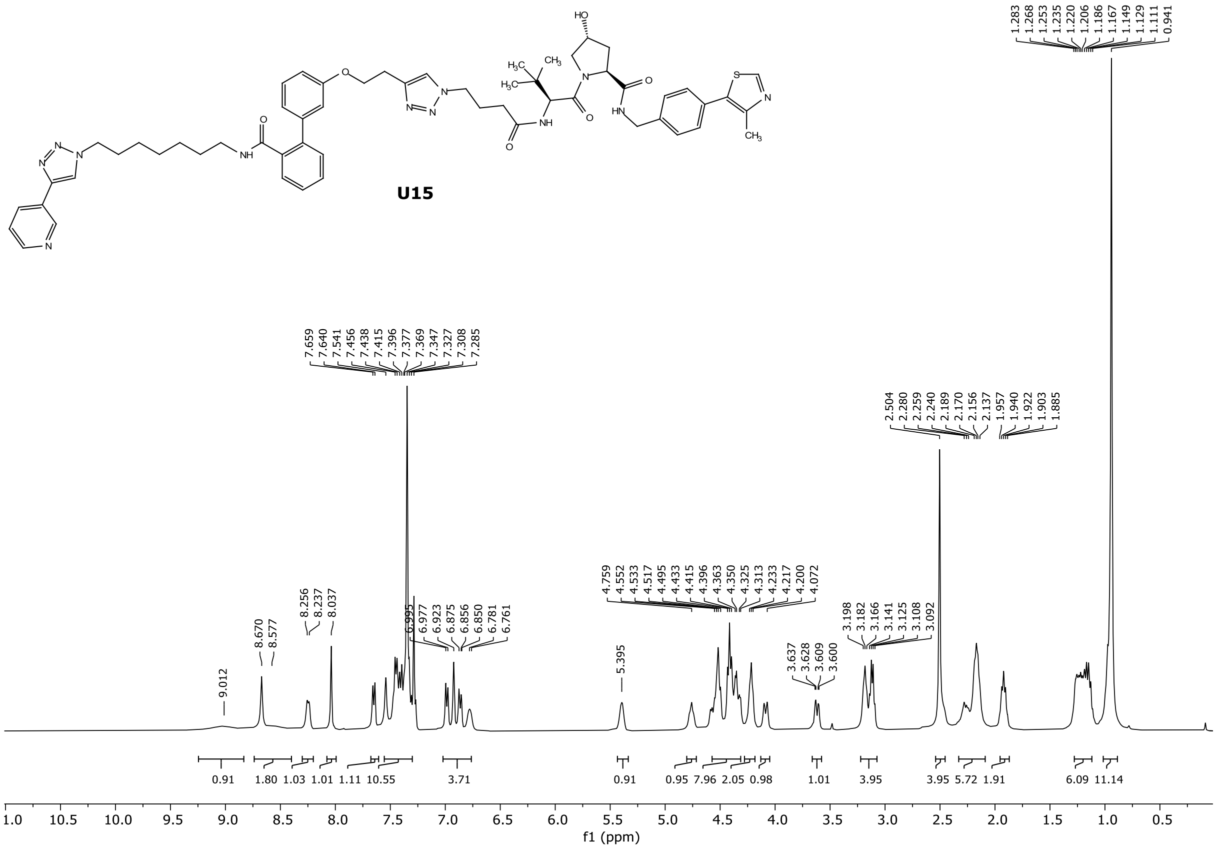

Figure S3. ^{1}H NMR spectrum of compound **U15.**

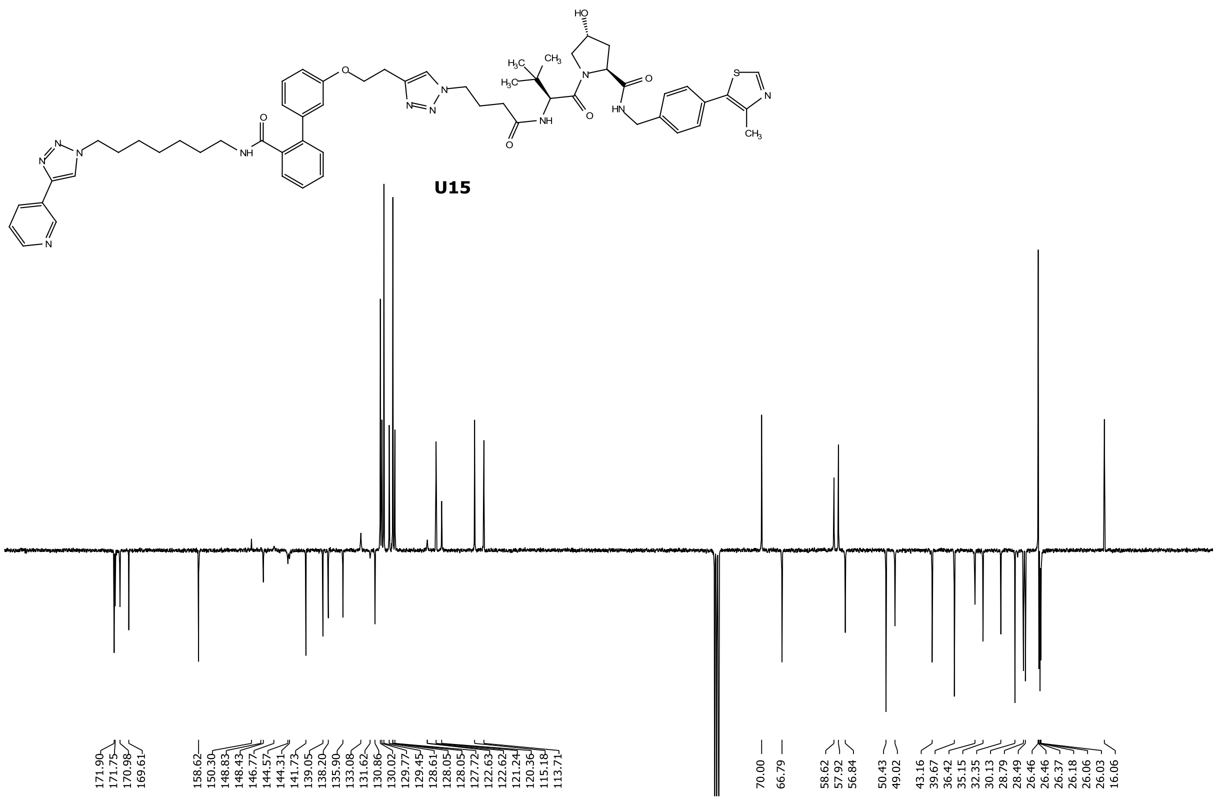

Figure S4. ^{13}C APT NMR spectrum of compound **U15**.

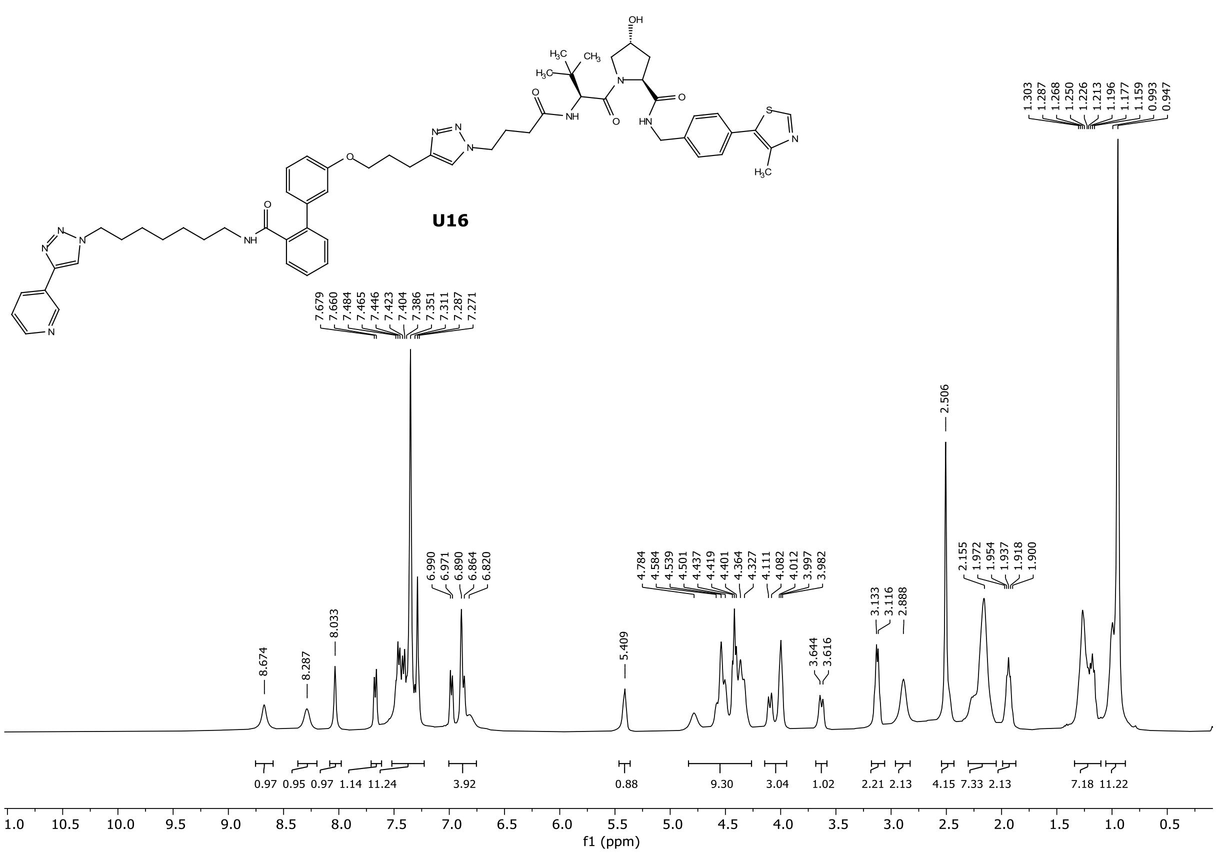

Figure S5. ^{1}H NMR spectrum of compound **U16**.

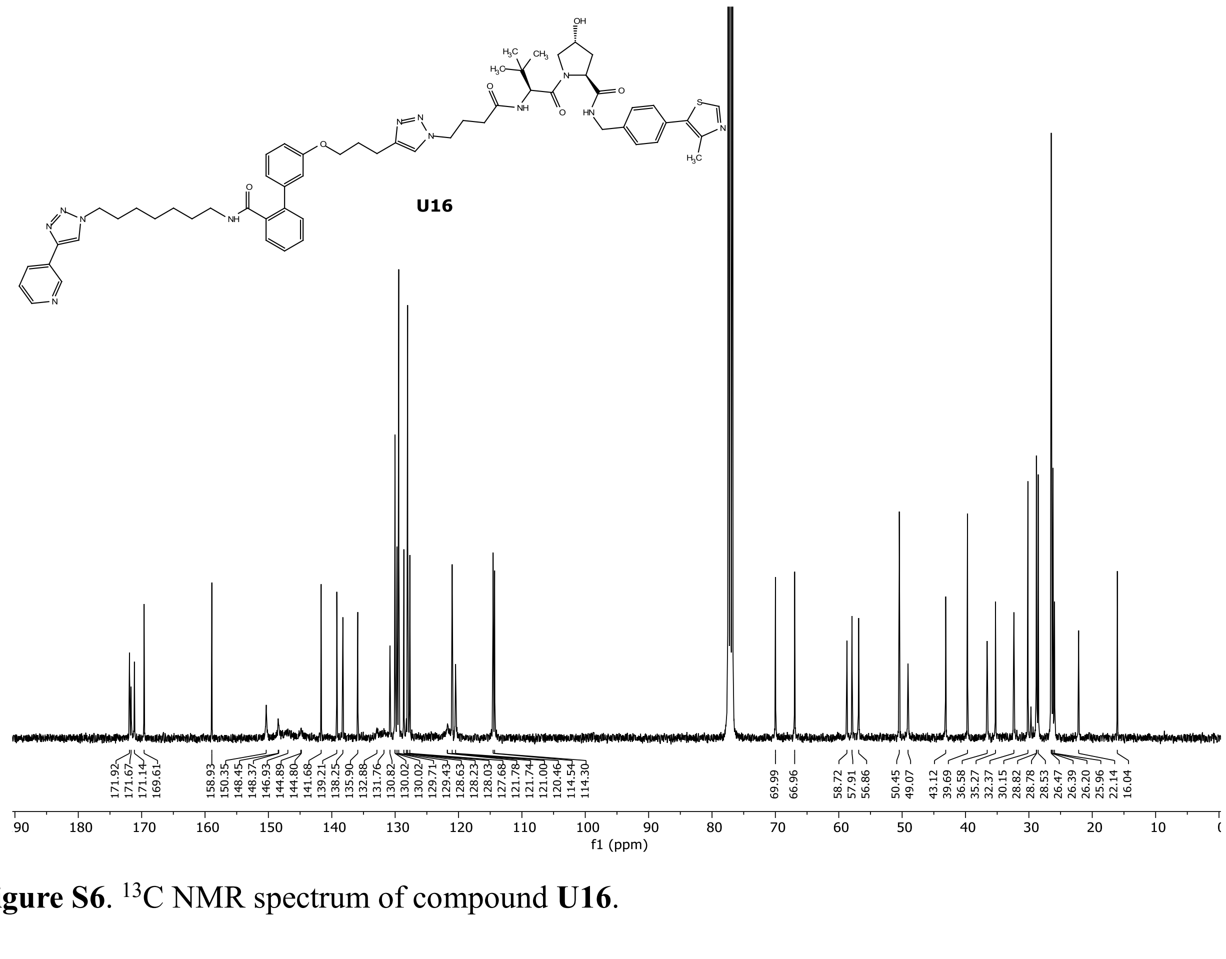

Figure S6. ^{13}C NMR spectrum of compound **U16**.

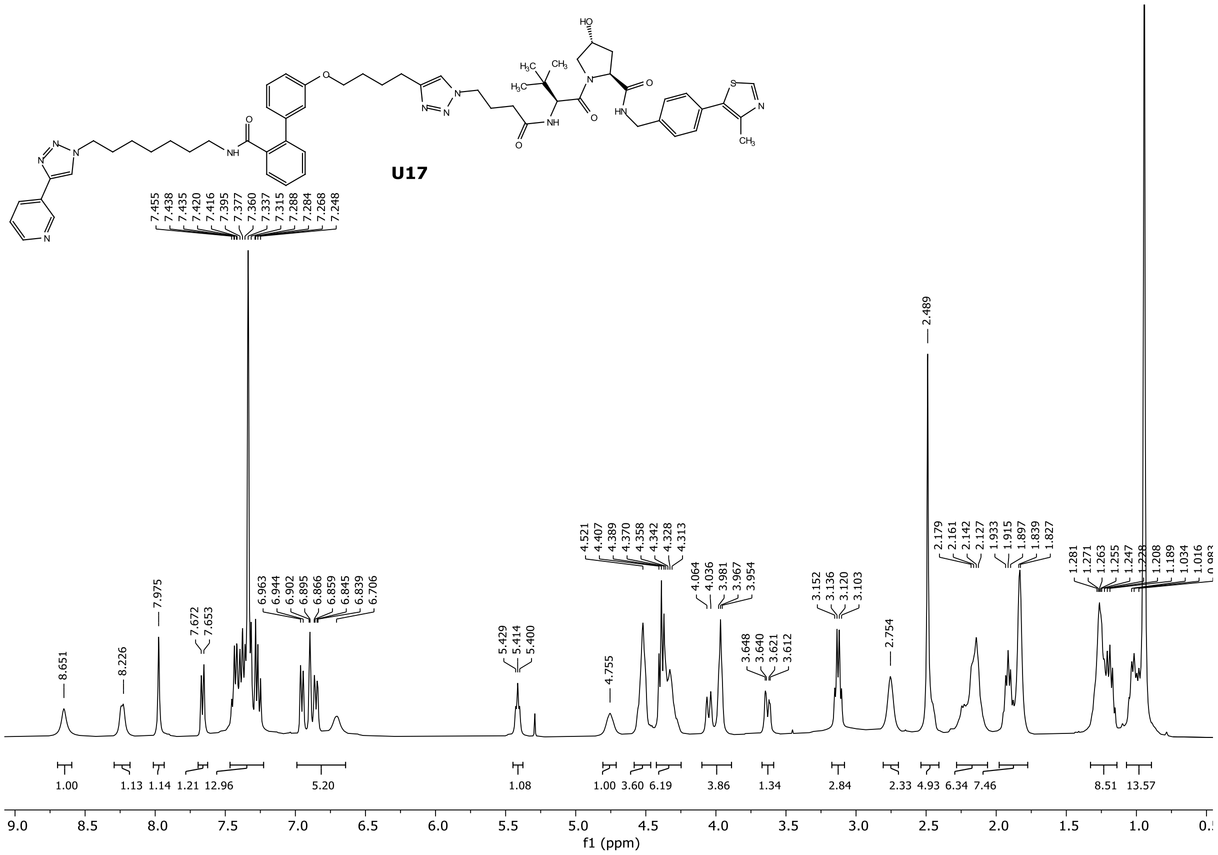

Figure S7. ^{1}H NMR spectrum of compound **U17**.

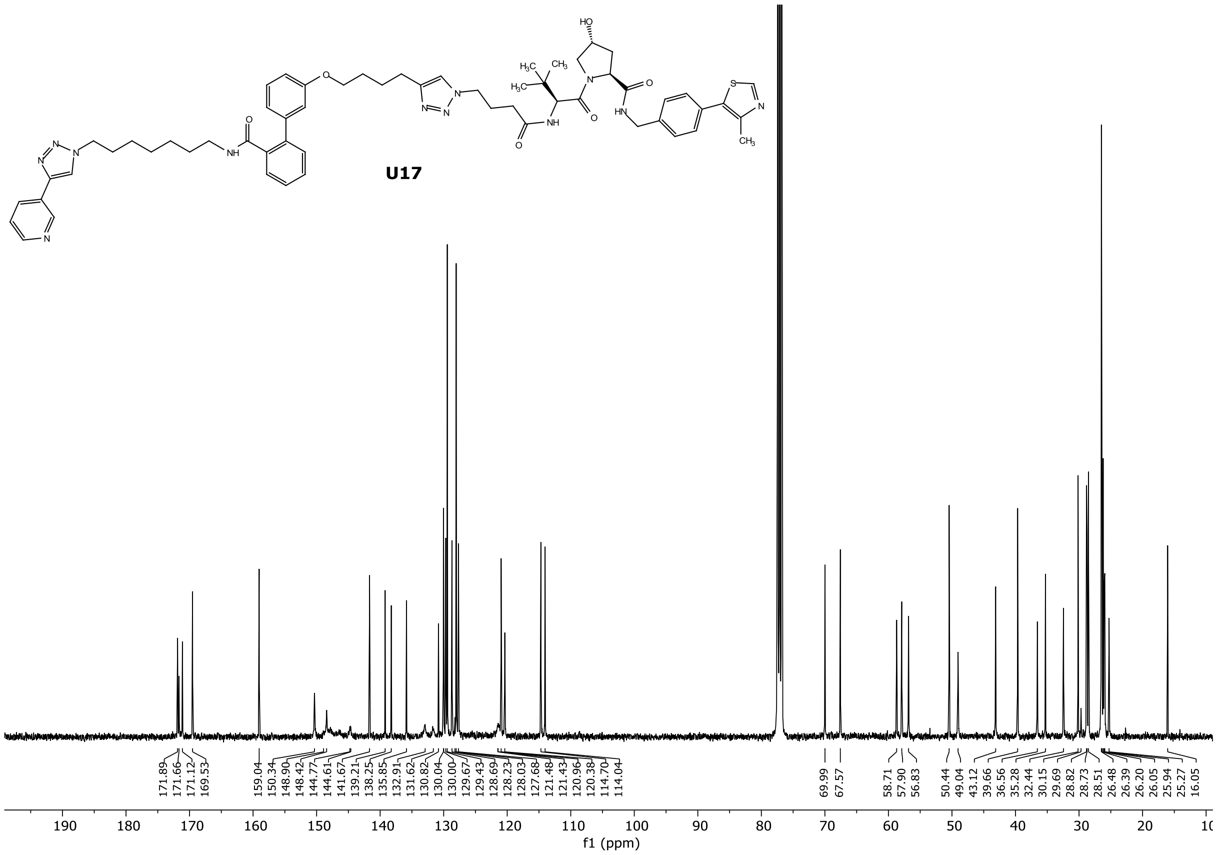

Figure S8. ^{13}C NMR spectrum of compound **U17**.

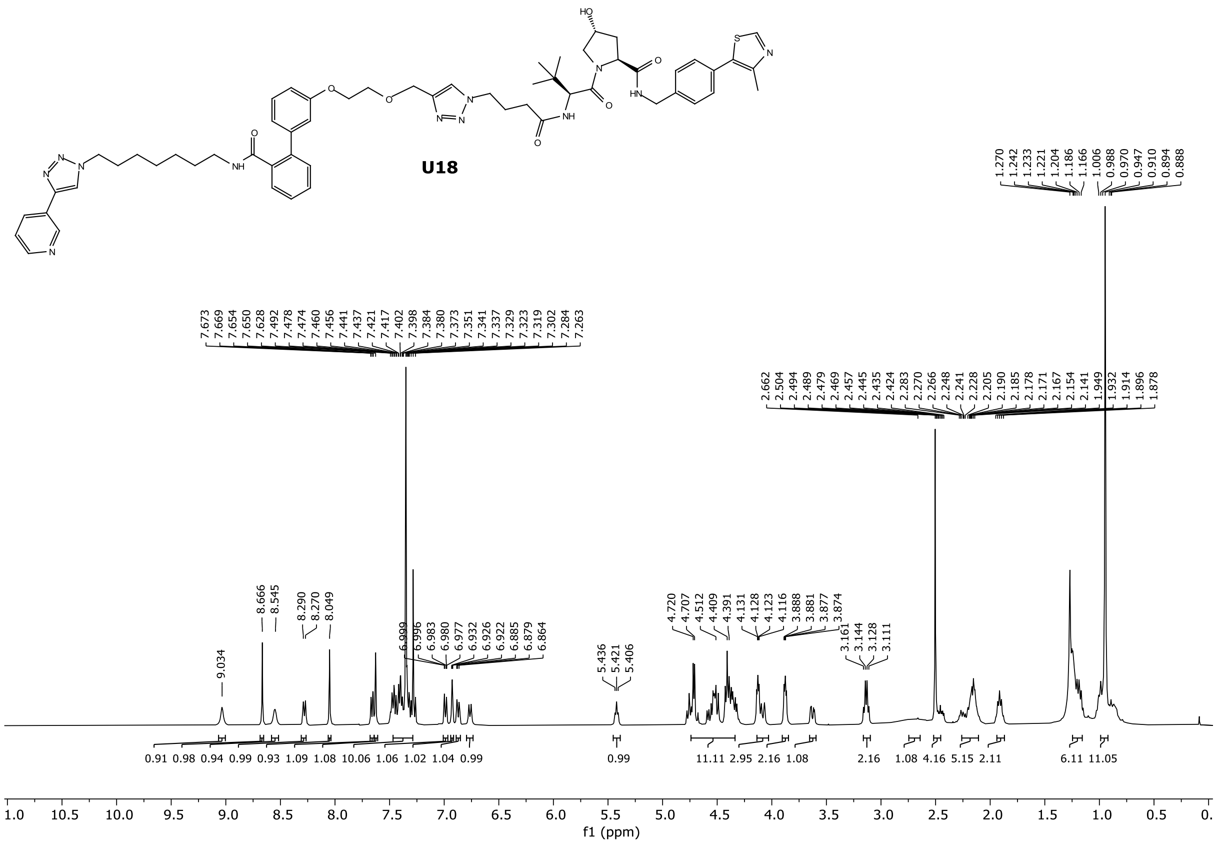

Figure S9. ^{1}H NMR spectrum of compound **U18**.

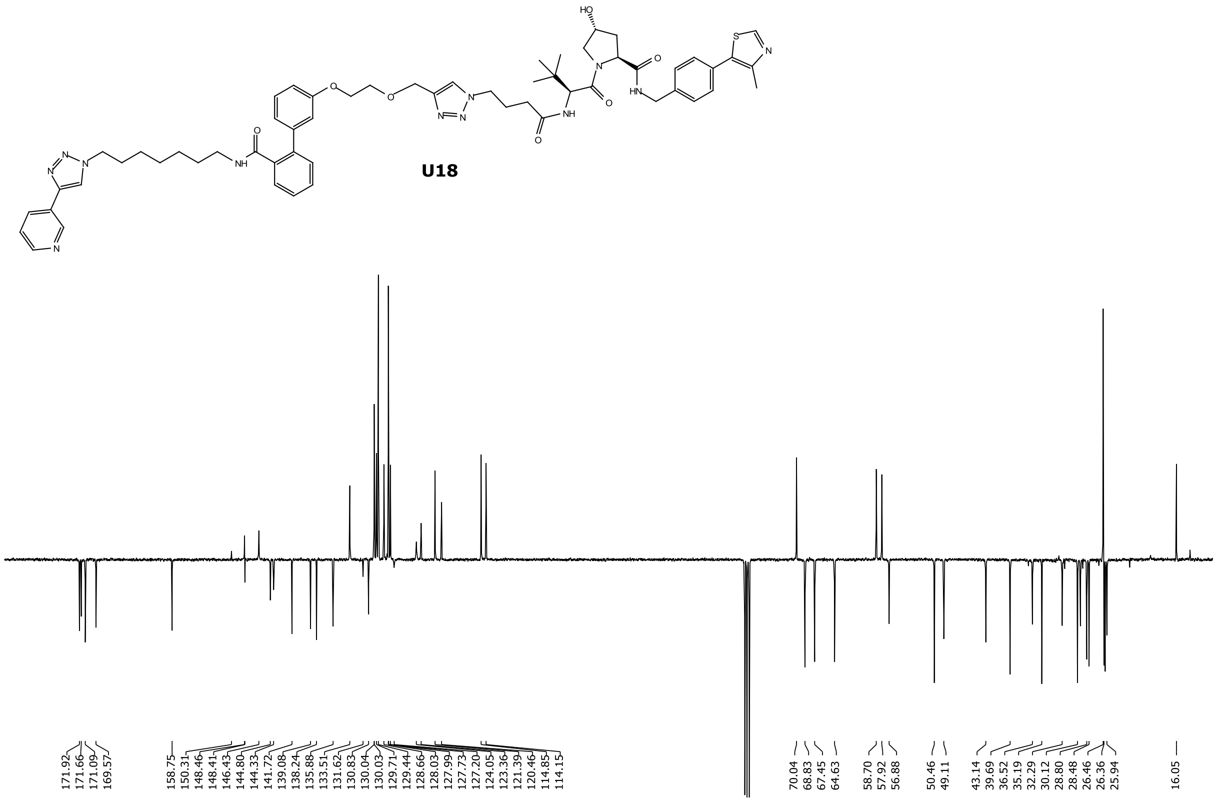

Figure S10. ^{13}C APT NMR spectrum of compound **U18**.

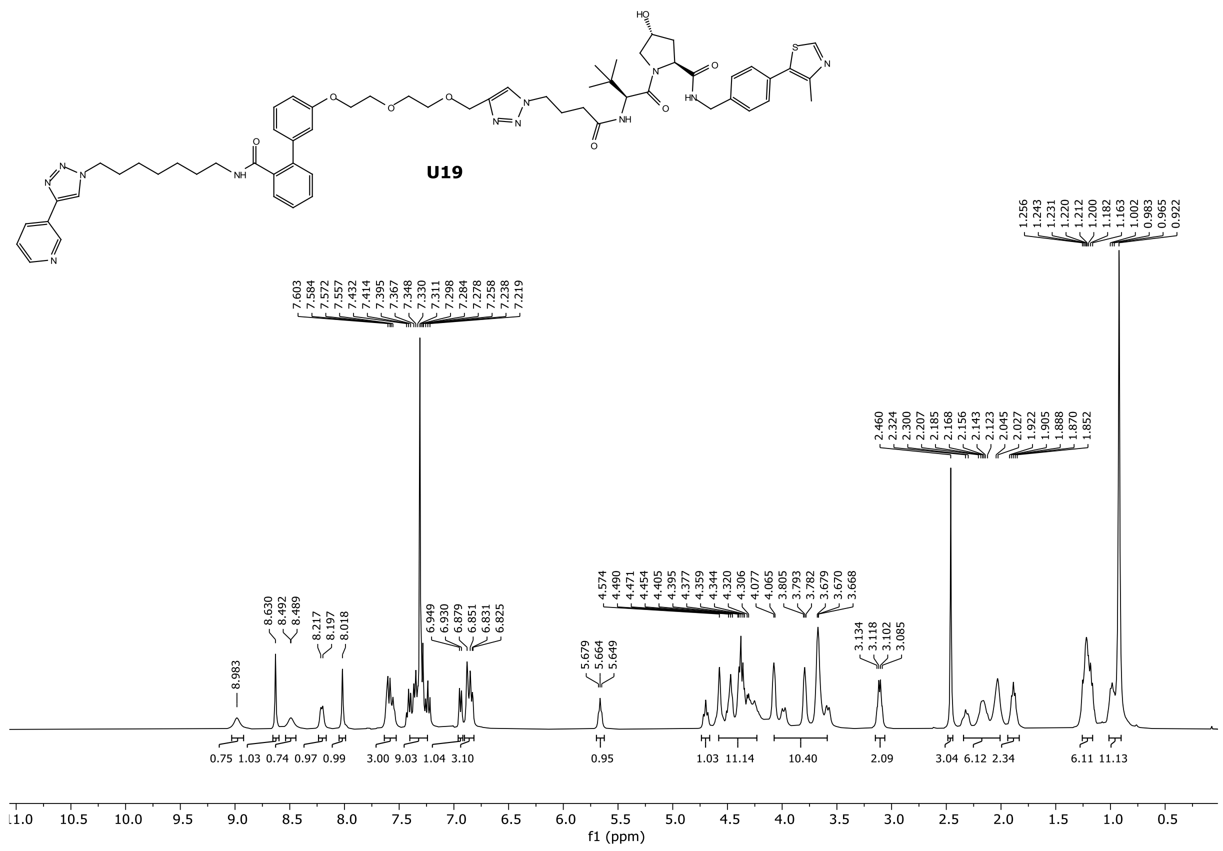

Figure S11. ^{1}H NMR spectrum of compound **U19**.

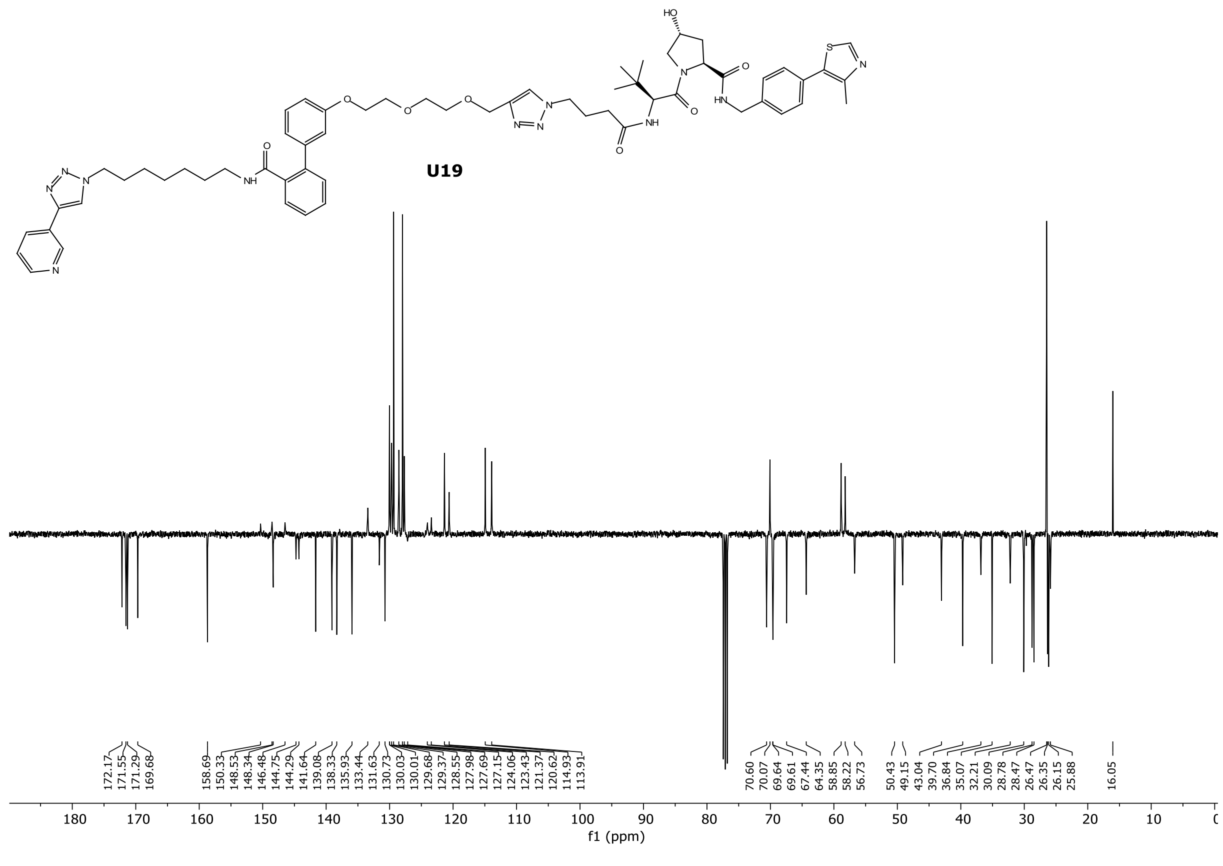

Figure S12. ^{13}C APT NMR spectrum of compound **U19**.

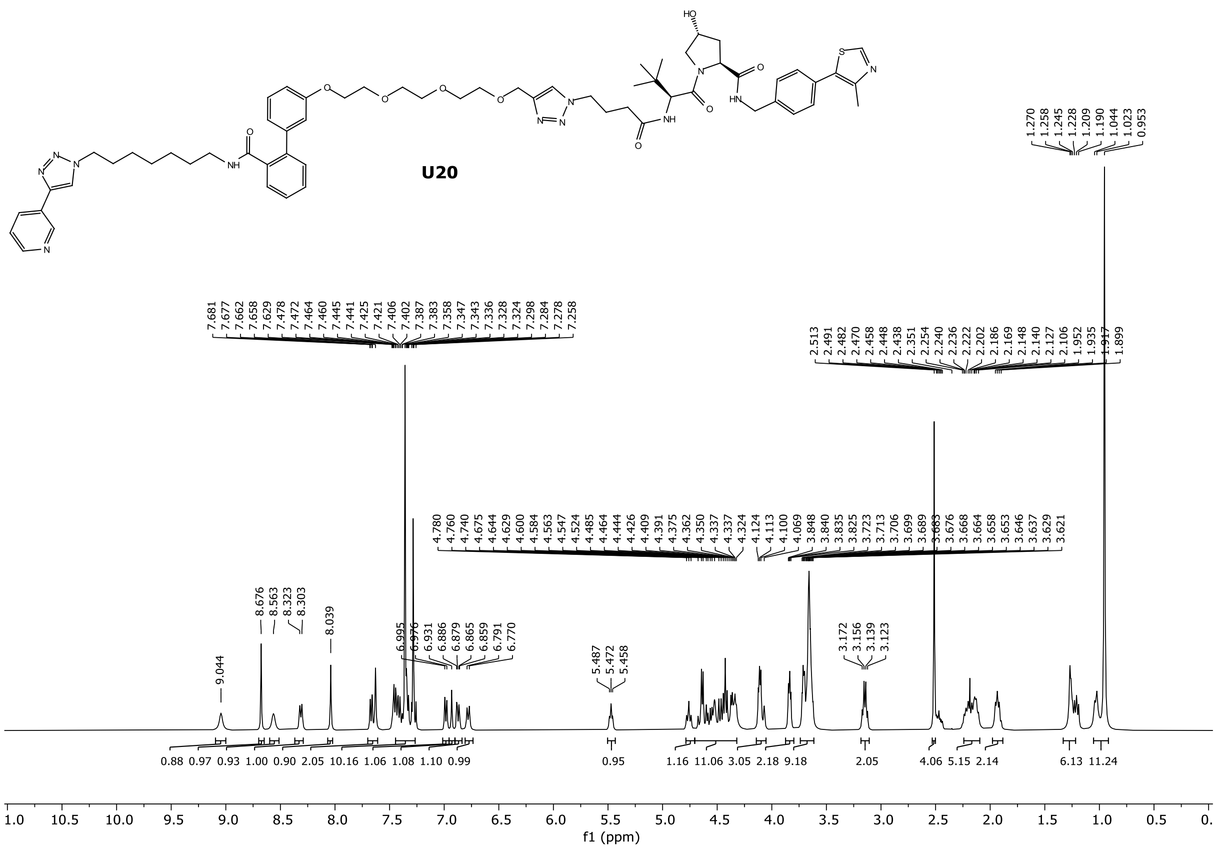

Figure S13. ^{1}H NMR spectrum of compound **U20**.

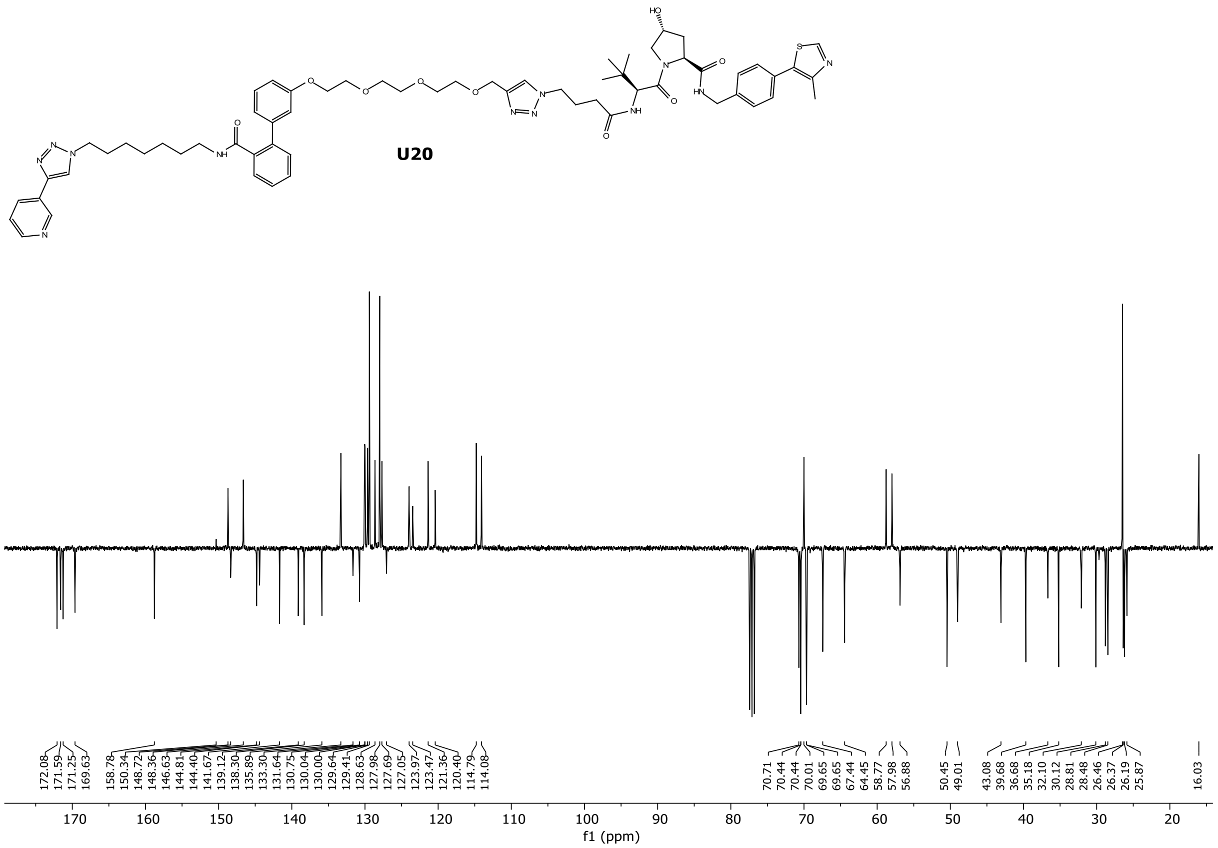

Figure S14. ^{13}C APT NMR spectrum of compound **U20**.

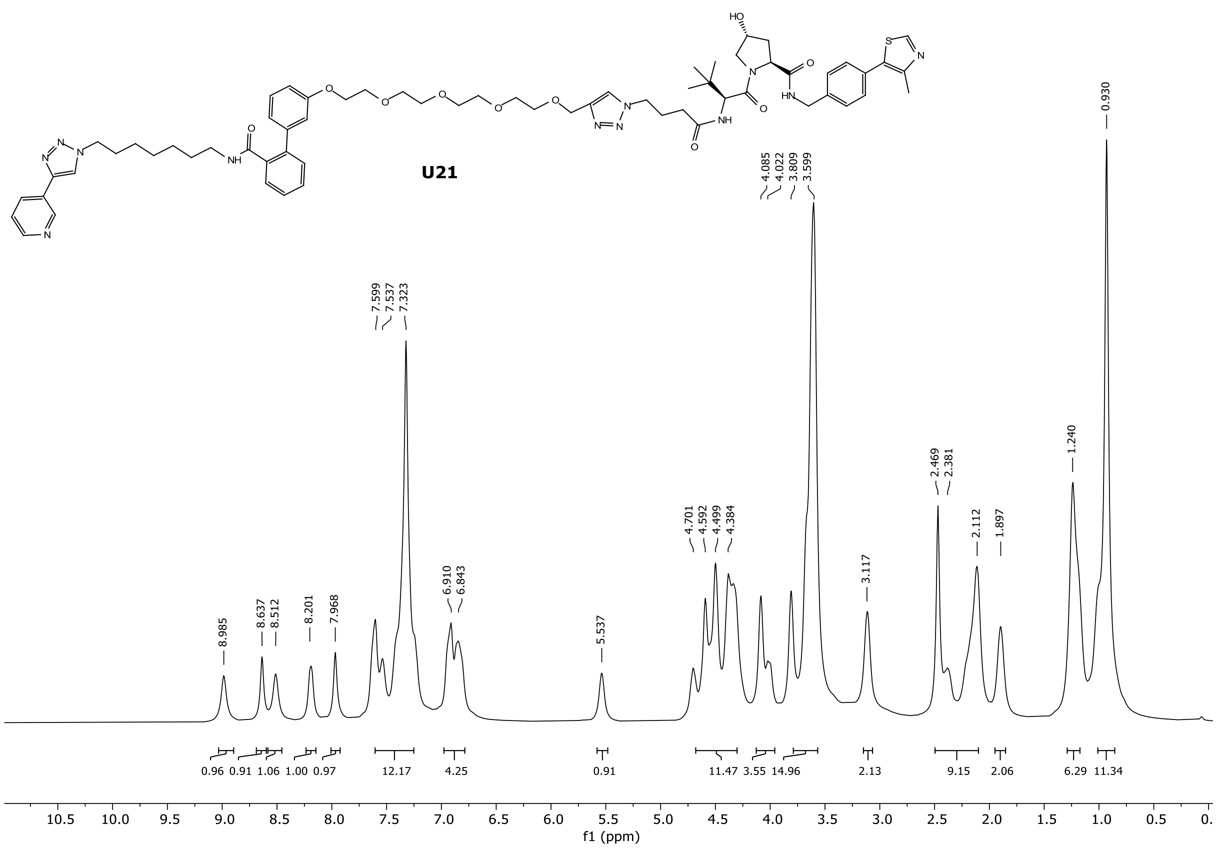

Figure S15. ^{1}H NMR spectrum of compound **U21**.

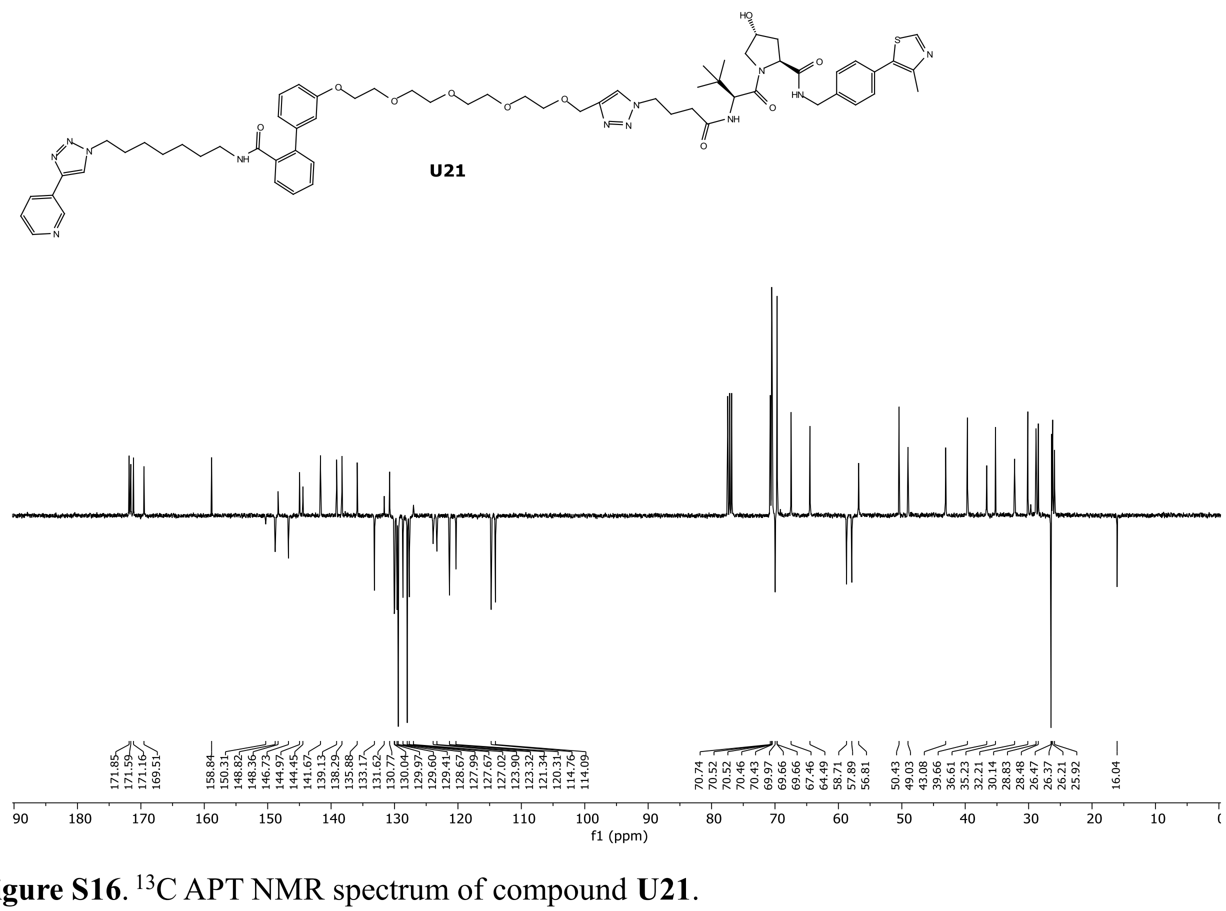

Figure S16. ^{13}C APT NMR spectrum of compound **U21**.

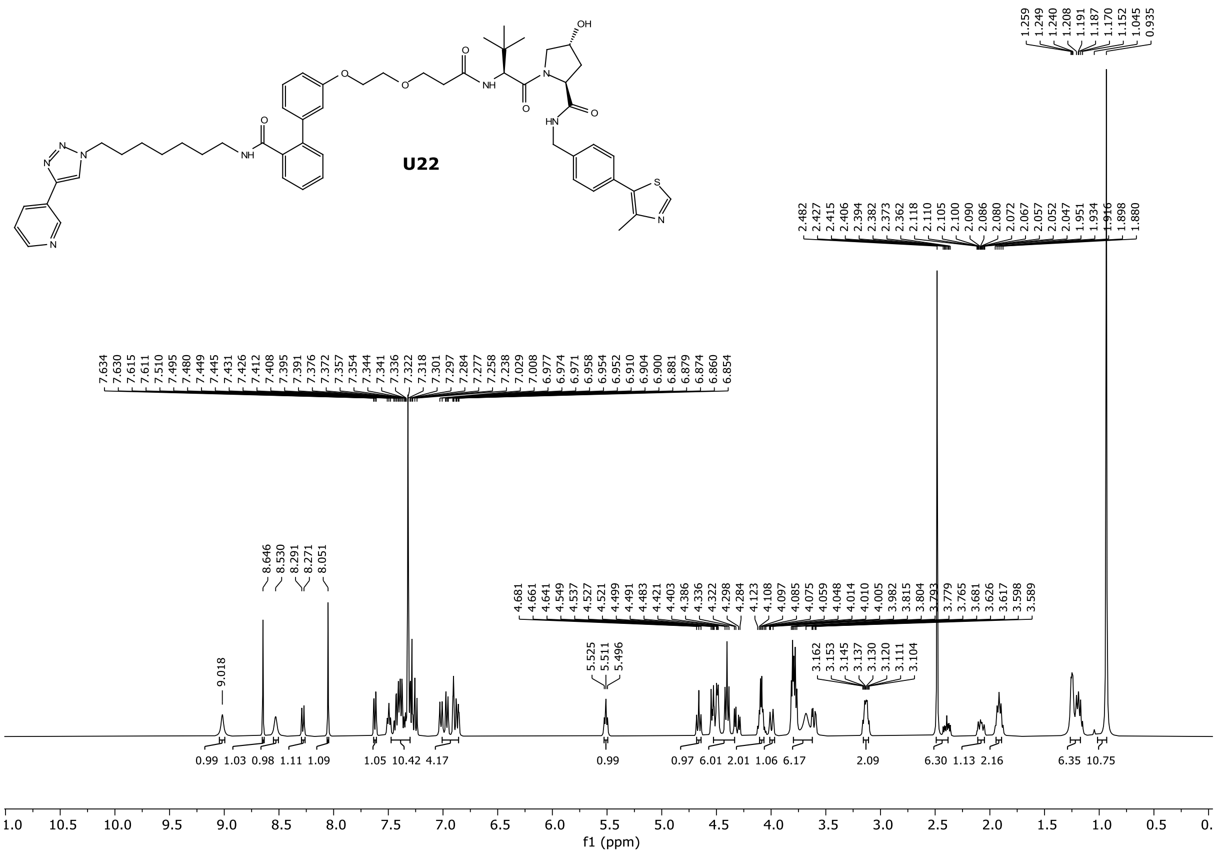

Figure S17. ^{1}H NMR spectrum of compound **U22**.

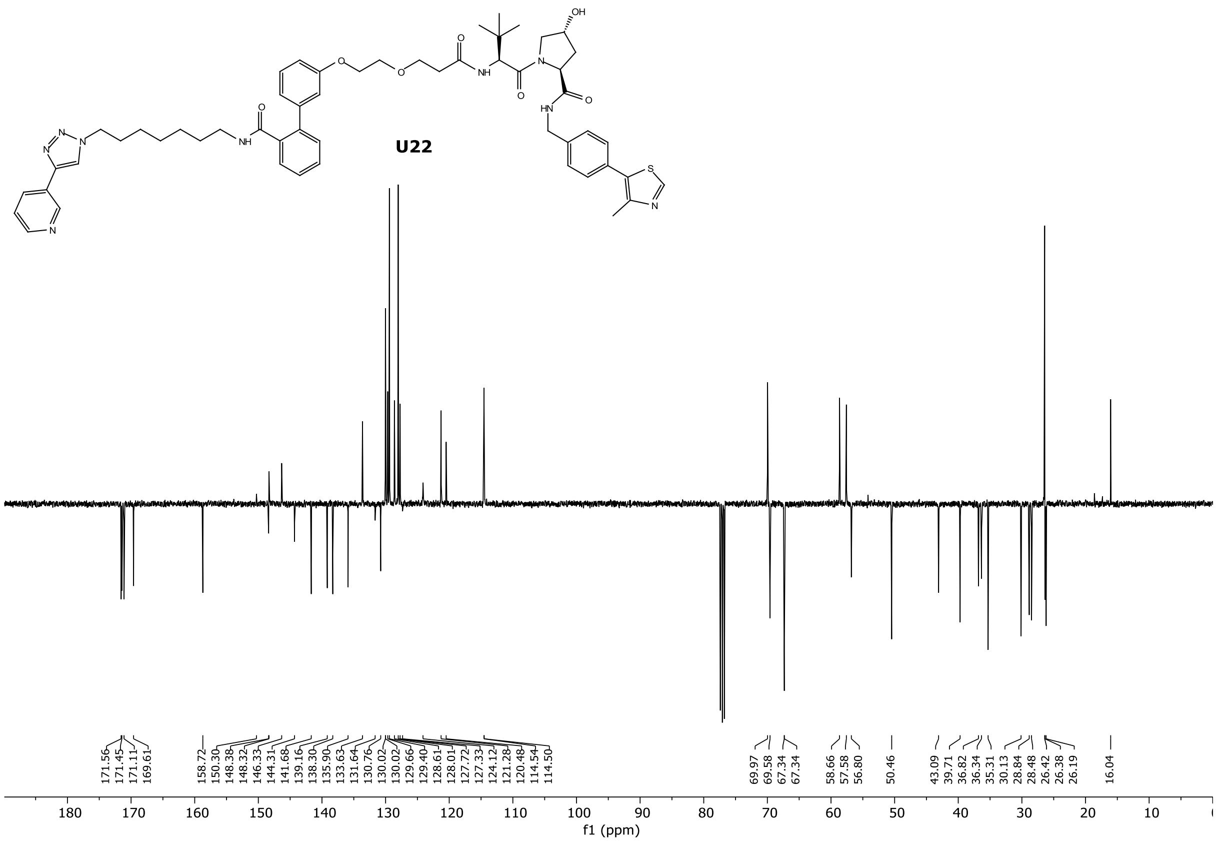

Figure S18. ^{13}C APT NMR spectrum of compound **U22**.

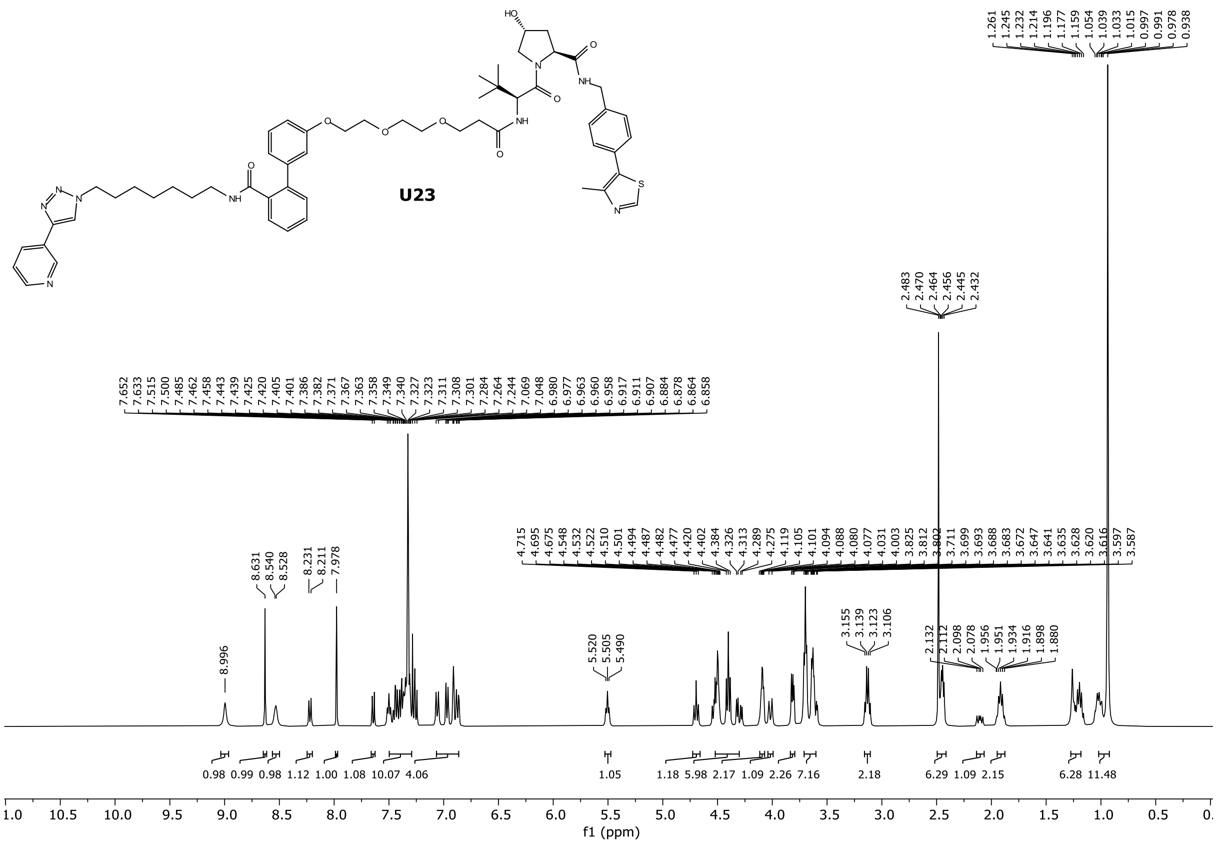

Figure S19. ^{1}H NMR spectrum of compound **U23**.

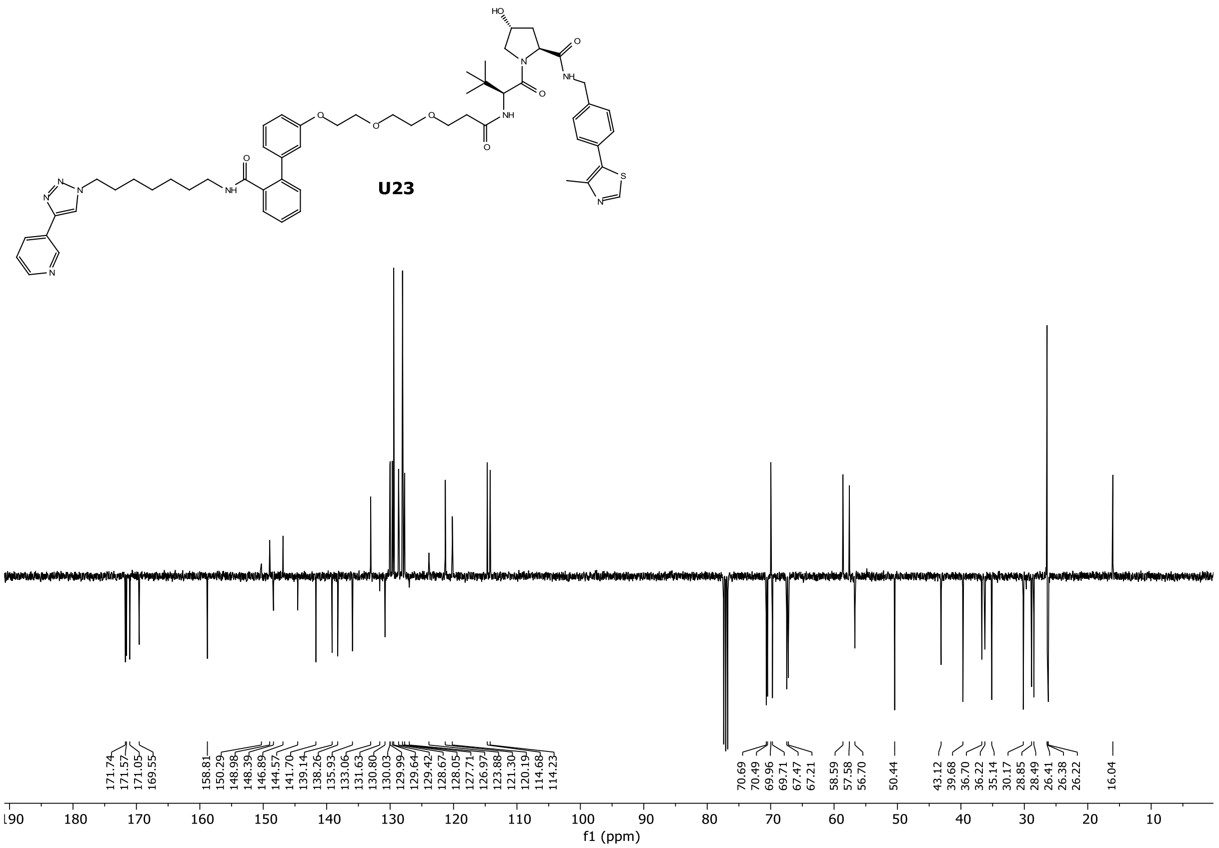

Figure S20. ^{13}C APT NMR spectrum of compound **U23**.

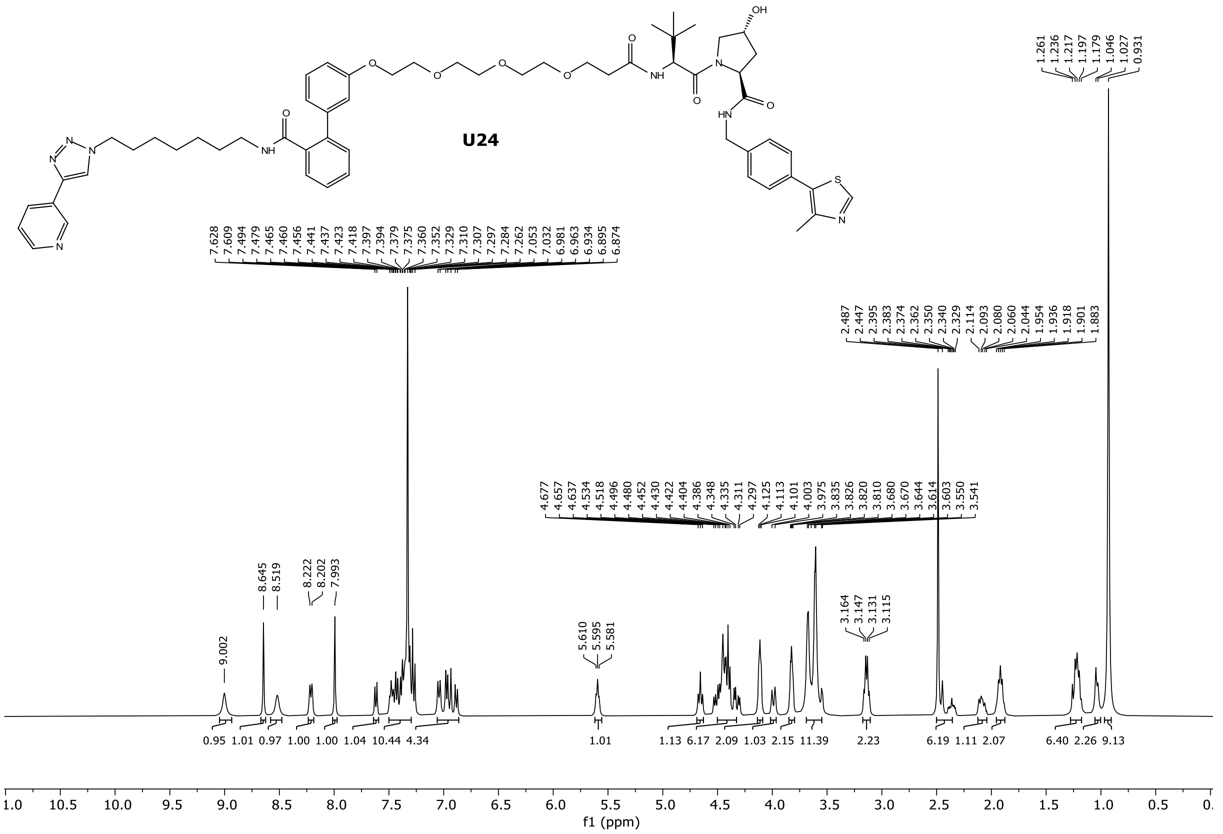

Figure S21. ^{1}H NMR spectrum of compound **U24**.

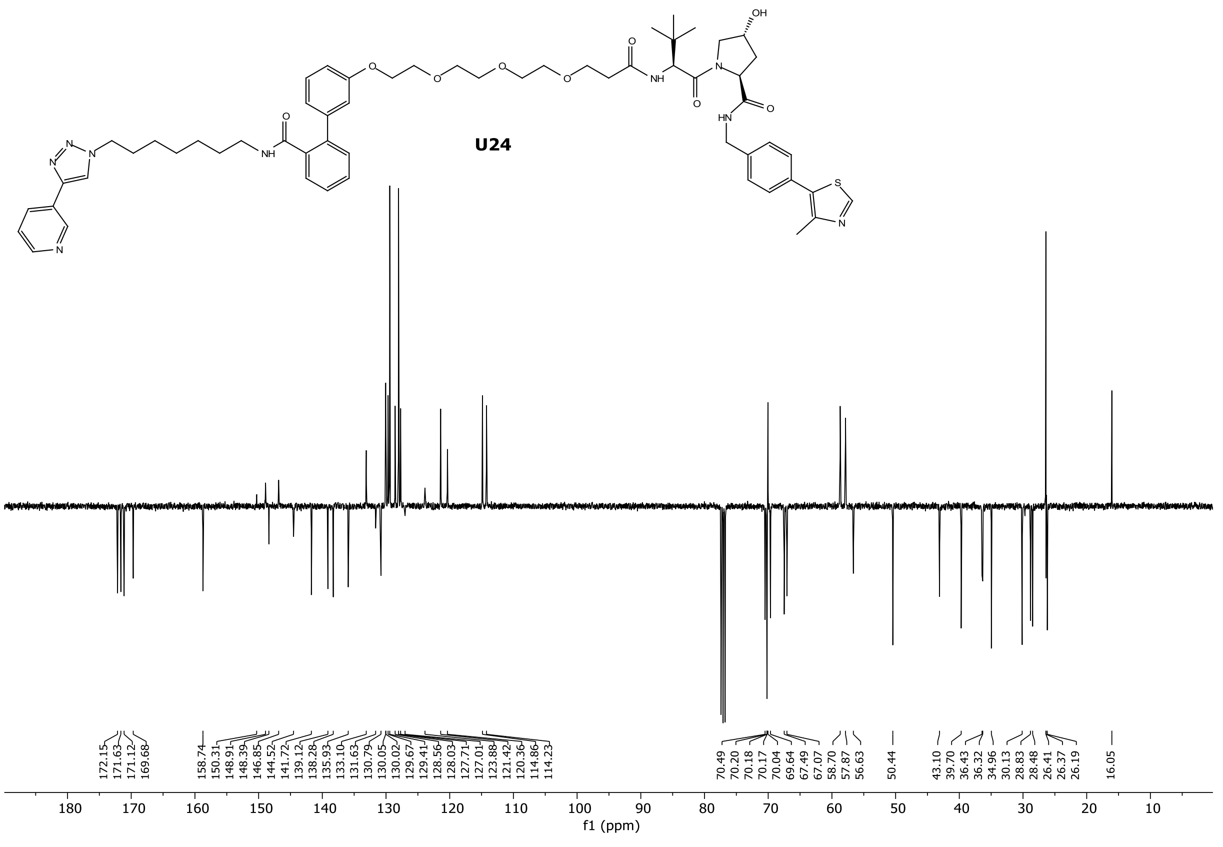

Figure S22. ^{13}C APT NMR spectrum of compound **U24**.

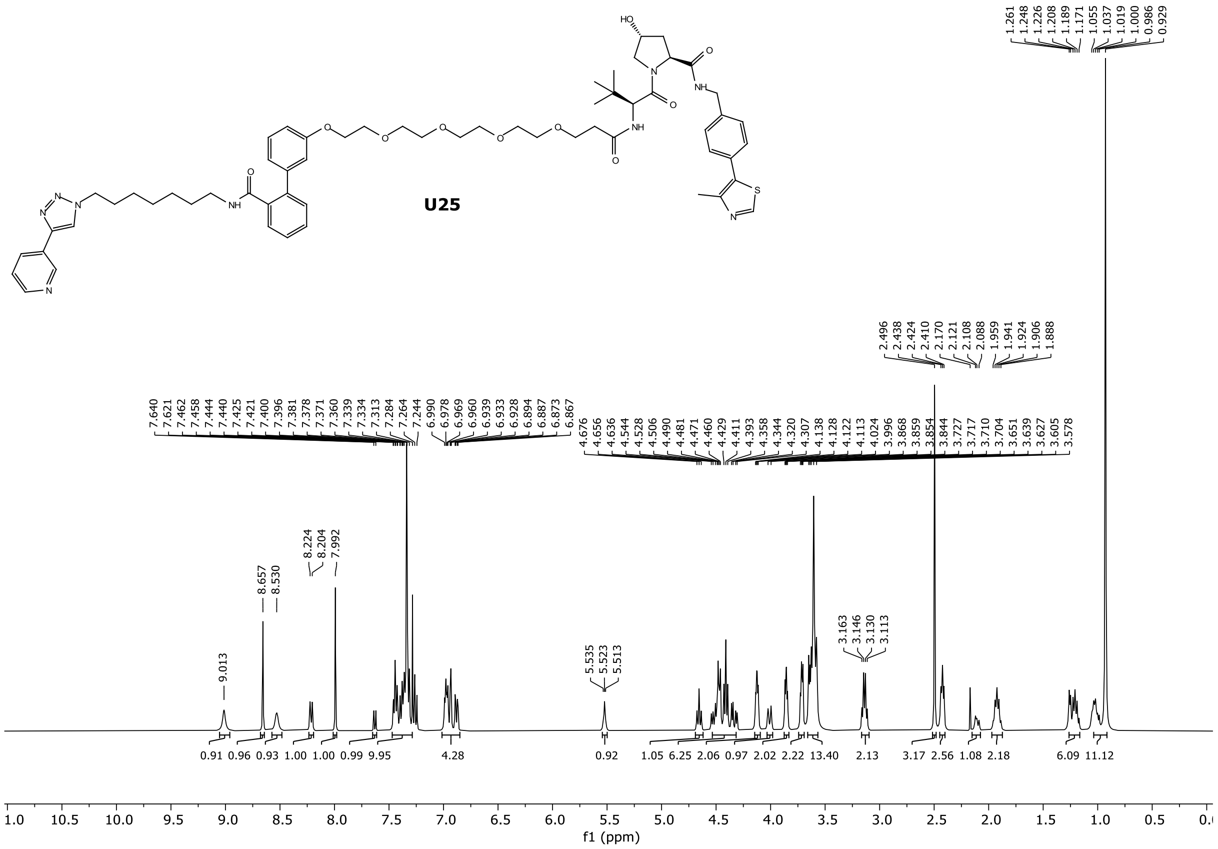

Figure S23. ^{1}H NMR spectrum of compound **U25**.

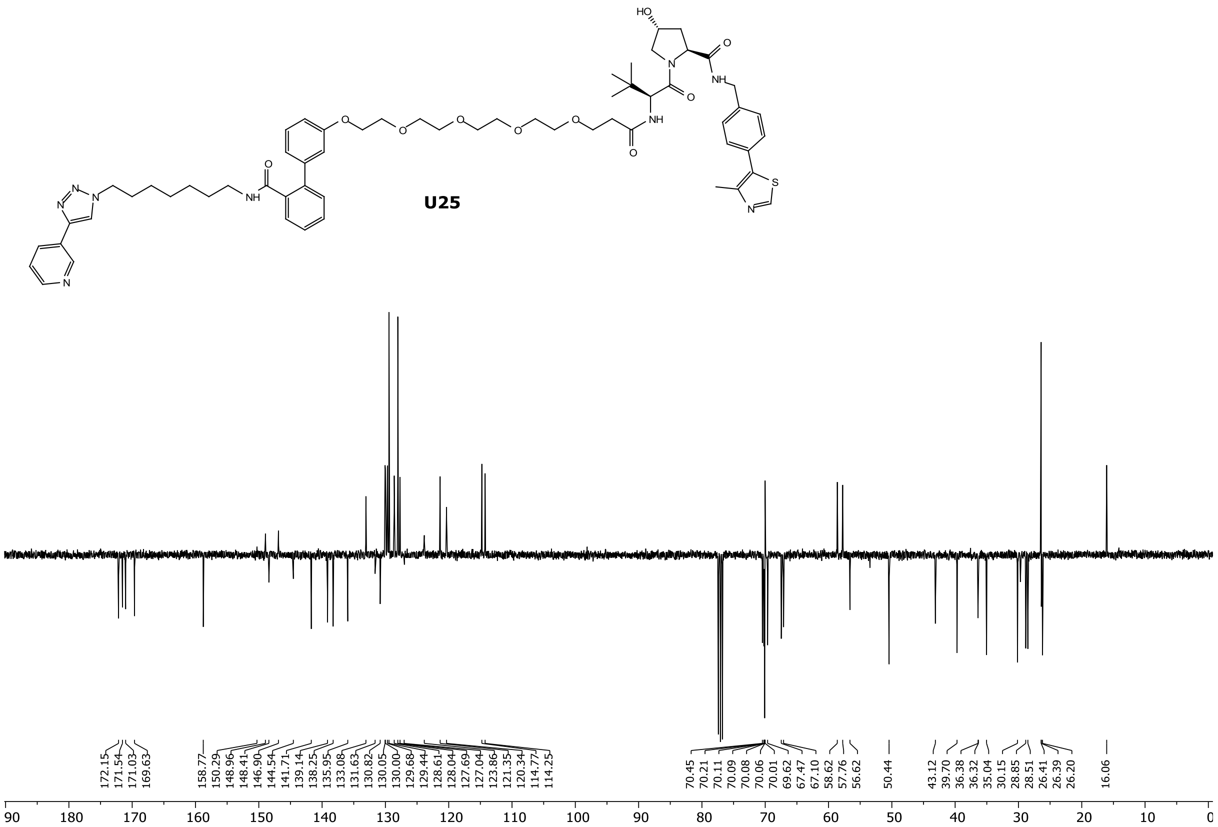

Figure S24. ^{13}C APT NMR spectrum of compound **U25**.

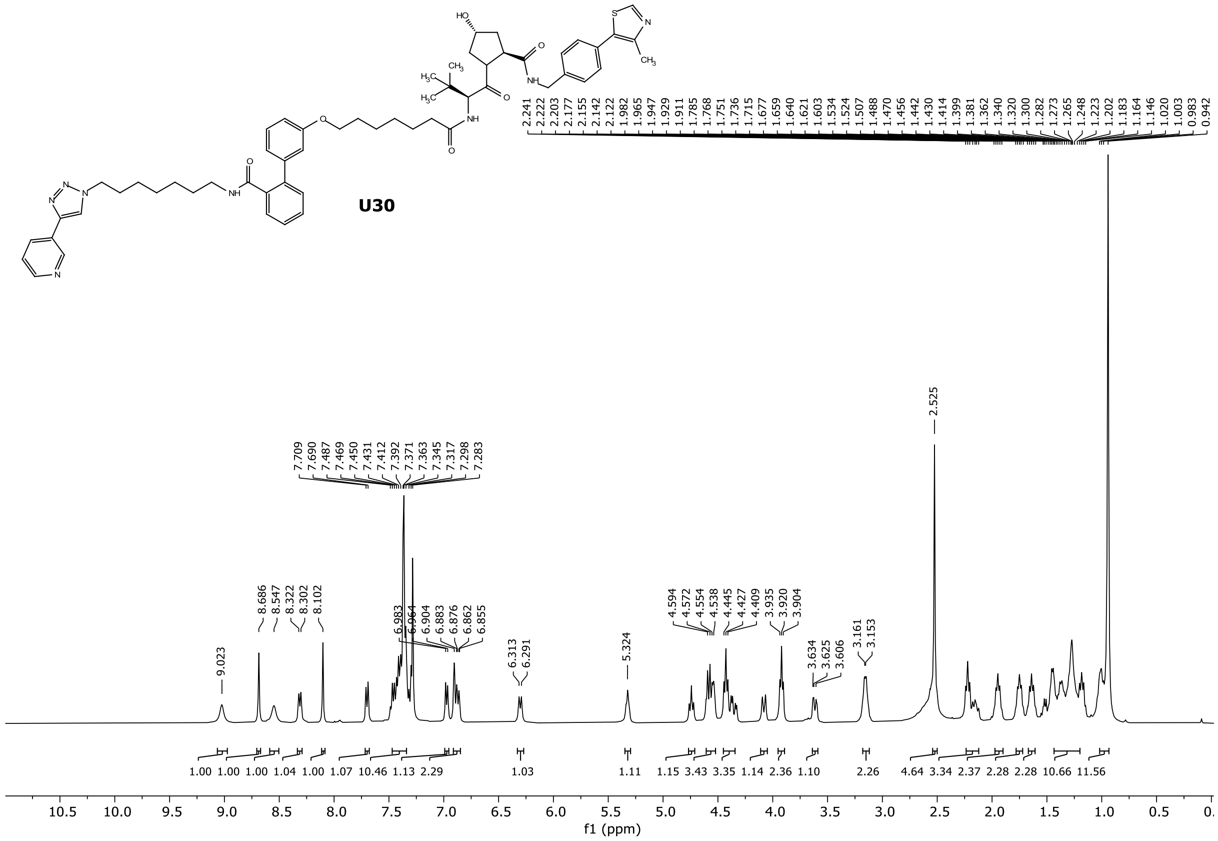

Figure S25. ^{1}H NMR spectrum of compound **U30**.

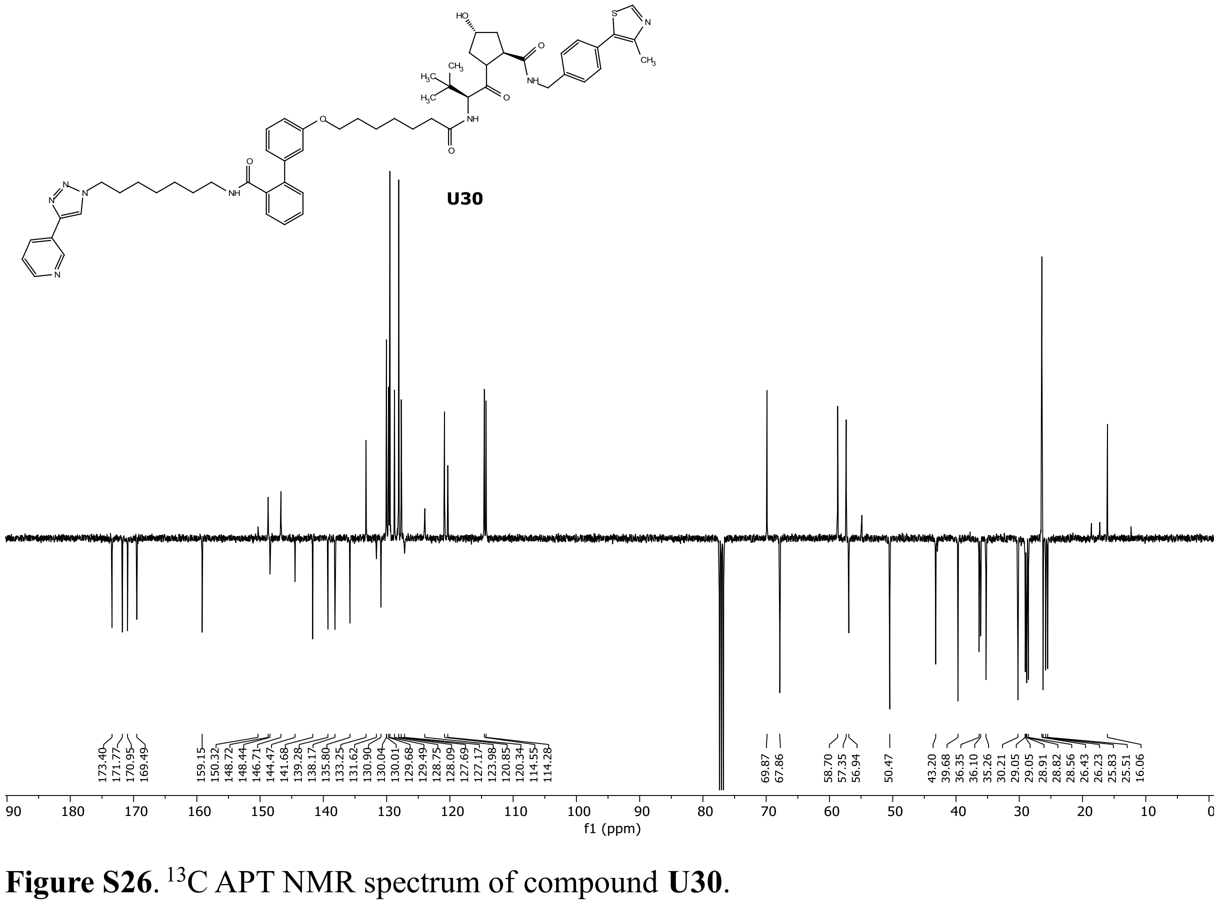

Figure S26. ^{13}C APT NMR spectrum of compound **U30**.

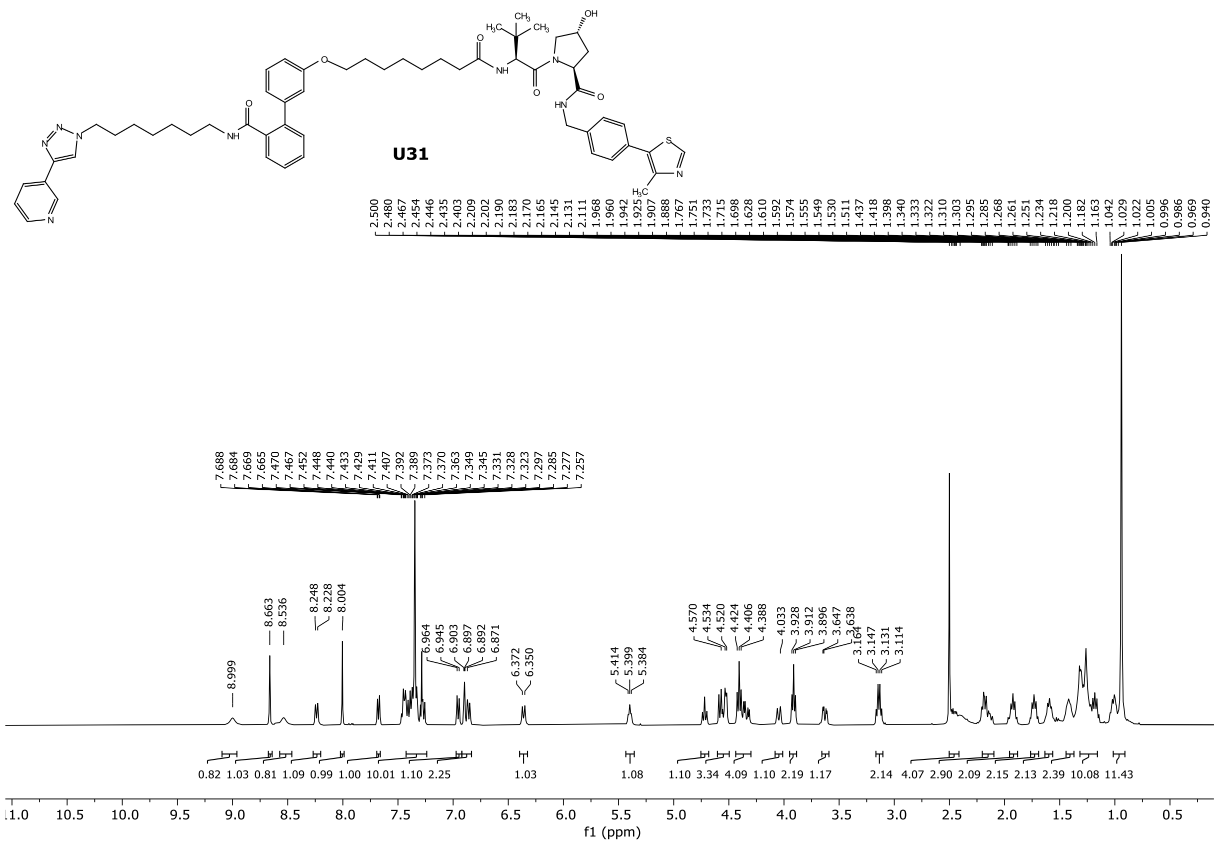

Figure S27. ^{1}H NMR spectrum of compound **U31**.

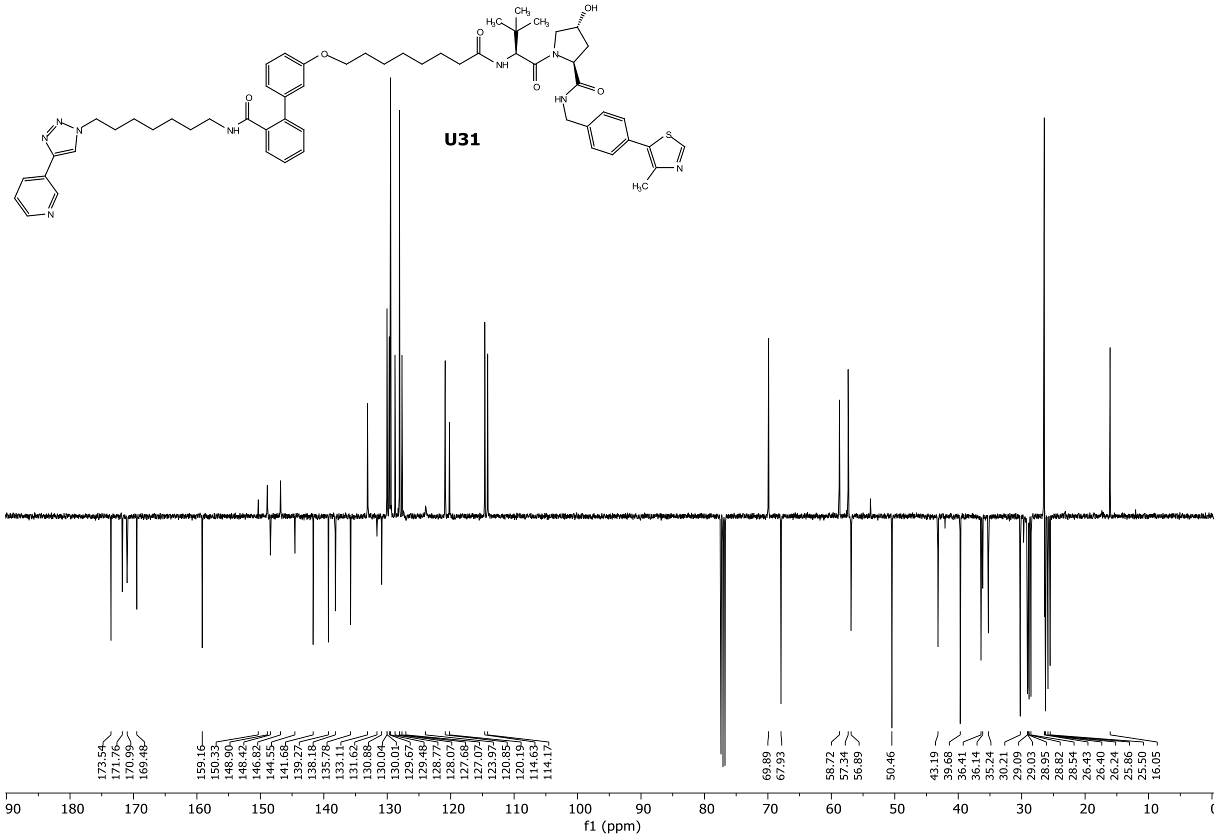

Figure S28. ^{13}C APT NMR spectrum of compound **U31**.

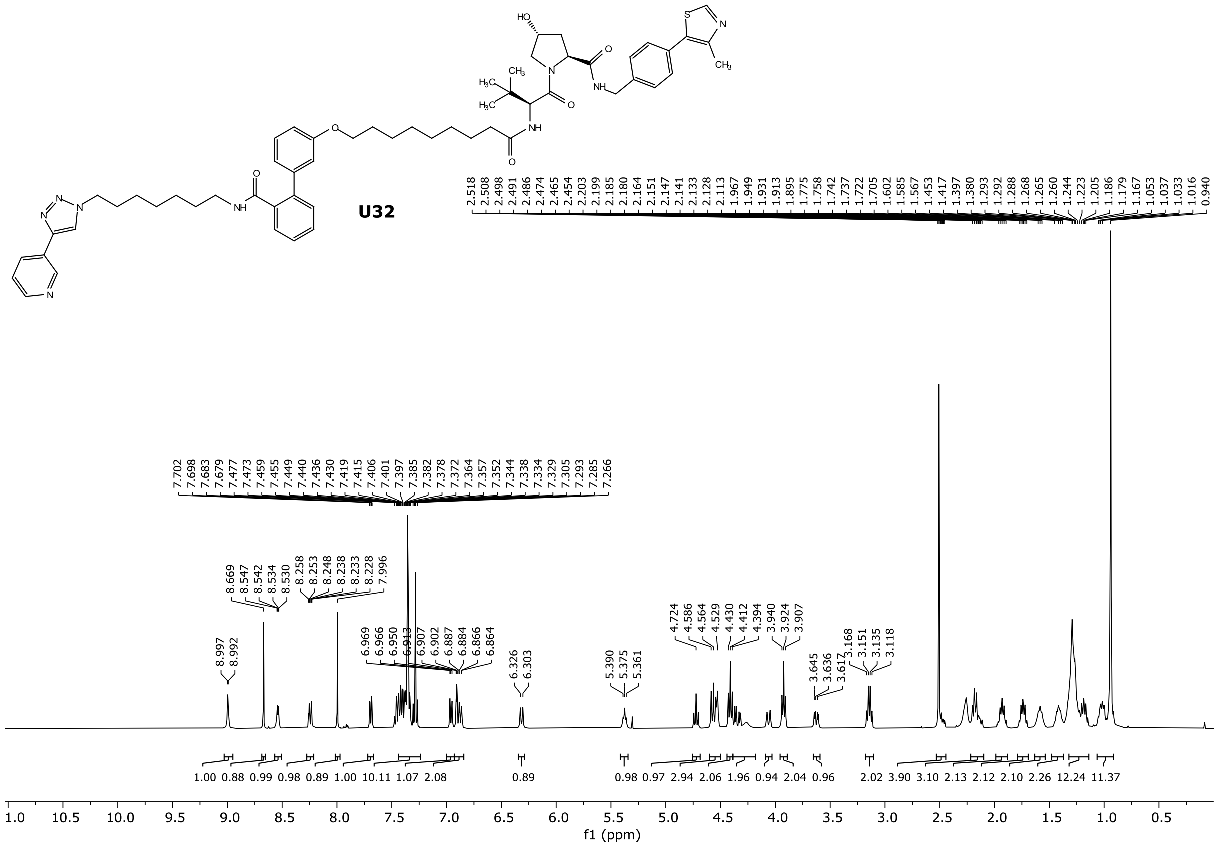

Figure S29. ^{1}H NMR spectrum of compound **U32**.

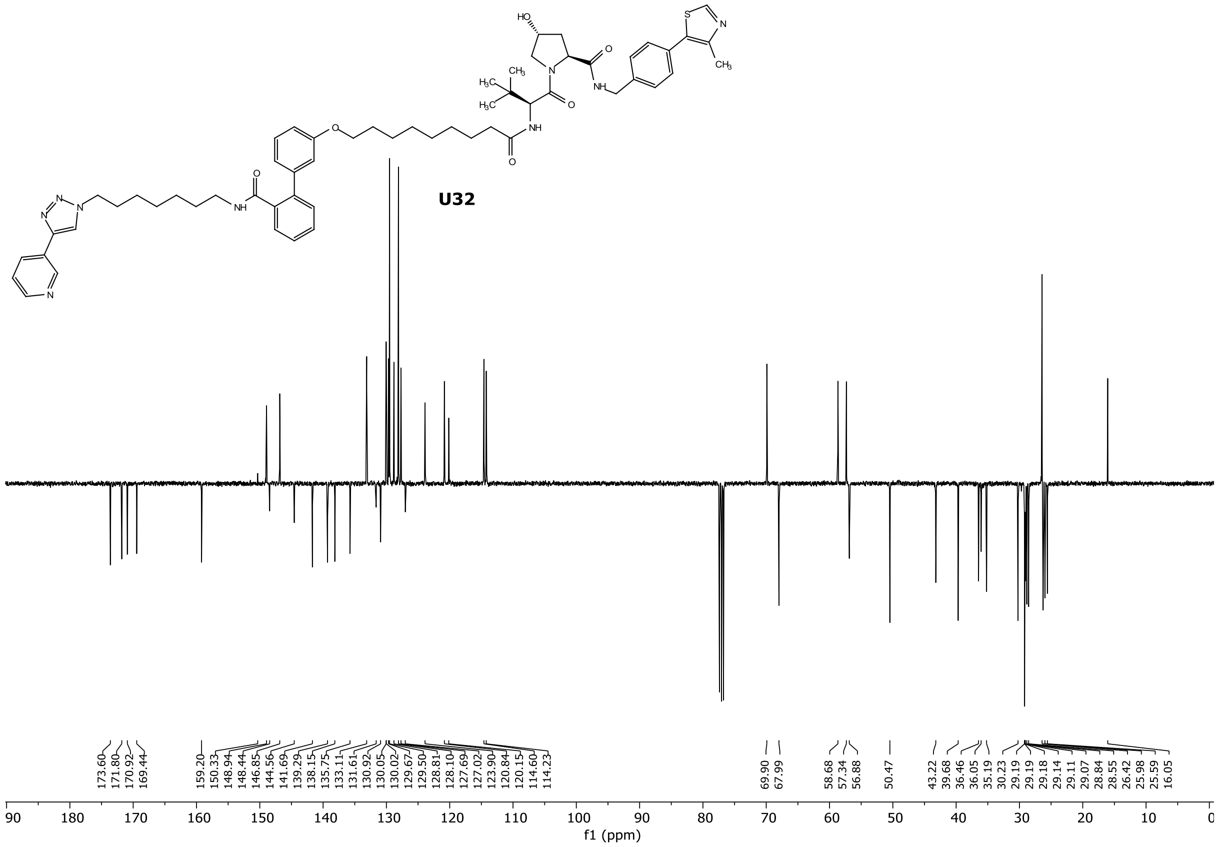

Figure S30. ^{13}C APT NMR spectrum of compound **U32**.

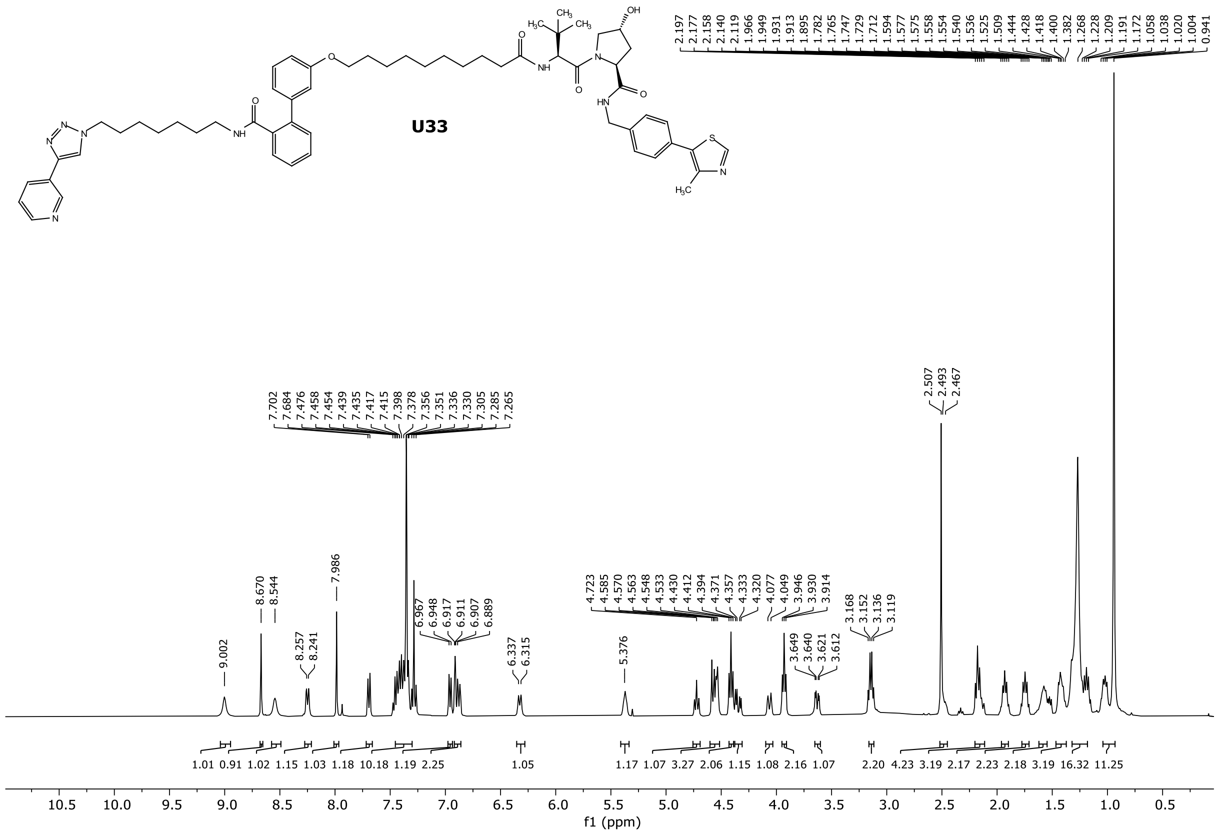

Figure S31. ^{1}H NMR spectrum of compound **U33**.

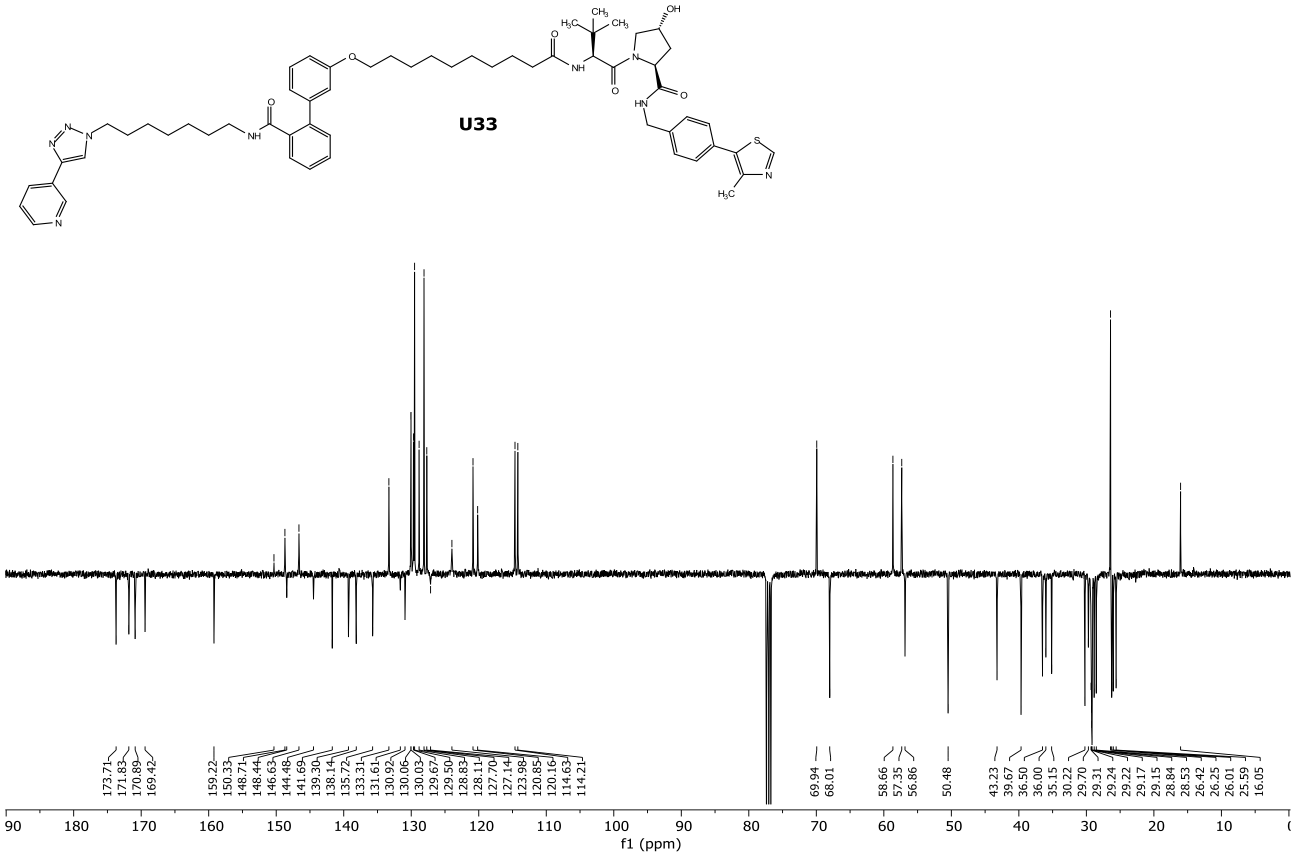

Figure S32. ^{13}C APT NMR spectrum of compound **U33**.

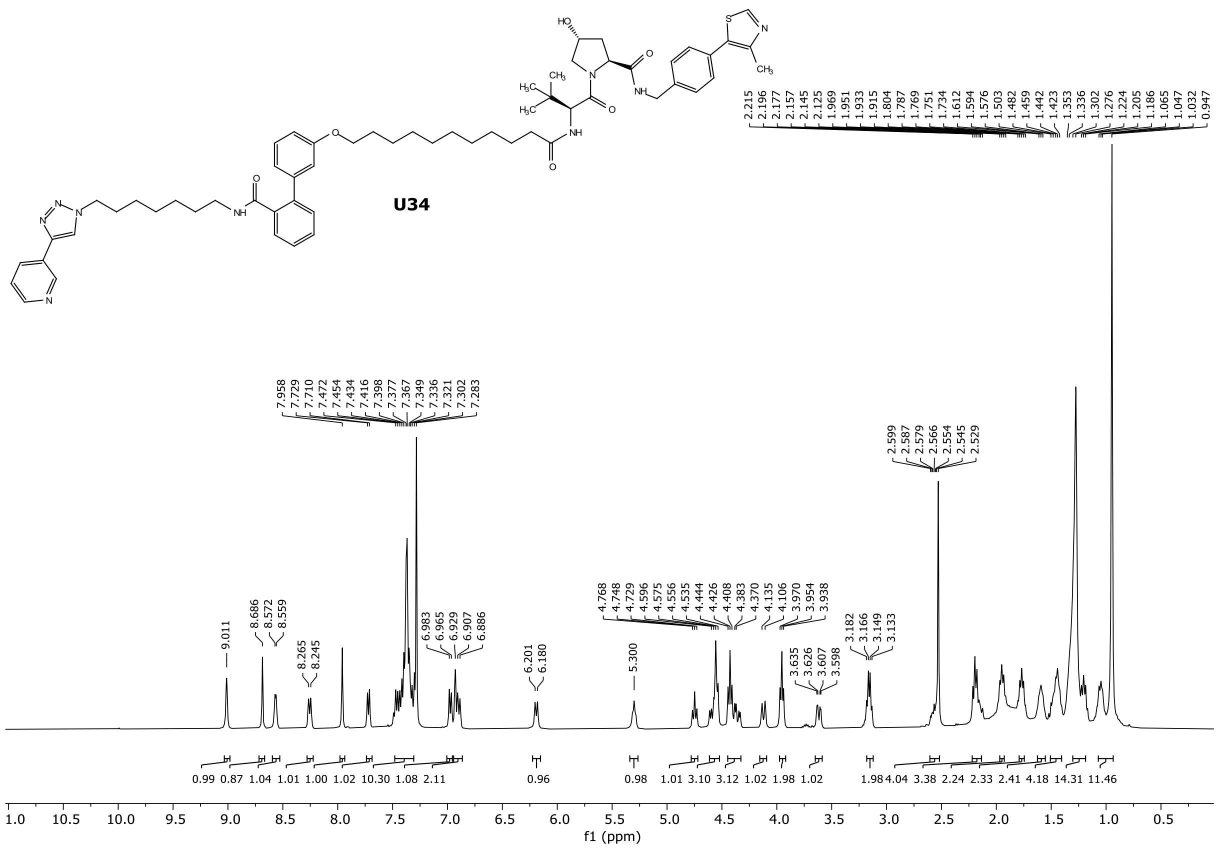

Figure S33. ^{1}H NMR spectrum of compound **U34**.

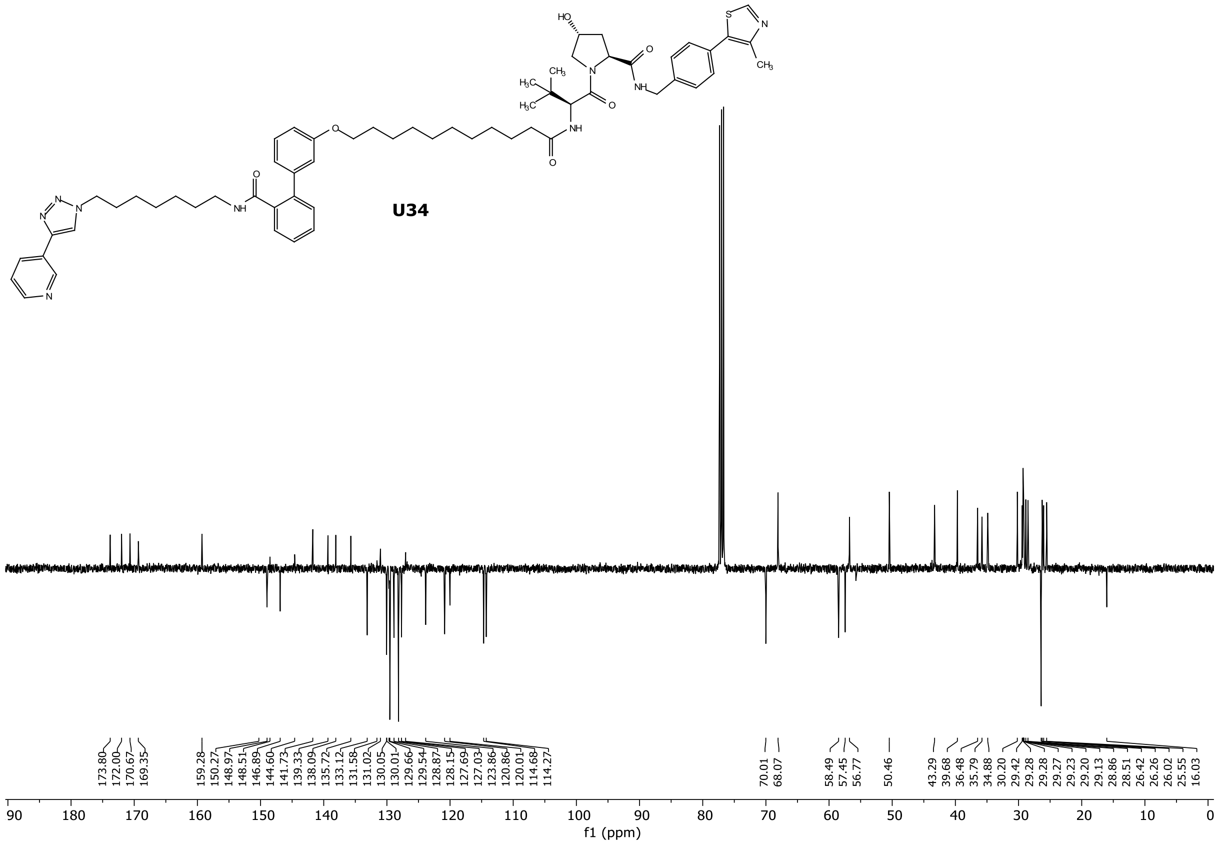

Figure S34. ^{13}C APT NMR spectrum of compound **U34**.

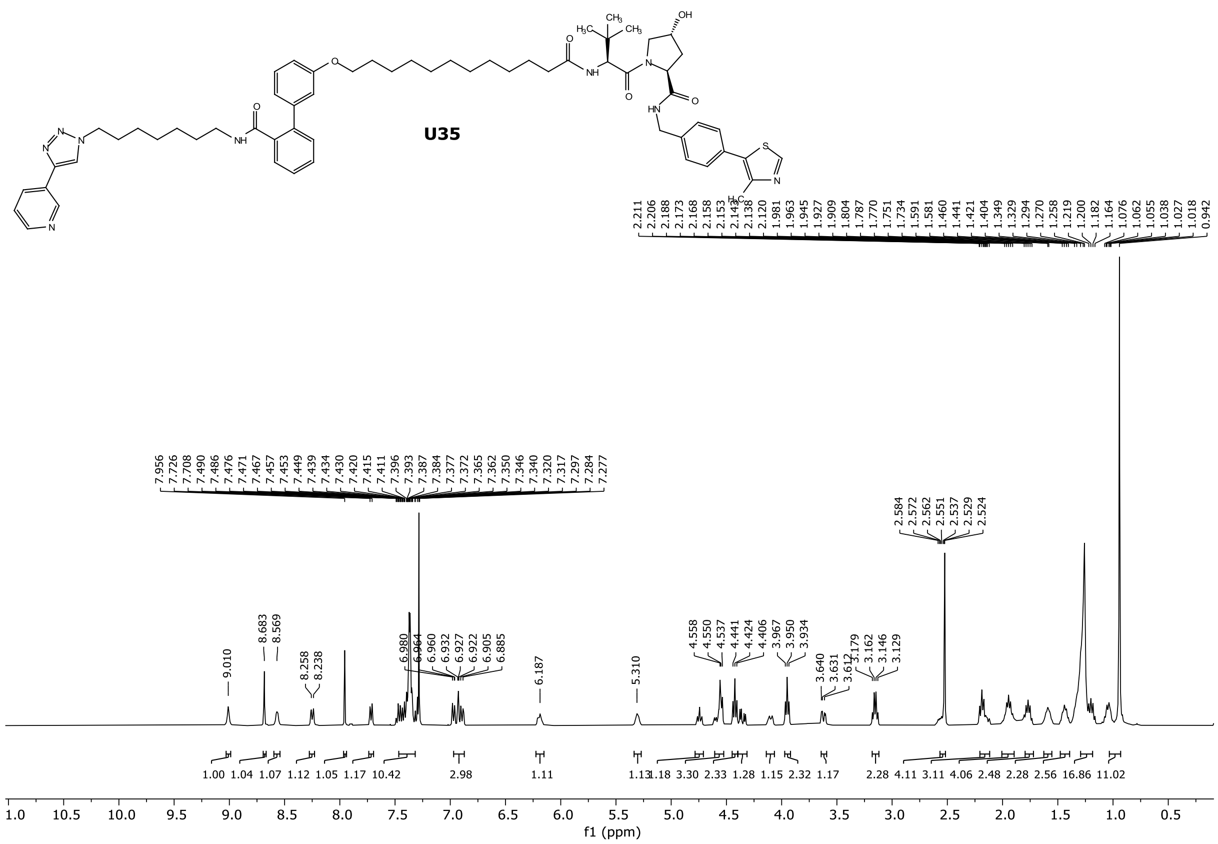

Figure S35. ^{1}H NMR spectrum of compound **U35**.

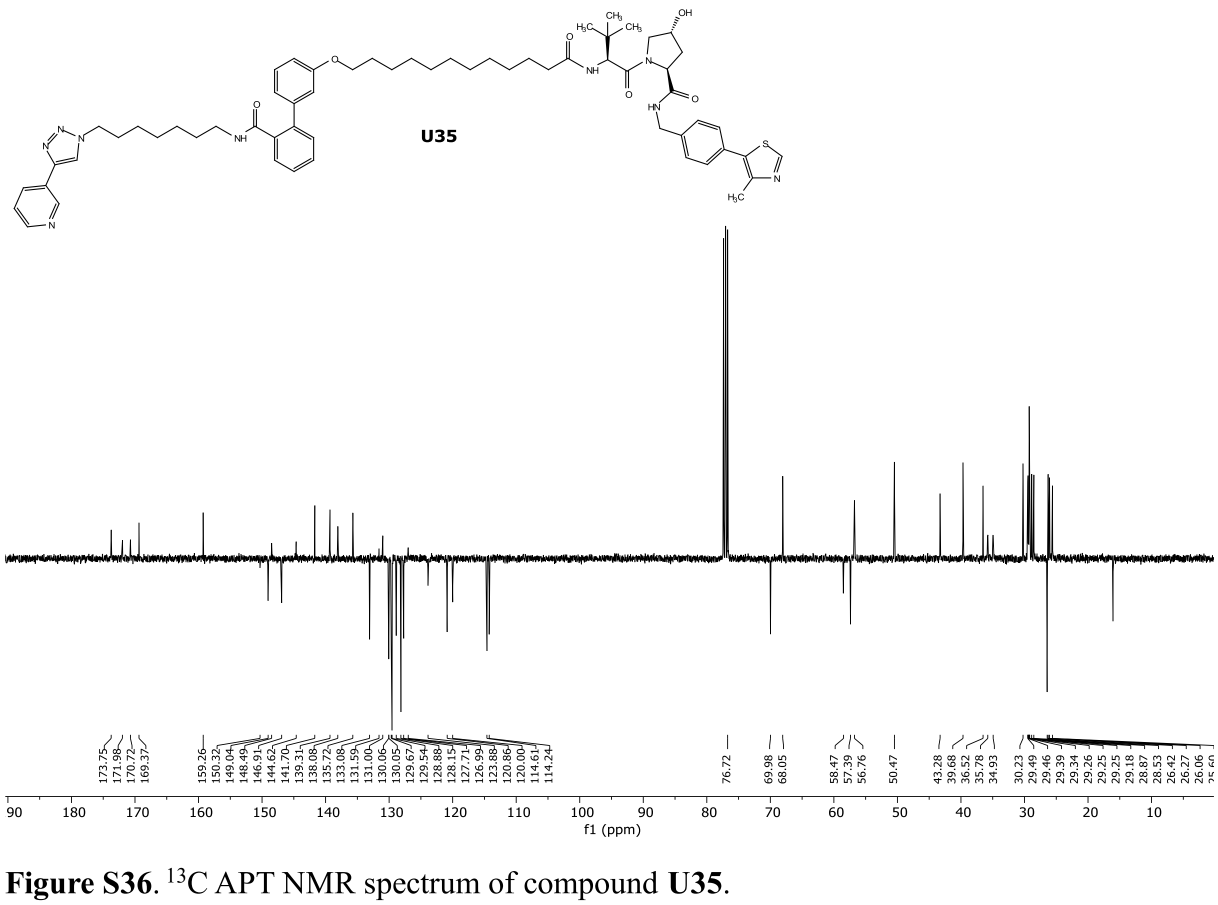

Figure S36. ^{13}C APT NMR spectrum of compound **U35**.

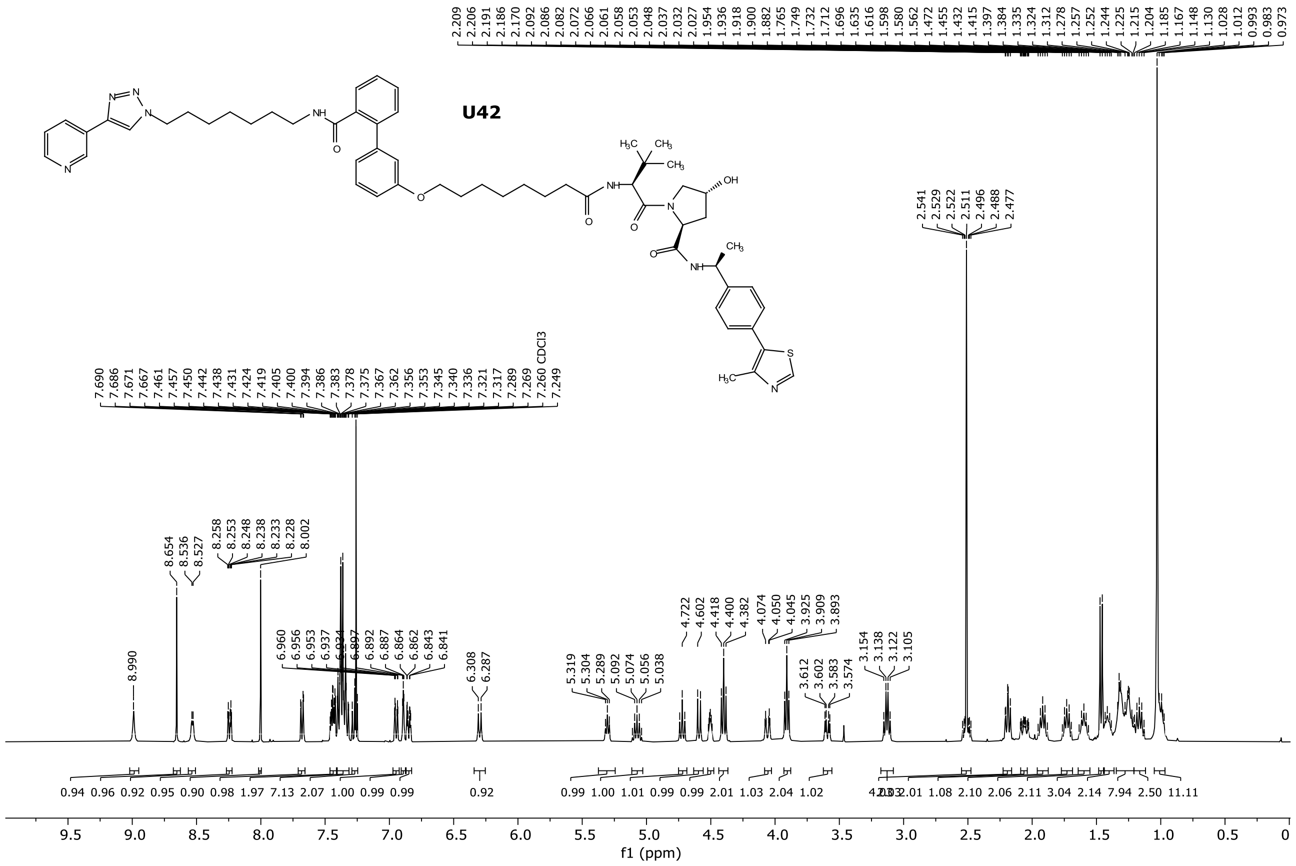

Figure S37. ^{1}H NMR spectrum of compound **U42**.

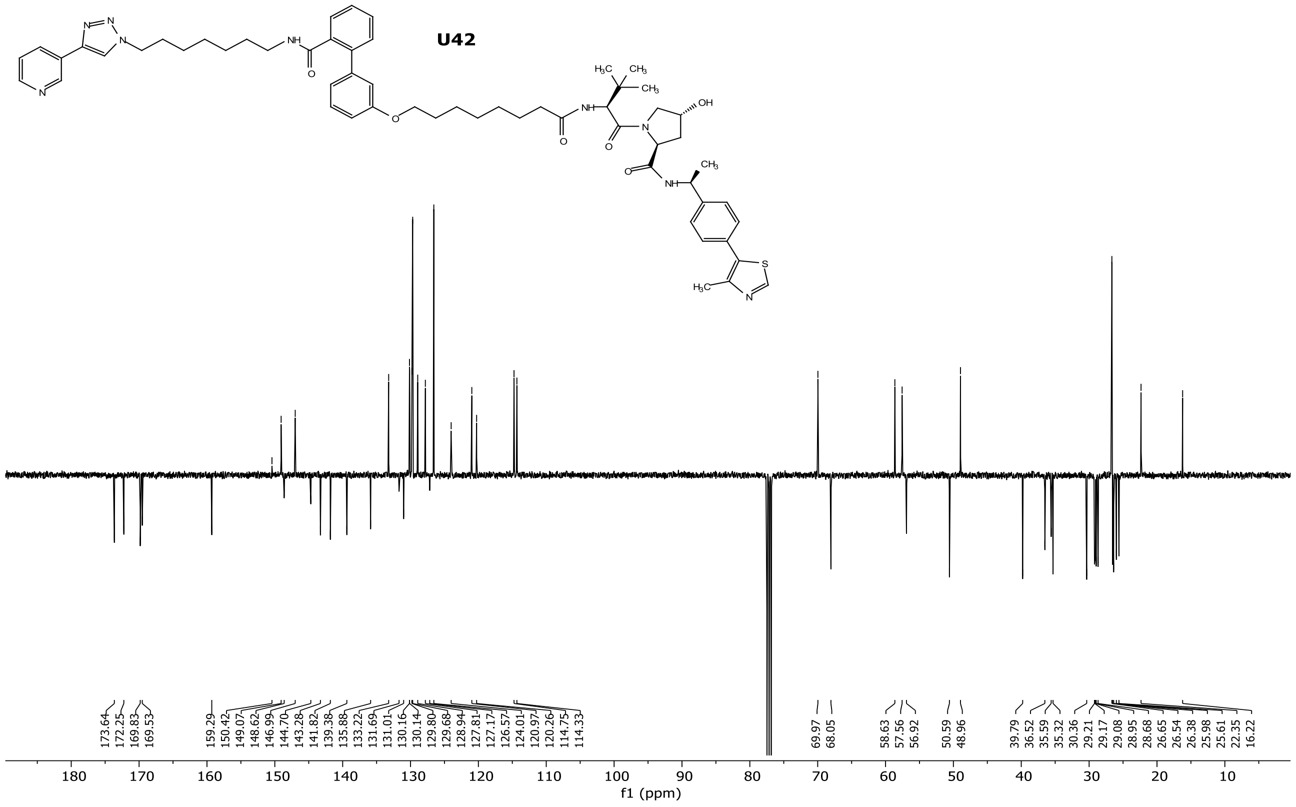

Figure S38. ^{13}C APT NMR spectrum of compound **U42**.

HRMS spectra of PROTACs U14-25, U30-35, U42.

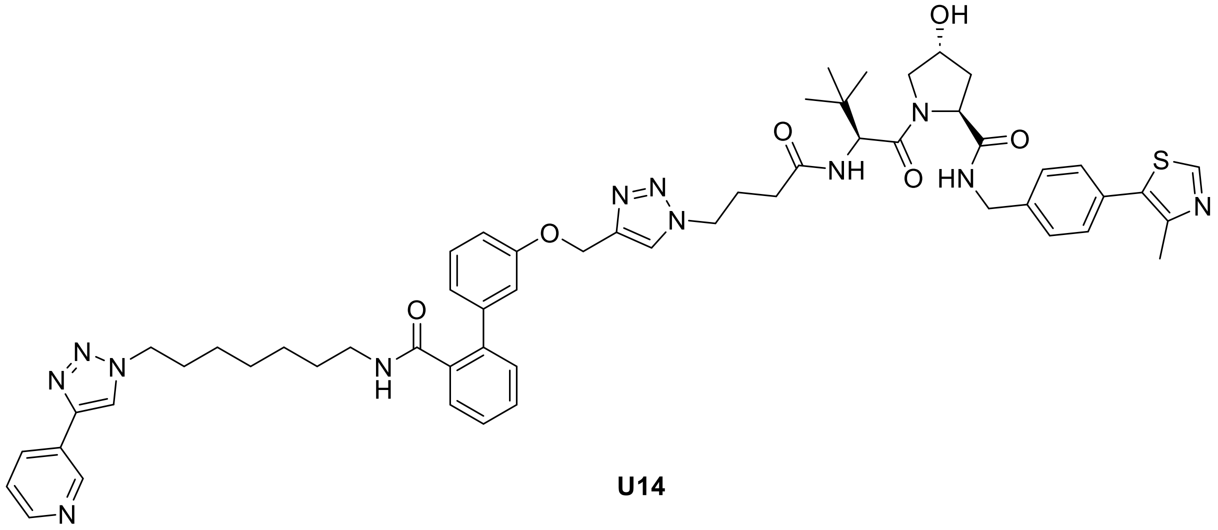

U14

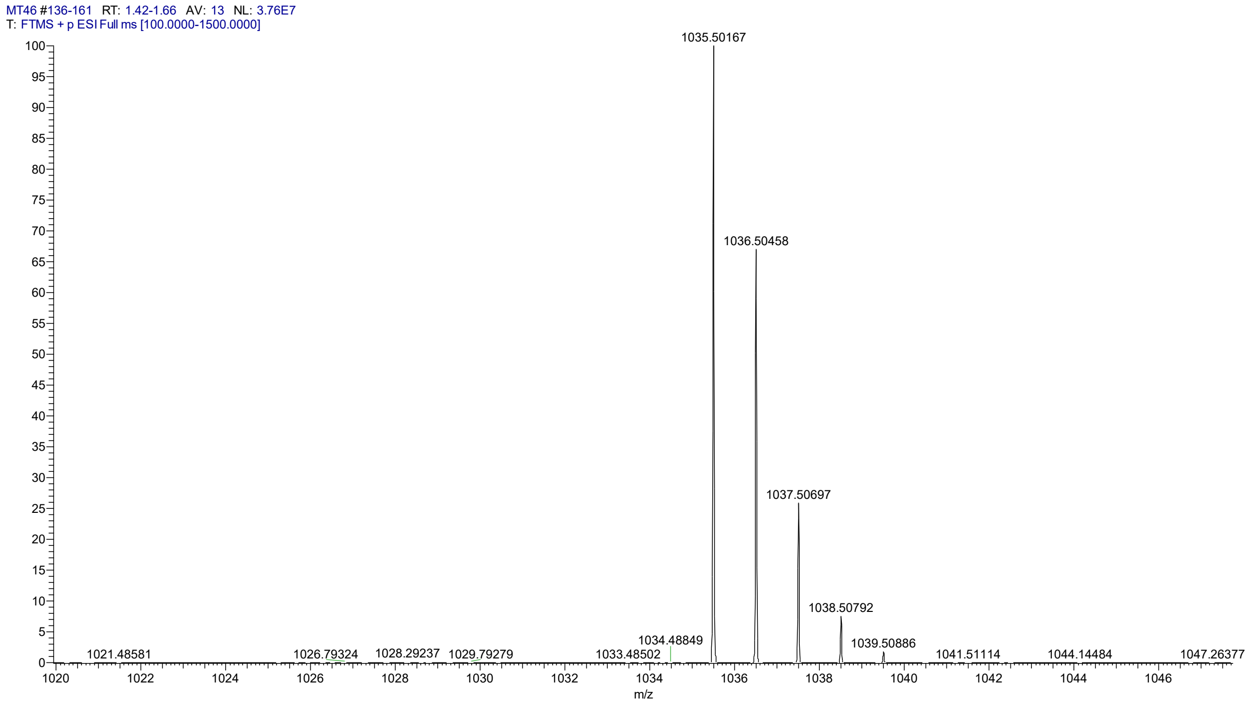

Figure S39. HRMS spectrum of compound **U14**.

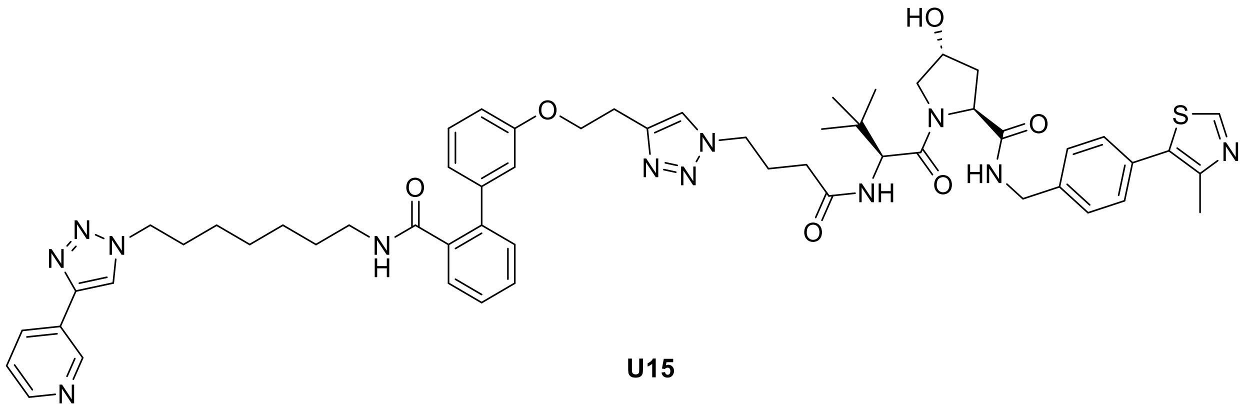

U15

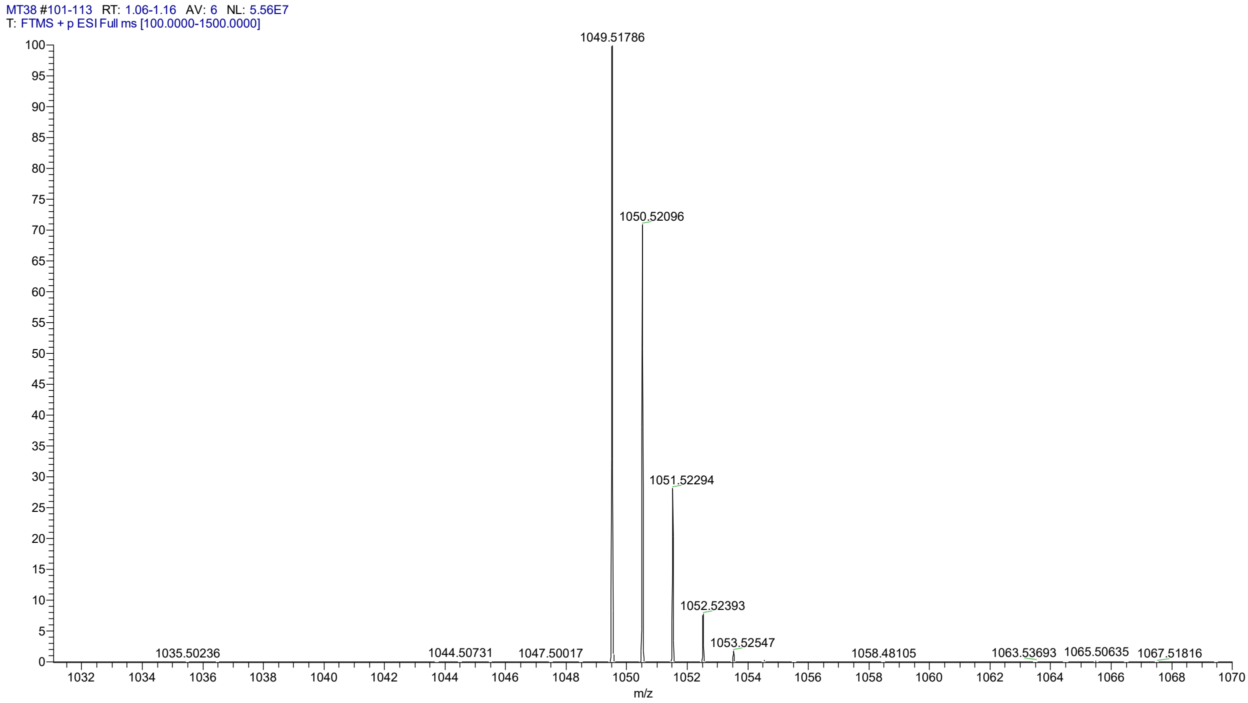

Figure S40. HRMS spectrum of compound **U15**.

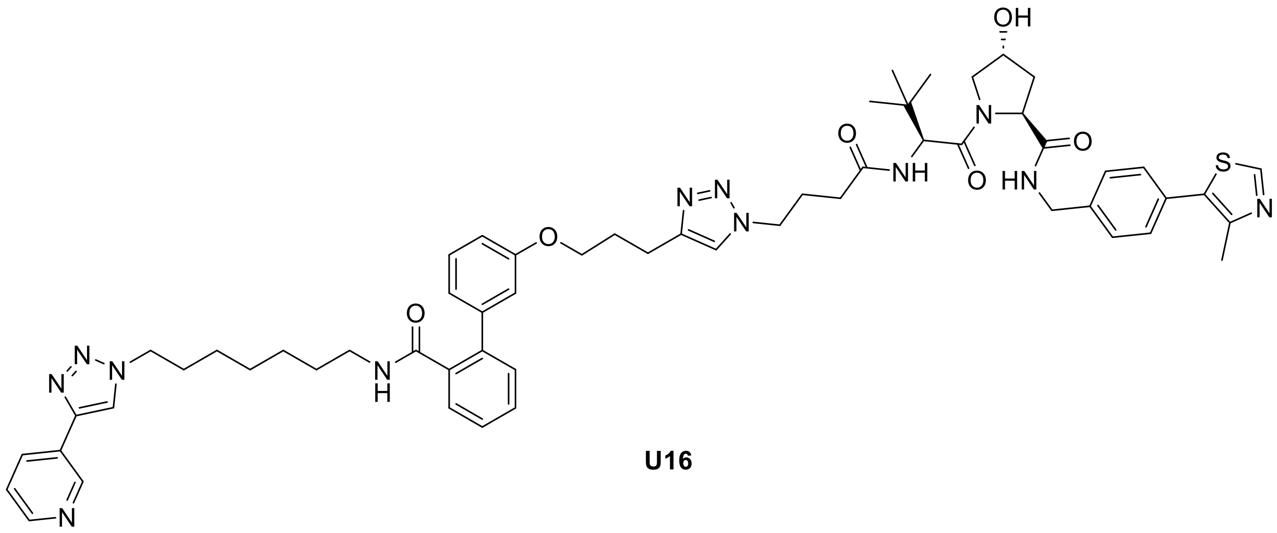

U16

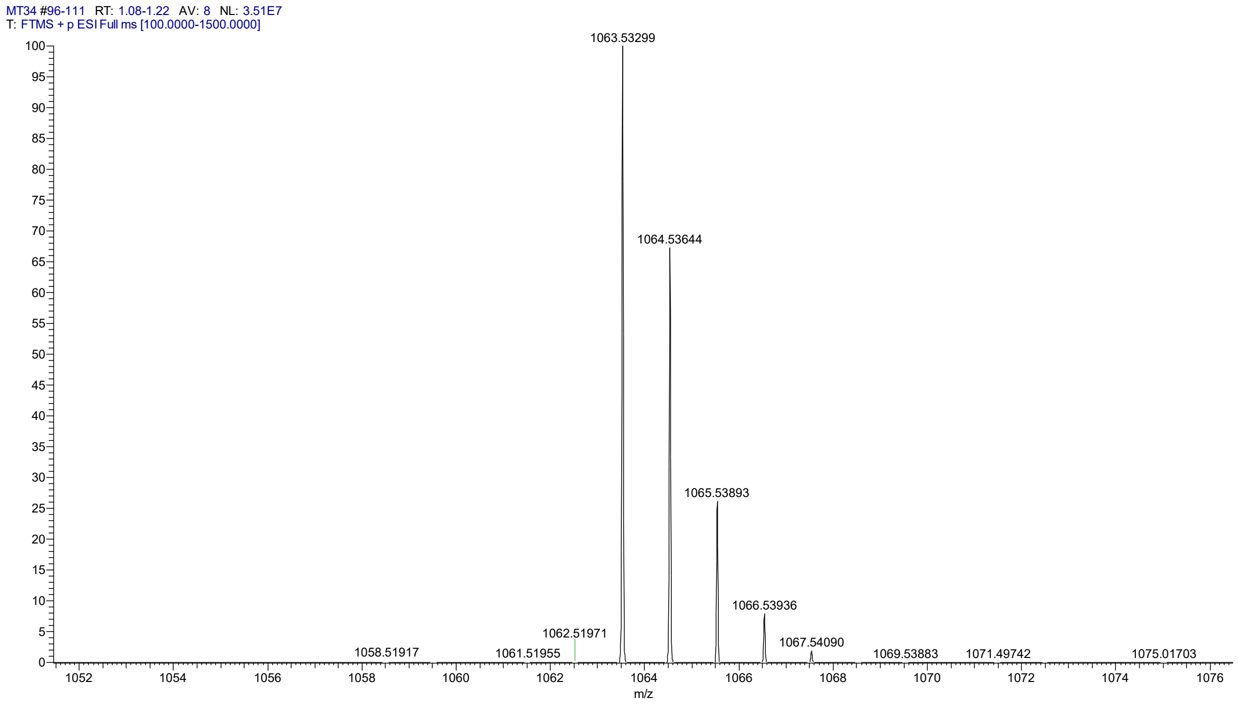

Figure S41. HRMS spectrum of compound **U16**.

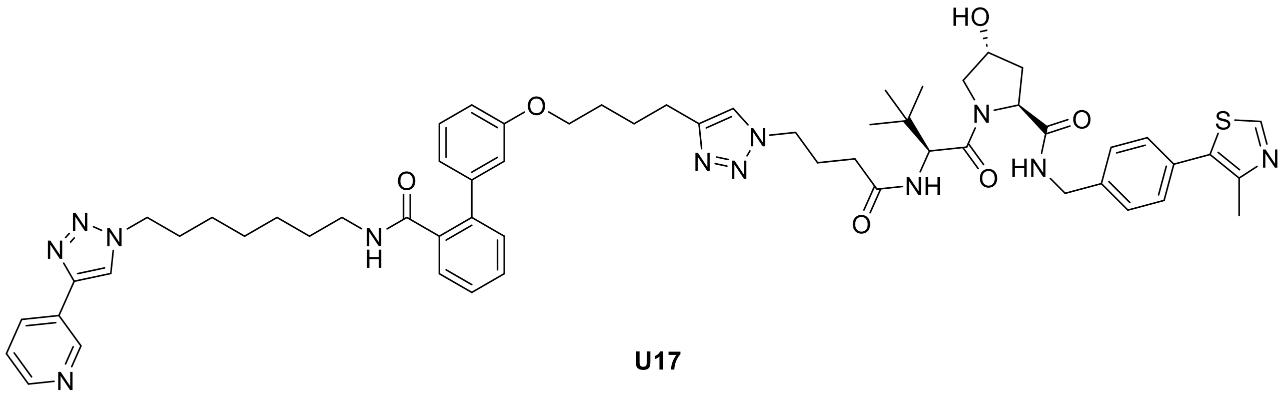

U17

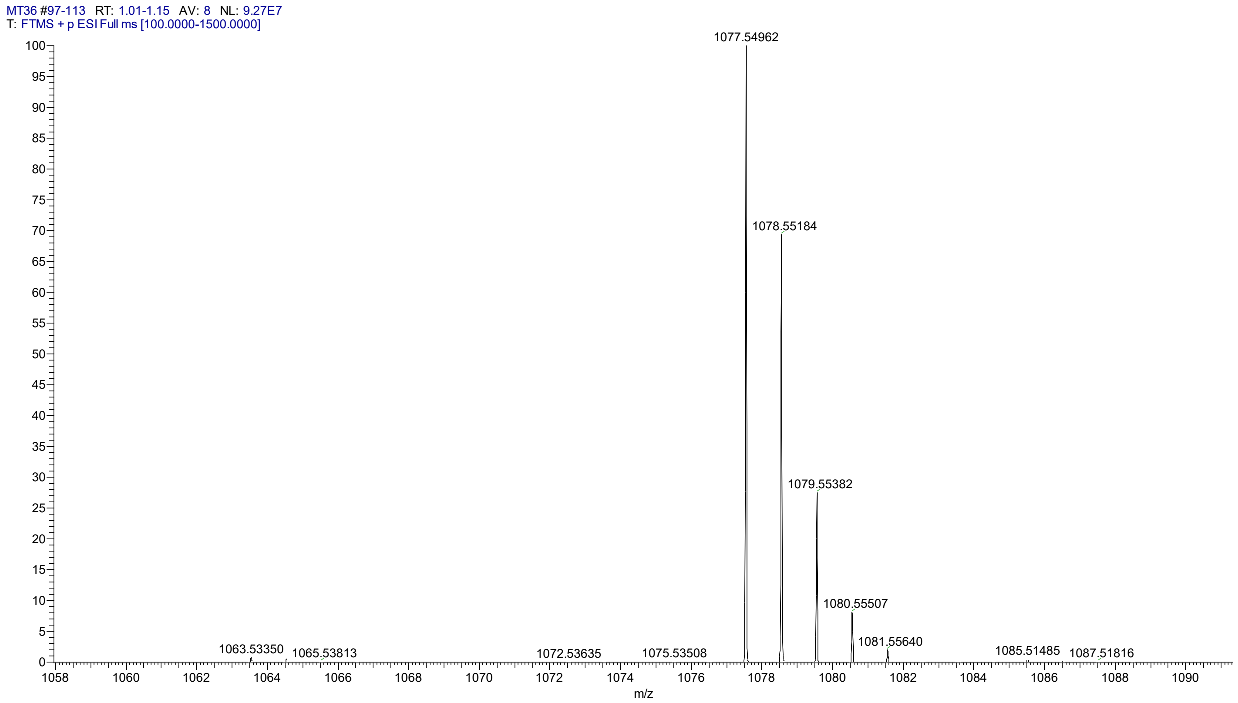

Figure S42. HRMS spectrum of compound **U17**.

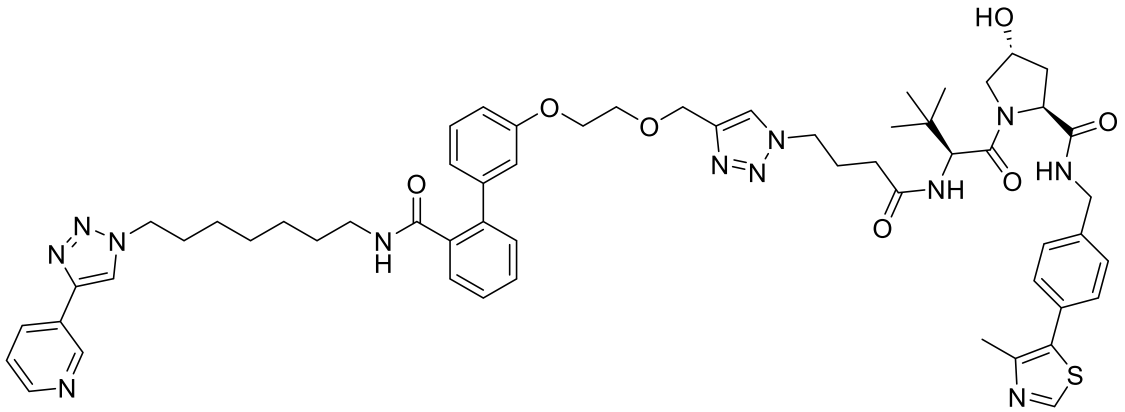

U18

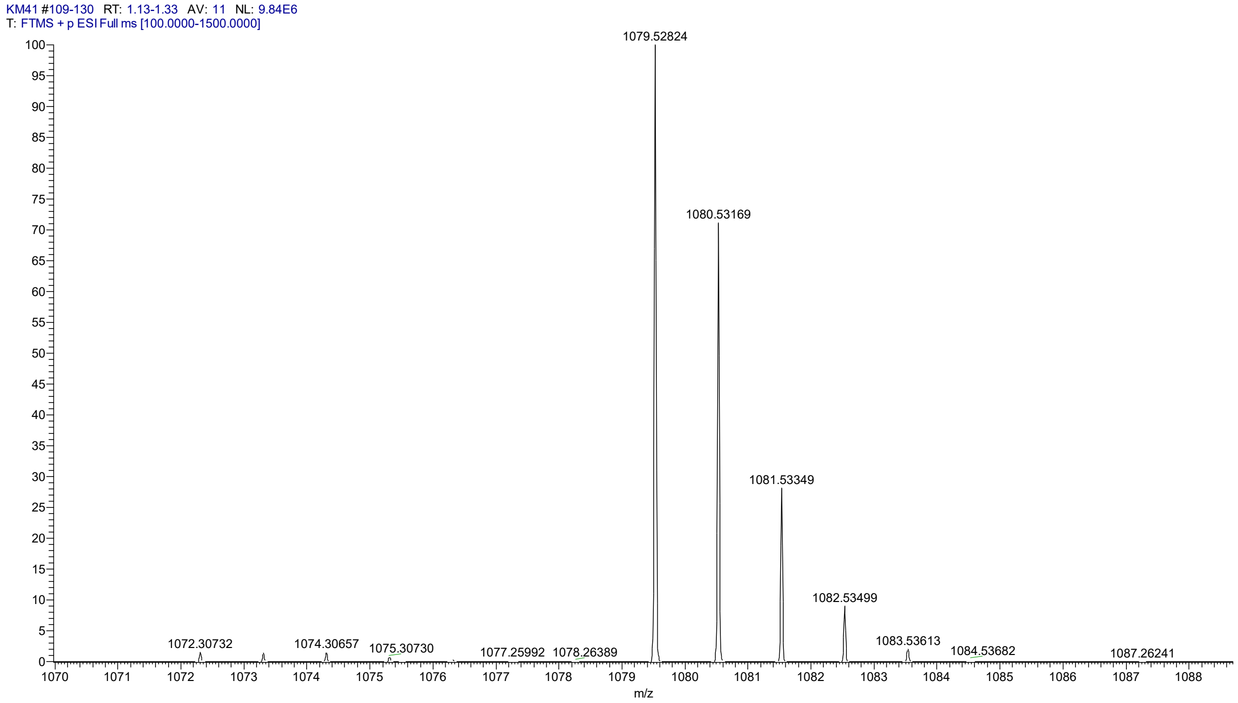

Figure S43. HRMS spectrum of compound **U18**.

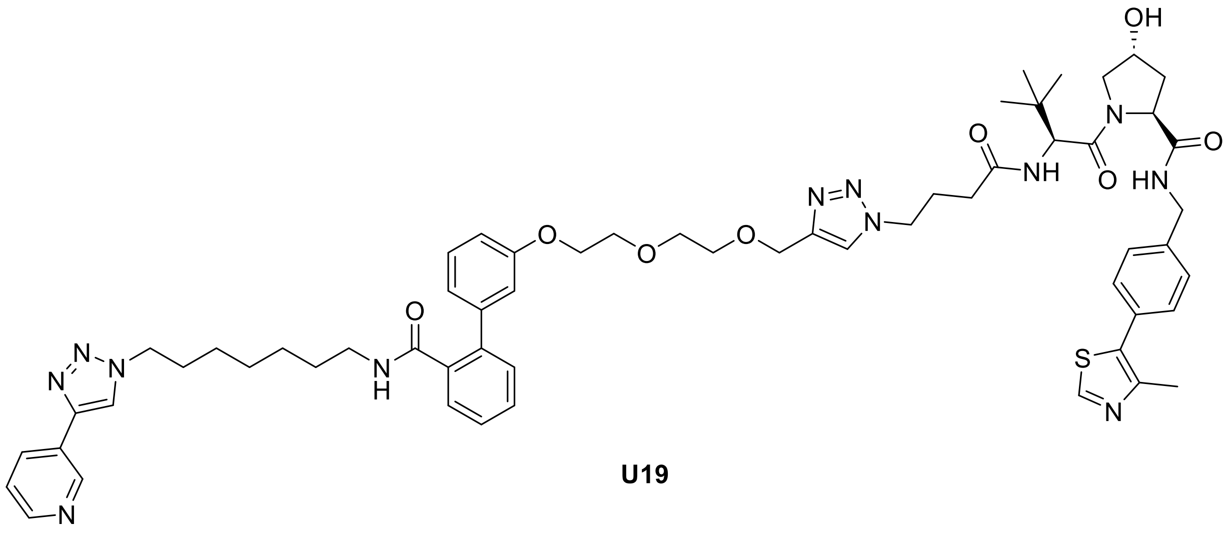

U19

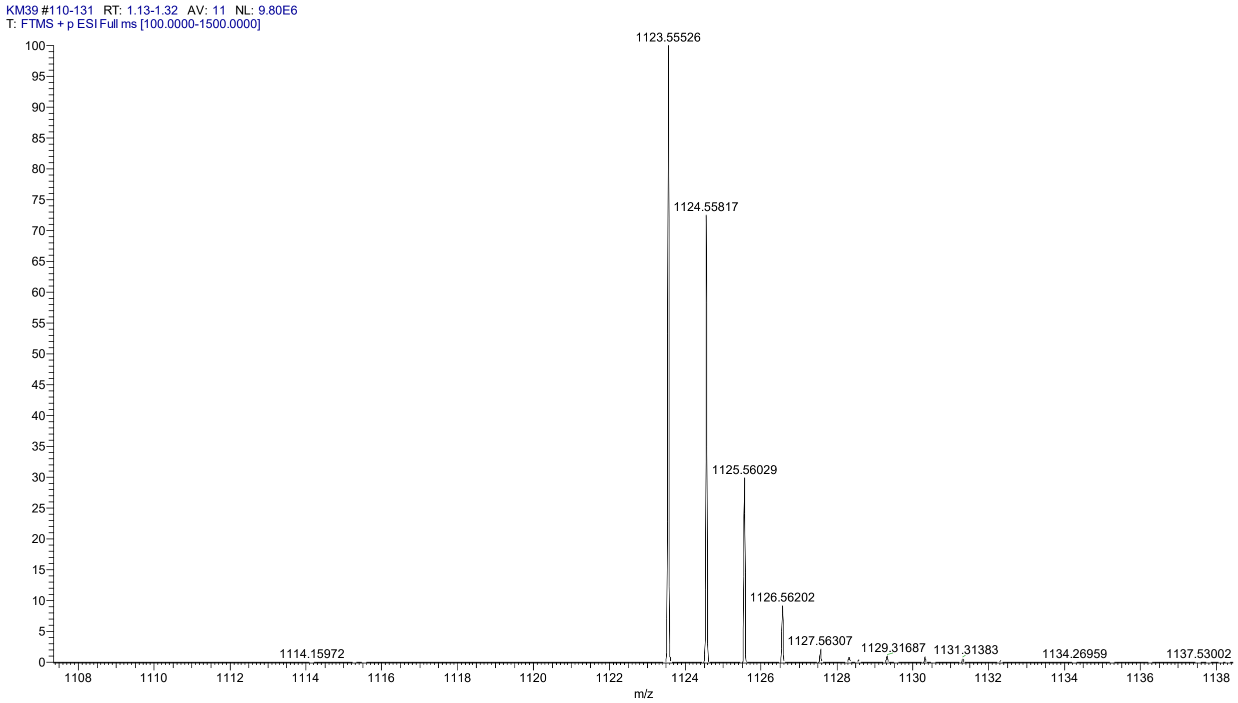

Figure S44. HRMS spectrum of compound **U19**.

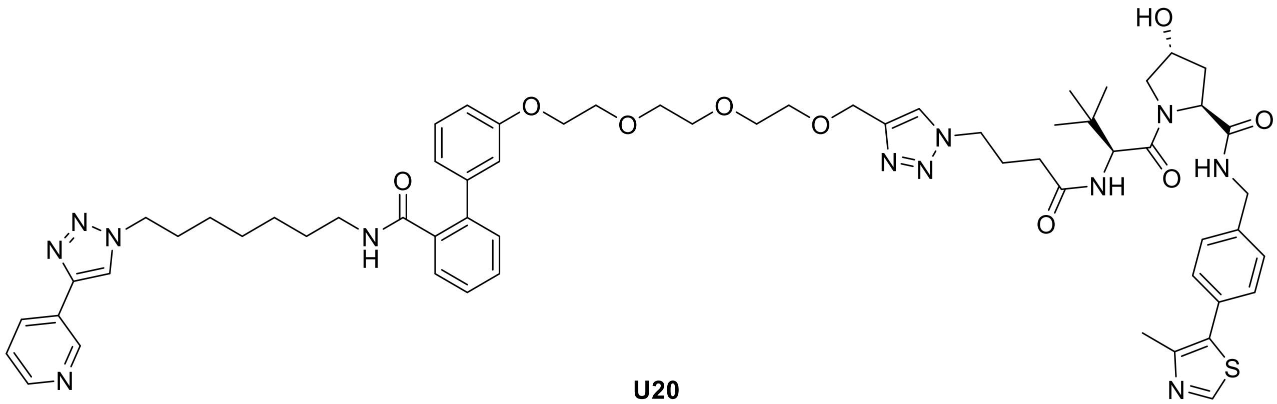

U20

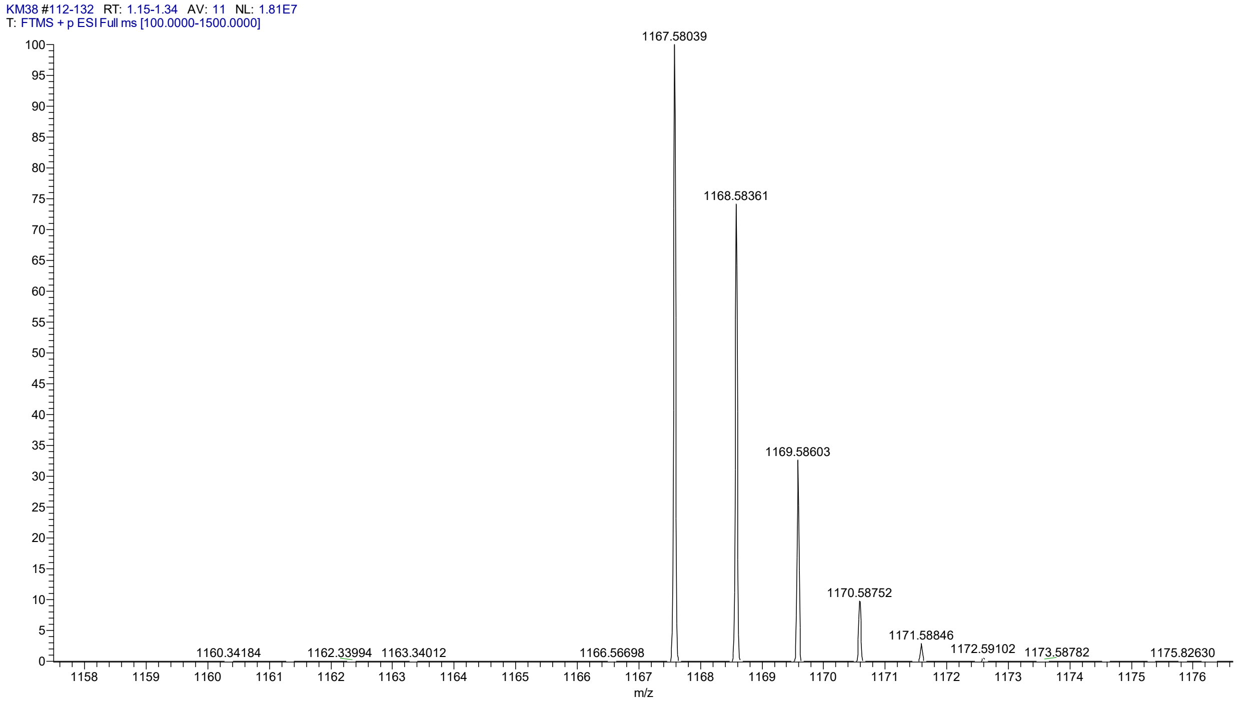

Figure S45. HRMS spectrum of compound **U20**.

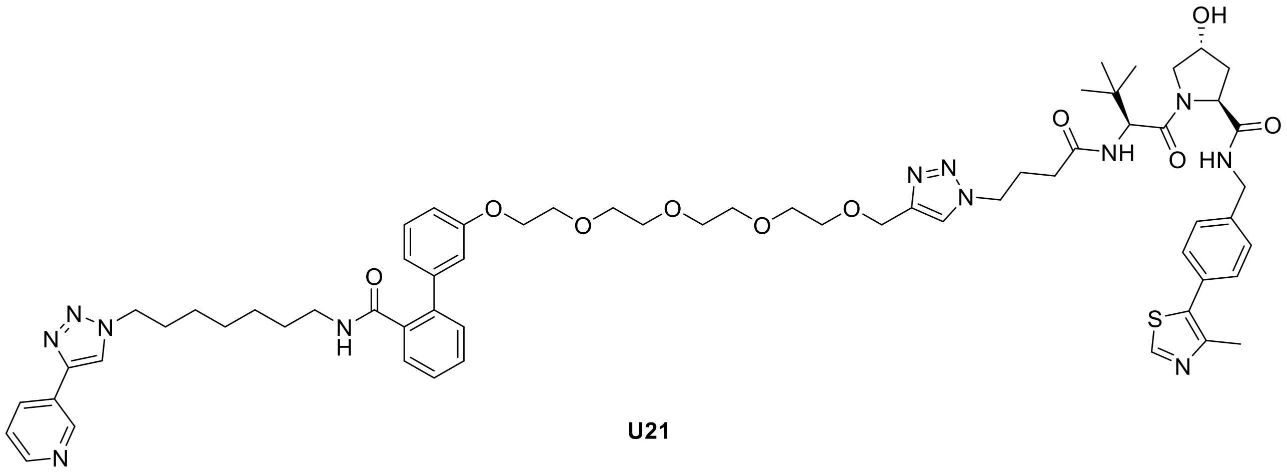

U21

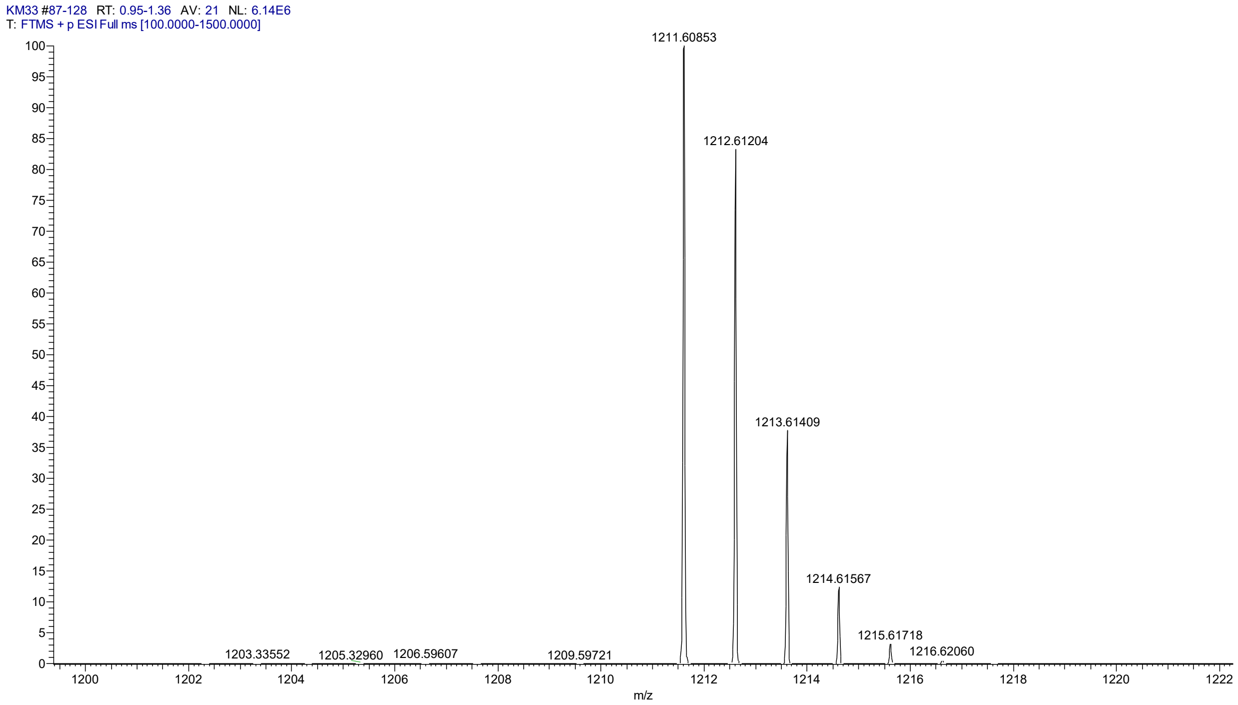

Figure S46. HRMS spectrum of compound **U21**.

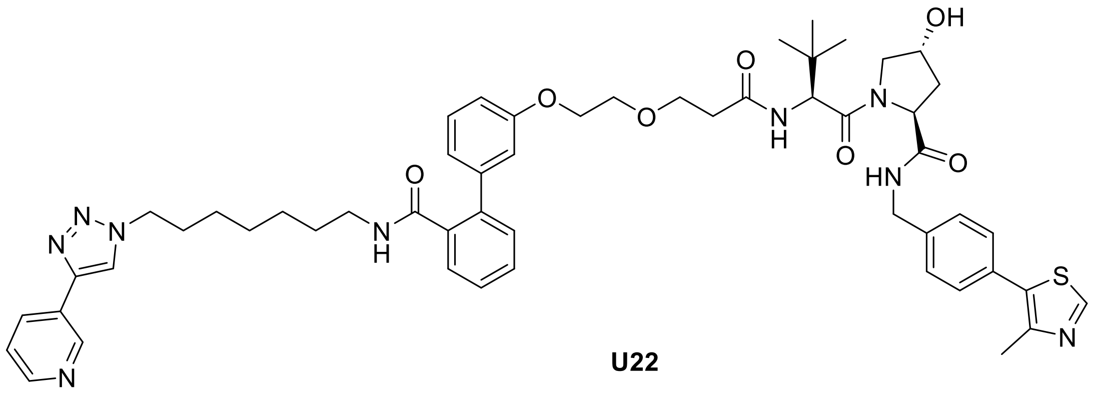

U22

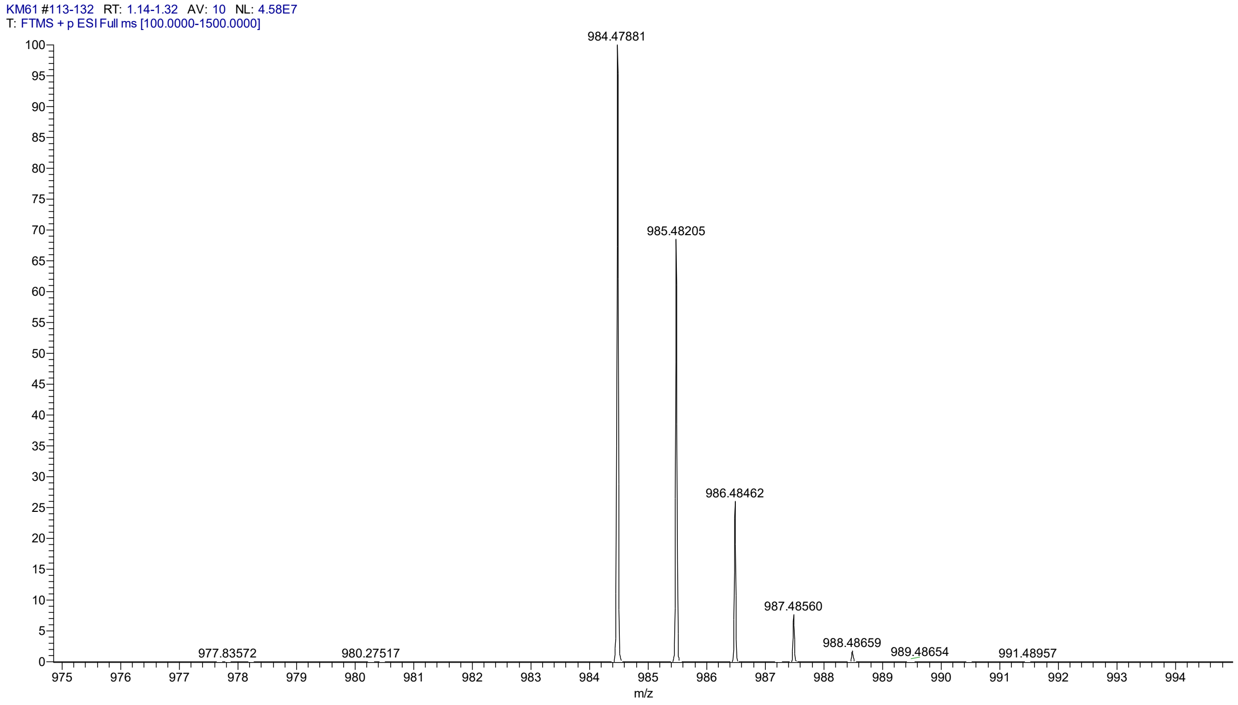

Figure S47. HRMS spectrum of compound **U22**.

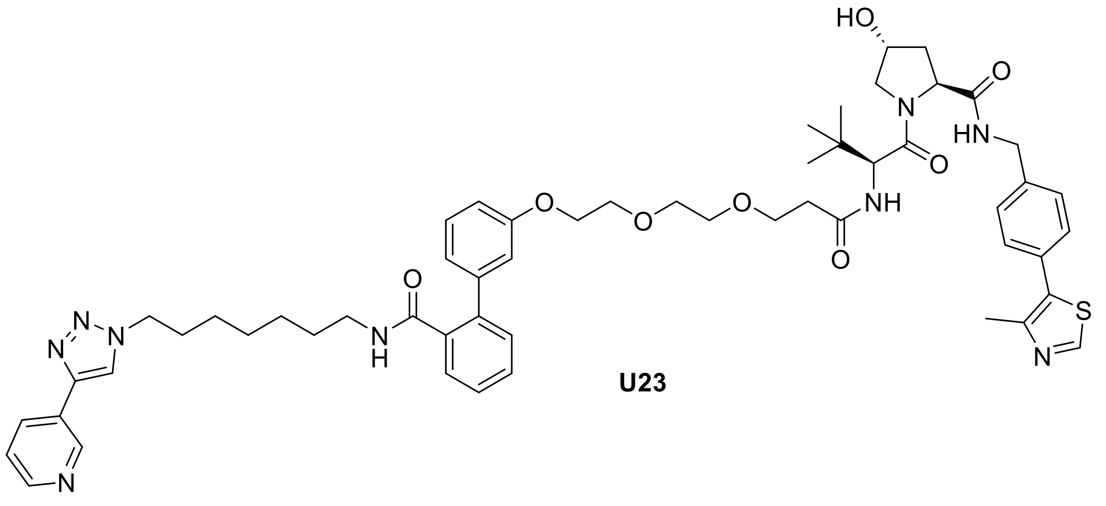

U23

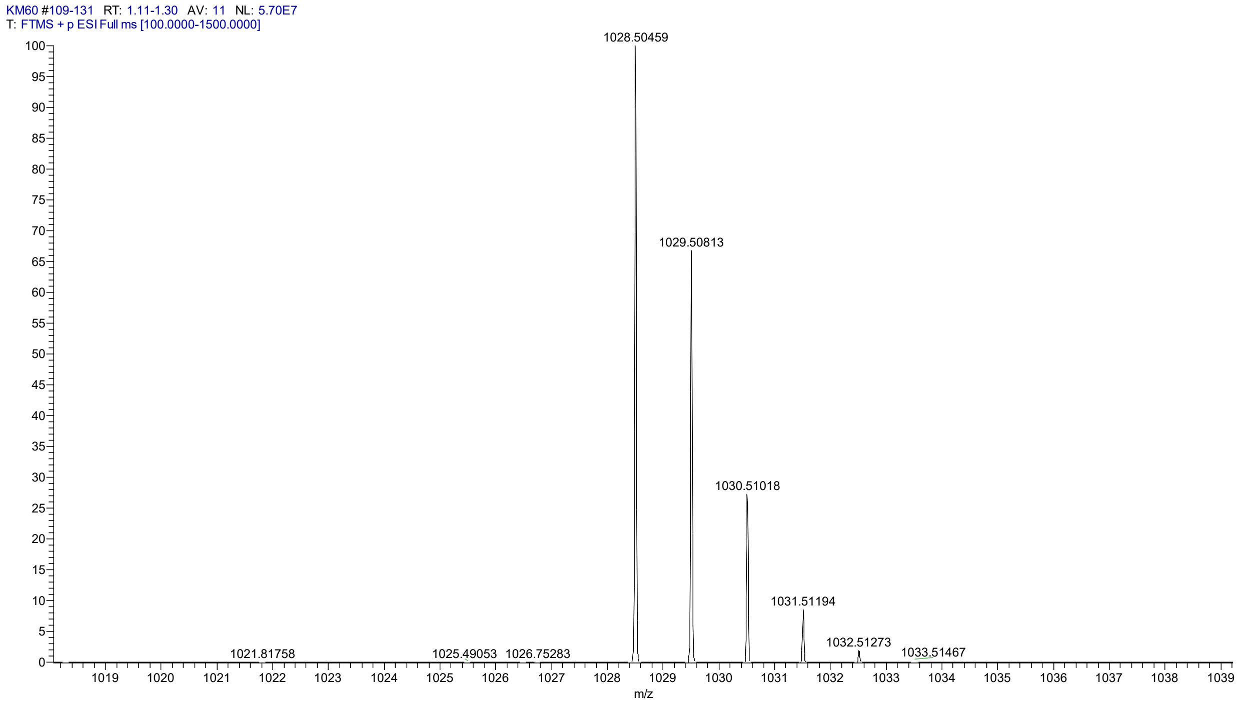

Figure S48. HRMS spectrum of compound **U23**.

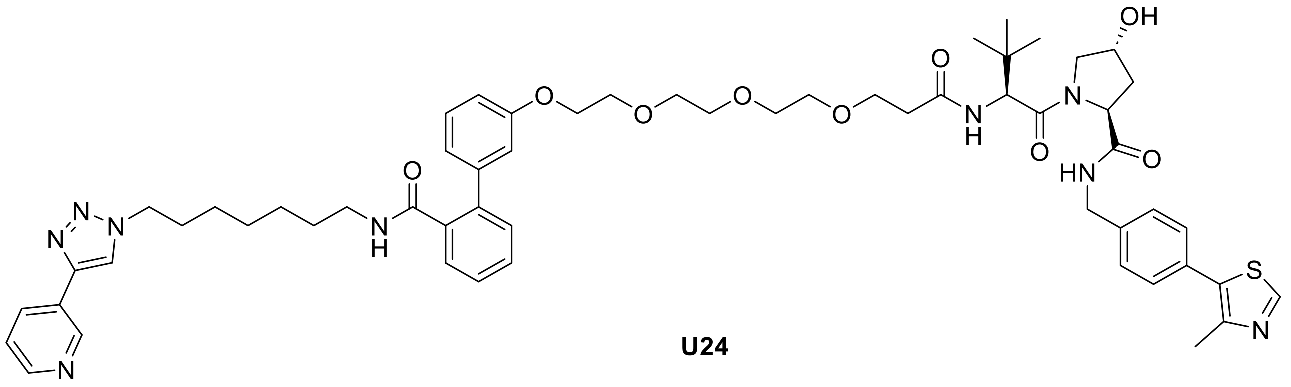

U24

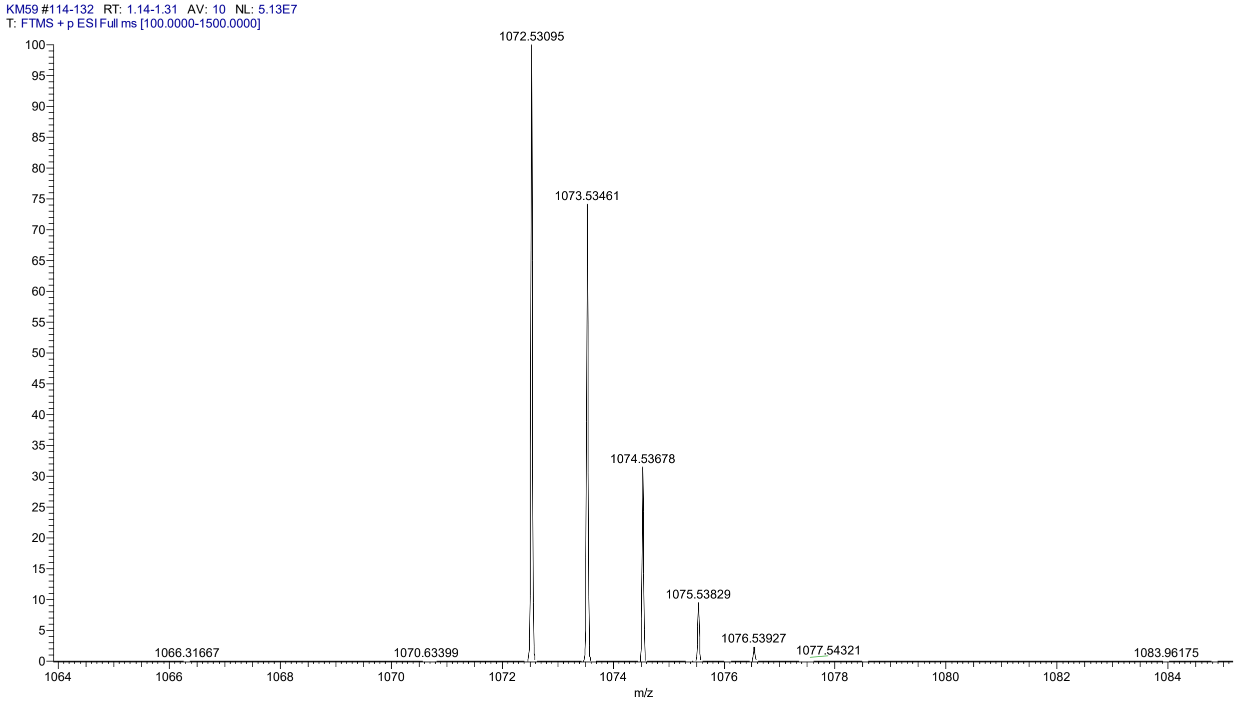

Figure S49. HRMS spectrum of compound **U24**.

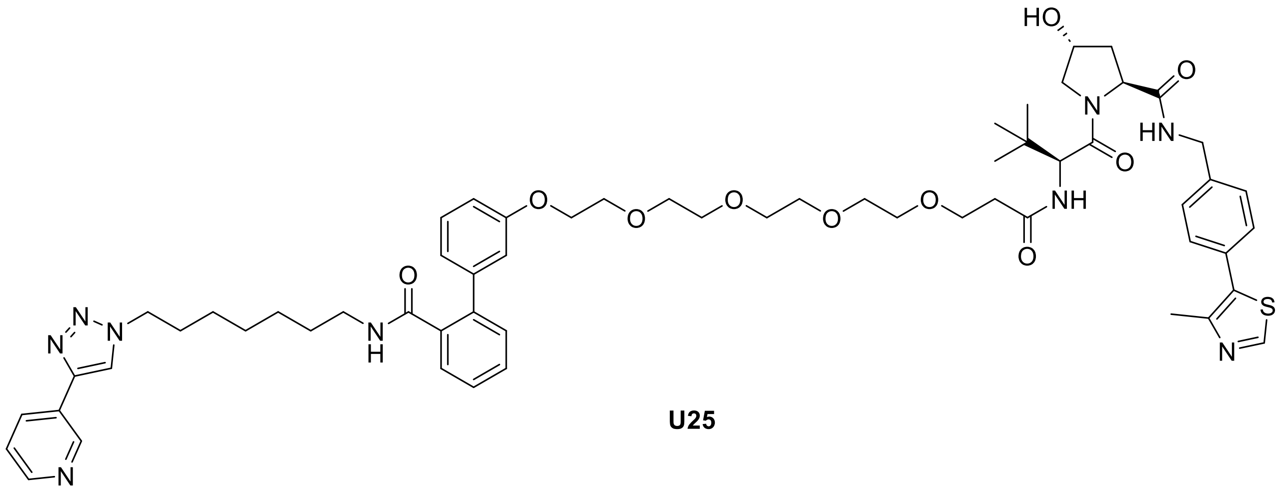

U25

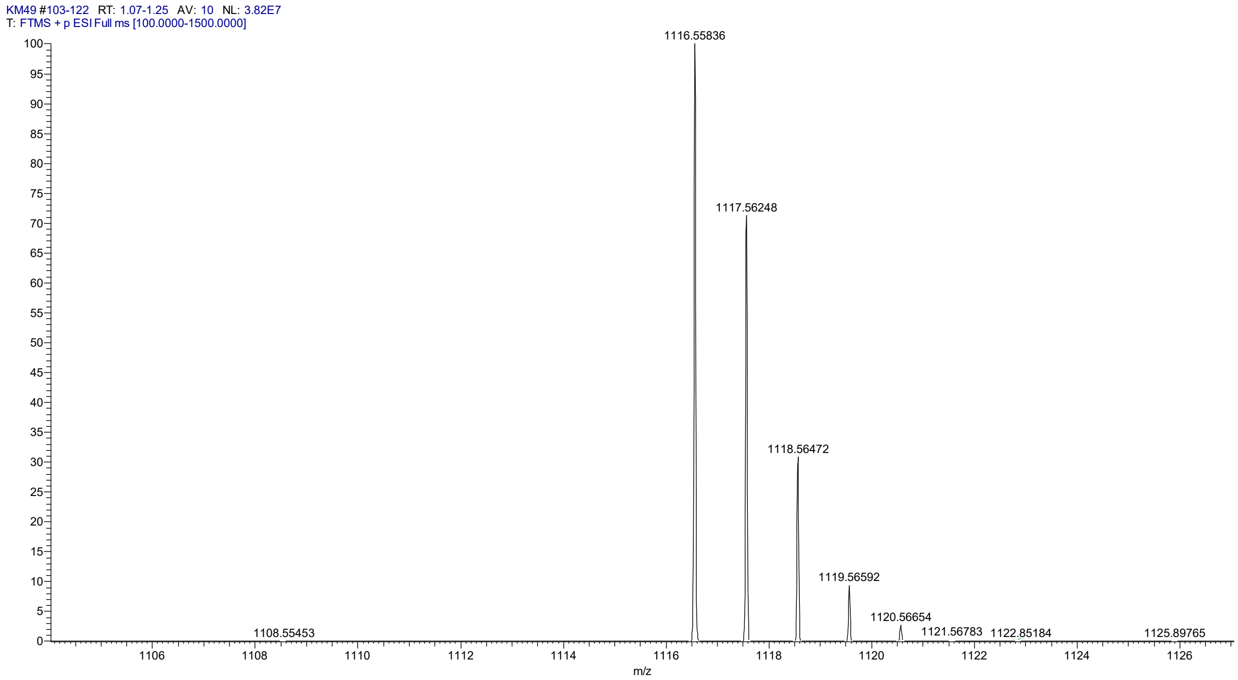

Figure S50. HRMS spectrum of compound **U25**.

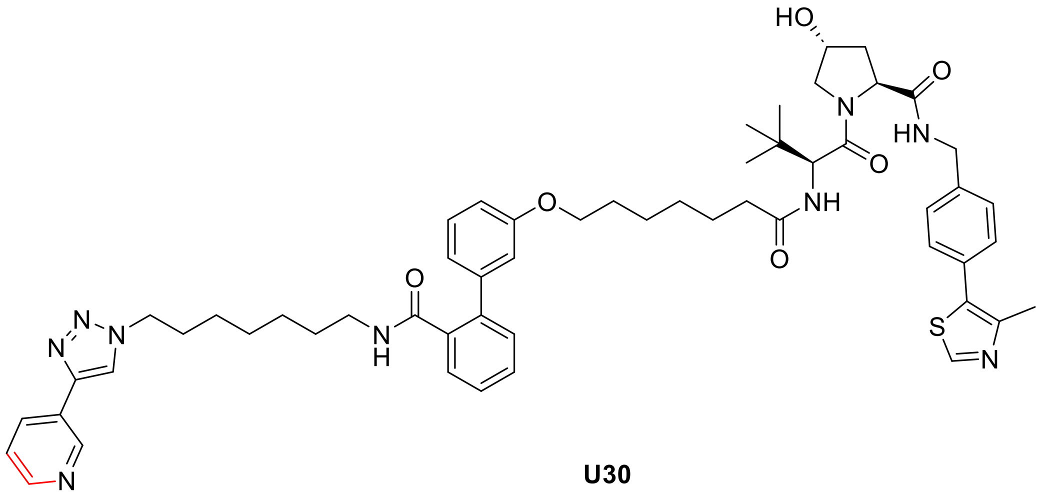

U30

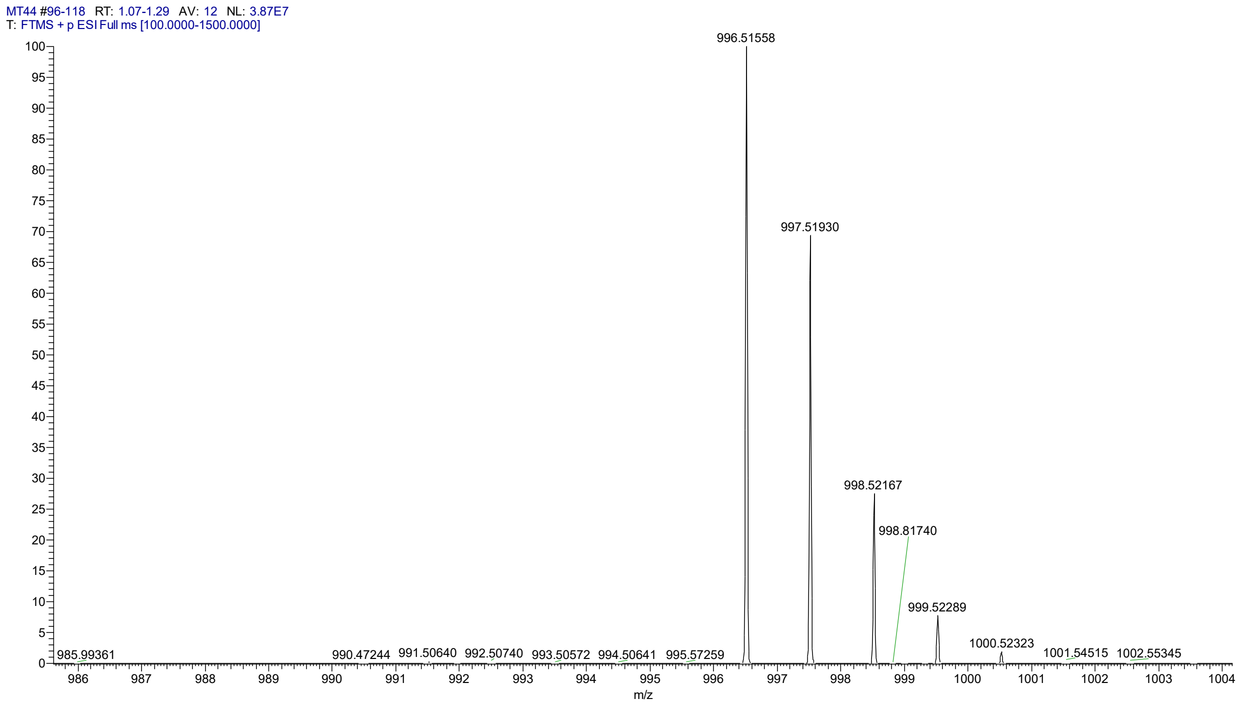

Figure S51. HRMS spectrum of compound **U30**.

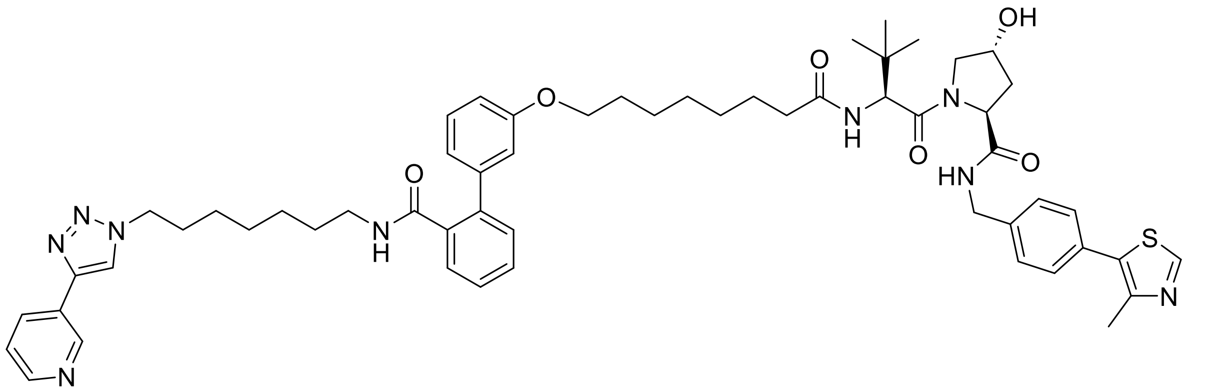

U31

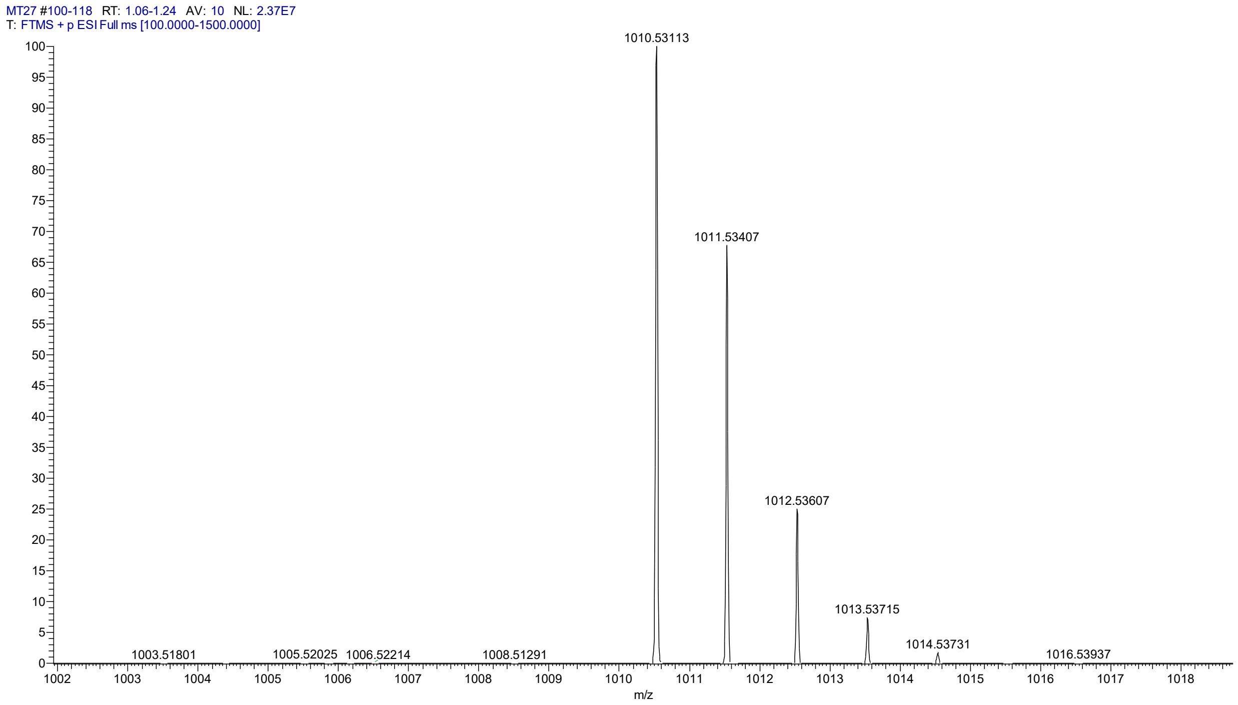

Figure S52. HRMS spectrum of compound **U31**.

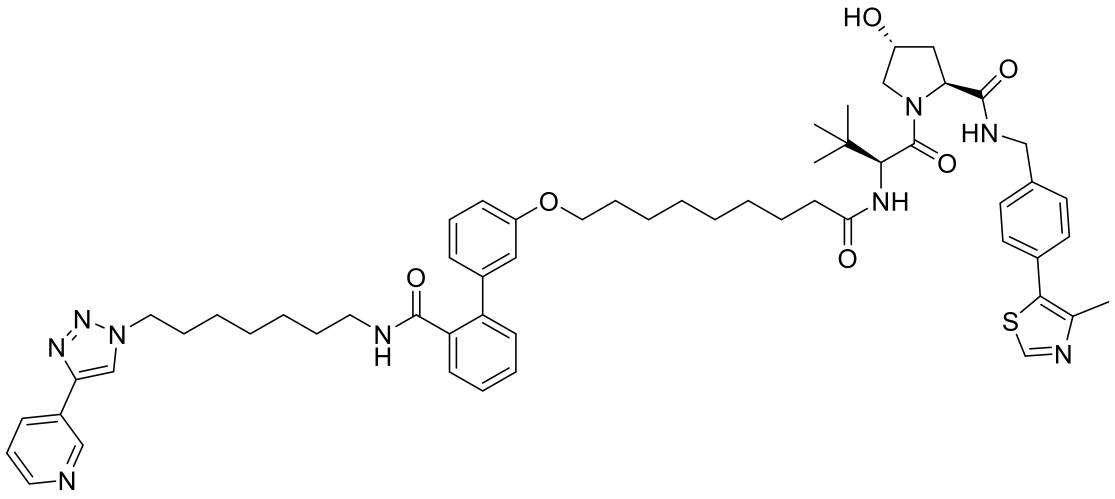

U32

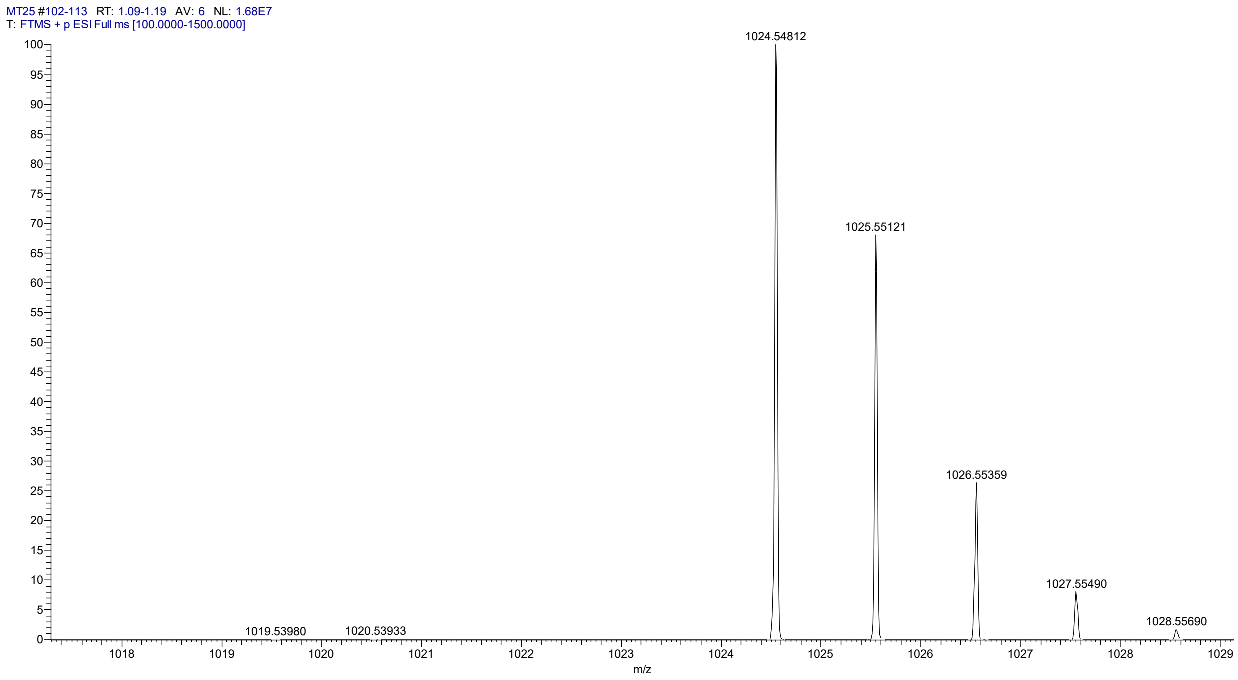

Figure S53. HRMS spectrum of compound **U32**.

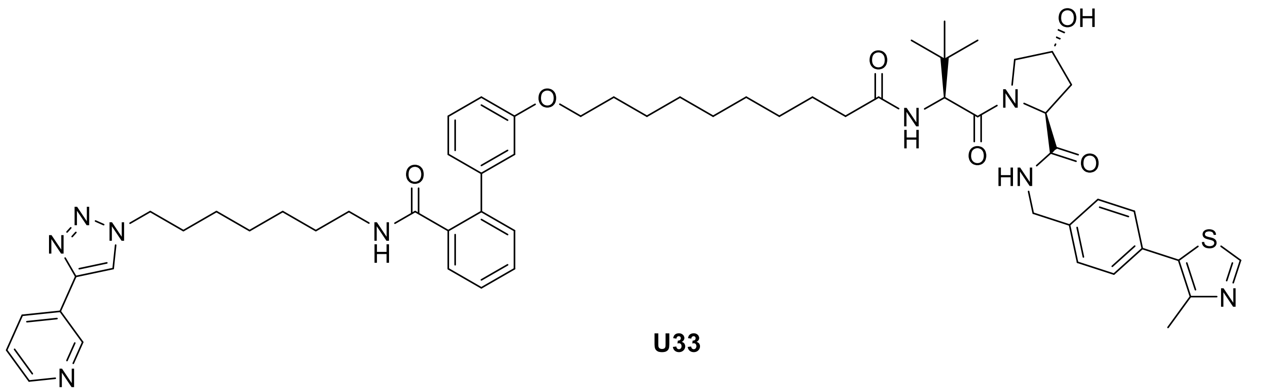

U33

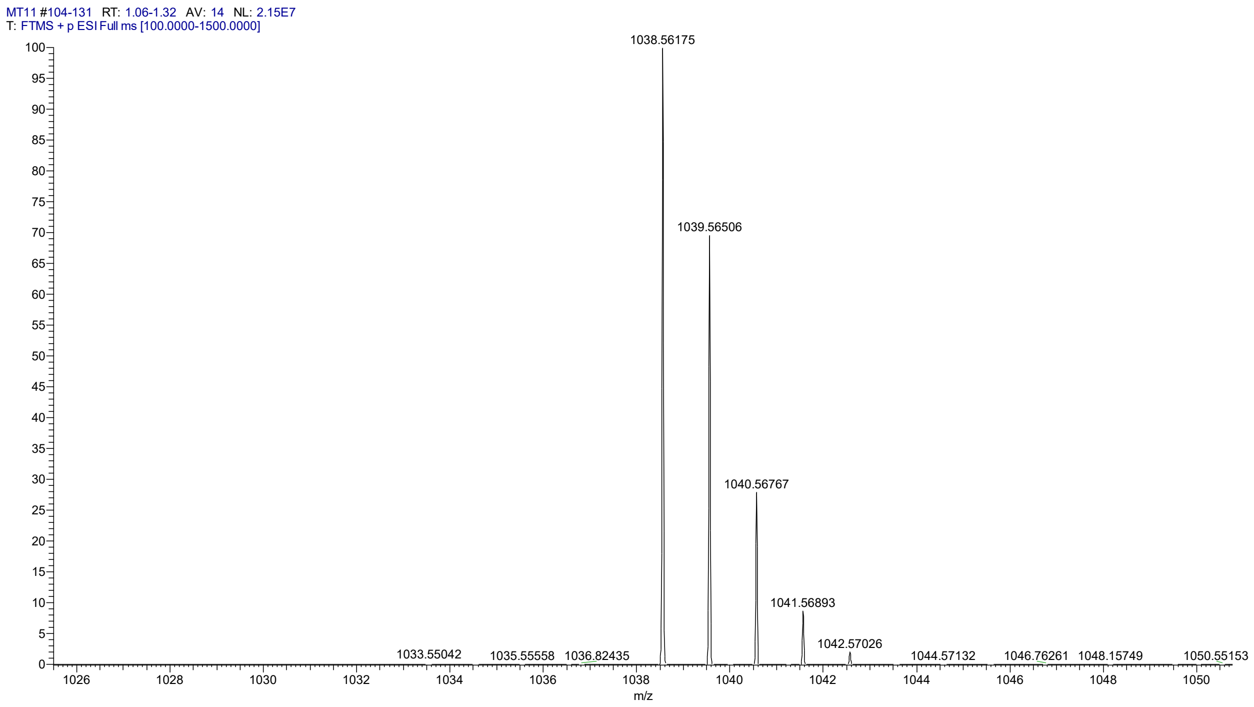

Figure S54. HRMS spectrum of compound **U33**.

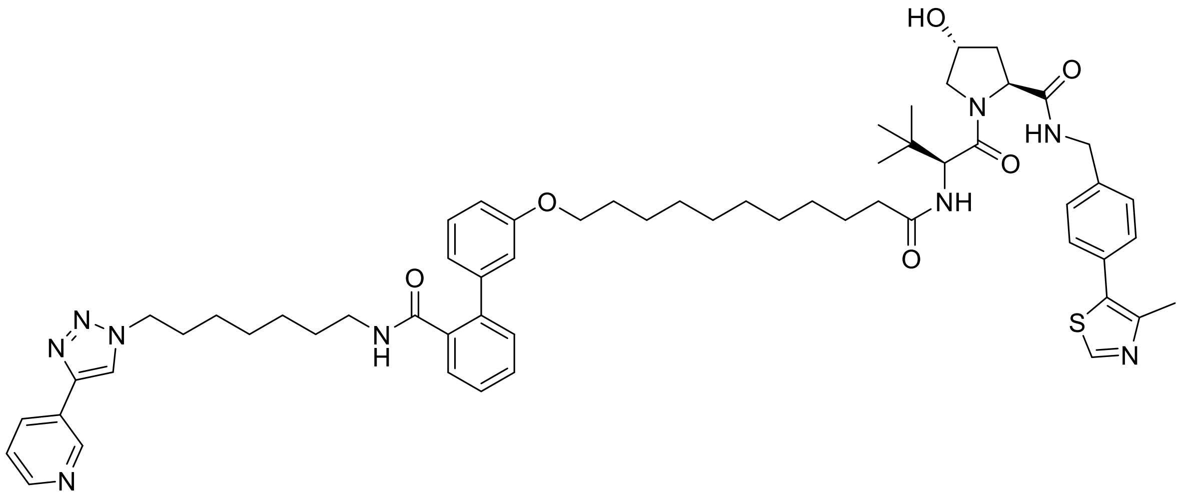

U34

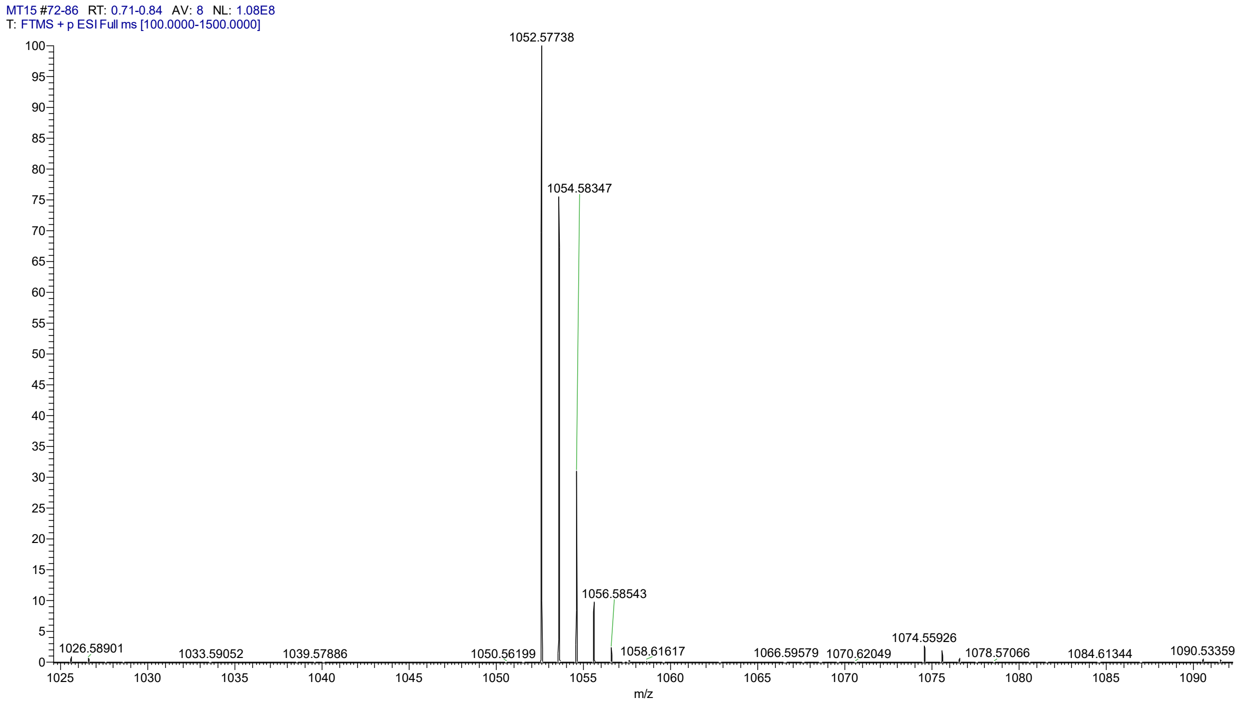

Figure S55. HRMS spectrum of compound **U34**.

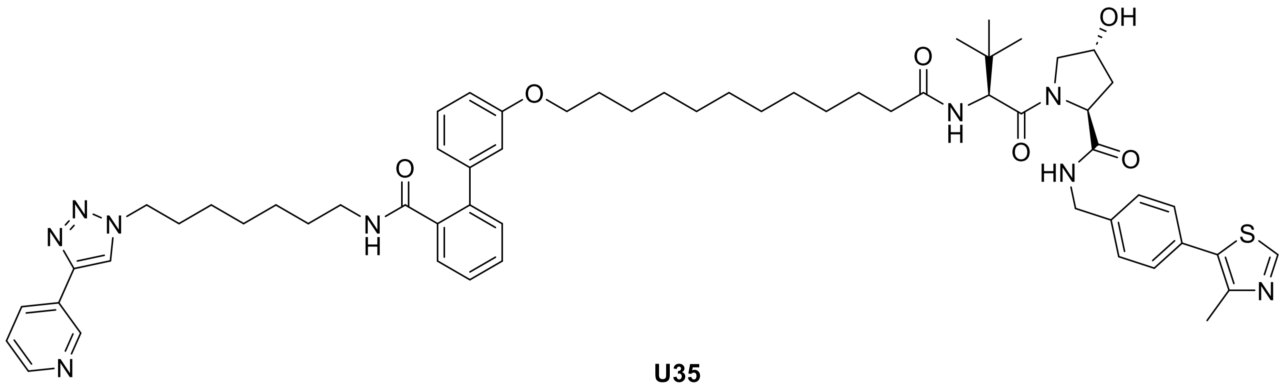

U35

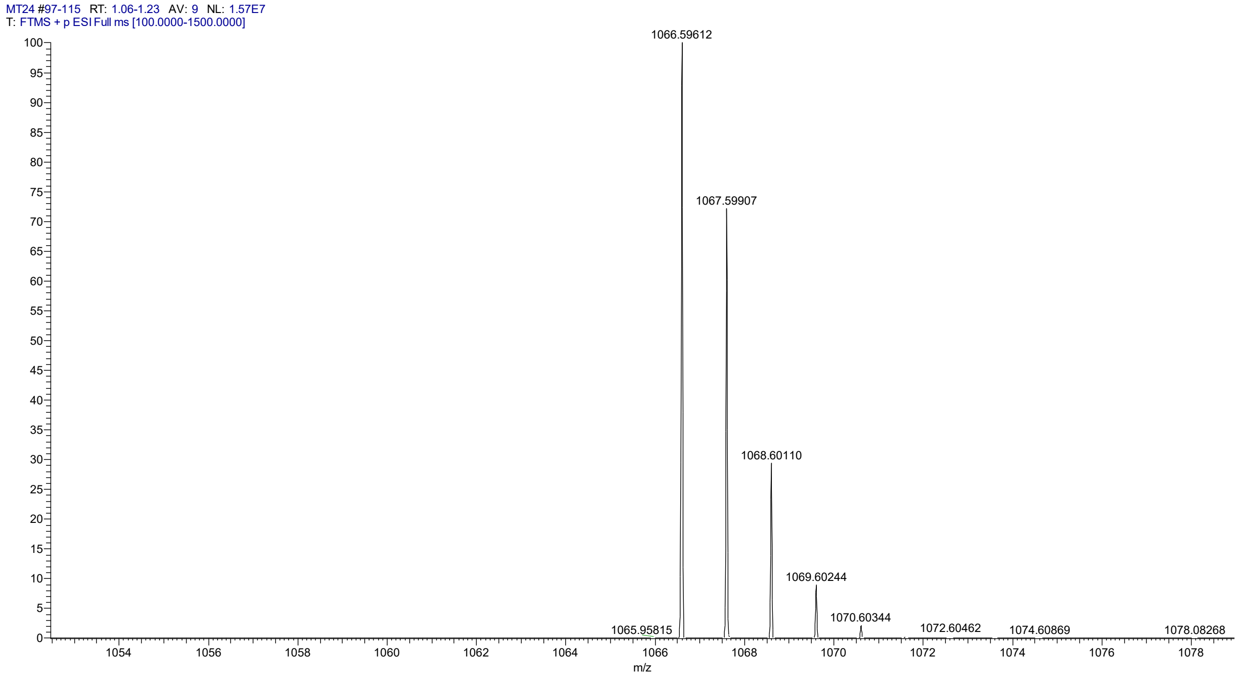

Figure S56. HRMS spectrum of compound **U35**.

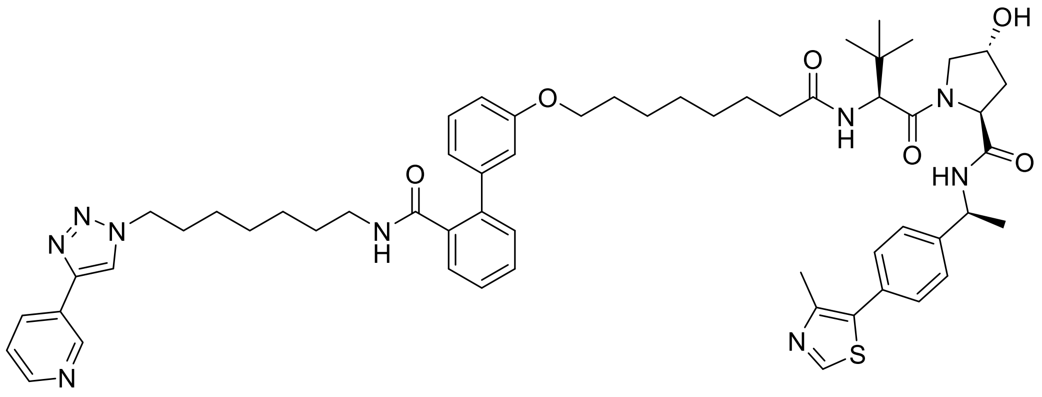

U42

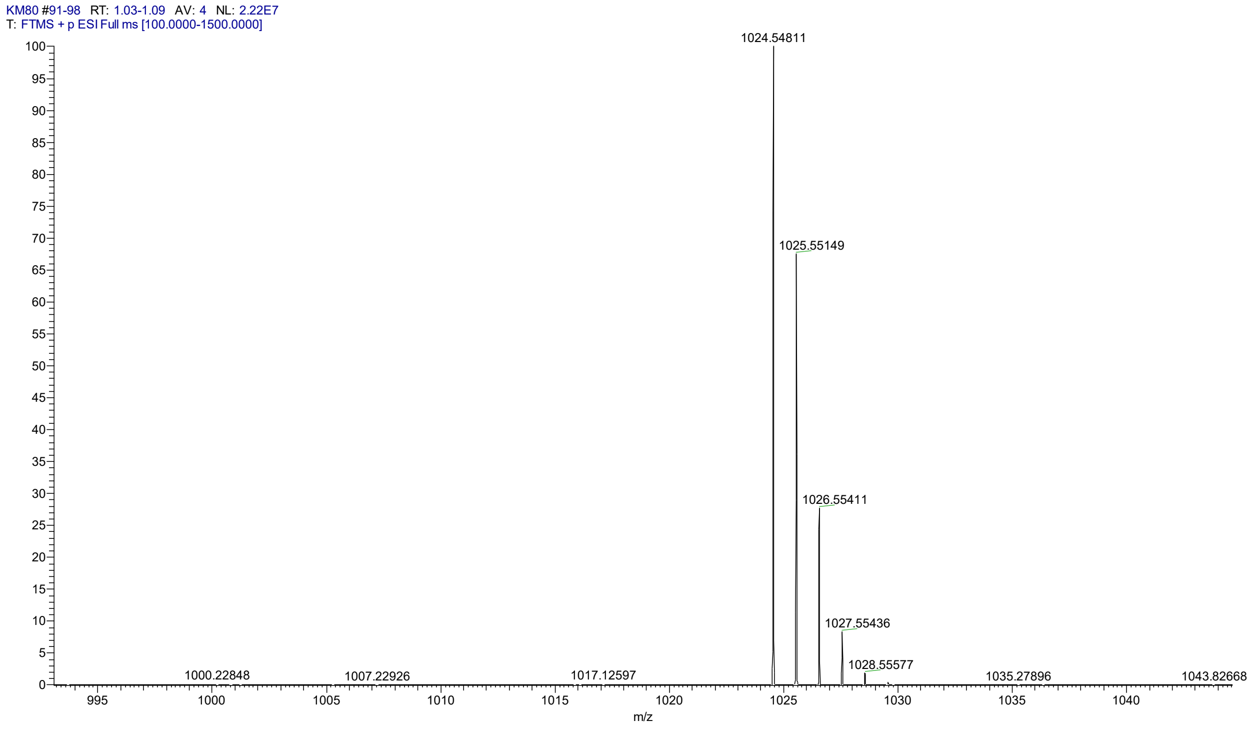

Figure S57. HRMS spectrum of compound **U42**.

Purity evaluation of selected PROTACs by HPLC-UV analysis.

Instrumentation:	Shimadzu HPLC system LC-10AD series
Column:	Syncronis aQ 5 µm d_p, 150 × 4.6 mm (Thermo Scientific).
Mobile Phase	Phase A: 0.2% formic acid in water HPLC grade
	Phase B: 0.2% formic acid in methanol HPLC grade
Analysis mode:	Gradient of concentration.

Time (min)	% B
0.00	30
9.00	90
14.50	90
15.00	30
20.00	30

Detection:	λ= 254, 220 nm
Flow rate:	1 mL/min
Injection volume:	5 µL of 1 mg/mL compounds solution dissolved in methanol

- **U14**

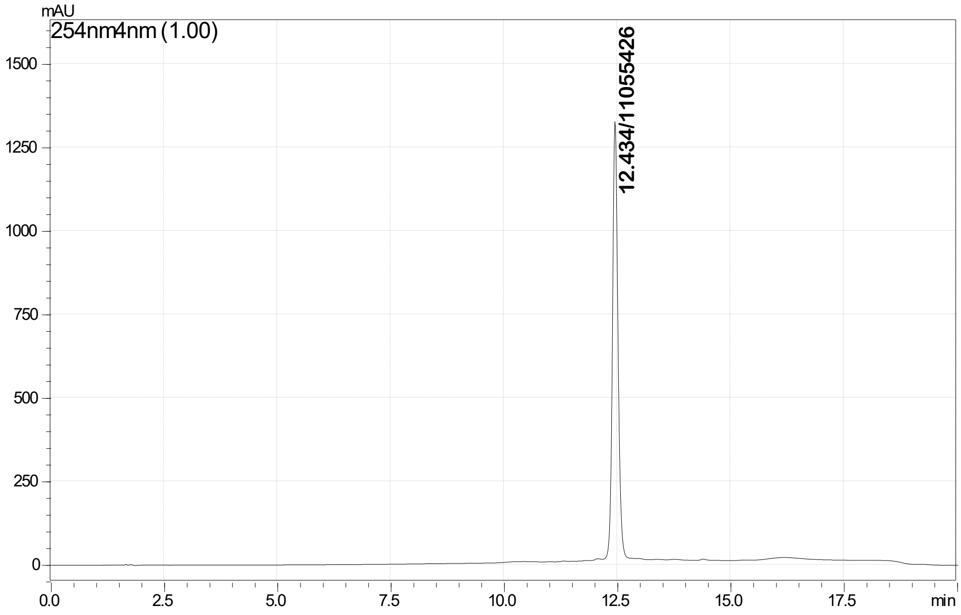

Purity λ: 254 nm >99%

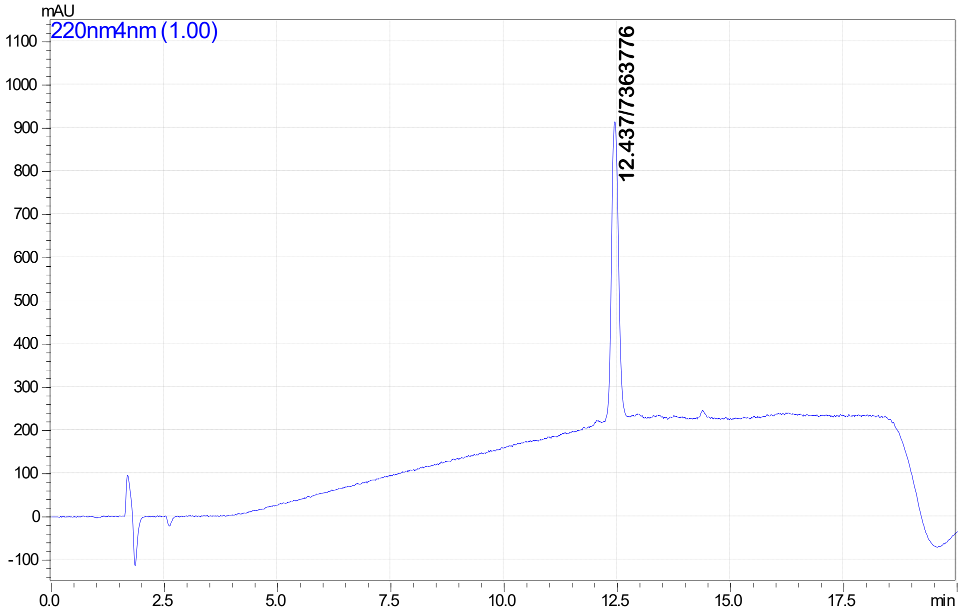

Purity λ: 220 nm >99%

- **U15**

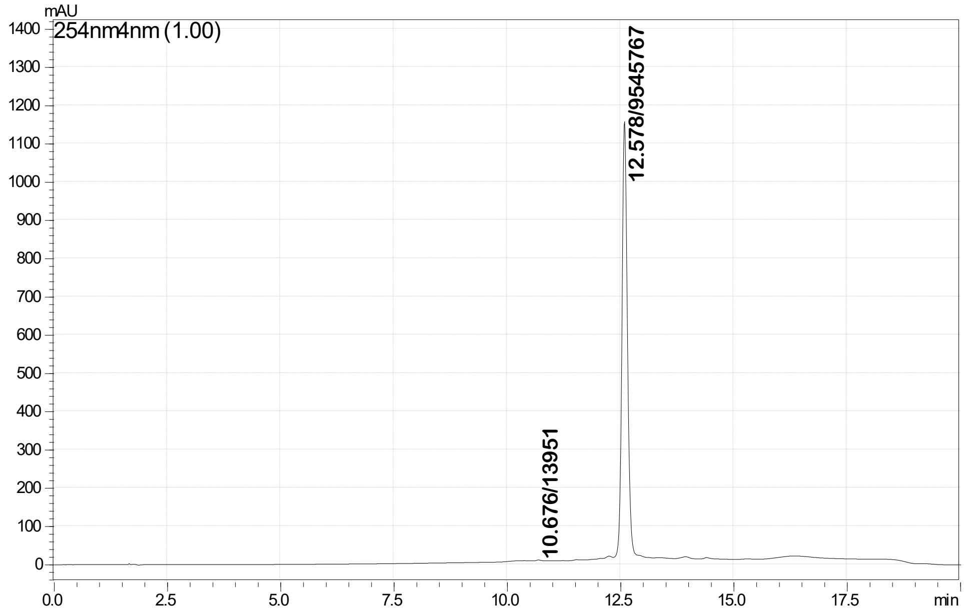

Purity λ: 254 nm >99%

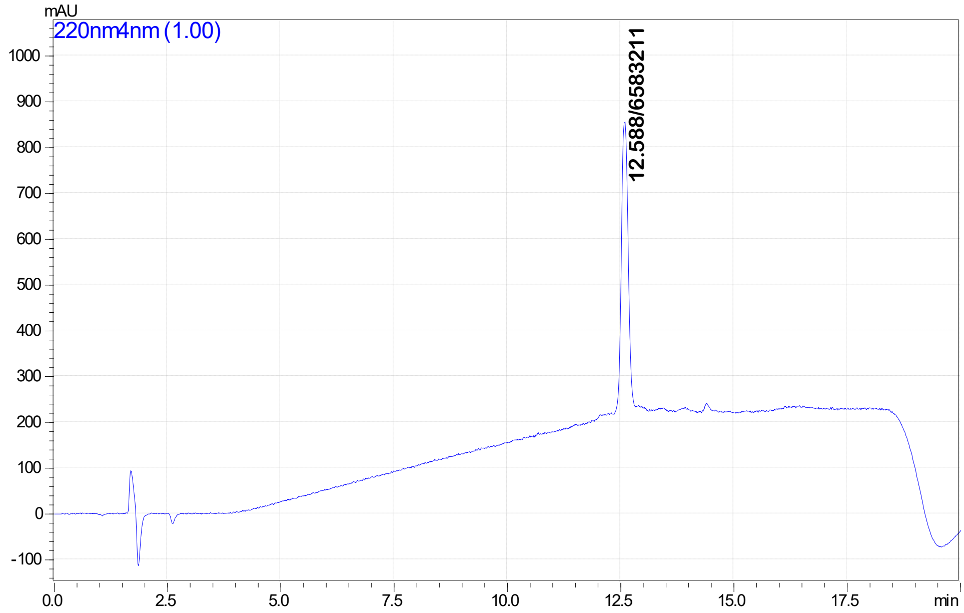

Purity λ: 220 nm >99%

- **U16**

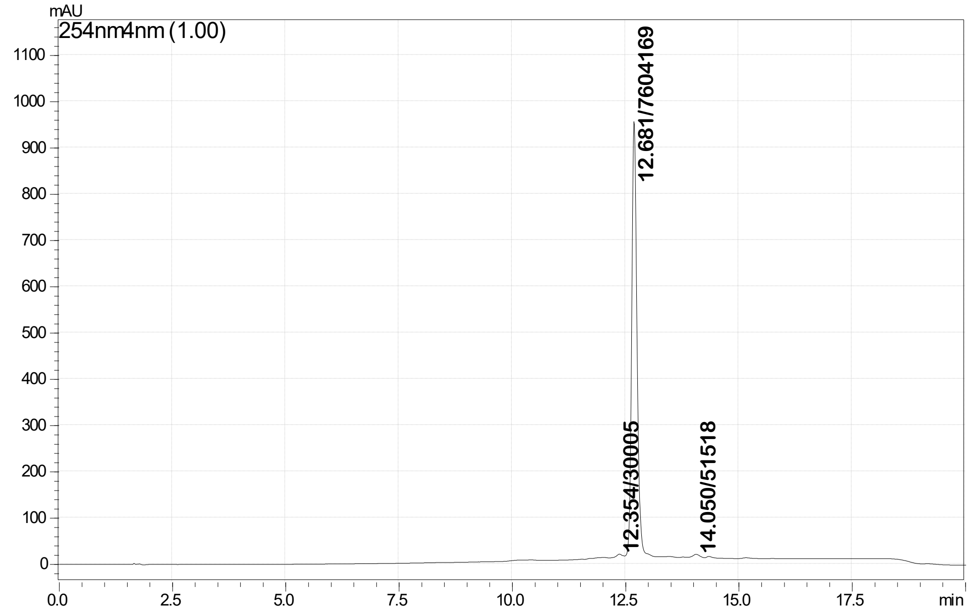

Purity λ: 254 nm =98.9%

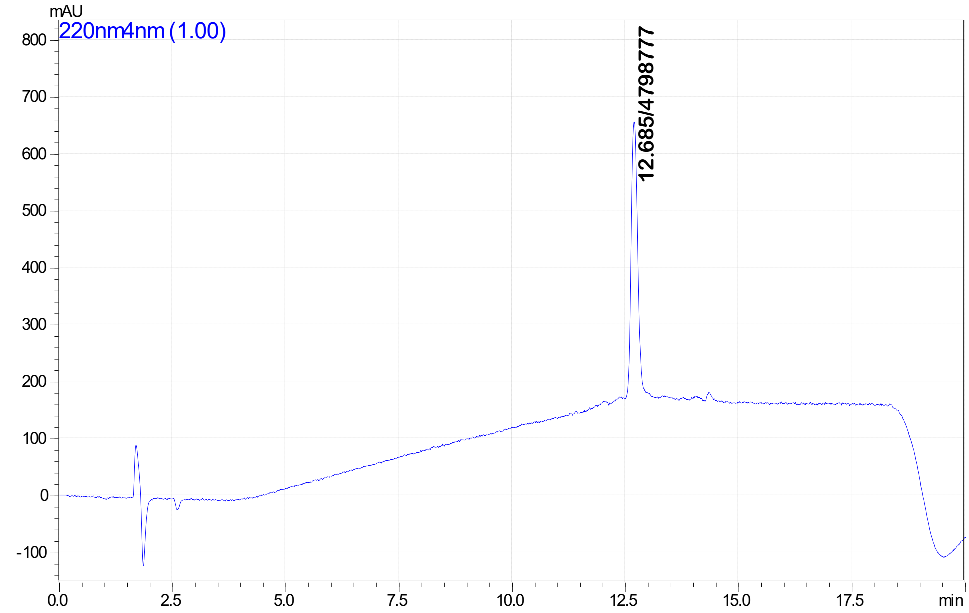

Purity λ: 220 nm >99%

- **U17**

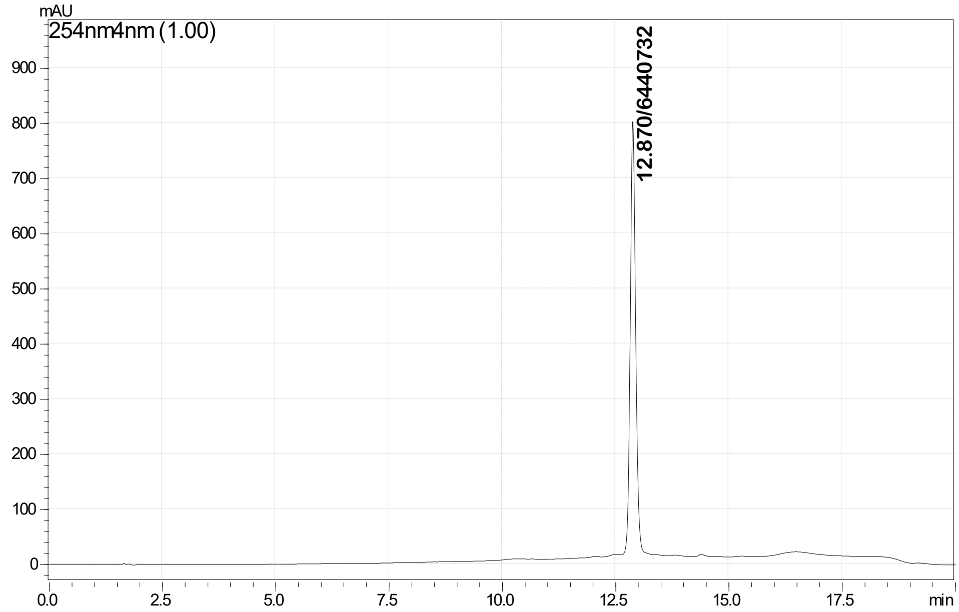

Purity λ: 254 nm =98.9%

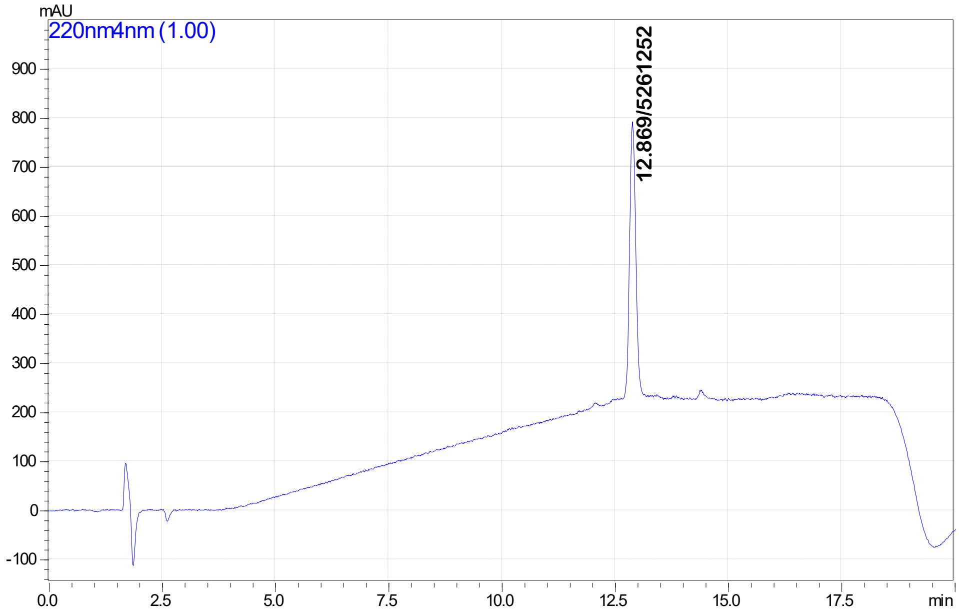

Purity λ: 220 nm >99%

- **U18**

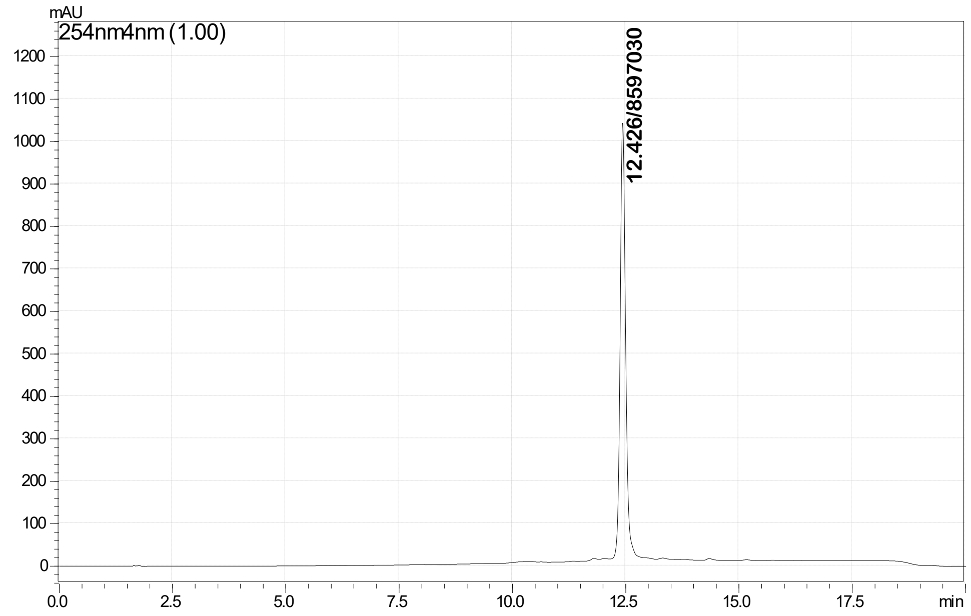

Purity λ: 254 nm >99%

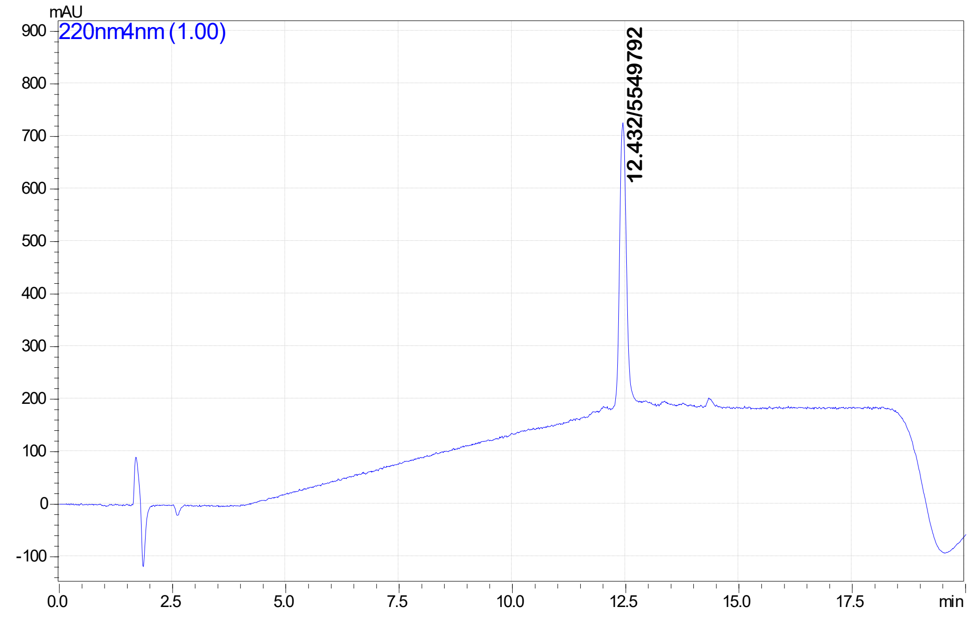

Purity λ: 220 nm >99%

- **U19**

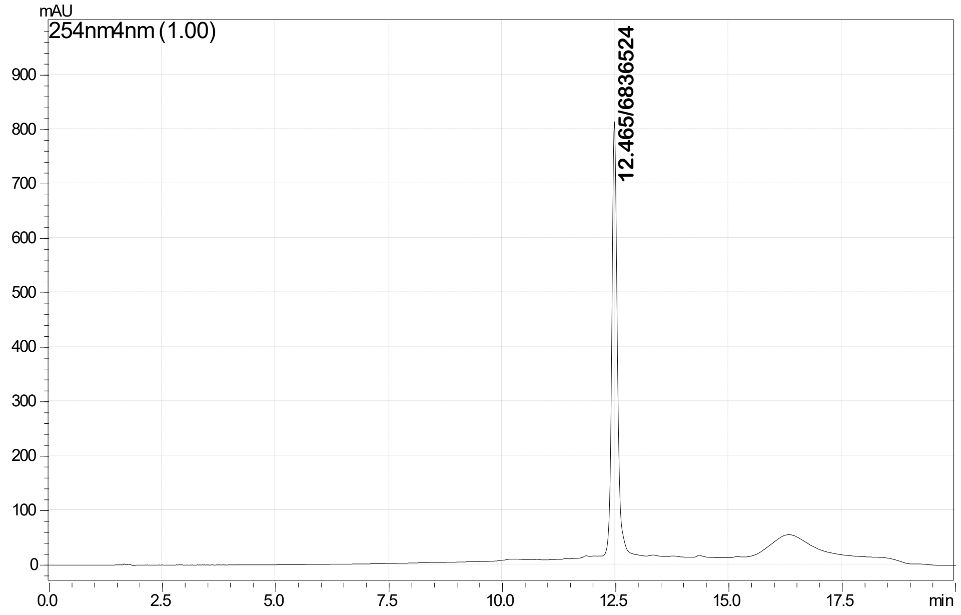

Purity λ: 254 nm >99%

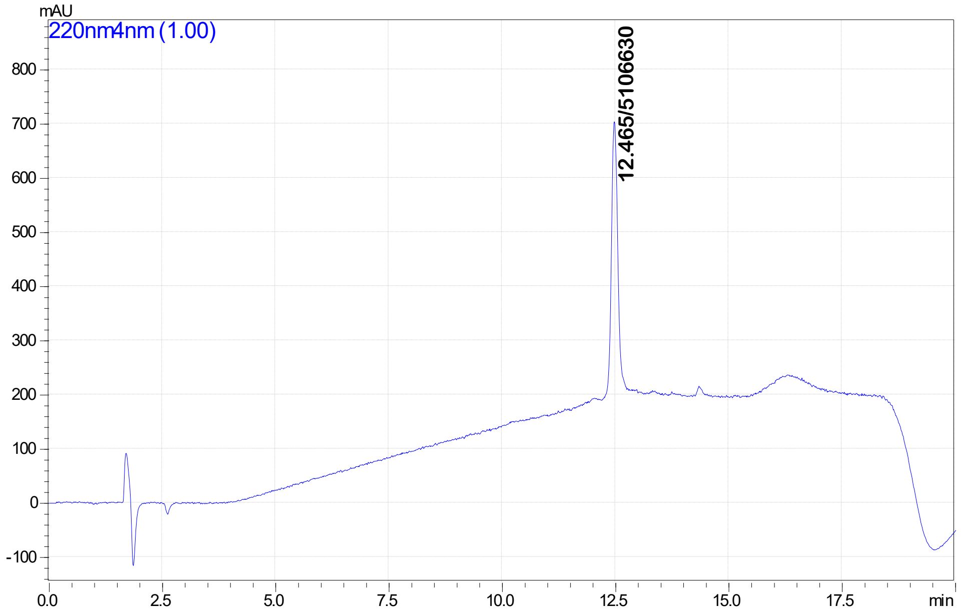

Purity λ: 220 nm >99%

- **U20**

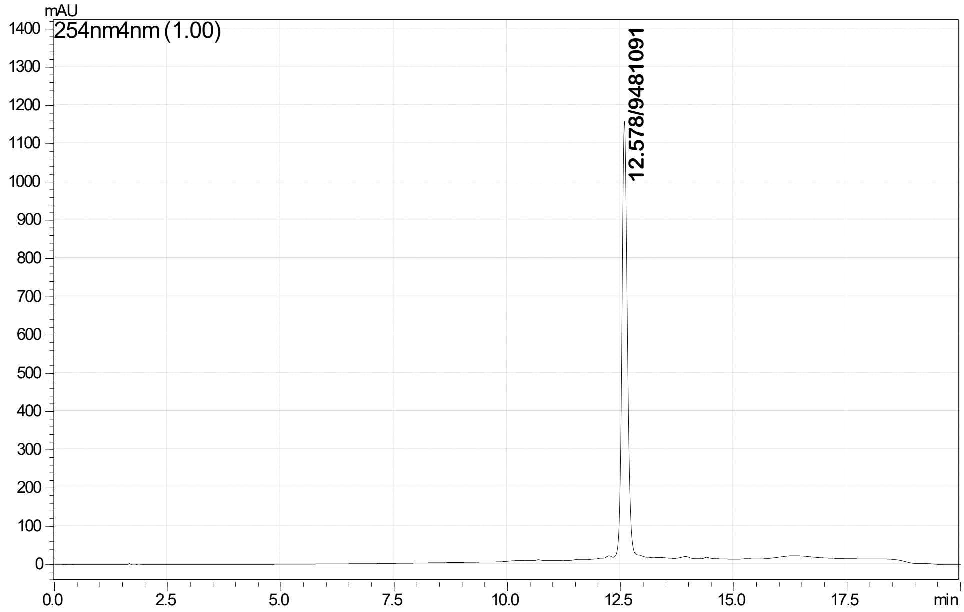

Purity λ: 254 nm >99%

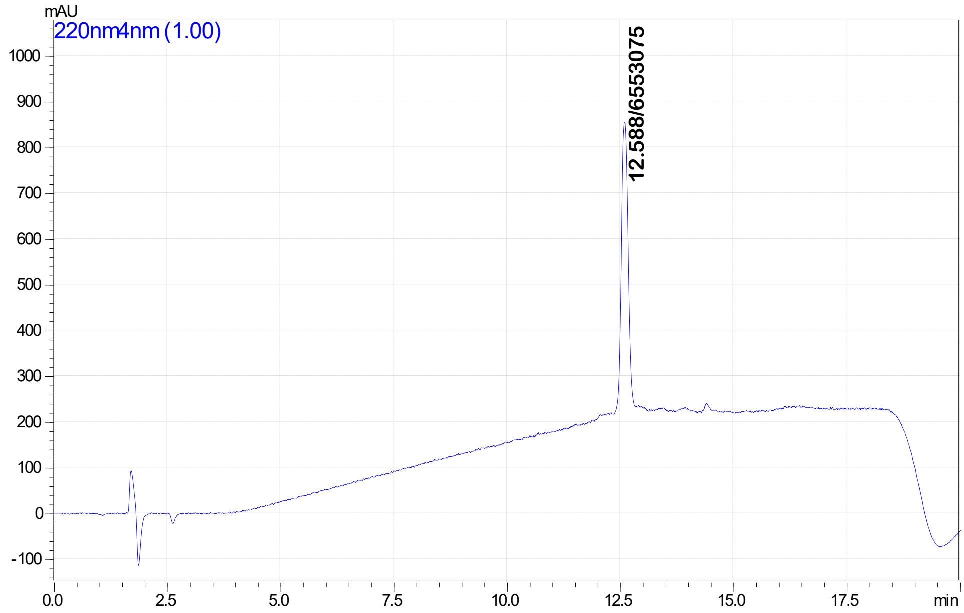

Purity λ: 220 nm >99%

- **U21**

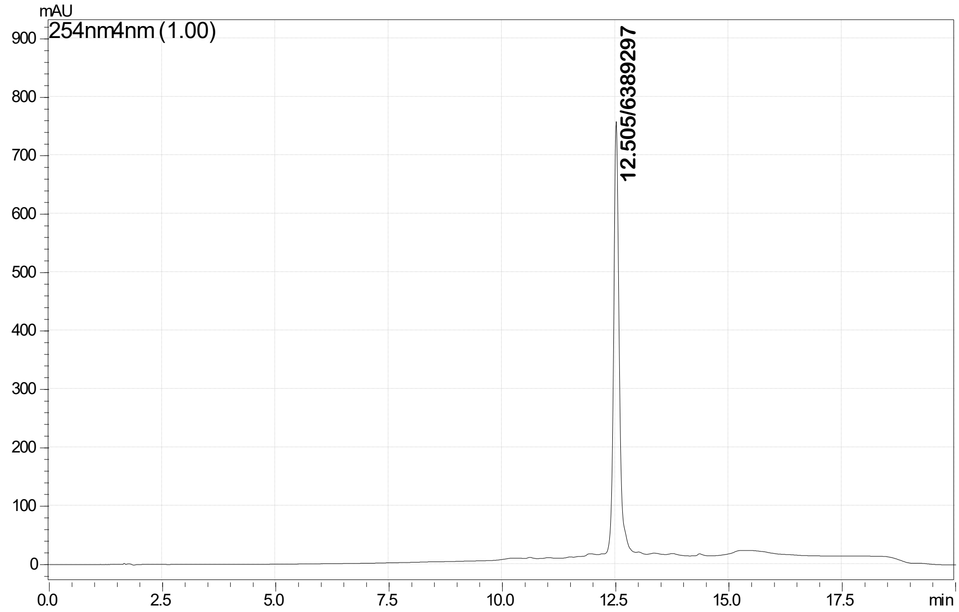

Purity λ: 254 nm >99%

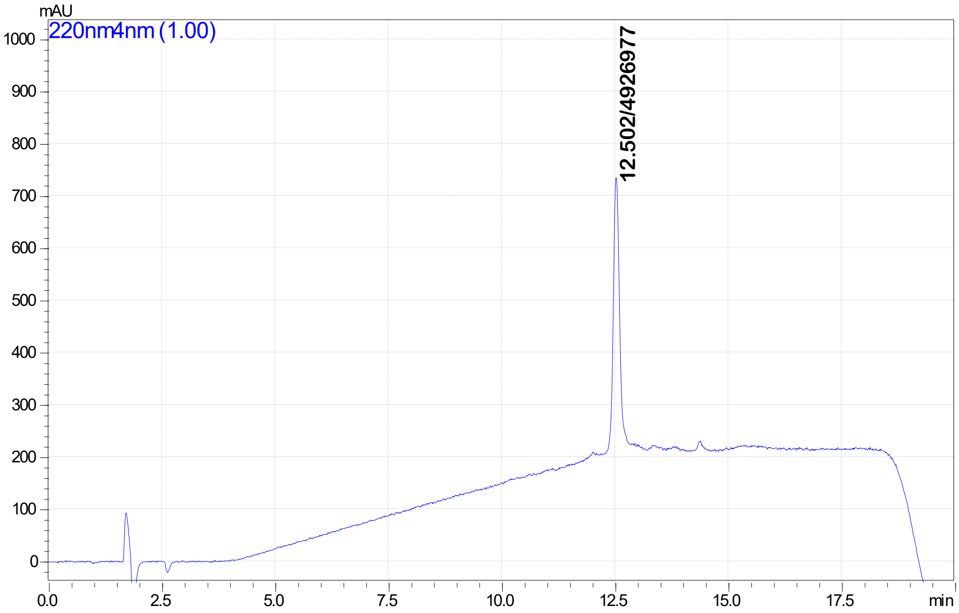

Purity λ: 220 nm >99%

- **U22**

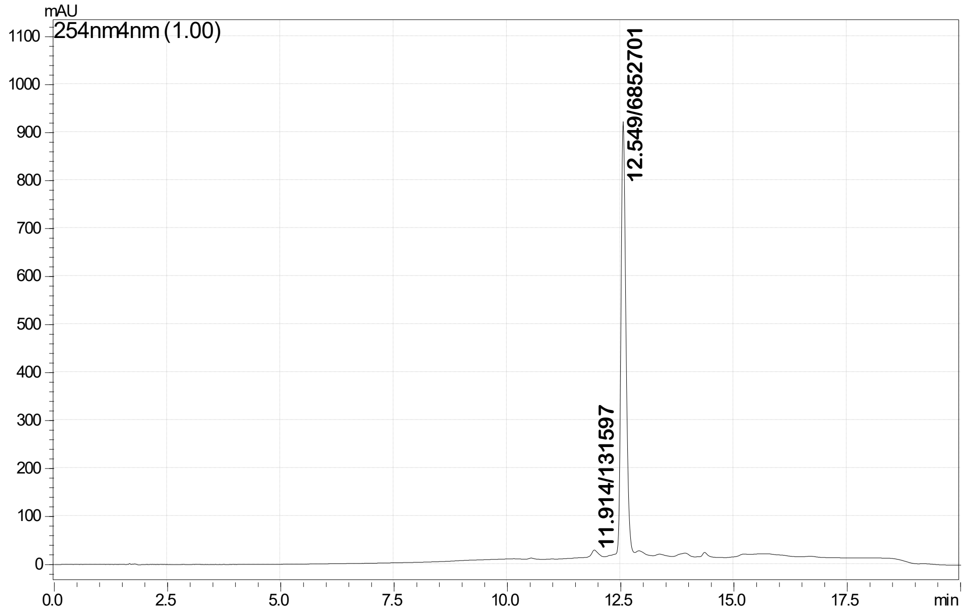

Purity λ: 254 nm: 98.1%

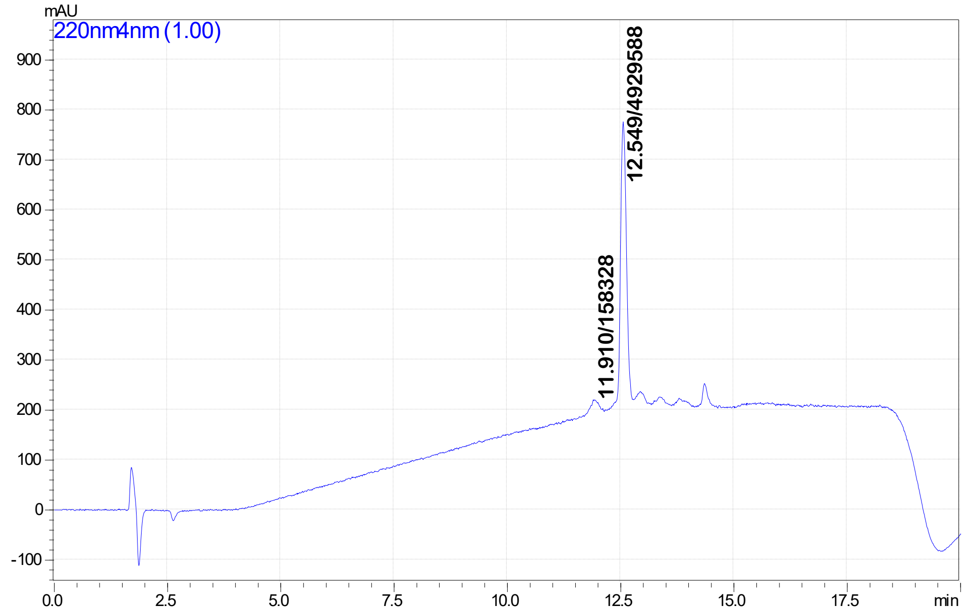

Purity λ: 220 nm: 96.9%

- **U23**

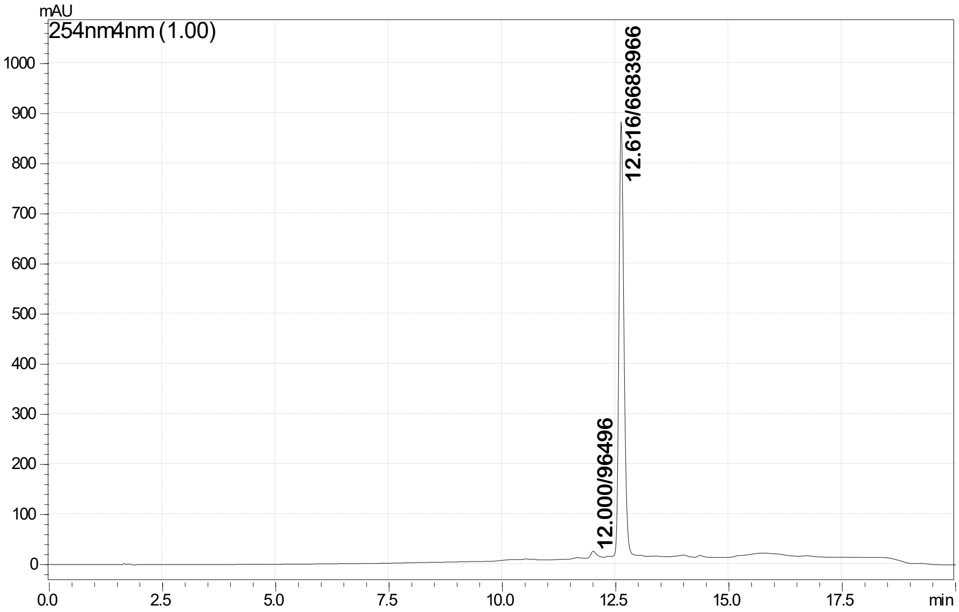

Purity λ: 254 nm= 98.6%

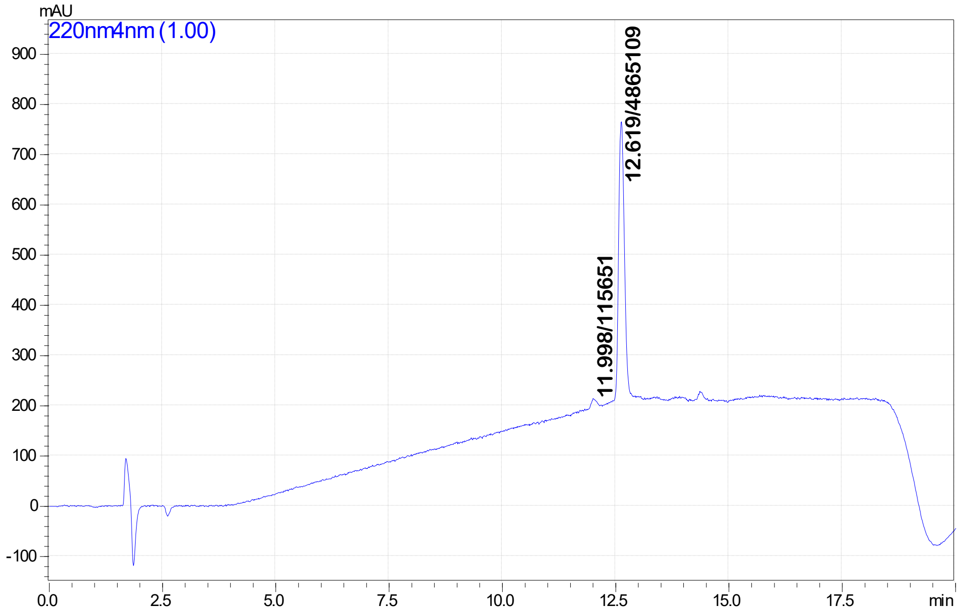

Purity λ: 220 nm= 97.7%

- **U24**

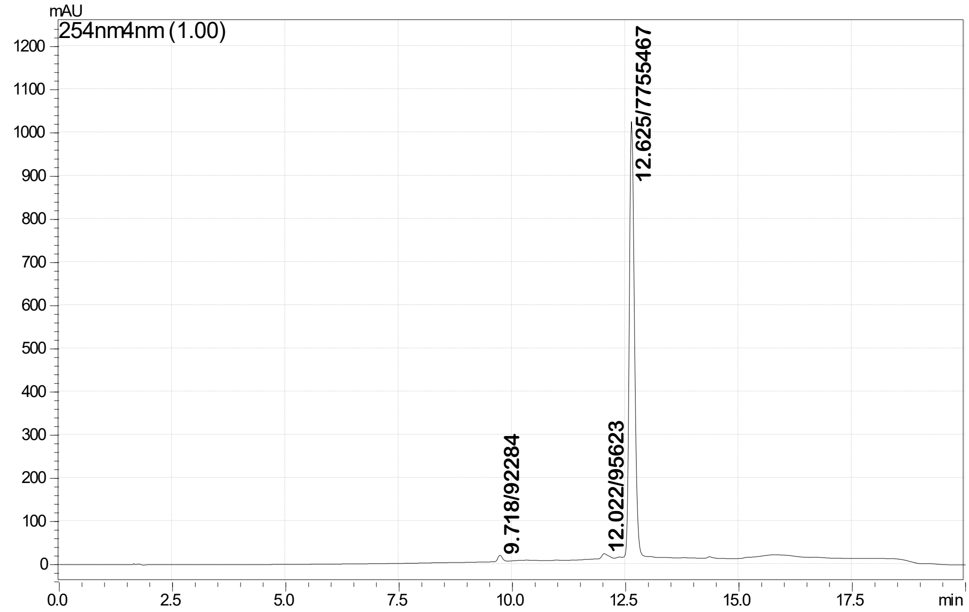

Purity λ: 254 nm=97.6 %

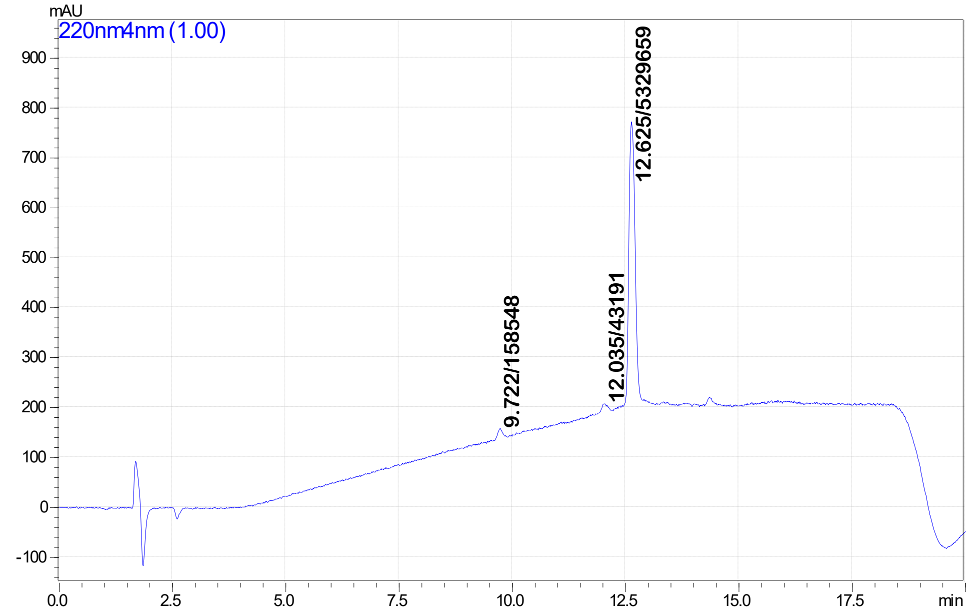

Purity λ: 220 nm=96.4 %

- **U25**

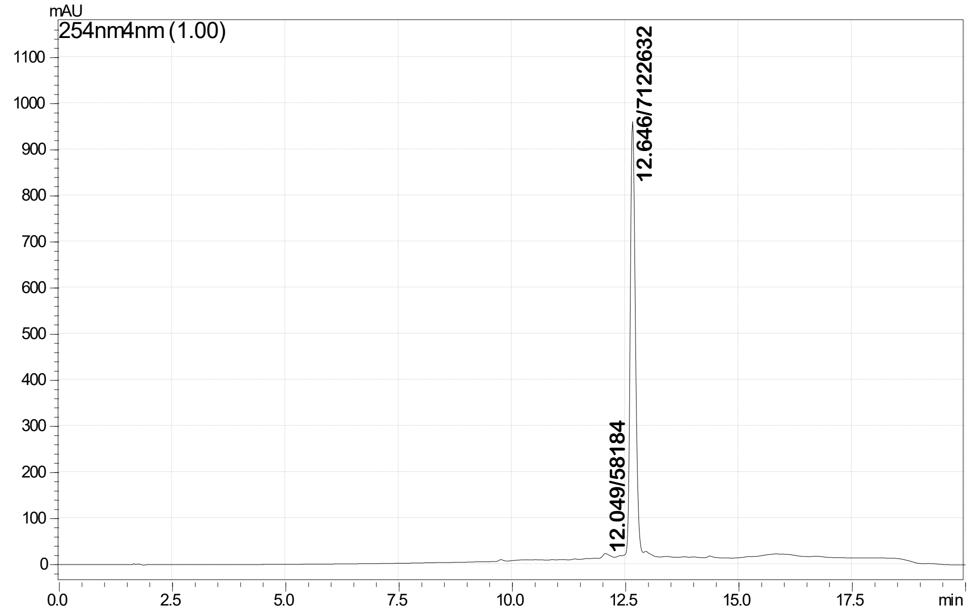

Purity λ: 254 nm=99.2%

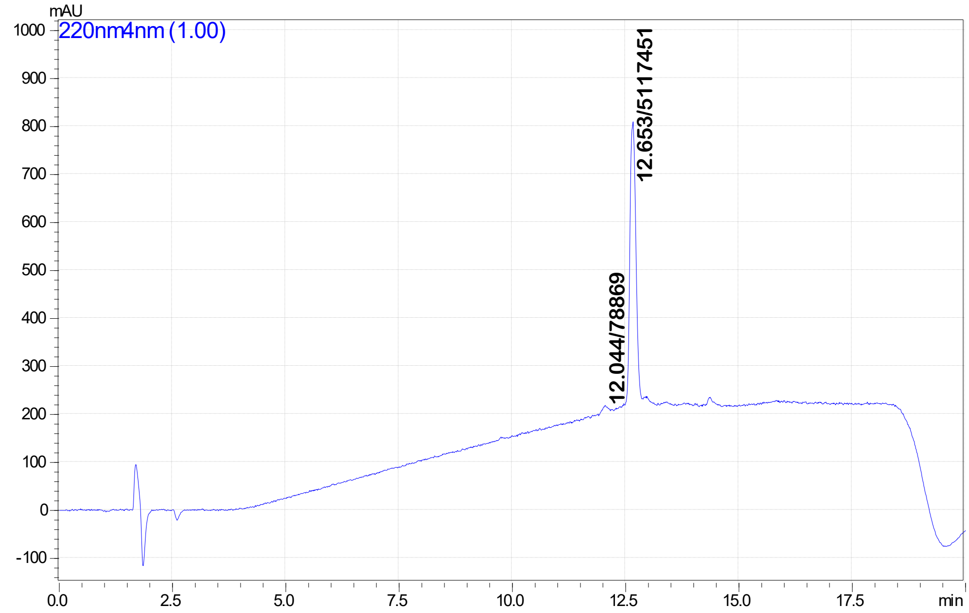

Purity λ: 220 nm=98.5%

- **U30**

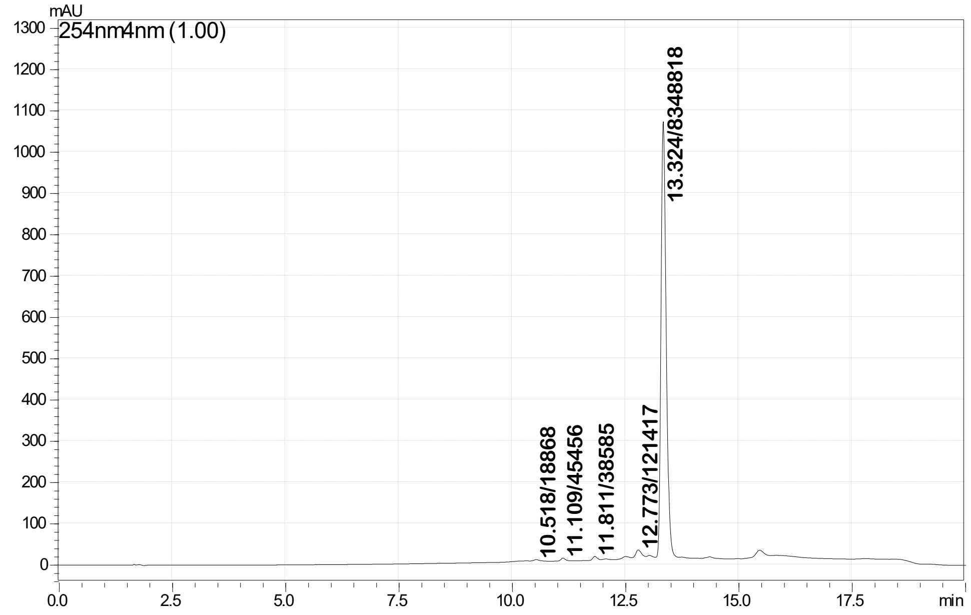

Purity λ: 254 nm=97.4%

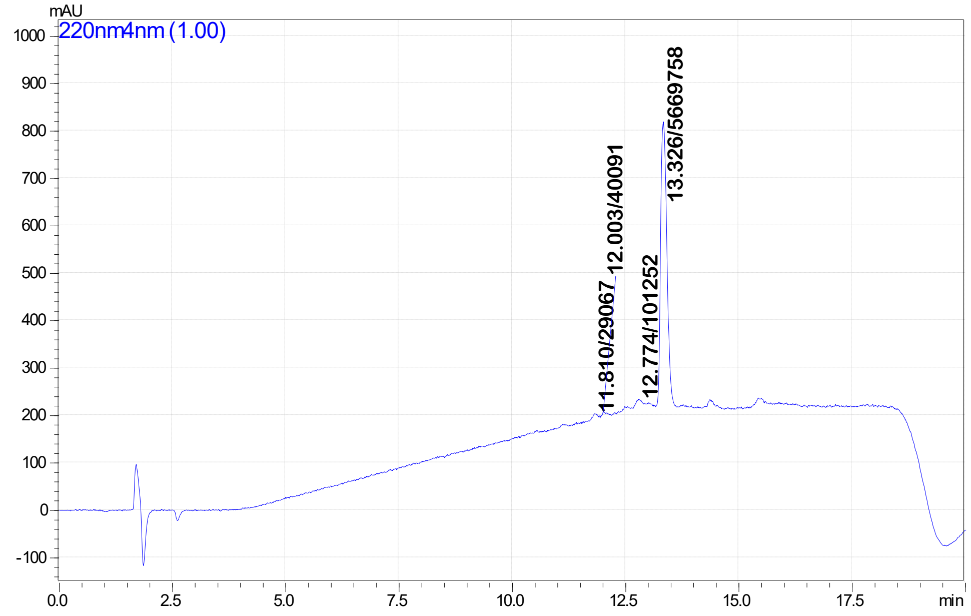

Purity λ: 220 nm=97.0%

- **U31**

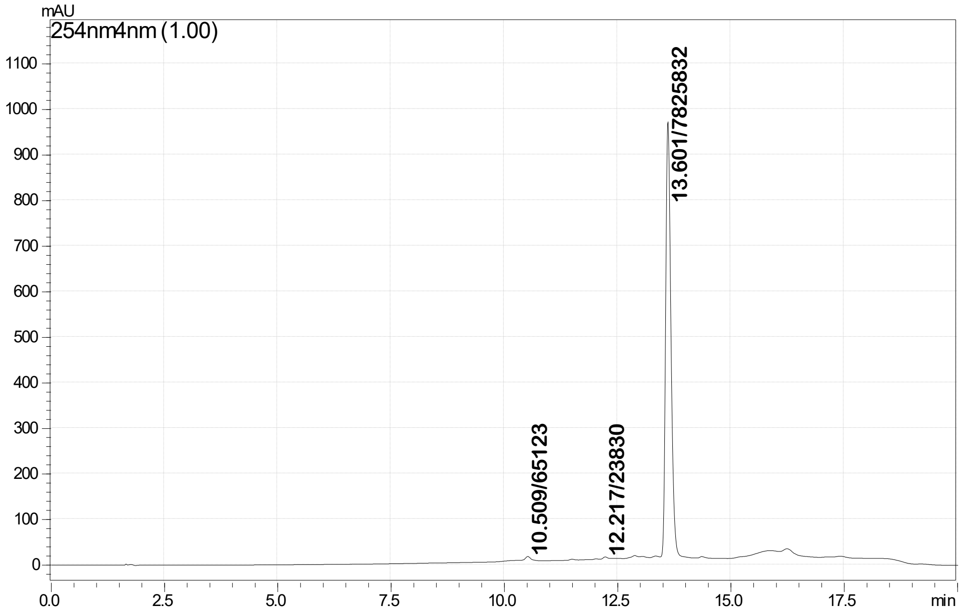

Purity λ: 254 nm=98.9%

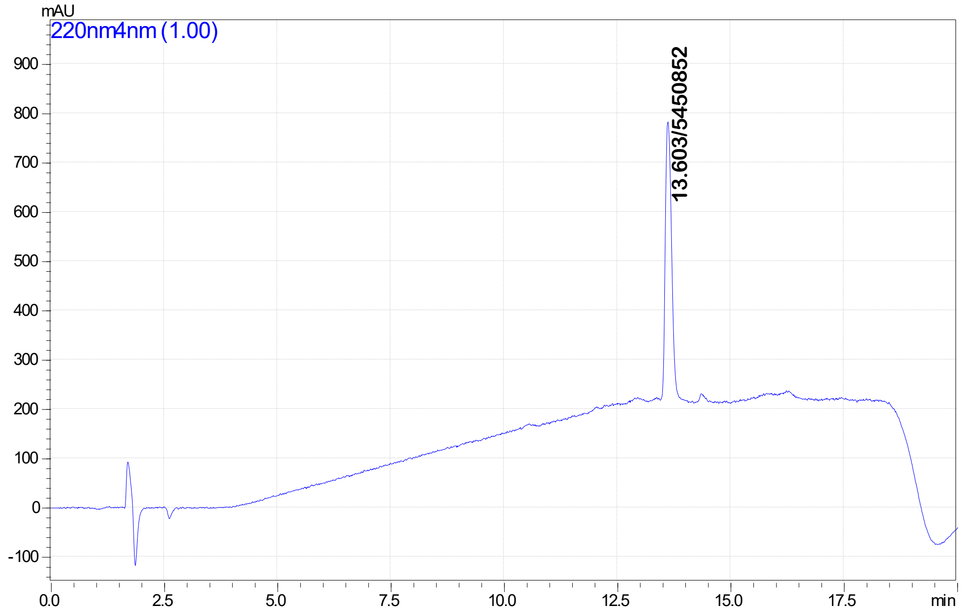

Purity λ: 220 nm >99%

- **U32**

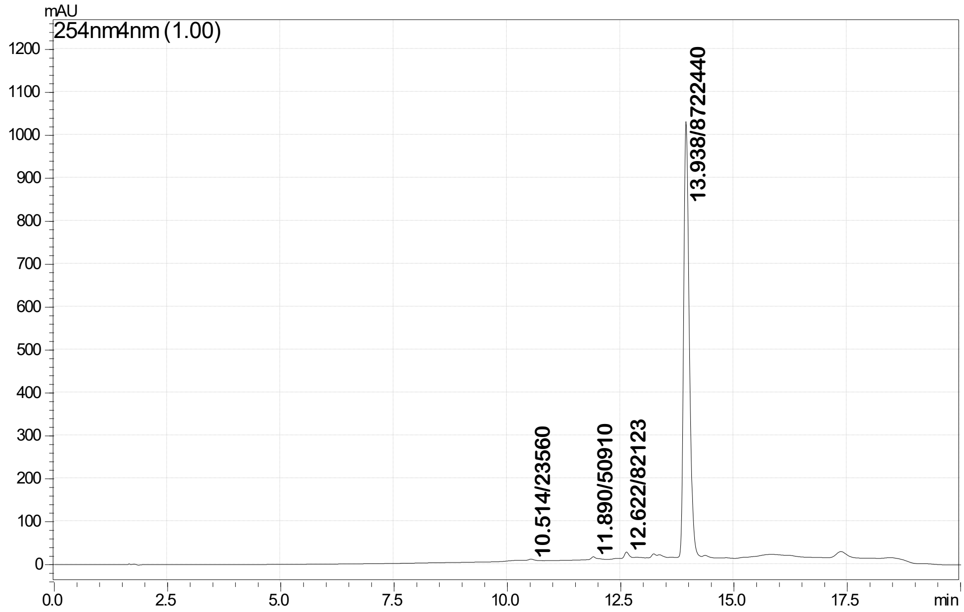

Purity λ: 254 nm=98.2%

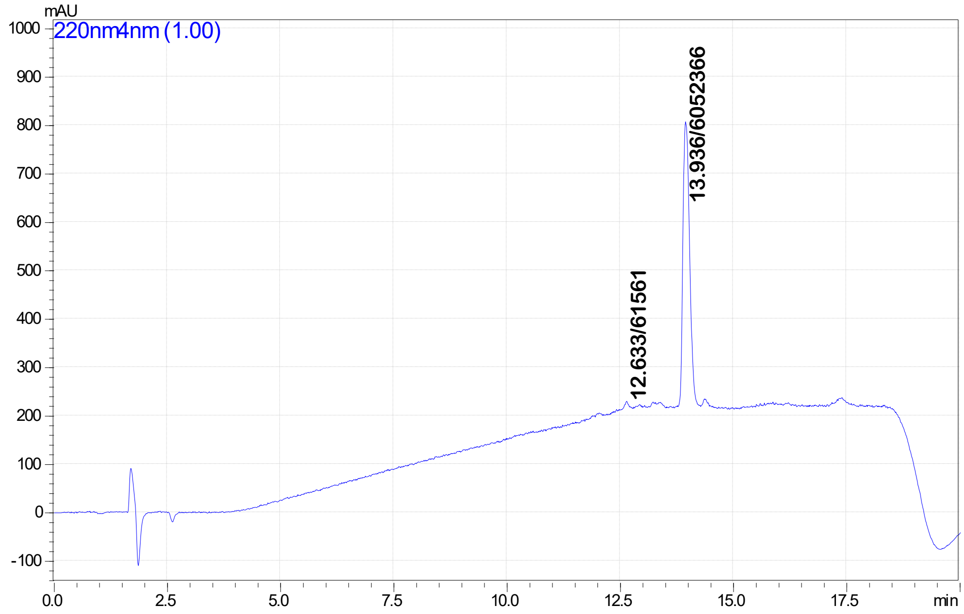

Purity λ: 220 nm=99.0%

- **U33**

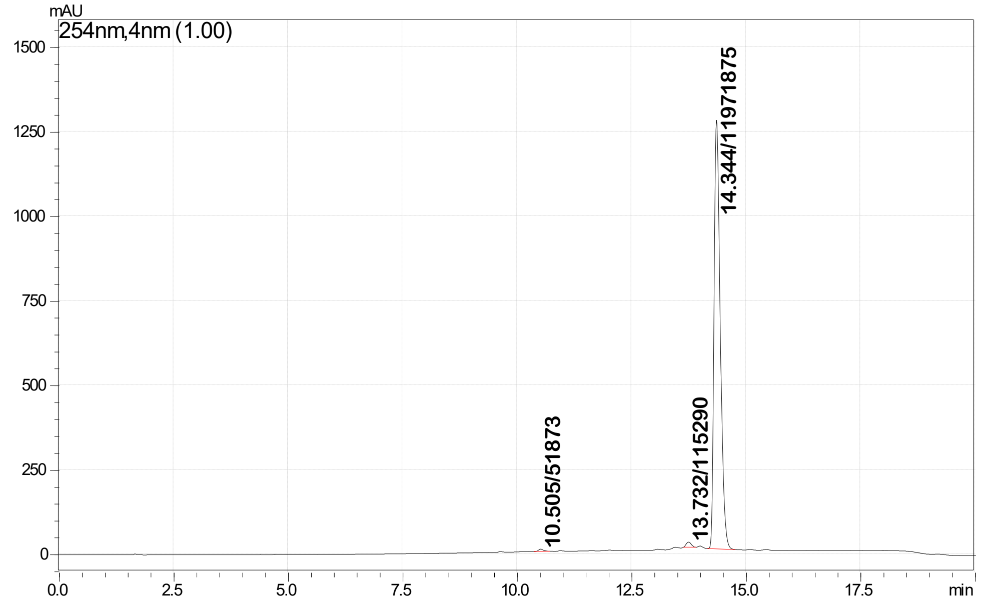

Purity λ: 254 nm=98.6%

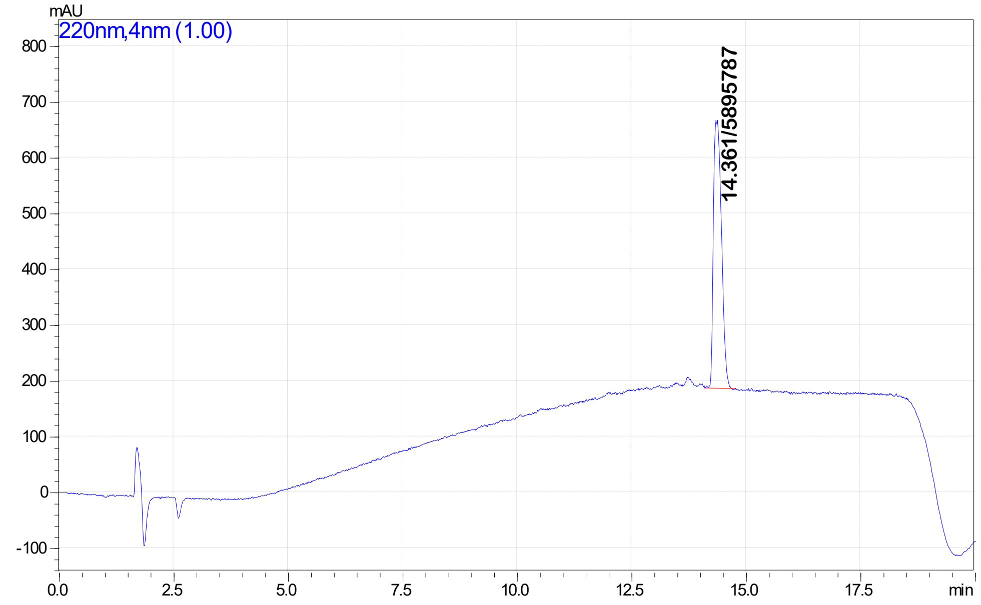

Purity λ: 220 nm>99%

- **U34**

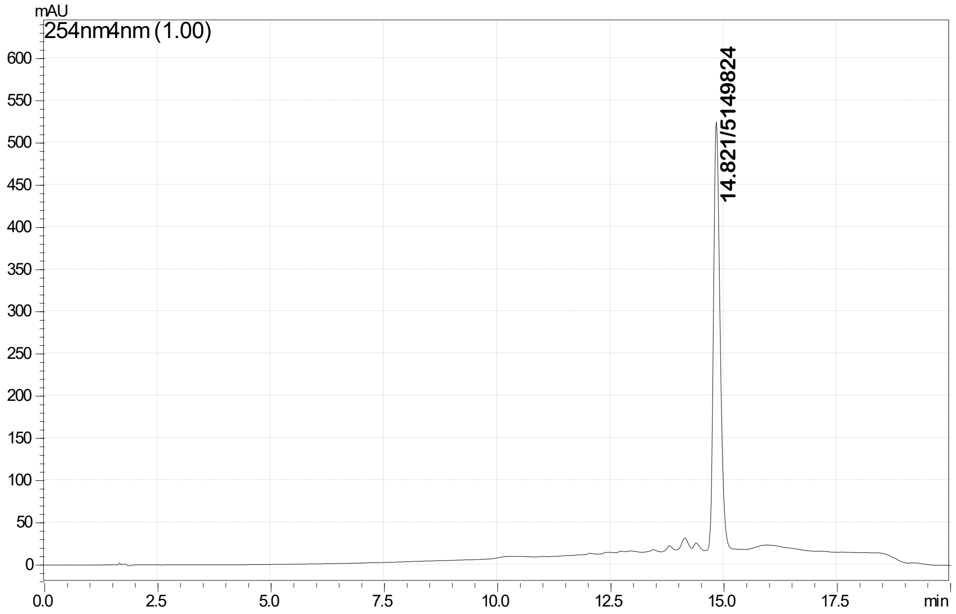

Purity λ: 254 nm >99%

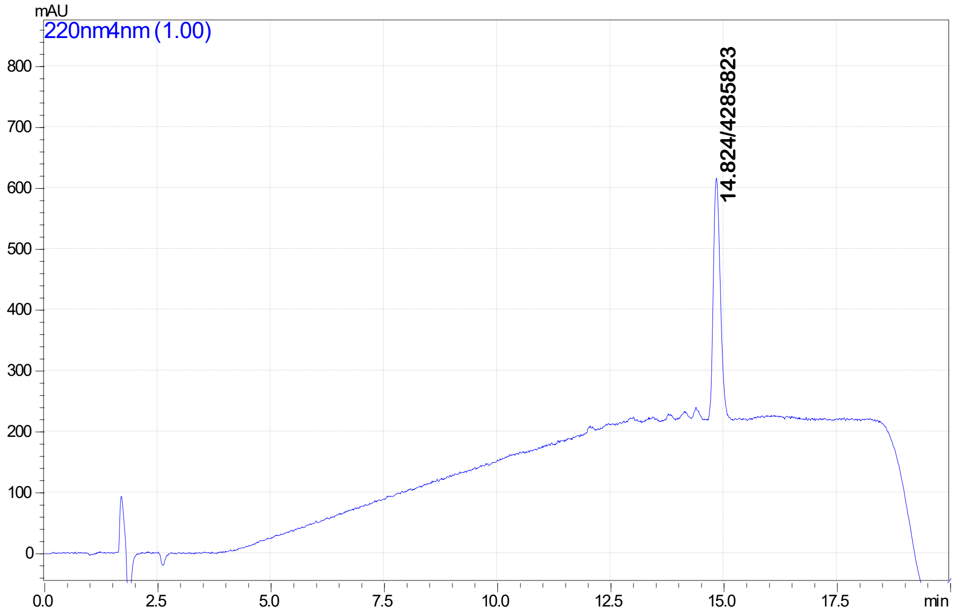

Purity λ: 220 nm >99%

- **U35**

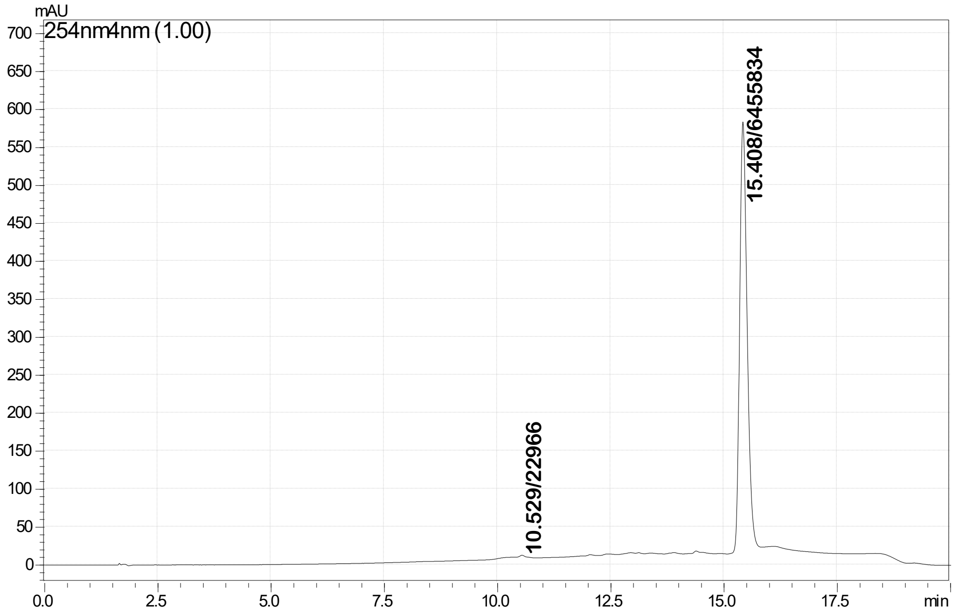

Purity λ: 254 nm >99%

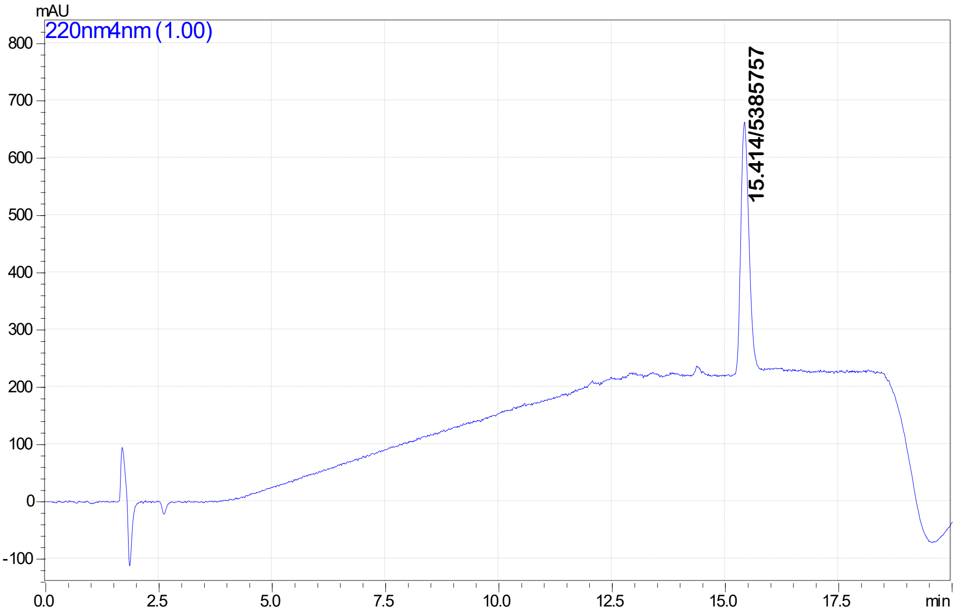

Purity λ: 220 nm >99%

- **U42**

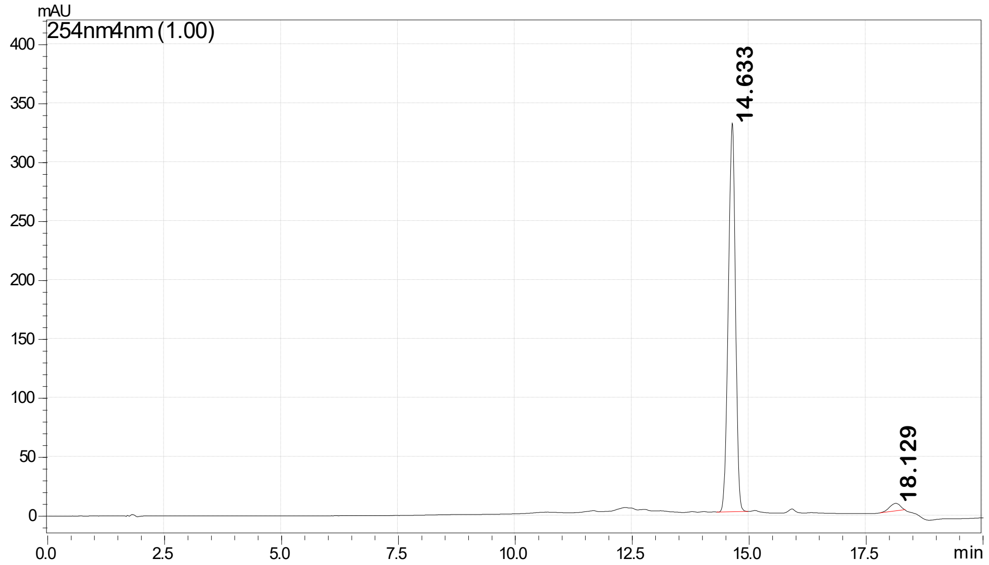

Purity λ: 254 nm >97.5 %

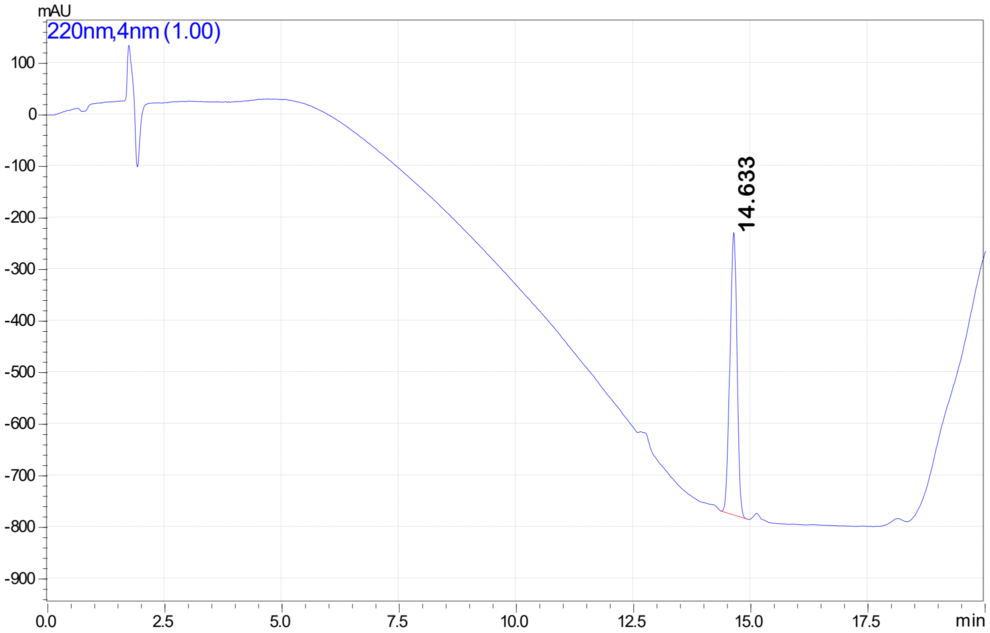

Purity λ: 220 nm >99%

- **Methanol**

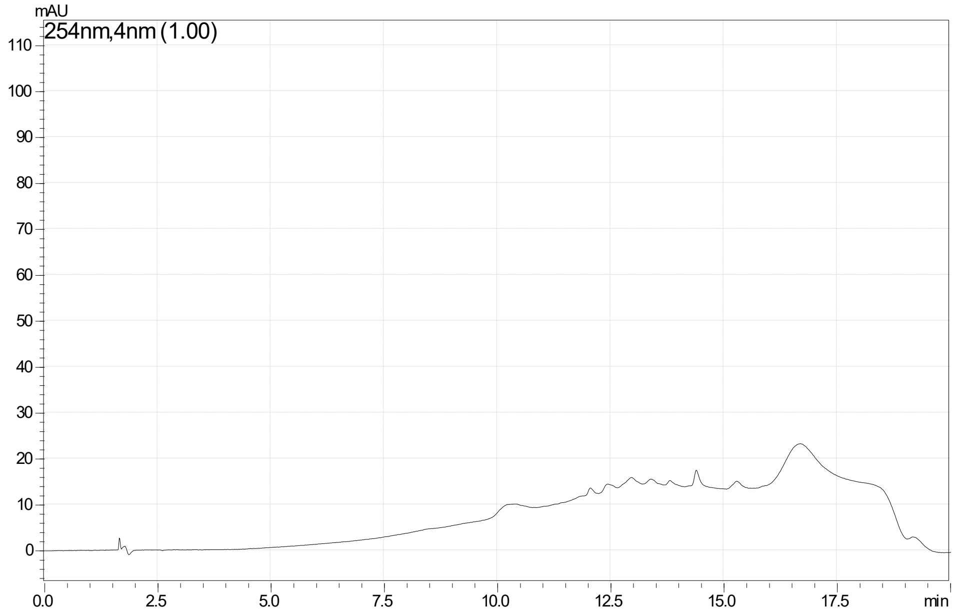
mAU
254nm,4nm (1.00)
110
100
90
80
70
60
50
40
30
20
10
0
0.0
2.5
5.0
7.5
10.0
12.5
15.0
17.5
min

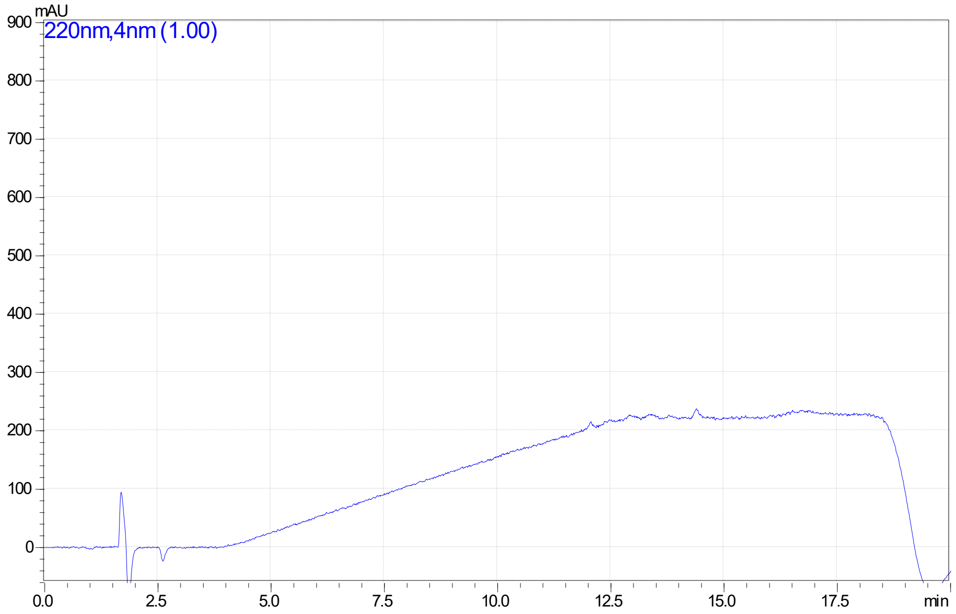
mAU
900
220nm,4nm (1.00)
800
700
600
500
400
300
200
100
0
0.0
2.5
5.0
7.5
10.0
12.5
15.0
17.5
min

LC-UV method for metabolic stability evaluation.

Instrumentation:	Shimadzu HPLC system LC-10AD series
Column:	Syncronis aQ C18 (150 × 4.6 mm, 5 µm d_p) (Thermo Scientific).
Mobile Phase	Phase A: 0.2% formic acid in water HPLC grade
	Phase B: 0.2% formic acid in acetonitrile HPLC grade
Analysis mode:	Gradient of concentration.

Time (min)	% B
0.00	20
11.00	85
14.50	85
15.00	20
20.00	20

Detection:	λ= 254 nm
Flow rate:	1 mL/min
Injecttion volume:	20 µL

LC-HRMS method for pharmacokinetic analysis of compounds U31 and U42.

Instrumentation: Hybrid quadrupole-orbitrap, Thermo Scientific Q-exactive *Plus*, equipped with a Vanquish UHPLC system.

Column: Luna Omega Polar C18 (150 × 2.1 mm, 5 µm d_p) (Phenomenex).

Mobile Phase Phase A: 0.1% formic acid in water UHPLC grade.

Phase B: 0.1% formic acid in acetonitrile UHPLC grade.

Analysis mode: Gradient of concentration.

Time (min)	% B
0.00	20
6.00	60
7.00	95
11.50	95
12.00	20
16.00	20

Flow rate: 0.300 mL/min

Column temperature: 40 °C

Sample temperature: 15 °C

Injection volume: 5 µL

The operating conditions of the HESI were as follows:

Parameter	Value
sheath gas flow rate (N_2)	45 Auxiliary Units
auxiliary gas flow rate	10 Auxiliary Units
sweep gas flow rate	1 Auxiliary Units
spray voltage	3.50 kV
capillary temperature	300 °C
auxiliary gas heater temperature	350 °C

The detector acquisition parameters were the followings:

+ Full scan-SIM	Value
Microscan	1
Resolution	35,000
AGC target	1e5
Maximum IT	100 ms
Isolation wind.	2.5 *m/z*
Isolation offset	1.1 *m/z*

LC-HRMS method for metabolites identification of compound U42.

Instrumentation:	Hybrid quadrupole-orbitrap, Thermo Scientific Q-exactive *Plus*, equipped with a Vanquish UHPLC system.
Column:	Luna Omega Polar C18 (150 × 2.1 mm, 5 µm d_p) (Phenomenex).
Mobile Phase	Phase A: 0.1% formic acid in water UHPLC grade.<br>Phase B: 0.1% formic acid in acetonitrile UHPLC grade.
Analysis mode:	Gradient of concentration.

Time (min)	% B
0.00	20
16.00	60
17.00	80
19.50	80
20.00	20
25.00	20

Flow rate:	0.250 mL/min
Column temperature:	40 °C
Sample temperature:	15 °C
Injection volume:	5 µL

The operating conditions of the HESI were as follows:

Parameter	Value
sheath gas flow rate (N_2)	45 Auxiliary Units
auxiliary gas flow rate	10 Auxiliary Units
sweep gas flow rate	1 Auxiliary Units
spray voltage	3.50 kV
capillary temperature	300 °C
auxiliary gas heater temperature	350 °C

The detector acquisition parameters were the followings:

**+ Full scan**	**Value**
Microscan	1
Resolution	70,000
AGC target	3e6
Maximum IT	200 ms

**+PRM (MS^2)**	**Value**
Microscan	1
Resolution	35,000
AGC target	1e5
Maximum IT	120 ms
Loop count	1
MSX count	1
Isolation wind.	4.5 *m/z*
Isolation offset	2.0 *m/z*
NCE	20, 30, 50

**+ ddMS^2**	**Value**
Microscan	1
Resolution	17,500
AGC target	1e5
Maximum IT	60 ms
Loop count	4
MSX count	1
Isolation wind.	4.5 *m/z*
Isolation offset	2.0 *m/z*
NCE	20, 30, 50

LC-HRMS data of U42 and its metabolites in MLM and HLM.

- **U42**

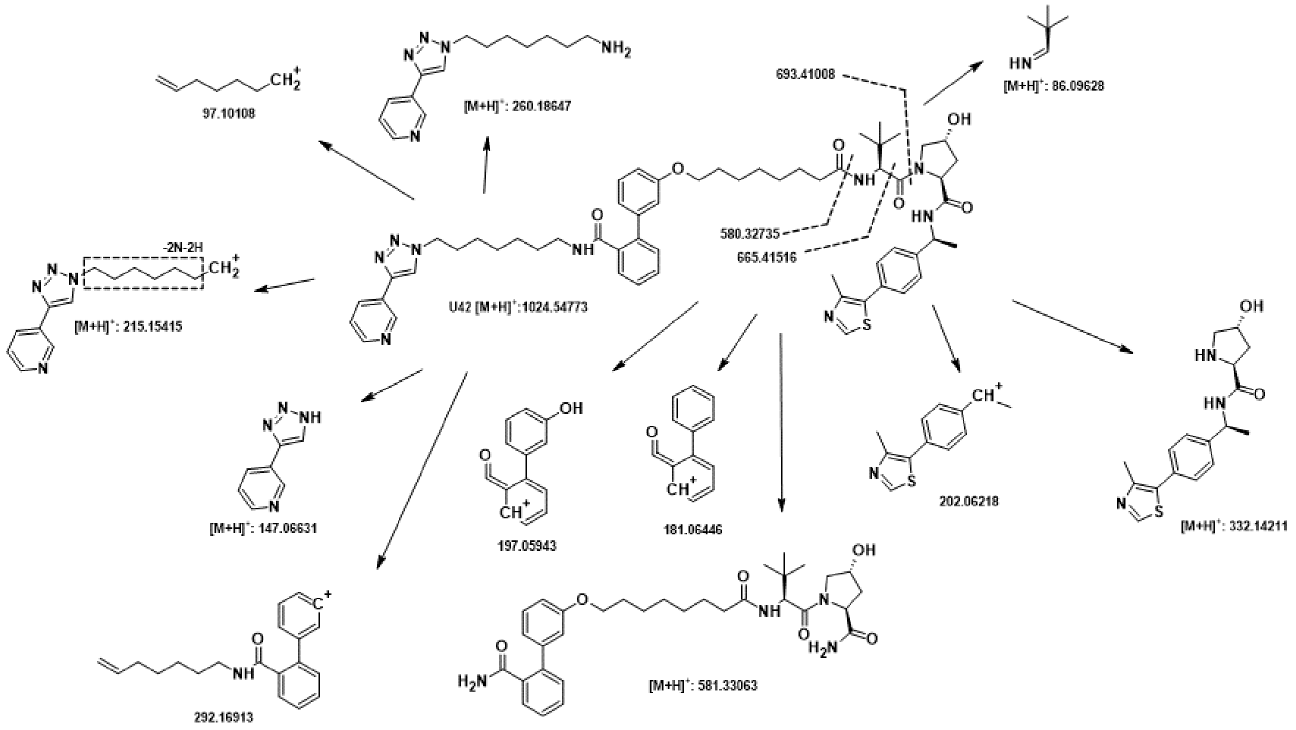

U42 [M+H]$^{+}$= 1024.54773

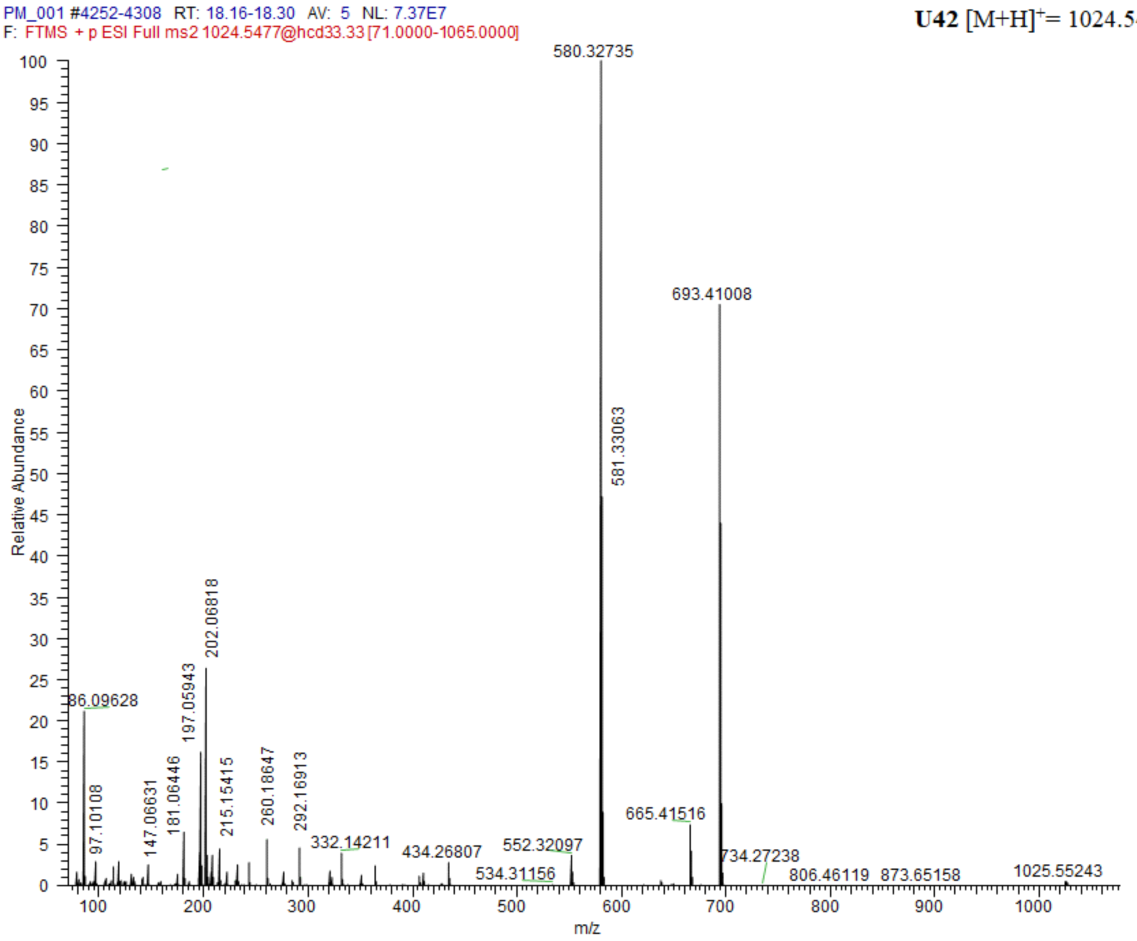

- **M1-M3**

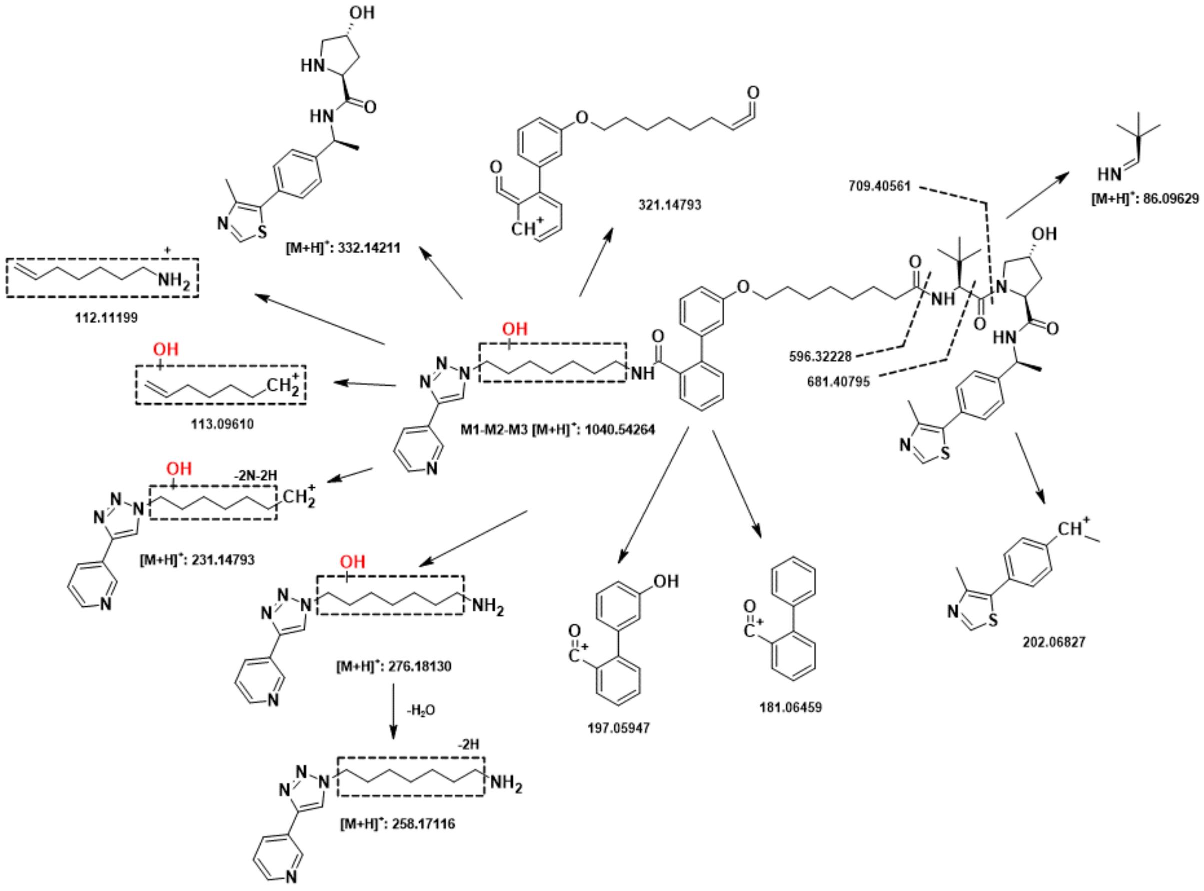
[M+H]+: 332.14211
321.14793
709.40561
[M+H]+: 86.09629
112.11199
113.09610
596.32228
681.40795
M1-M2-M3 [M+H]+: 1040.54264
-2N-2H
[M+H]+: 231.14793
[M+H]+: 276.18130
-H2O
-2H
[M+H]+: 258.17116
197.05947
181.06459
202.06827

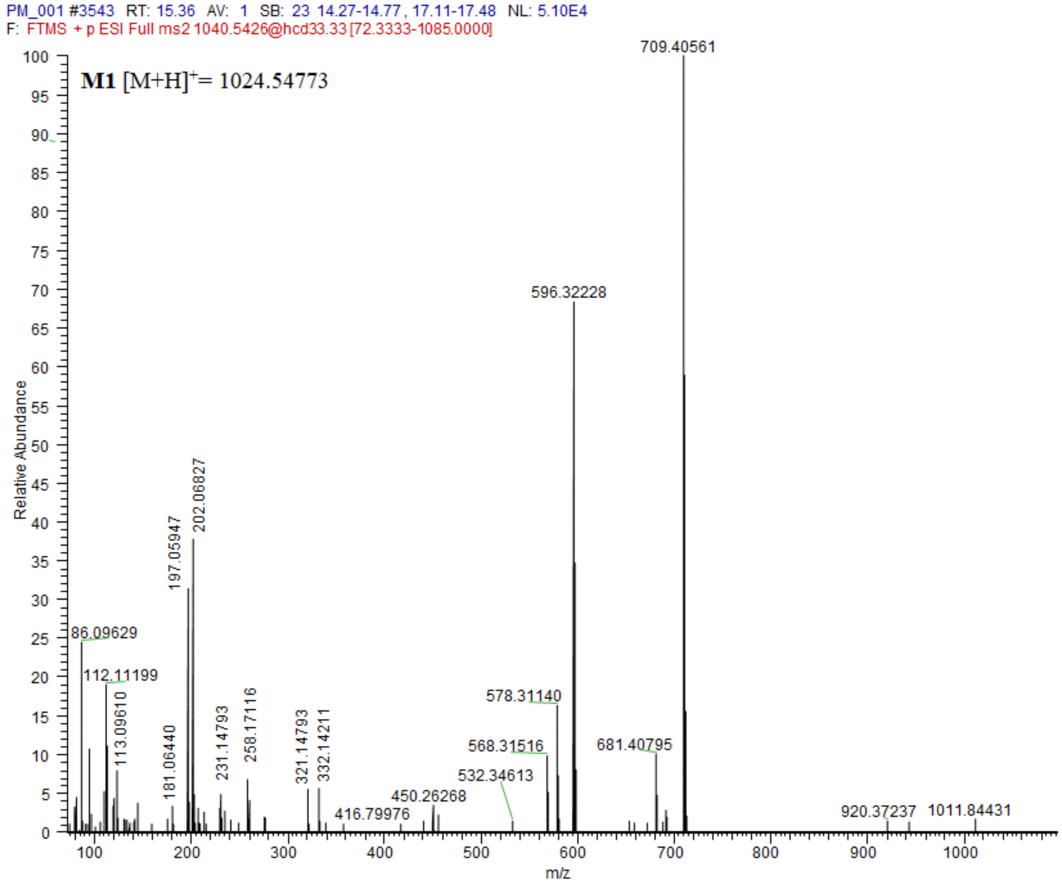
PM_001 #3543 RT: 15.36 AV: 1 SB: 23 14.27-14.77 , 17.11-17.48 NL: 5.10E4
F: FTMS + p ESI Full ms2 1040.5426@hcd33.33 [72.3333-1085.0000]
M1 [M+H]+= 1024.54773
Relative Abundance
m/z
709.40561
596.32228
202.06827
197.05947
86.09629
112.11199
113.09610
181.06440
231.14793
258.17116
321.14793
332.14211
416.79976
450.26268
532.34613
568.31516
578.31140
681.40795
920.37237
1011.84431

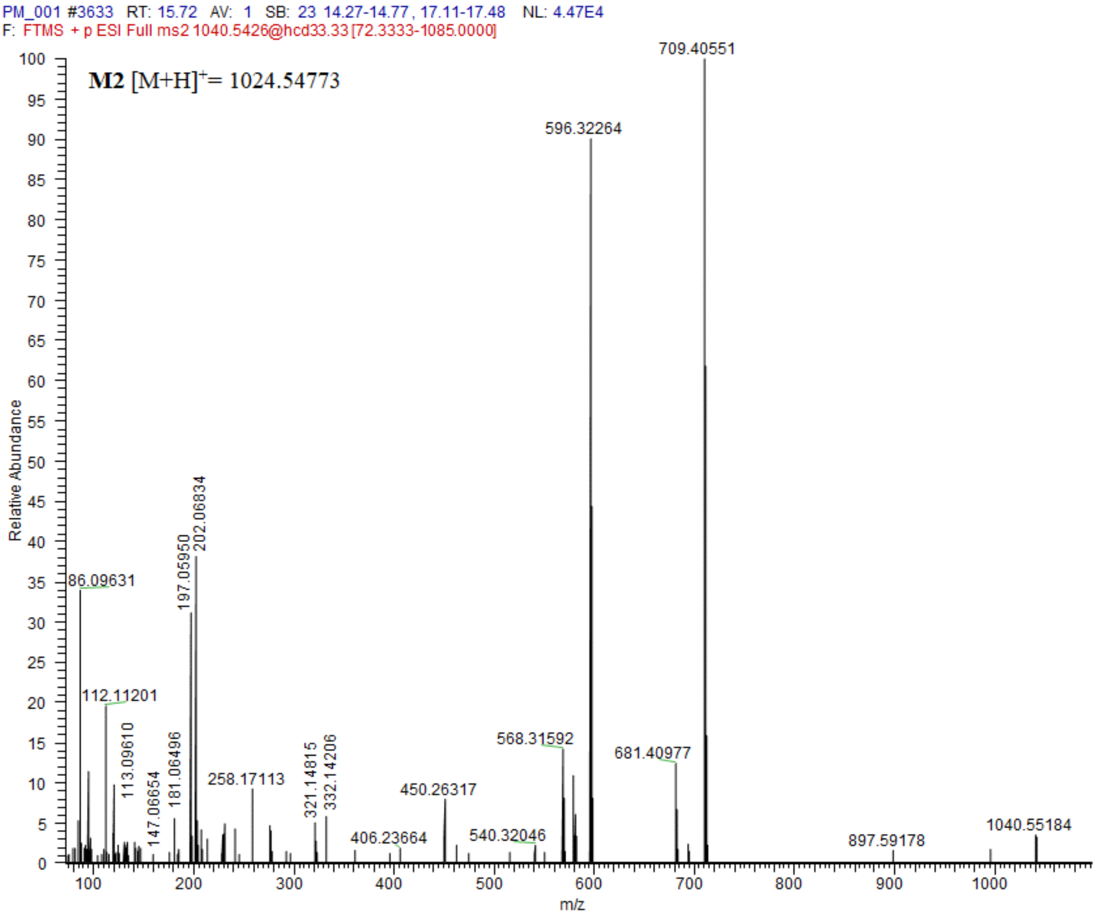

PM_001 #3633 RT: 15.72 AV: 1 SB: 23 14.27-14.77 , 17.11-17.48 NL: 4.47E4
F: FTMS + p ESI Full ms2 1040.5426@hcd33.33 [72.3333-1085.0000]
M2 [M+H]+= 1024.54773
Relative Abundance
m/z
709.40551
596.32264
86.09631
197.05950
202.06834
112.11201
113.09610
147.06654
181.06496
258.17113
321.14815
332.14206
406.23664
450.26317
540.32046
568.31592
681.40977
897.59178
1040.55184

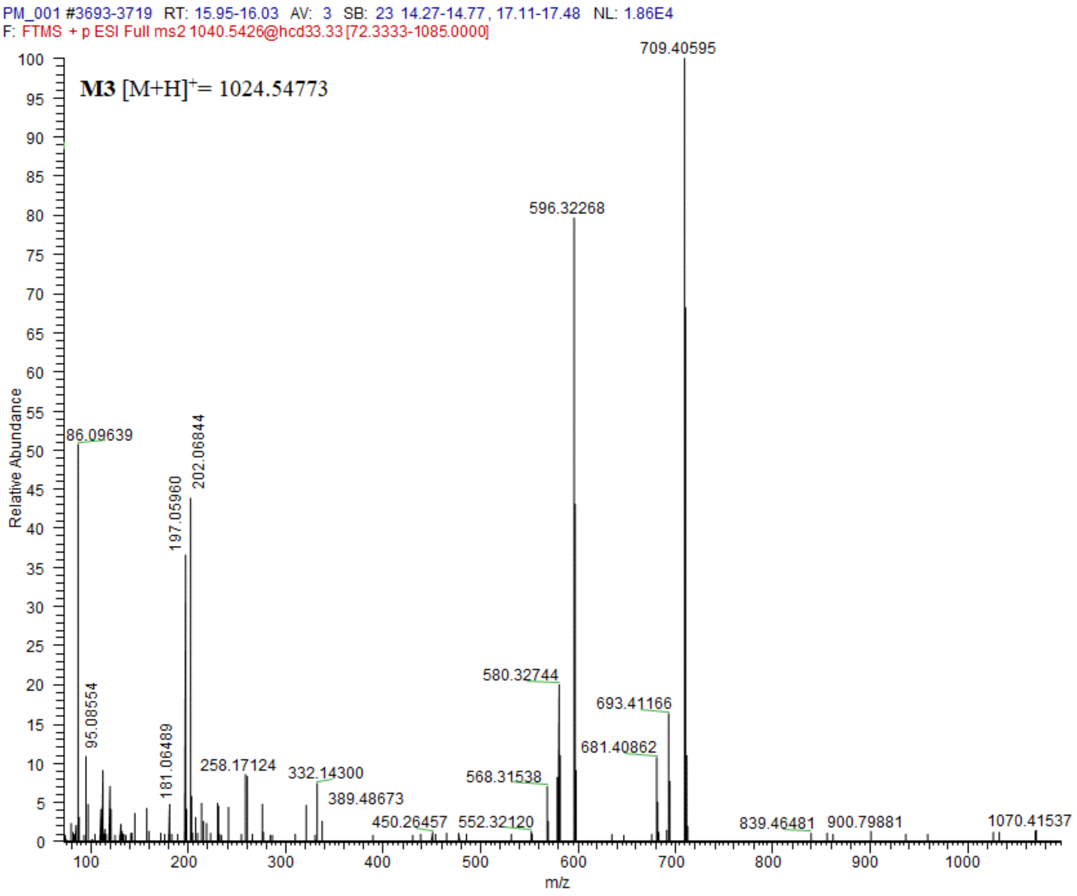

PM_001 #3693-3719 RT: 15.95-16.03 AV: 3 SB: 23 14.27-14.77 , 17.11-17.48 NL: 1.86E4
F: FTMS + p ESI Full ms2 1040.5426@hcd33.33 [72.3333-1085.0000]
M3 [M+H]+= 1024.54773
Relative Abundance
m/z
709.40595
596.32268
86.09639
197.05960
202.06844
95.08554
181.06489
258.17124
332.14300
389.48673
450.26457
552.32120
568.31538
580.32744
693.41166
681.40862
839.46481
900.79881
1070.41537

- **M4**

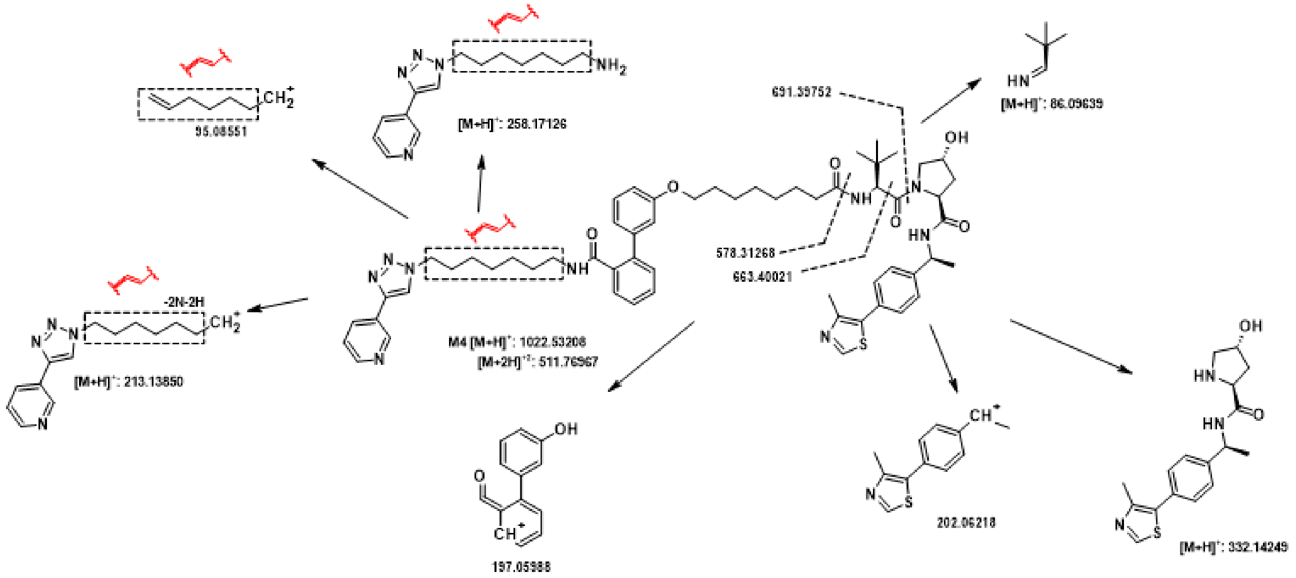

M4 [M+2H]$^{+2}$= 511.76967

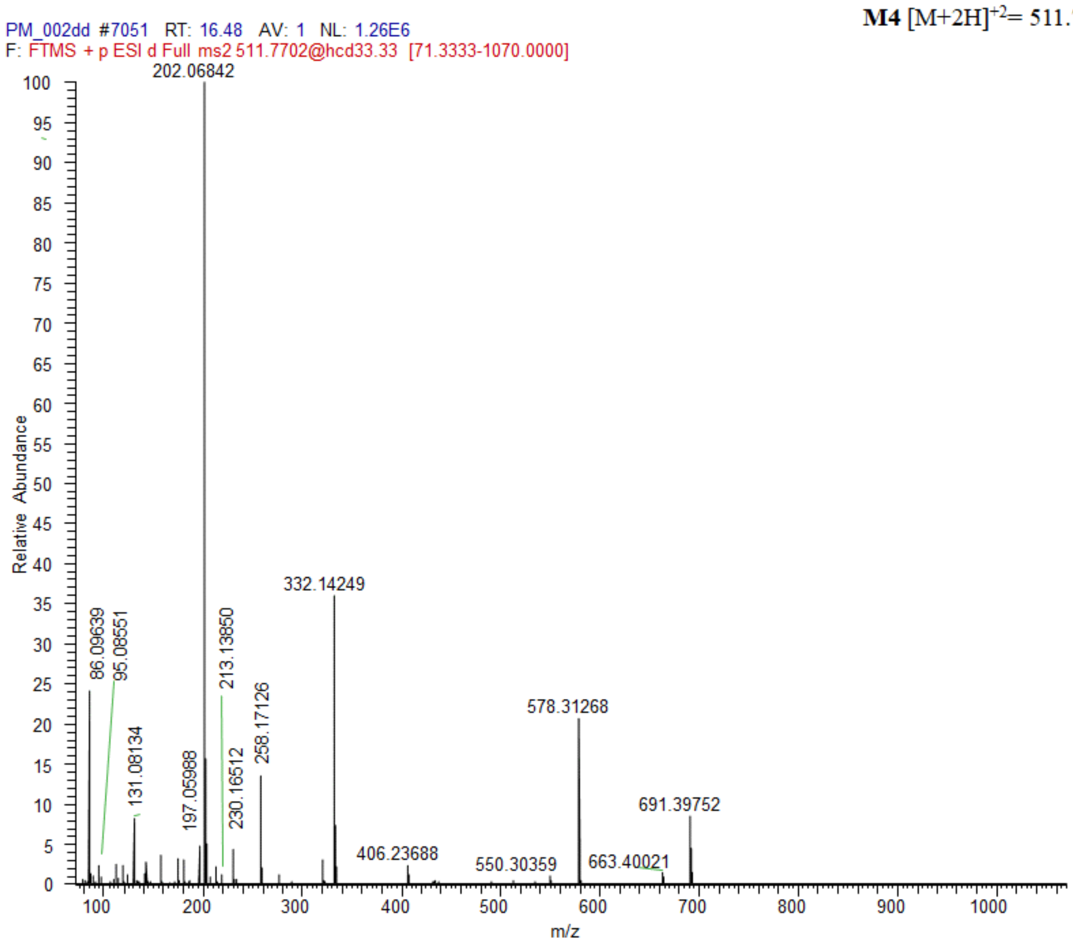

- **M5**

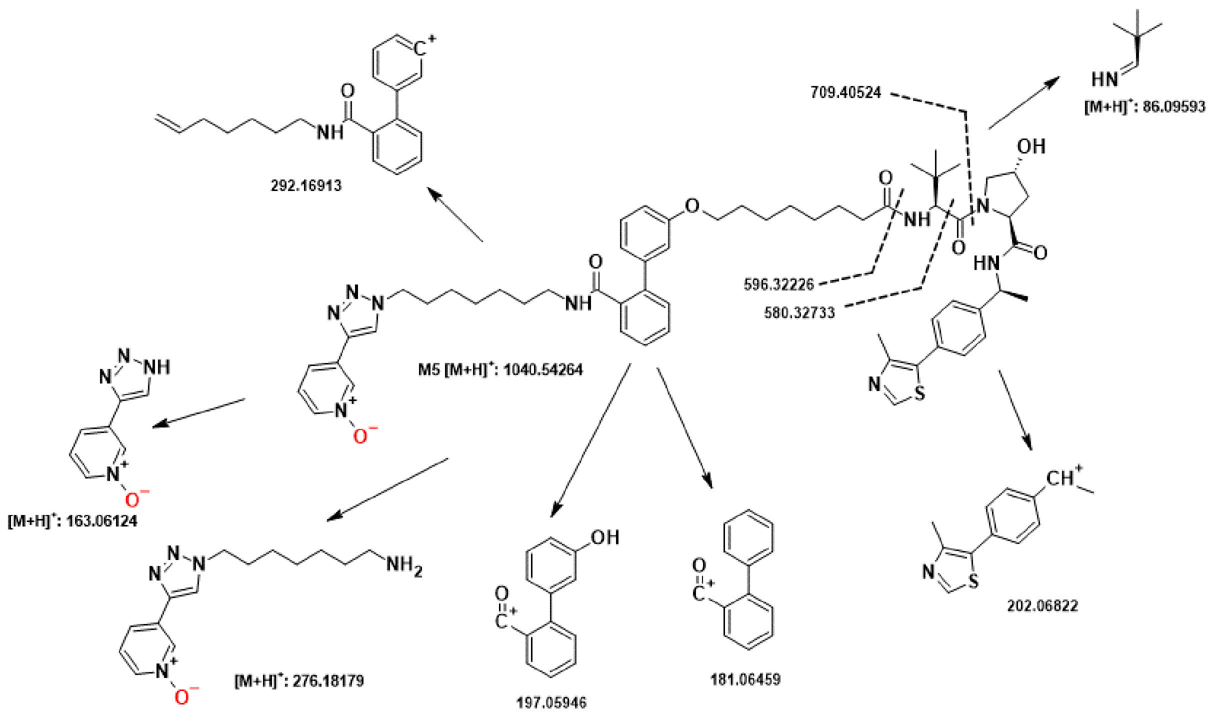
292.16913
709.40524
[M+H]+: 86.09593
M5 [M+H]+: 1040.54264
596.32226
580.32733
[M+H]+: 163.06124
[M+H]+: 276.18179
197.05946
181.06459
202.06822

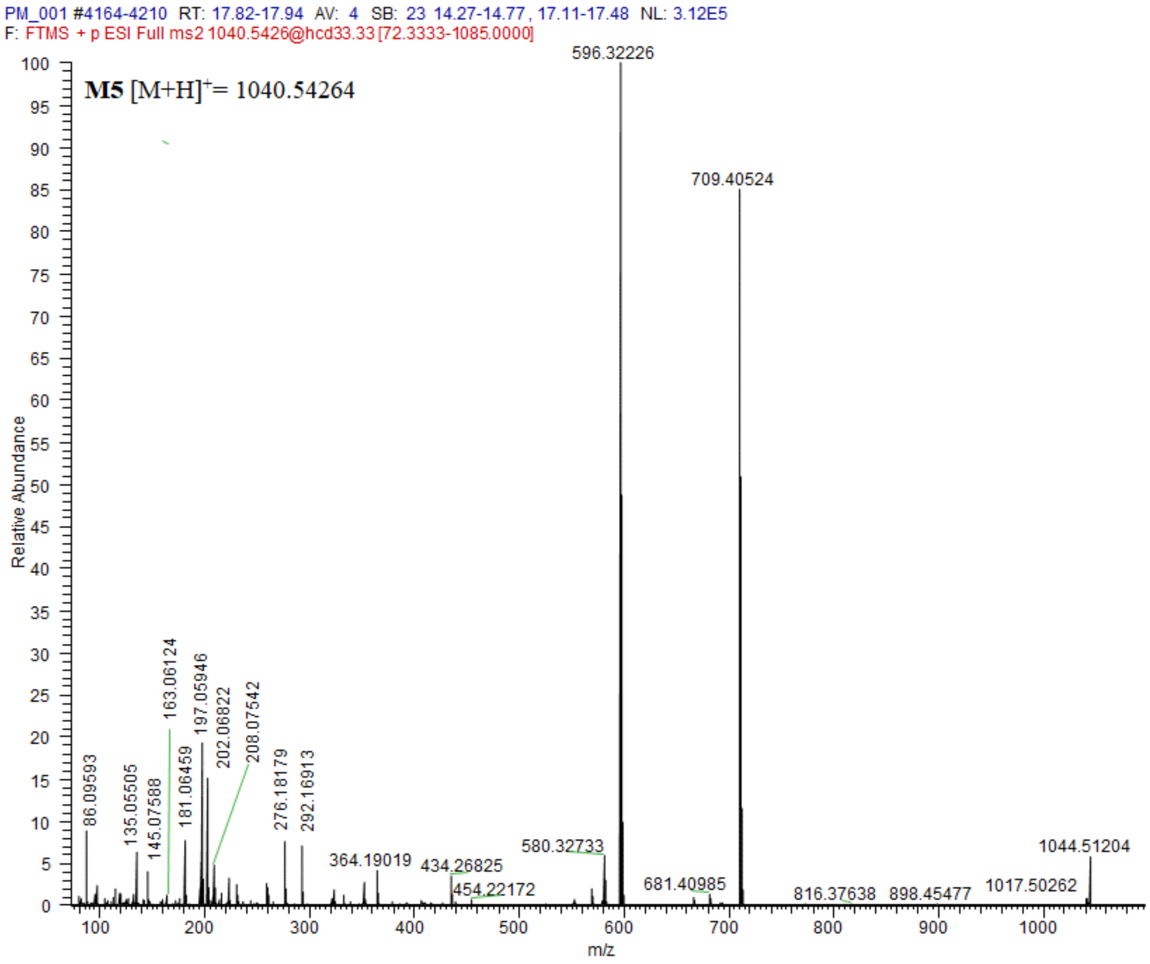
PM_001 #4164-4210 RT: 17.82-17.94 AV: 4 SB: 23 14.27-14.77 , 17.11-17.48 NL: 3.12E5
F: FTMS + p ESI Full ms2 1040.5426@hcd33.33 [72.3333-1085.0000]
M5 [M+H]+= 1040.54264
Relative Abundance
m/z
596.32226
709.40524
86.09593
135.05505
145.07588
163.06124
181.06459
197.05946
202.06822
208.07542
276.18179
292.16913
364.19019
434.26825
454.22172
580.32733
681.40985
816.37638
898.45477
1017.50262
1044.51204

- **M6**

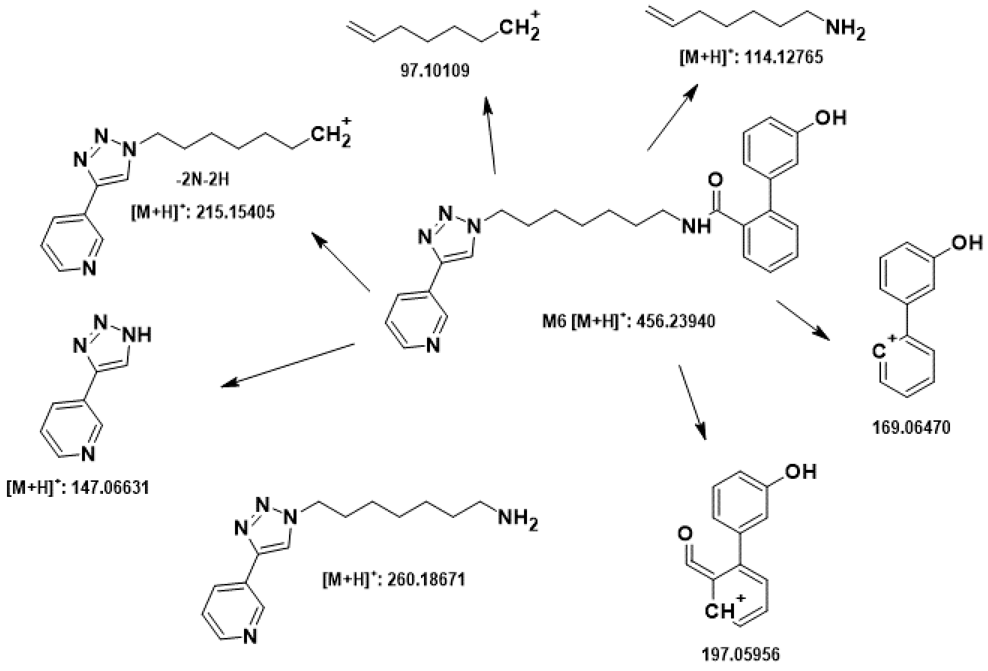

97.10109
[M+H]+: 114.12765
-2N-2H
[M+H]+: 215.15405
M6 [M+H]+: 456.23940
169.06470
[M+H]+: 147.06631
[M+H]+: 260.18671
197.05956

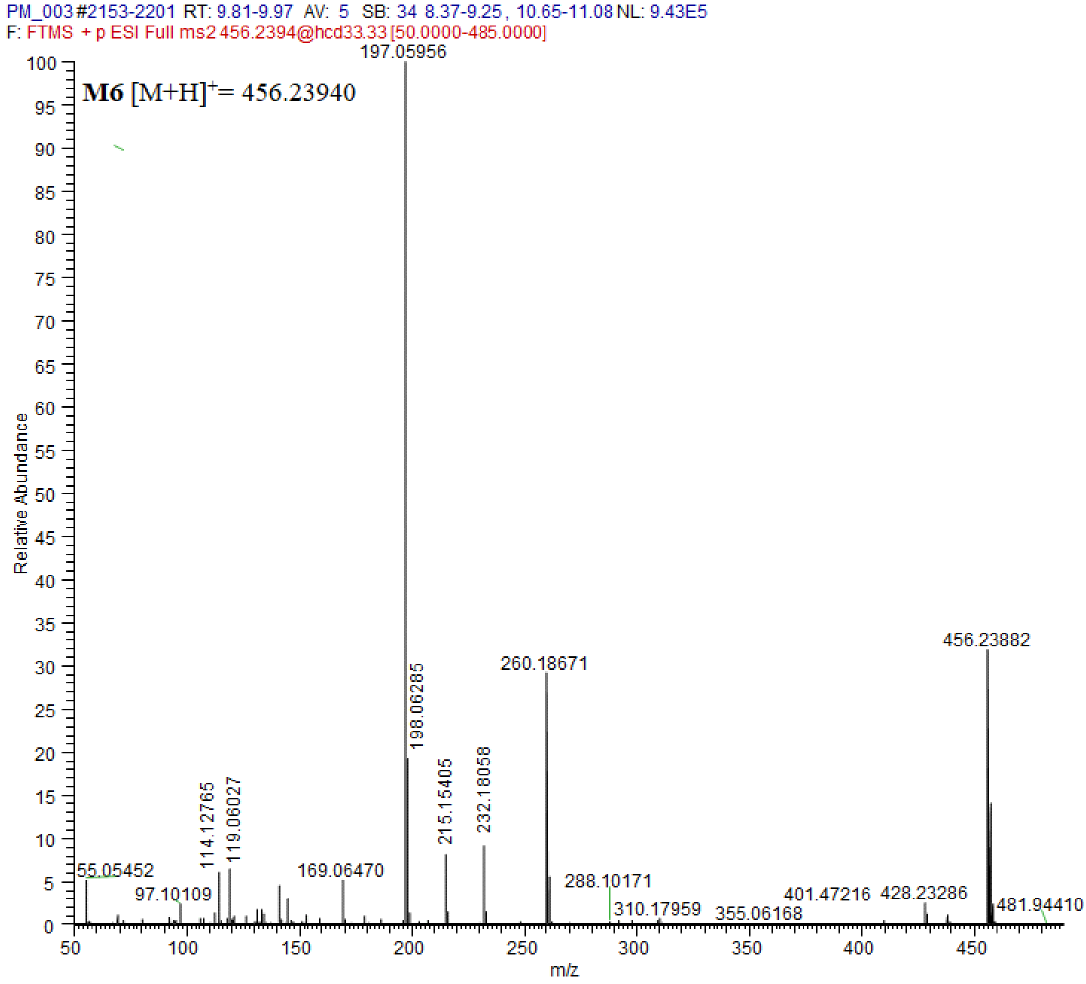

PM_003 #2153-2201 RT: 9.81-9.97 AV: 5 SB: 34 8.37-9.25 , 10.65-11.08 NL: 9.43E5
F: FTMS + p ESI Full ms2 456.2394@hcd33.33 [50.0000-485.0000]
M6 [M+H]+= 456.23940
Relative Abundance
m/z
197.05956
456.23882
260.18671
198.06285
215.15405
232.18058
114.12765
119.06027
55.05452
97.10109
169.06470
288.10171
310.17959
355.06168
401.47216
428.23286
481.94410

- **M7**

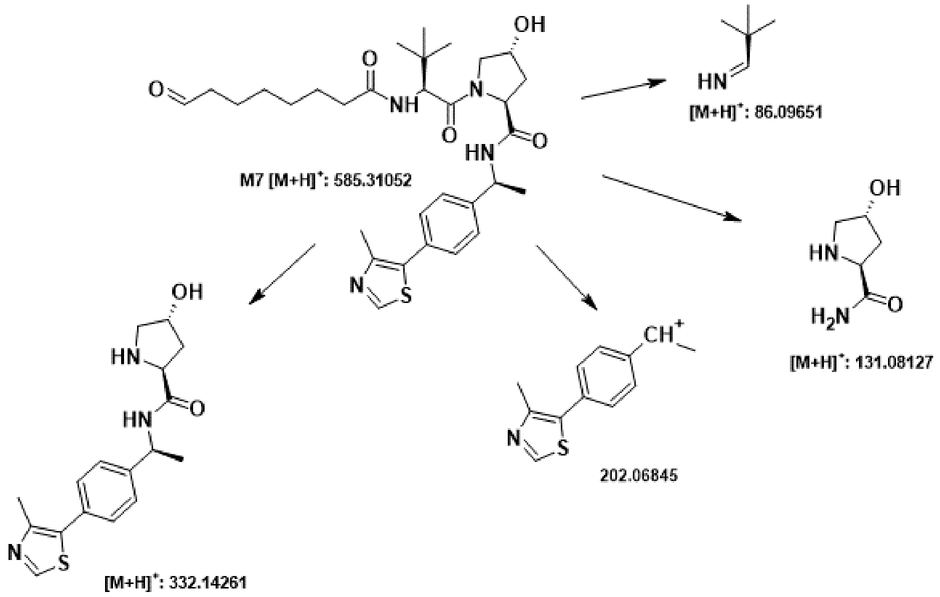

M7 [M+H]+: 585.31052
[M+H]+: 86.09651
[M+H]+: 131.08127
202.06845
[M+H]+: 332.14261

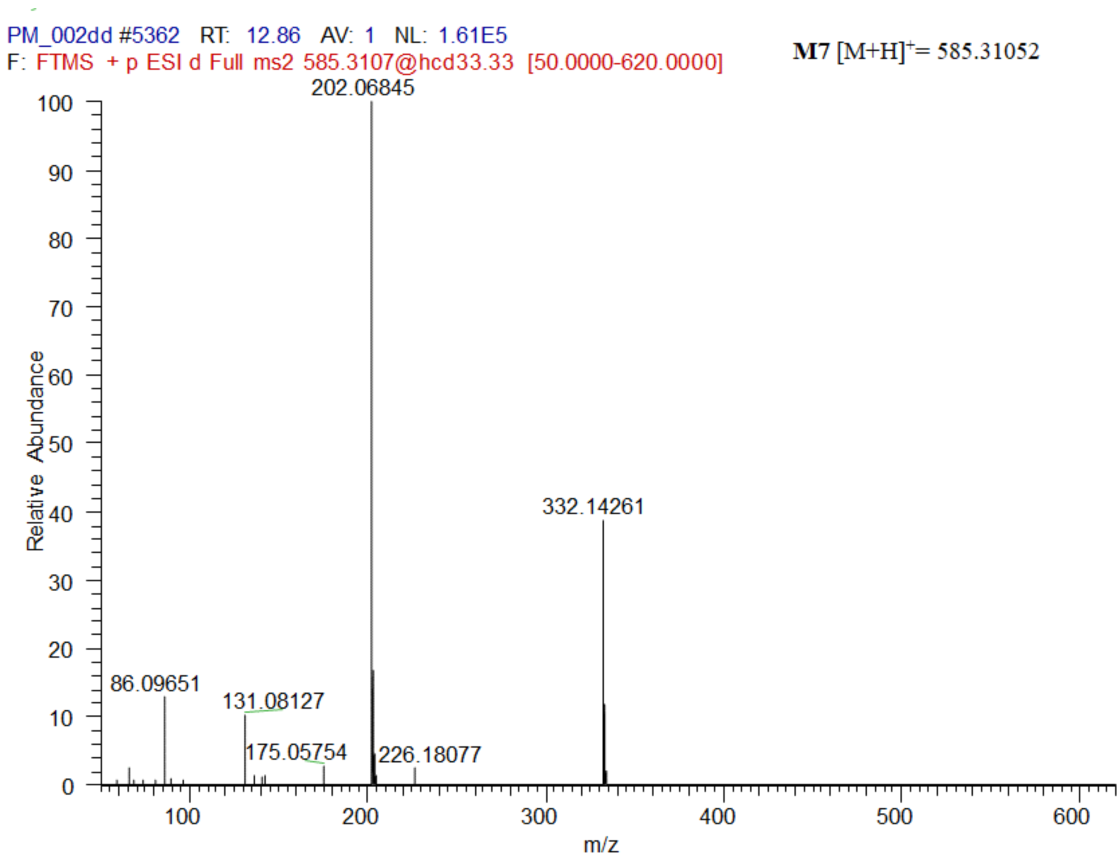

PM_002dd #5362 RT: 12.86 AV: 1 NL: 1.61E5
F: FTMS + p ESI d Full ms2 585.3107@hcd33.33 [50.0000-620.0000]
M7 [M+H]+= 585.31052
202.06845
332.14261
86.09651
131.08127
175.05754
226.18077
Relative Abundance
m/z

- **M8**

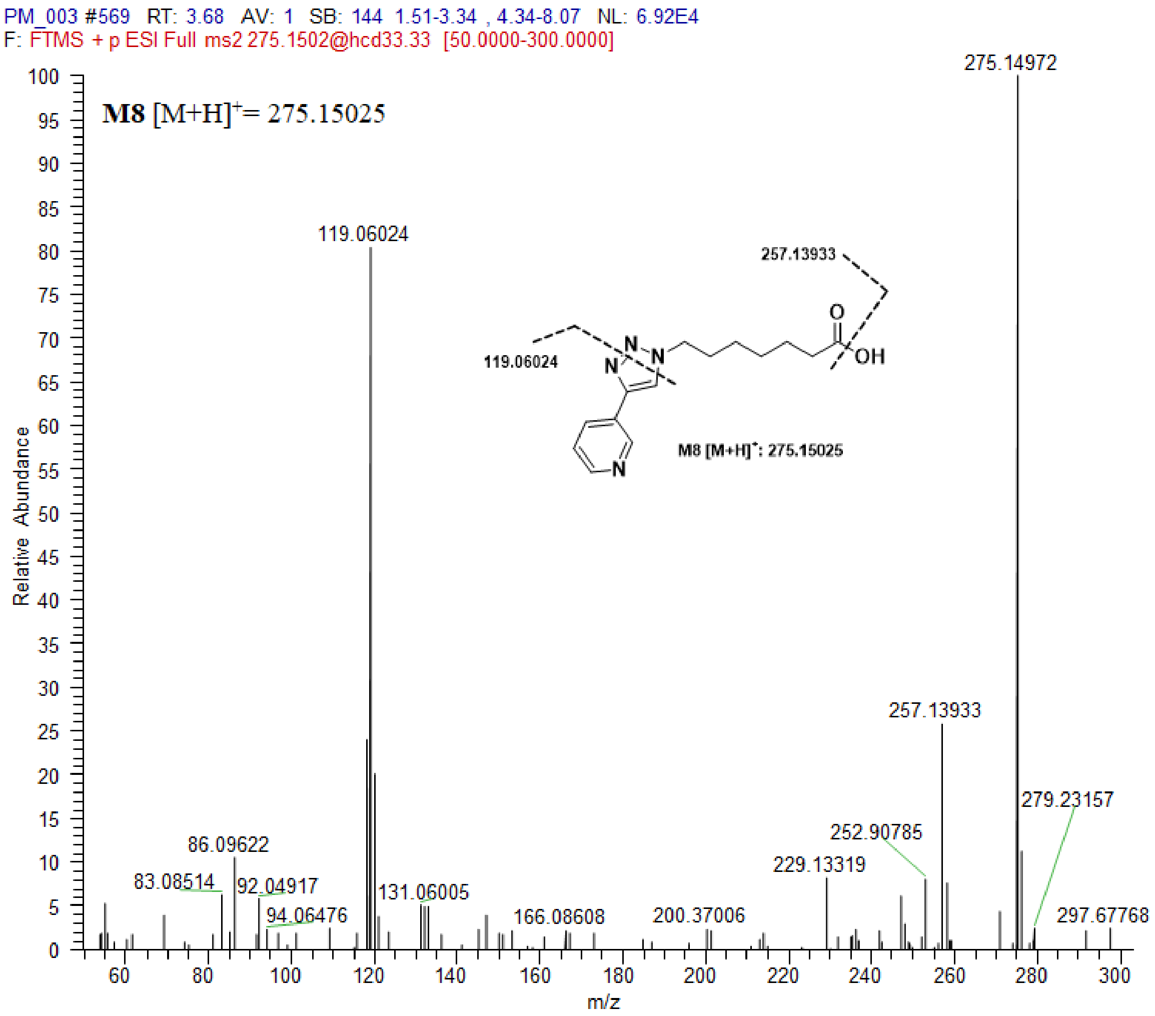
PM_003 #569 RT: 3.68 AV: 1 SB: 144 1.51-3.34 , 4.34-8.07 NL: 6.92E4
F: FTMS + p ESI Full ms2 275.1502@hcd33.33 [50.0000-300.0000]
M8 [M+H]+= 275.15025
275.14972
119.06024
257.13933
279.23157
252.90785
229.13319
86.09622
83.08514
92.04917
94.06476
131.06005
166.08608
200.37006
297.67768
119.06024
257.13933
O
OH
N
N
N
N
M8 [M+H]+: 275.15025
Relative Abundance
m/z

- **M9**

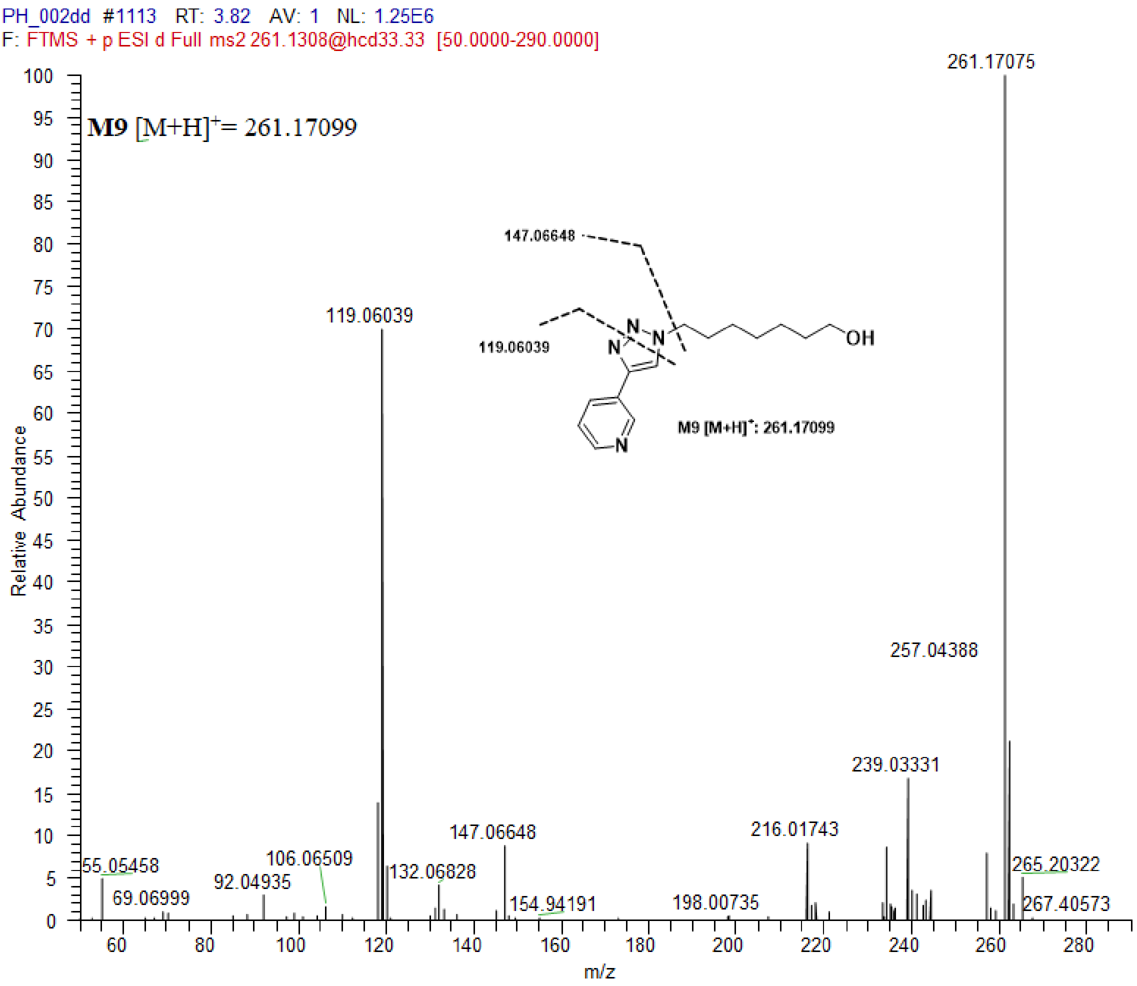
PH_002dd #1113 RT: 3.82 AV: 1 NL: 1.25E6
F: FTMS + p ESI d Full ms2 261.1308@hcd33.33 [50.0000-290.0000]
M9 [M+H]+= 261.17099
261.17075
119.06039
257.04388
239.03331
147.06648
216.01743
106.06509
92.04935
132.06828
55.05458
69.06999
154.94191
198.00735
265.20322
267.40573
147.06648
119.06039
OH
N
M9 [M+H]+: 261.17099
Relative Abundance
m/z

- **M10**

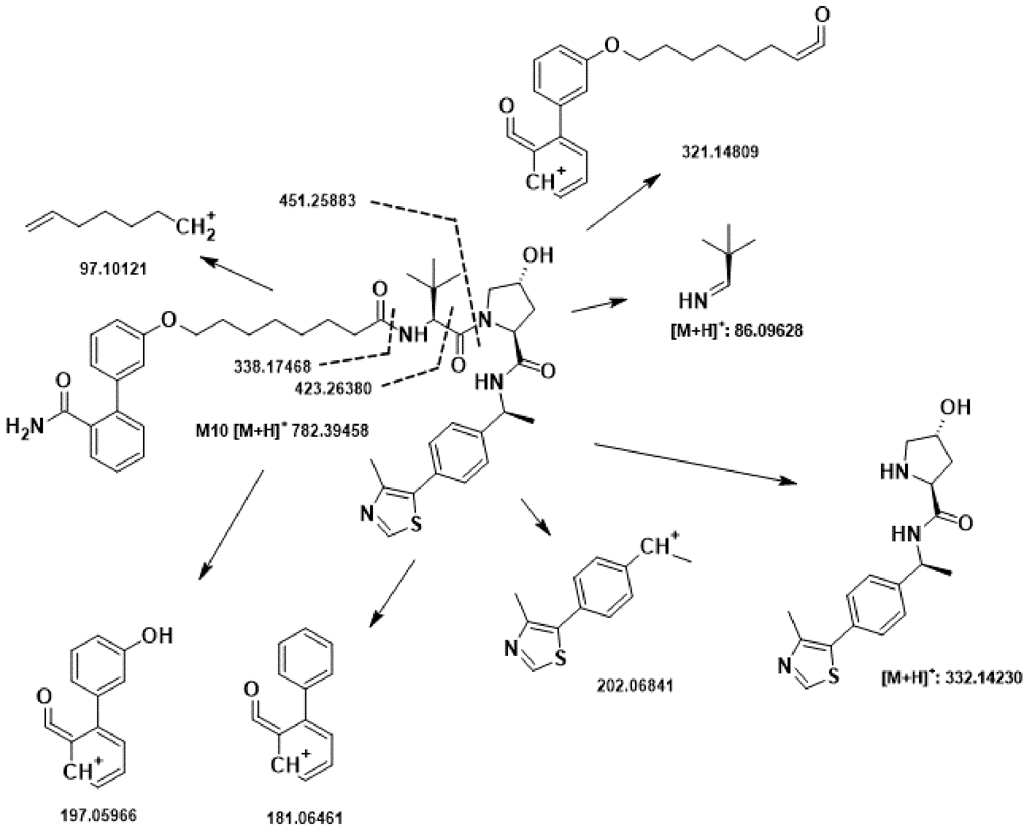
321.14809
451.25883
97.10121
338.17468
423.26380
M10 [M+H]+ 782.39458
[M+H]+: 86.09628
202.06841
[M+H]+: 332.14230
197.05966
181.06461

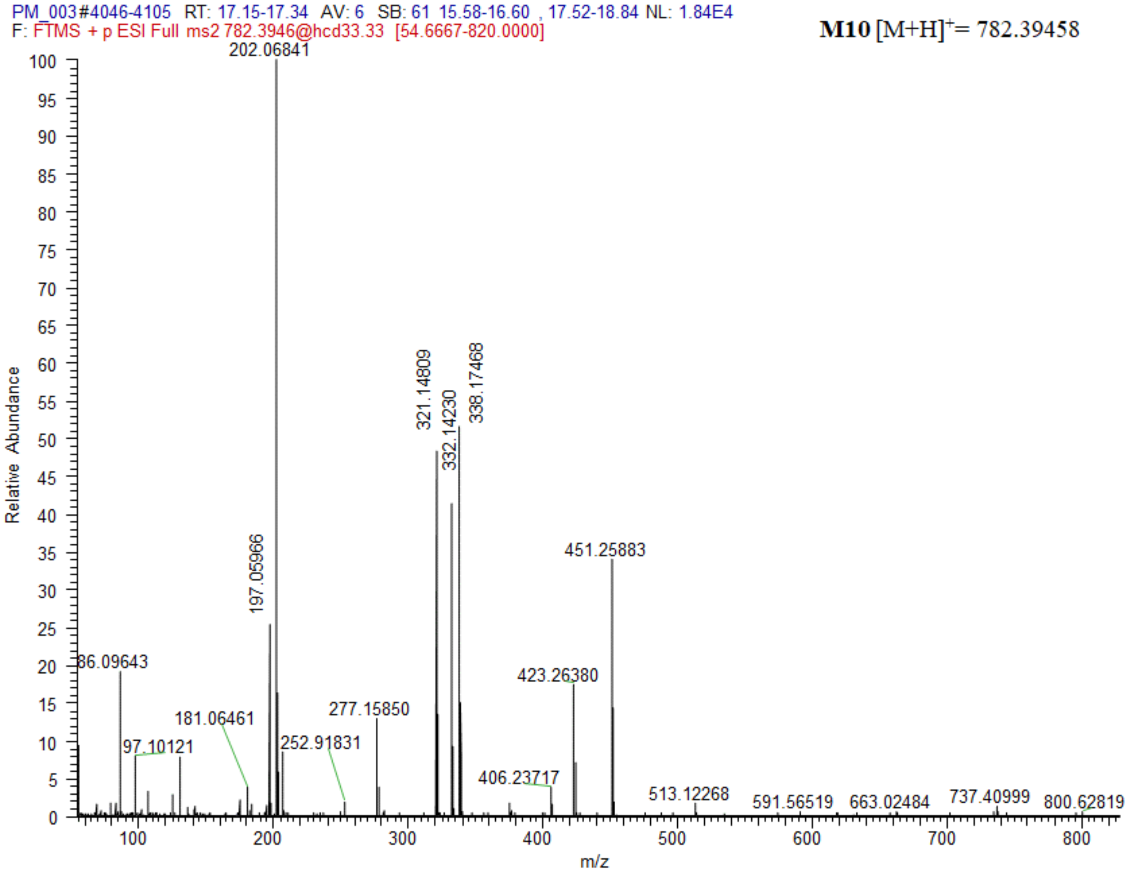
PM_003#4046-4105 RT: 17.15-17.34 AV: 6 SB: 61 15.58-16.60 , 17.52-18.84 NL: 1.84E4
F: FTMS + p ESI Full ms2 782.3946@hcd33.33 [54.6667-820.0000]
M10 [M+H]+= 782.39458
Relative Abundance
m/z
202.06841
321.14809
332.14230
338.17468
451.25883
197.05966
86.09643
423.26380
181.06461
277.15850
97.10121
252.91831
406.23717
513.12268
591.56519
663.02484
737.40999
800.62819

- **Positive full-mass chromatograms**

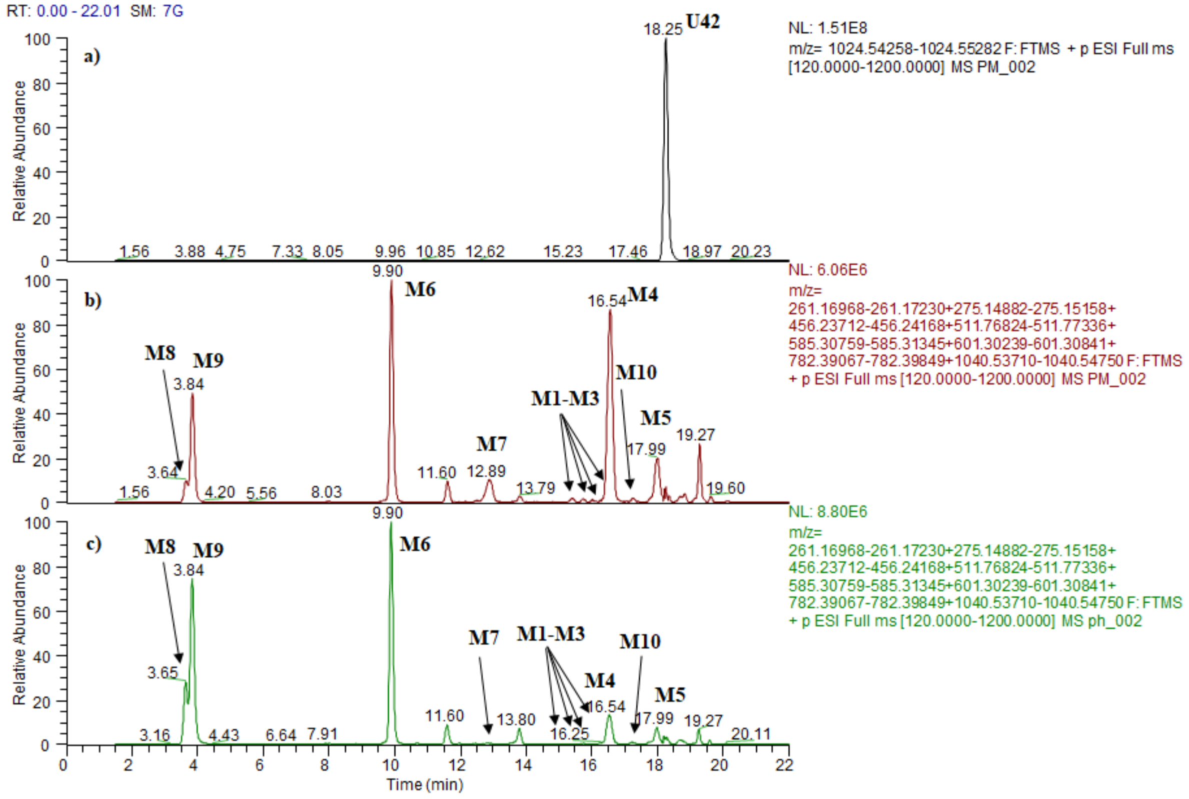

a) mass range $[M+H]^+$: **U42**

b) mass range $[M+H]^+$: metabolites in mouse liver microsomes

c) mass range $[M+H]^+$: metabolites in luman liver microsomes
